# Coordination-cage-templated multivalent glycan arrays: effects of mixed-glycan composition and chain length on lectin binding

**DOI:** 10.1039/d6sc01981a

**Published:** 2026-09-28

**Authors:** Callum L. Pritchard, Melissa Ligorio, Filip Kusnir, Matthew I. Gibson, Michael D. Ward

**Affiliations:** a Department of Chemistry, University of Warwick Coventry CV4 7AL UK m.d.ward@warwick.ac.uk; b Department of Chemistry, Manchester Institute of Biotechnology, University of Manchester UK matt.gibson@manchester.ac.uk

## Abstract

The multivalent display of glycans in glycoclusters is typically achieved on geometry- and valency-disperse scaffolds such as polymers or nanoparticles, bearing a single glycan type; but the less explored heterogeneous display of multiple glycan types in a cluster may allow access to more avid or multifunctional glycomaterials. Here we deploy two distinct synthetic strategies to obtain self-assembled M_8_L_12_ coordination cages bearing 24 glycan residues of two distinct types, pendant from a strictly monodisperse, structurally predictable coordination cage scaffold formed in a single self-assembly step. Turbidimetry measurements revealed that the heterogeneous glycan displays result in significant differences in how the cages bind to representative lectins compared to homo-glycan arrays based on the same cages, underlining the value of structurally predictable, programmable, coordination cages as scaffolds which give access to increasingly complex glycomaterials, offering precise control over heterogeneous glycan displays which is not possible with other materials-led approaches. In addition we have examined the effect of increasing chain length (connecting cage core to pendant glycan array) and have found significant changes in lectin binding affinities with spacer length which will inform the design of next-generation glyco-cages.

## Abstract 2

Coordination cages are used as scaffolds to assemble monodisperse glycoclusters of 24 glycan units incorporating mixtures of glycans, or varying spacer lengths, to determine effects on lectin binding.

## Graphical abstract

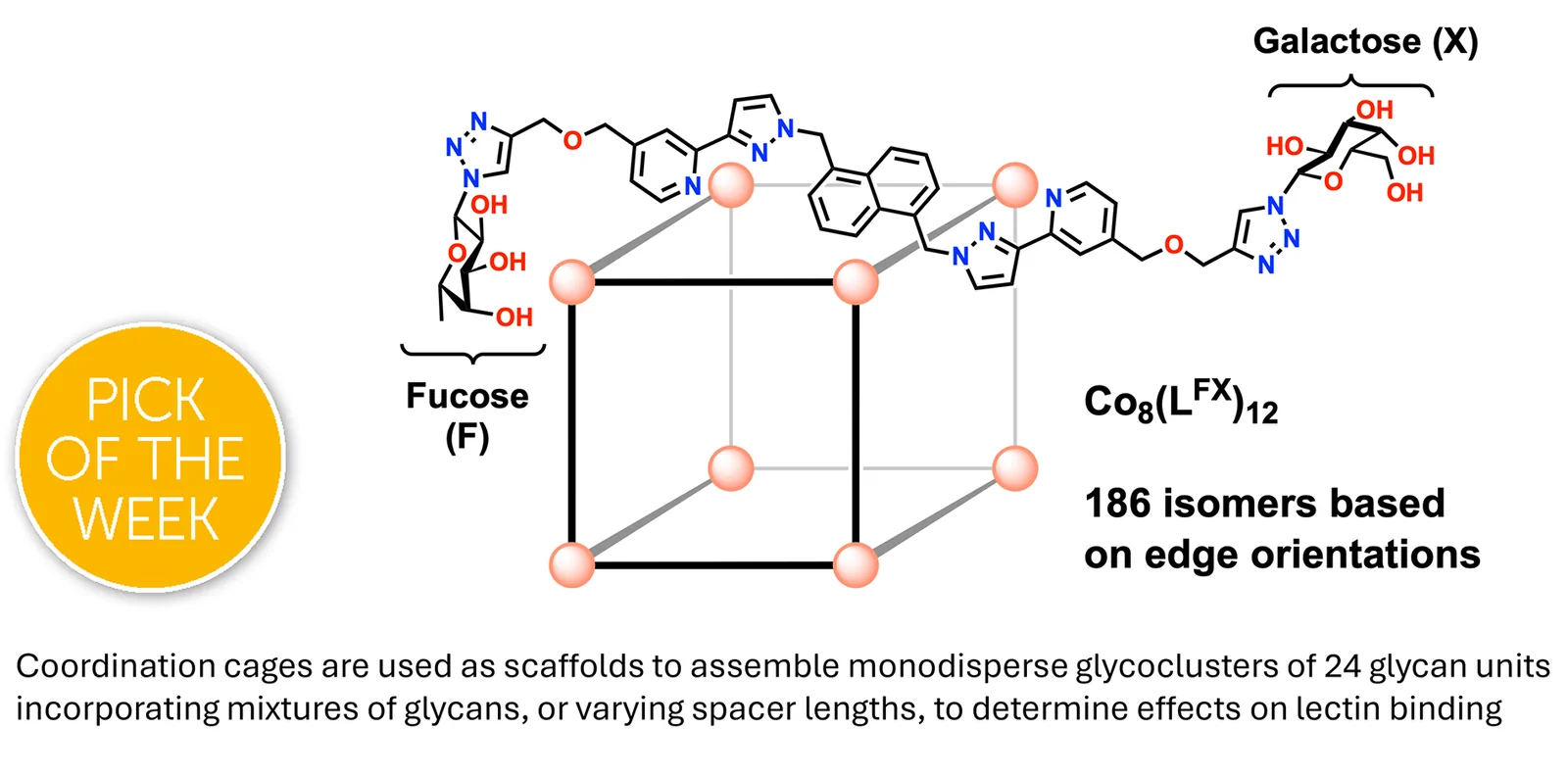

## 1 Introduction

High-affinity binding between components in a biological context often requires the cooperative interaction of multiple binding interactions.^1^ A particularly important class of these is the binding between sugar residues (glycans) and the proteins to which they bind (lectins) which, whilst individually weak, become strong when multiple glycan units are involved: the cluster glycoside effect.^2^ A wide range of cell signalling, communication and regulation process are mediated by (multi)-glycan/lectin interactions,^3^ and this in turn means that synthetic multi-glycan arrays are of particular interest:^4^ such compounds have been extensively investigated based on dendrimers, polymers and nanoparticles bearing multiple glycan pendant groups.^5^

Strict monodispersity and precise geometric control of a glycan array in a molecule are difficult to achieve^5*g*^ using, for example, polymers and nanoparticles as supports for the glycan arrays, with obvious problems for predictability of binding and determining structure/affinity relationships. Accordingly we^6^ and others^7^ have recently been interested in the use of self-assembled coordination cages as scaffolds for formation of monodisperse and geometrically predictable multi-glycan arrays. The fact that a large number of relatively simple bridging ligands can combine with metal ions into a highly symmetric polynuclear metal/ligand assembly^8^ provides a synthetically convenient basis to make such multi-glycan arrays, by decorating the ligands with a small number of pendant glycans and then letting the self-assembly process with metal ions dictate the formation of the multi-glycan array. The use of metal complex based self-assembly to organise multiple glycan-appended units in this way is an example of what has been termed ‘self-assembly multivalency’,^9^ and it is still the case that there are very few examples, with the most recent examples coming from ourselves^6^ and Stauber and co-workers.^7^

In a recent paper we reported how M_4_L_6_ and M_8_L_12_ coordination cages – based on tetrahedral and cubic metal-ion arrays, with ditopic bridging ligands connecting a pair of metal ions along every edge – could be used as the basis for formation of glycoclusters containing (respectively) 12 or 24 pendant glycan units, with one glycan unit pendant from the pyridyl ligand at either end of the bridging ligand.^6^ A wide range of mono- or tri-saccharides was used to form the outer glycan layer, with clear evidence of a cluster glycoside effect being apparent in that the 24-glycan assemblies showed stronger binding to their partner lectins than did the 12-glycan assemblies. Overall this cage family presents opportunities for precision analysis of binding interactions with proteins, and the optical/photophysical characteristics of the cages also present opportunities for their use in selective sensing methods. In this contribution we extend that earlier work in two directions.

Firstly, and forming the major part of this paper, we report the preparation and study of mixed-glycan arrays. A deliberate limitation of previous work – and implicit in related work by others – is that all glycan units within an assembly are of the same type: the rationale for this is obvious for (a) synthetic simplicity and (b) limiting the number of variables in a particular study. There are countless such homo-glycan glycoclusters. We are interested, however, to extend our self-assembly-based synthetic methodology further to more complex mixed-glycan arrays. Incorporating a mixture of glycan units in a hetero-glycocluster array constitutes a substantial step towards modelling the complexity of real glycocalyx layers,^10,11^ and it has become apparent that such assemblies can behave substantially differently from homo-glycan arrays in their binding to target lectins. This includes, for example, a hetero-cluster effect whereby a mixed-glycan array can bind to a lectin target more strongly than does an isostructural homo-glycan array, even when the secondary glycan residues are not on their own good ligands for that lectin, due to a range of cooperative and (often loosely-defined) synergistic effects arising from the availability of a mixture of glycans.^11^ Another issue is that some lectins are not highly specific for a single glycan type but can show a degree of promiscuity, implying that mixed-glycan arrays have value for probing glycocluster binding to such lectins. The development of synthetic routes to such mixed-glycan assemblies – especially with control over composition and 3-D arrangement of the assemblies – is therefore of particular interest.^10^ Accordingly in the first part of this work we demonstrate for the first time how the principle of self-assembly multivalency using coordination cages as scaffolds^6,7,12^ can be extended to prepare two different types of mixed-glycan array: we observe in some cases differences in the ability of the mixed-glycan arrays to induce aggregation of lectins compared to each other and compared to their simpler homo-glycan analogues.

Secondly, we have also investigated the effect of linker length (between cage core and pendant glycan array) on lectin-binding behaviour when the composition of a cage-bound glycan array does not change. This has been done by including an additional PEG-type chain between the M_8_L_12_ cage core and the 24 (identical) pendant glycans. We demonstrated earlier that there was a clear cluster glycoside effect in our glycocages, with the larger cages – bearing more pendant glycans – showing greater avidity for lectin binding:^6^ as a complement to this, here we demonstrate that this additional length (and flexibility) of the spacers has significantly different consequences for glycocluster/lectin recognition with different glycan/lectin combinations.

Overall the new synthetic strategies reported here add substantially to the scope and complexity of recent work on the use of metal coordination cages to generate elaborate multi-valent mixed-glycan arrays with tailored properties.^6,7,12^

## 2 Results and discussion

### 2.1. Design and composition of mixed-glycan complexes

We have developed two different routes to assemble 24-glycan arrays, based on the M_8_L_12_ octanuclear cubic cage core as the scaffold,^13^ that are in essence isostructural with complexes reported previously^6^ – but which contain two different types of glycan in the shell surrounding the cage core. Based on the glycan set glucose (abbreviated G), fucose (abbreviated F) and galactose (abbreviated X) we have prepared three types of mixed-glycan assembly based on F/G, F/X or G/X combinations whose binding to lectins may be compared with each other and with the homo-glycan complexes containing all-G, all-F or all-X glycan arrays.

The two synthetic strategies (Scheme 1) lead to different degrees of complexity in the ensembles. The first synthetic approach (type A) is based on the preparation of non-symmetrical edge-bridging ligands such as L^FX^ (and likewise L^GX^) in which each terminus of the ligand is now appended with a different glycan following a stepwise synthetic process during which each glycan is appended in turn to the ligand core. Assembly of a non-symmetrical ligand of this type with M(II) ions forms the complexes M_8_(L^YZ^)_12_ (where Y and Z are generic labels denoting different glycan types) whose glycan shell has a single composition containing exactly 12 each of the glycan residues Y and Z: but, given that the orientation of each ligand along its M⋯M edge is random, M_8_(L^YZ^)_12_ exists as 186 possible isomers, each with a different spatial disposition of the two glycan types.††This arises from application of Burnside's lemma (see https://en.wikipedia.org/wiki/Burnside%27s_lemma). We consider that each edge of a cube has an arrow pointing one way or the other to denote the loss of symmetry associated with two different glycan termini on each edge-bridging ligand. It is necessary to consider each type of rotational symmetry of a cube and determine how many arrangements of edges each symmetry operation allows; the number of distinct arrangements (cage isomers) is then the average size of that fixed set. For a cube we have five different rotational symmetry operations: (i) 1× identity which allows all 212 orientational possibilities of the ligand set. (ii) 6 × 90° rotations about the axes through the centre of an opposed pair of faces: there are three such axes, with +90° and −90° rotations around each. For a face to be allowed to rotate by 90° we have the constraint that all four arrows along the edges must have the same orientation, since each rotates into the next, so each of two faces can be considered ‘clockwise’ or ‘anticlockwise’ (=4 possibilities). Likewise the set of four edges linking the two faces must have its arrows all ‘up’ or ‘down’ (=2 more possibilities) to allow them to rotate into one another, so there are 8 ligand orientation possibilities in total for each of the three axes. (iii) 3 × 180° rotations about the same set of axes. For these to be allowed requires that six pairs of edges can interconvert; that means two orientational possibilities per pair, or 2^6^ for each of the three rotational operations. (iv) 8 × 120° rotations about axes going through two diagonally opposite corners (four such axes, with +120° and −120° rotations about each). Such a threefold rotation splits the cube into four sets of three edges that must be able to interconvert cyclically, so each set can be all ‘up’ or all ‘down’ in terms of arrow orientations, giving 2^4^ orientational possibilities per rotation. (v) 6 × 180° rotations about axes which bisect a diagonally opposite pair of edges: no such rotations are allowed however as the two edges transform into themselves which is not allowed given the sense of direction implied by an arrow. The average of these is 1/24[(1 × 2^12^) + (6 × 2^3^) + (3 × 2^6^) + (8 × 2^4^) + 0] = 186, which is the number of isomeric ligand arrangements in a type A mixed-glycan cage (ignoring the fact that the vertices are not all equivalent). There is accordingly a single, fixed glycan composition for each member of the ensemble but multiple (186) geometric arrangements of the units.

**Scheme 1:**
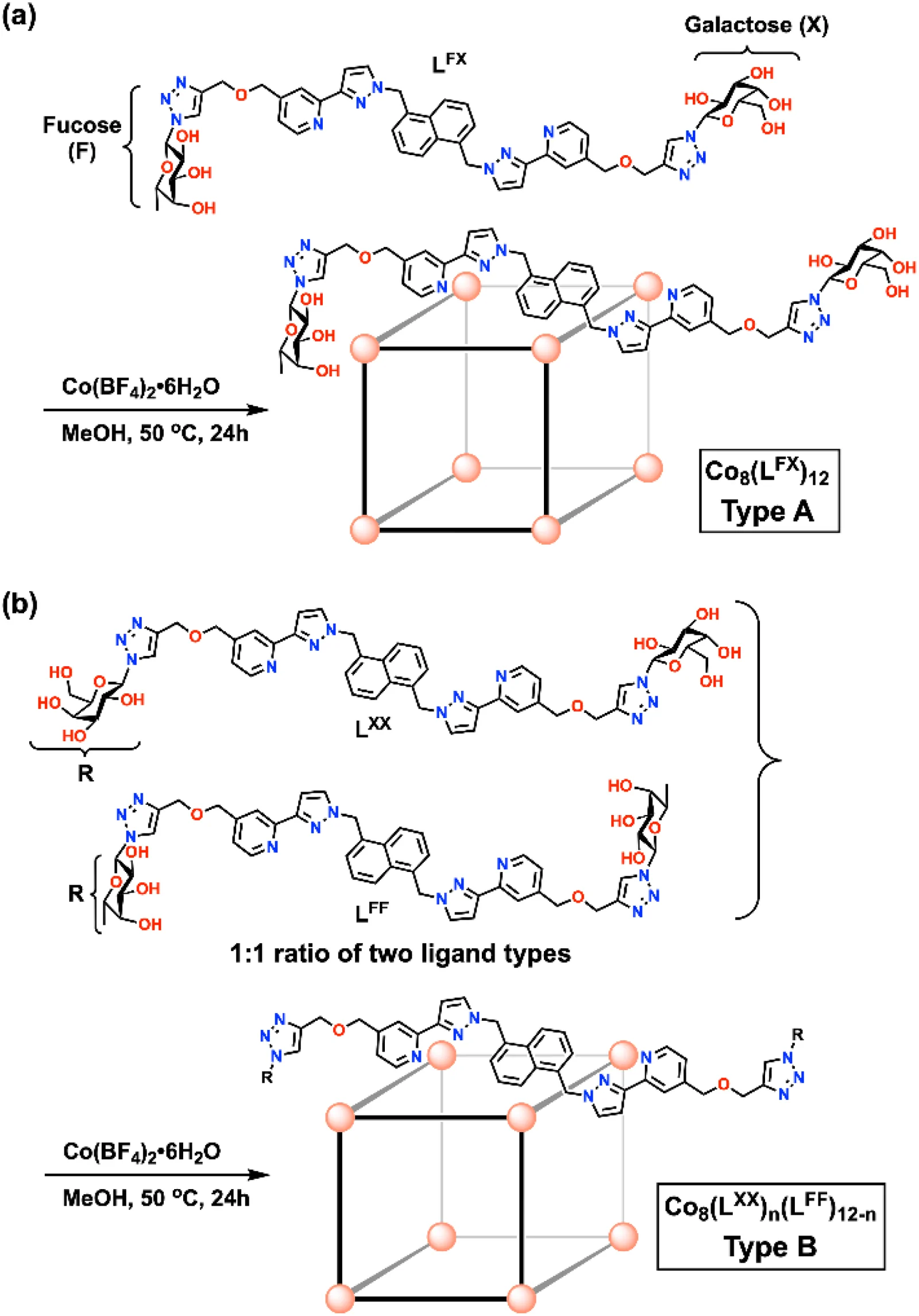
Sketches of the structures of (a) the type A cages M_8_(L^YZ^)_12_ containing 12 identical ligands which each contain a different glycan at each terminus (Y/Z = F/X or G/X) and (b) the type B cages M_8_(L^YY^)_*n*_(L^ZZ^)_12−*n*_ (Y, Z = F, G, X) containing a statistical mixture of two different homo-glycan ligand types.

The second synthetic approach (which we denote type B) introduces an additional degree of variability. A 1 : 1 mixture of two symmetrical ligands (denoted L^YY^ and L^ZZ^) is used as the basis of the self-assembly process such that 8 M(ii) ions are combined with six ligands of each type. This affords a statistical mixture of assemblies of composition M_8_(L^YY^)_*n*_(L^ZZ^)_12−*n*_. Overall there is necessarily a 1 : 1 ratio of the two types of glycan unit in the ensemble, but there will be thirteen different compositions of cage (*n* = 0–12), each containing a different proportion of L^YY^ and L^ZZ^ ligands. Within each composition there will be (for all but the most trivial cases of *n* = 0 or 1) multiple isomers: giving a total ensemble containing 218 distinct compounds‡‡For the statistical mixed-ligand assemblies (type B) M_8_(L^YY^)_*n*_(L^ZZ^)_12−*n*_, where Y and Z are different glycans but each ligand is now symmetrical (both glycan termini are the same in each ligand), the number of isomers for each composition is as follows (for *n* = 0–12): 1, 1, 5 (see Fig. 1), 13, 27, 38, 48, 48, 27, 13, 5, 1, 1, giving a total of 218 unique species in the ensemble, again ignoring that all vertices of the cage are not equivalent. based on a mixture of compositions as well as multiple isomers for most of the compositions.

### 2.2. Synthesis and characterisation of new ligands for mixed-glycan cages

Ligands L^GG^ and L^XX^, bearing (respectively) a glucose residue (G) or a galactose residue (X) pendant from each of the terminal pyridyl rings of the bridging ligand, were reported earlier.^6^ The new ligand L^FF^ (used in some of the type B complexes) was made using an identical strategy (Scheme 2, left). The doubly alkyne-appended ligand L^(15-CC)^ was subjected to Cu-AAC ‘Click’ reaction conditions with β-l-fucopyranosyl azide triacetate^14^ to yield the per-acetyl-protected ligand L^FF-Ac^ in 78% yield after purification by size-exclusion chromatography. Removal of the acetyl protecting groups in 95% yield was then achieved under Zemplén conditions (NaOMe/MeOH)^15^ to afford L^FF^.

**Scheme 2:**
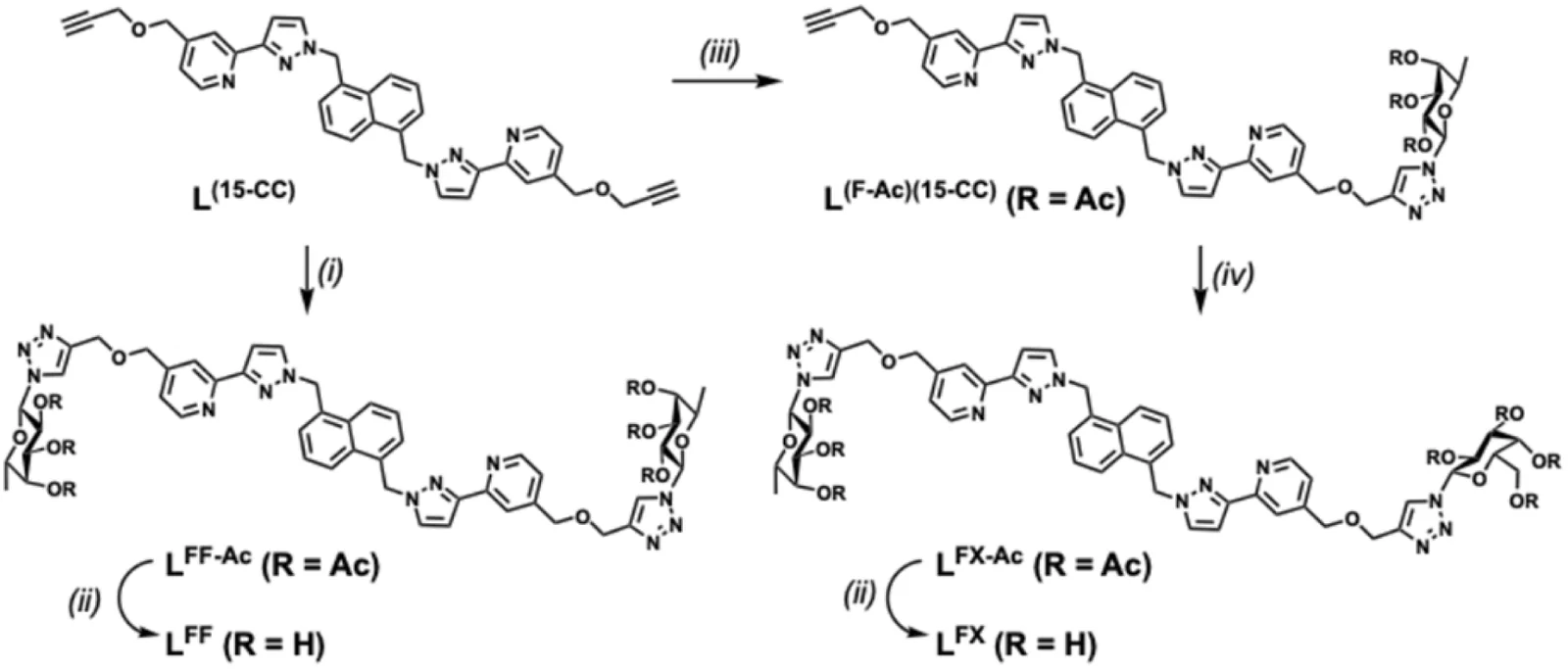
Syntheses of symmetric ligand L^FF^ (left) and (ii) non-symmetrical ligand L^FX^ (right). Reagents: (i) β-l-fucopyranosyl azide triacetate (excess); (ii) NaOMe/MeOH; (iii) β-l-fucopyranosyl azide triacetate (<1 equiv.); (iv) 1-azido-1-deoxy-β-d-galactopyranoside tetraacetate.

Asymmetric ligands L^GX^ and L^FX^, bearing a different glycan at each terminus and used to prepare the type A complexes, required a stepwise synthetic procedure in which each glycan unit is added in turn to the L^(15-CC)^ core: this could be achieved by control of the ratio of reactants at each step. Thus reaction of L^(15-CC)^ with a sub-stoichiometric amount (0.83 equiv.) of 1-azido-1-deoxy-β-d-glucopyranoside tetraacetate under the same Cu-AAC ‘Click’ reaction conditions as before^6^ allowed isolation of the intermediate L^(G-Ac)(15-CC)^, with one unreacted alkyne group and one appended (acetyl-protected) G residue, in 93% yield. A second Cu-AAC ‘Click’ reaction with 1-azido-1-deoxy-β-d-galactopyranoside tetraacetate resulted in incorporation of the second glycan to give the protected ligand L^GX-Ac^ in 87% yield, which was then deacetylated as before to afford L^GX^. The other mixed-glycan asymmetric ligand L^FX^ was prepared similarly (see Scheme 2).

Detailed ^1^H NMR spectroscopic, and high-resolution mass spectrometric data for the new ligands are presented in the SI along with the full experimental methods.

### 2.3. Synthesis and characterisation of mixed-glycan cages

All mixed-glycan cages were prepared using the standard methodology that we have regularly used before,^13^ by combining in aqueous MeOH the required M(BF_4_)_2_ salt with the relevant bis(pyrazolyl-pyridine) ligand (or, for type B cage complexes, a 1 : 1 mixture of two differently-substituted ligands) in proportions ensuring an overall 8 : 12 ratio of metal ions to ligands, as required for cage assembly. After 24 h of stirring at 323 K, evaporation of solvents followed by chromatography on Sephadex® G-50 was used to effect purification. Using this methodology the homo-glycan cage M_8_(L^FF^)_12_ was prepared to supplement the existing M_8_(L^GG^)_12_ and M_8_(L^XX^)_12_ cages from our earlier work.^6^

The type A cages based on the non-symmetrical ligands each contain a single cage composition but with multiple different isomers arising from different orientations of the ligands L^FX^ and L^GX^ along the edges. The ^1^H NMR spectra of these new glyco-cages (see SI) are entirely consistent with previously-reported NMR spectra of cages of this type and confirm the cages' formation.^13^§§The spectra of the paramagnetic Co(ii) cages are highly diagnostic. Signals are broad because of the paramagnetism, which spreads signals over a range of nearly 200 ppm, and also because of the slow tumbling of the large molecules in a viscous solvent (D_2_O).^13*c*^ However the pattern of signals is very distinctive with the *S*_6_ symmetry of the metal complex core resulting in two independent ligand environments, each having no internal symmetry,^13^ which means that each proton type within a ligand (*e.g*. pyridyl H^6^) occurs in four distinct but similar environments in the assembled cage. Assignment of NMR signals of the unsubstituted Co(ii) cage – as far as is possible given absence of any ^1^H–^1^H coupling information – was reported in the SI of ref. 13d, based on correlation of peak widths (hence, *T*_1_ values) with crystallographically-determined H⋯Co separations. The 0–10 ppm region of the NMR spectra of these glyco-cages is dominated by the glycan signals, which are remote from the paramagnetic centres and hence lie in the normal chemical shift region. Beyond this however the more shifted cage signals outside the 0–10 ppm region agree very closely with previously reported Co_8_L_12_ cages of this type, including some crystallographically-characterised examples,^13^ and also including our recently-reported glycan-substituted cages such as Co_8_(L^GG^)_12_.^6^ The peripheral glycans are too remote from the cage core for the different isomeric glycan arrangements to influence the NMR signals of the cage core, which remain consistent with local *S*_6_-symmetry, diagnostic of cage assembly.^13^ The high-resolution ES mass spectra likewise confirmed the cage compositions as Co_8_L_12_ (L = L^FX^ and L^GX^) with a set of signals in each case for differently-charged species based on loss of varying numbers of tetrafluoroborate anions. Dynamic light scattering (DLS) measurements (see SI) showed the presence of species with a diameter consistent with the expected size of isolated cages (20–30 Å) with traces of some larger aggregates also present, a known behaviour with large glycoclusters.^7*c*,16^

The more complex type B ensemble is a family of cages: a statistical mixture of assemblies Co_8_(L^YY^)_*n*_(L^ZZ^)_12−*n*_ (with *n* = 0–12; Y and Z denote any glycan). It was prepared by combining a 1 : 1 mixture of two different homo-glycan ligands L^YY^ and L^ZZ^ with Co(BF_4_)_2_·6H_2_O in a 4 : 3 : 3 ratio in MeOH as required for exact self-assembly of the M_8_L_12_ core. This is again the standard method for cage preparation,^13^ but the novelty here lies in the 1 : 1 mixture of different ligands bearing different glycan pendants. As mentioned earlier, with the exception of the trivial cases with *n* = 0 or 1, there are multiple isomers for each composition to a total of 218 species in the ensemble.

Fig. 1 illustrates the set of five isomers for *n* = 2 (or 10), of which two are enantiomers of one another; given the chiral environment surrounding the cage core (the glycan array) these are diastereoisomers so all five isomers can be considered as physically distinct. The maximum number of isomers for a single composition (*n* = 6) is 48. Note that this calculation is just based on the assumption that the underlying cage core has cubic symmetry with all edges the same; in fact the M_8_L_12_ cage is not a regular cube but has *S*_6_ symmetry with two different edge environments,^13^ but for the purpose of the calculations in the footnote to illustrate the number of glycan arrangements,^†^ this has been ignored.

**Fig. 1:**
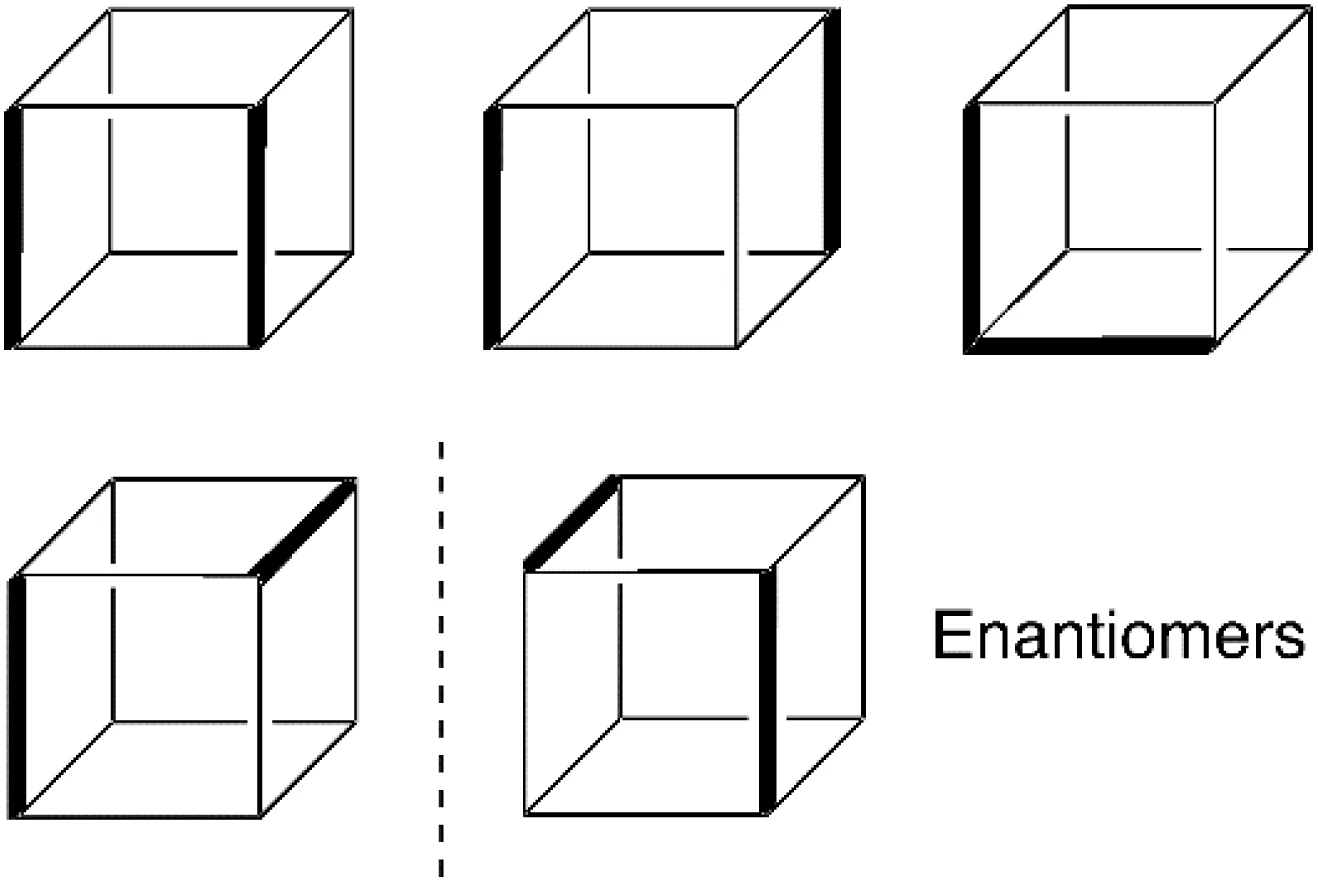
Five different arrangements of ligands in the M_8_ core of a mixed-ligand M_8_(L^YY^)_*n*_(L^ZZ^)_12−*n*_ cage (with *n* = 2 or 10).

The ^1^H NMR spectrum of each type B ensemble Co_8_(L^XX^)_*n*_(L^YY^)_12−*n*_ provides, as expected, no information on the distribution of compositions within the sample (which is expected to be binomial). However high-resolution ESI-MS spectra are more informative and confirm the expected presence of multiple species based on differing masses of different glycan units.

For example: in the ensemble Co_8_(L^FF^)_*n*_(L^GG^)_12−*n*_ (Fig. 2) the masses of L^FF^ (957.02) and L^GG^ (989.02) differ by 32 units. For each charge state, *e.g.* within the collection of peaks corresponding to [{Co_8_(L^FF^)_*n*_(L^GG^)_12−*n*_}(BF_4_)_7_]^9+^ (arising from loss of 9 of the 16 tetrafluoroborate anions from the intact neutral complex), the envelope of signals around *m*/*z* 1413 contains multiple components separated by *m*/*z* 3.56 which corresponds to the expected difference arising from adjacent values of *n* in the cage composition (9 × 3.56 = 32.0). The most intense component of the signal set for the species with charges of 7+, 8+ and 9+ corresponds to the composition with *n* = 5: *i.e.* five L^FF^ ligands and seven L^GG^ ligands appears to be marginally the most abundant cage species, rather than *n* = 6, though this may not be significant given the absolute signal intensities. The species with *n* from 1 to 10 could be discerned above the background noise. The similar type B cage ensembles Co_8_(L^YY^)_*n*_(L^ZZ^)_12−*n*_ were prepared based on 1 : 1 mixtures of L^FF^ + L^XX^ and L^GG^ + L^XX^ ligands, and their ES mass spectra likewise showed the presence of the expected mixture of compositions.

**Fig. 2:**
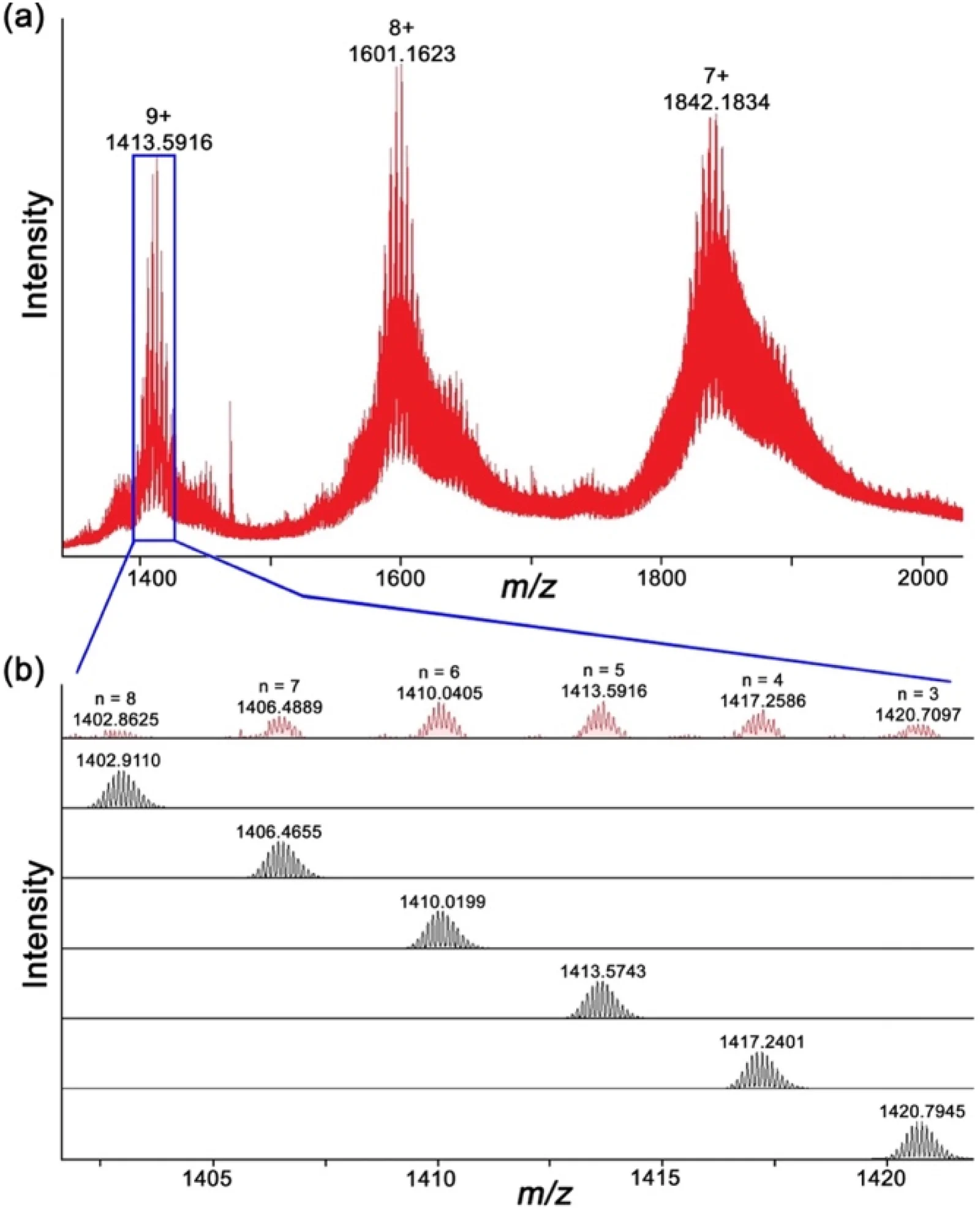
HR ESI mass spectrum of Co_8_(L^FF^)_*n*_(L^GG^)_12−*n*_ showing (a) peak clusters arising from loss of 7, 8 and 9 [BF_4_]^−^ anions, and (b) an expansion of the 9+ peak cluster corresponding to the composition [{Co_8_(L^FF^)_*n*_(L^GG^)_12−*n*_}(BF_4_)_7_]^9+^showing (top line in red) expansions of the measured signal components and (underneath, in black) the calculated spectra for different values of *n* as the glycan composition changes.

### 2.4. Interaction of the mixed-glycan cages with lectins

#### 2.4.1 General methodology

For these studies we used the two plant-based lectins Jacalin and *Ulex Europaeus* Agglutinin (UEA-I). Jacalin is known to have high affinity for β-d-galactose^17^ (which we denote X), though its binding site has sufficient plasticity for it to also bind other monosaccharides, albeit more weakly.^18^ UEA-I in contrast is selective for α-l-fucose (F) moieties.^19^

Interactions of the various multivalent cages with lectins were investigated using the solution-phase aggregation assay turbidimetry.^20^ The multivalency of the cages – and the rigid, pseudo-spherical shape of the cores – means that they can crosslink the lectins resulting in an increase in turbidity of the solution as aggregation occurs. This turbidity increase could be monitored by an increase in optical absorbance at 420 nm (*A*_420_),^6^ which signals lectin/cage binding. Thirty minutes after addition of the lectin to the cage, when aggregation is complete and the increase in optical density has plateaued, an excess of the relevant free competing glycan (d-galactose or l-fucose as appropriate) is added to disrupt the aggregates, reducing the *A*_420_ value back towards the dis-aggregated level, which confirms the basis of the lectin/cage binding.

We note that turbidity is based on a range of factors including aggregation and crosslinking behaviour and the solubility of the resulting aggregates, and not solely the thermodynamic strength of an individual glycocage/lectin interaction: this requires techniques such as isothermal calorimetry or biolayer interferometry, which we found to be impractical in these systems.^21^¶¶By far the commonest method of quantifying lectin/glyco-polymer interactions is ITC, but this technique was hampered by rapid glyco-cage/lectin precipitation: to a large extent ITC is challenging here because turbidimetry is effective.^21^ High dilution can help to minimise precipitation but requires very high affinities to be able to extract meaningful ITC data and was not successful. We note also that we attempted bio-layer interferometry experiments with some cage/lectin combinations but as reported earlier these were unsuccessful due to high non-specific binding of the glyco-cages to the probe surface, possibly arising from the high charge on the cages (+16).^6^ The turbidimetry measurements reported here confirm that some real and significant effects in the mixed-glycan cages and the cages with different spacer lengths: quantifying them will need future investigation based on significant method development. Turbidimetry is nonetheless a convenient and widely-used qualitative indication of relative binding affinities to a specific lectin.^20^ In particular, differences between experiments using the same lectin under the same conditions do provide a basis for qualitative comparisons between the abilities of different cages to bind to that lectin.

#### 2.4.2 Galactose-containing cages: binding to Jacalin

As baseline measurements to evaluate the effects of mixed-glycan arrays on lectin binding, we firstly need information on the lectin binding abilities of homo-glycan cage complexes, which all bear 24 glycans of the same type. The turbidimetry data are summarised in Fig. 3: all measurements were done in triplicate and averaged, with close agreement between runs. Some of these measurements, showing the binding of M_8_(L^GG^)_12_ and M_8_(L^XX^)_12_ with Jacalin, were reported initially in our earlier paper.^6^ However Fig. 3 includes additional measurements, and to ensure consistency and comparability across the whole set of data, the previously-reported measurements with M_8_(L^GG^)_12_ and M_8_(L^XX^)_12_ have been repeated in this work under the same conditions as the new measurements. Fig. 4 shows the same data but separated into two sets for clarity.

**Fig. 3:**
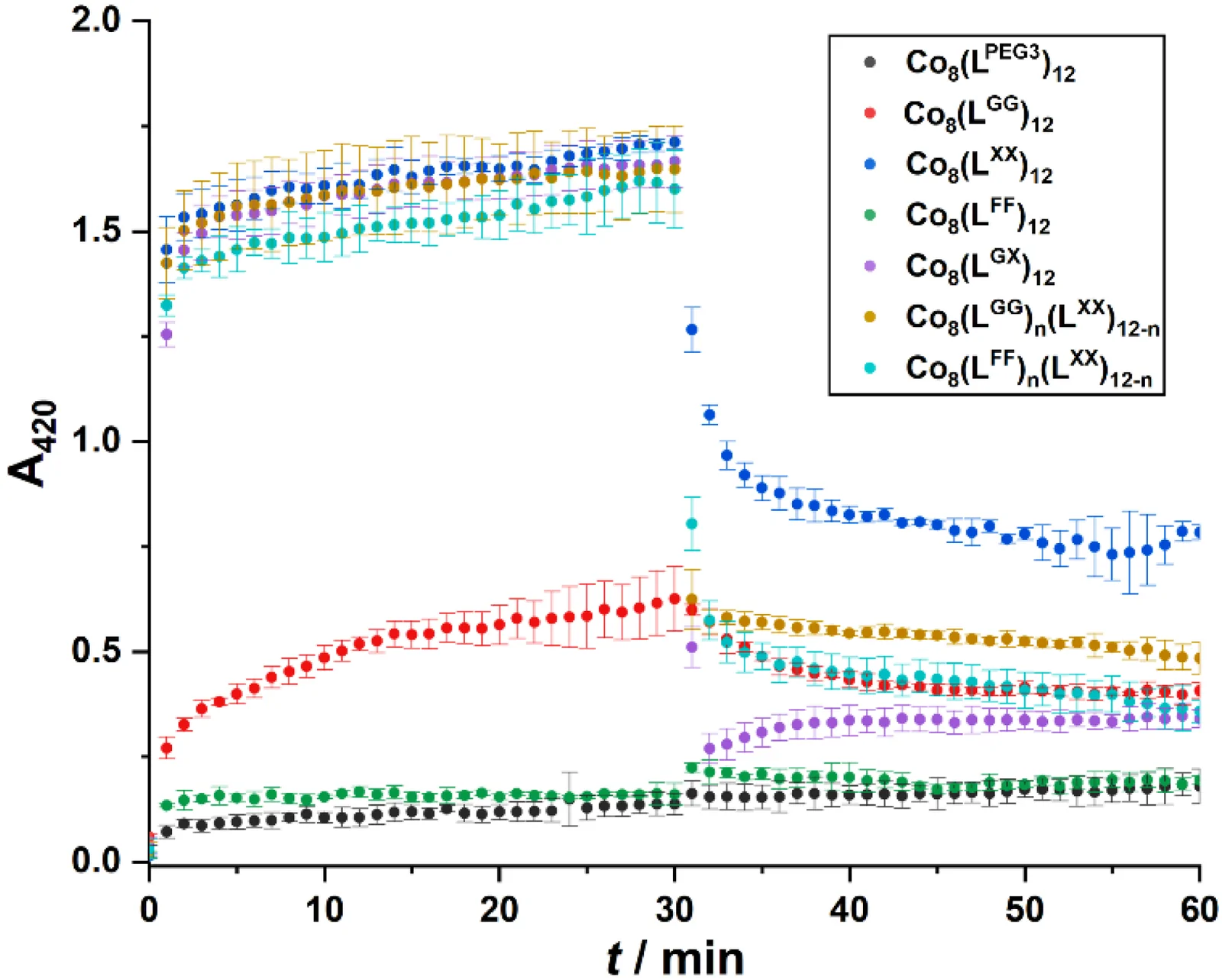
Results of turbidimetry experiments that measure absorbance changes at 420 nm for the binding of Jacalin (10 µM) to the indicated glyco-cages (500 µM). The *x*-axis denotes time after mixing Jacalin and the relevant glyco-cage. Measurements were performed in HEPES buffer (10 mM, plus 0.1 mM CaCl_2_, pH 8.5) at 298 K. In all cases an excess of free d-galactose was added after 30 minutes to disrupt the aggregations. Data points are average of three independent measurements with error bars indicating one standard deviation.

**Fig. 4:**
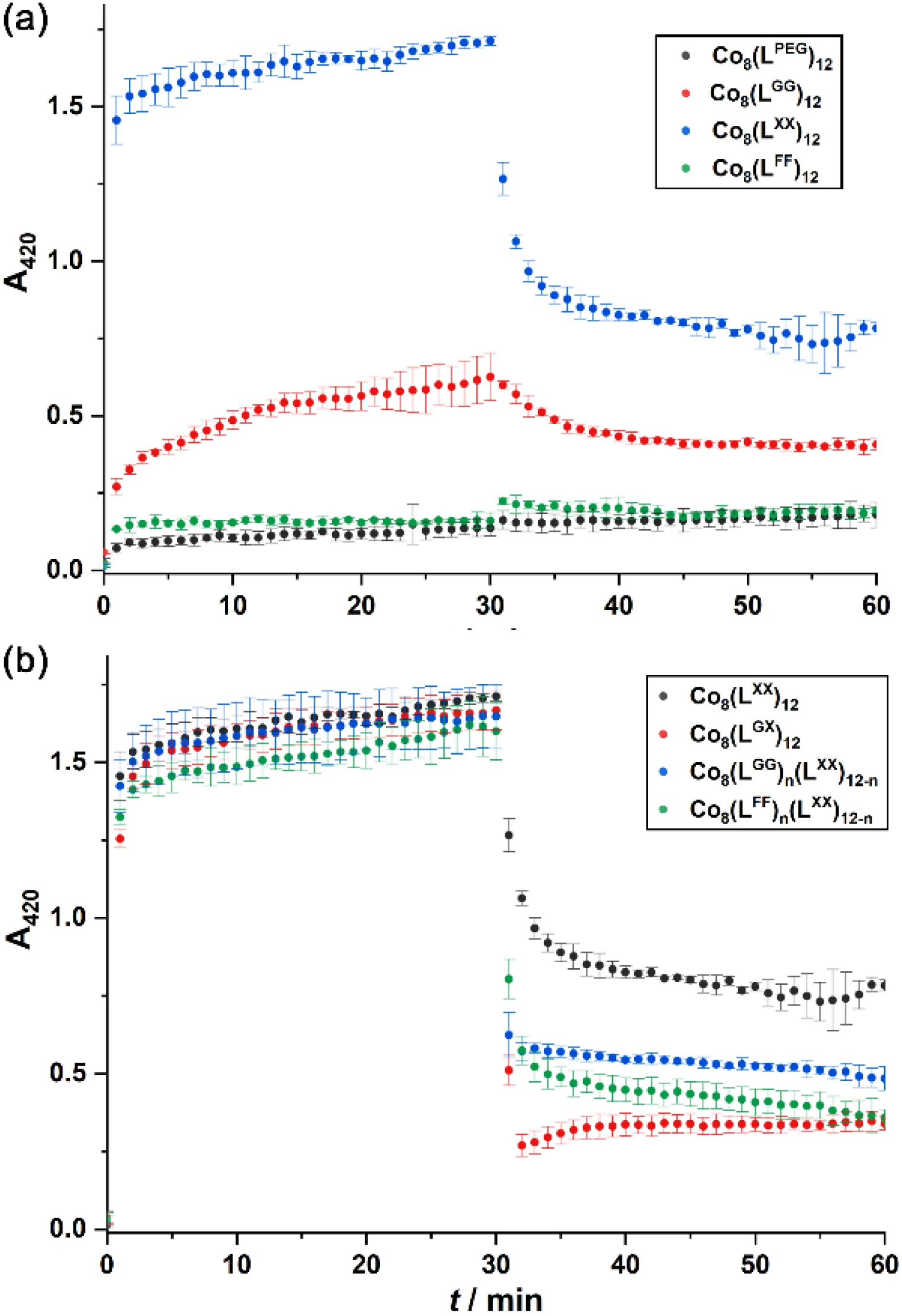
Separation of turbidimetry results from Fig. 3 into two sets for clarity: (a) data for the set of homo-glycan cages with Jacalin; (b) data for the set of mixed-glycan cages compared to the all-galactose homo-glycan cage.

Firstly, as a control experiment, we see that a PEG-ylated cage with no pendant glycans but bearing a triethylene glycol chain pendant from each pyridyl unit, Co_8_(L^PEG3^)_12_ (reported previously; see SI for structure)^22^ causes no significant aggregation with Jacalin. When glycans are added to each pyridyl unit using a Cu-AAC Click reaction^6^ to give the homo-glycan cages Co_8_(L^XX^)_12_, Co_8_(L^GG^)_12_ and Co_8_(L^FF^)_12_, each bearing 24 glycans, we see a clear turbidimetry response that reflects the differing affinities of the relevant glycans for Jacalin. Thus Co_8_(L^FF^)_12_ behaves very similarly to Co_8_(L^PEG^)_12_, inducing no significant aggregation. The glucose-pendant cage Co_8_(L^GG^)_12_ does show some aggregation, reaching an *A*_420_ value of ≈0.5 after 30 minutes, despite Jacalin being considered principally a binder of galactose:^17^ however the known ability of Jacalin to bind other monosaccharides^18^ is evident here, with the 24-glucose array surrounding M_8_(L^GG^)_12_ resulting in some crosslinking of Jacalin which can be reversed on addition of excess free galactose. However the all-galactose cage M_8_(L^XX^)_12_ results in substantially the greatest degree of aggregation in this series: the limiting *A*_420_ value with M_8_(L^XX^)_12_ is approximately 3× greater than with M_8_(L^GG^)_12_ and this limiting value is attained almost immediately. Significantly the addition of excess free galactose does not completely disrupt this aggregation but only results in a *ca.* 55% reduction in the *A*_420_ value, indicating that the M_8_(L^XX^)_12_/Jacalin interaction which results in crosslinking is sufficiently strong to withstand substantial competition from excess free galactose. The clear affinity order for Jacalin M_8_(L^XX^)_12_ > M_8_(L^GG^)_12_ > M_8_(L^FF^)_12_ is consistent with known Jacalin/glycan affinity patterns.^18^

The behaviour of the type B mixed-glycan cages Co_8_(L^XX^)_*n*_(L^GG^)_12−*n*_ and Co_8_(L^XX^)_*n*_(L^FF^)_12−*n*_ revealed significant differences from the homo-glycan cages, which is more apparent in the dis-aggregation phase than the initial aggregation phase. On combining these cages with Jacalin a rapid increase in *A*_420_ occurs in the first minute, with only a small additional increase thereafter. The magnitude of the limiting *A*_420_ values obtained with Co_8_(L^XX^)_*n*_(L^GG^)_12−*n*_ and Co_8_(L^XX^)_*n*_(L^FF^)_12−*n*_ are similar to that observed with the all-galactose cage Co_8_(L^XX^)_12_. Slightly lower limiting *A*_420_ values are evident in Fig. 3 and 4b for these mixed-glycan cages compared with the all-galactose cage, consistent with a reduction in the number of galactose residues, but these differences are at the limit of significance and to a good approximation we can say that reducing the density of galactose residues in the statistical type B ensemble, by a factor of 2 compared to the all-galactose cage, has had a negligible effect on crosslinking of these glyco-cages with Jacalin. This implies that an average number of galactose residues of 12 per cage (distributed statistically, as discussed earlier), instead of 24, is still sufficient to ensure crosslinking with Jacalin to the same extent as shown by Co_8_(L^XX^)_12_.

However the disaggregation behaviour on addition of free galactose after 30 minutes reveals the different behaviours of the homo-glycan and mixed-glycan cages more clearly, in that the aggregates of Co_8_(L^XX^)_*n*_(L^GG^)_12−*n*_ and Co_8_(L^XX^)_*n*_(L^FF^)_12−*n*_ with Jacalin are disrupted to a substantially greater extent than is the Co_8_(L^XX^)_12_/Jacalin assembly. The limiting *A*_420_ values after addition of free galactose (see the traces after *t* = 30 minutes in Fig. 3 and 4b) reveal an affinity order Co_8_(L^XX^)_12_ > Co_8_(L^XX^)_*n*_(L^GG^)_12−*n*_ > Co_8_(L^XX^)_*n*_(L^FF^)_12−*n*_, consistent with the affinity order revealed by the homo-glycan cages (X > G > F).

From these measurements with the type B mixed-glycan cages we can conclude that:

• Replacing half of the galactose residues with either glucose or fucose residues has little effect on cage/lectin crosslinking under these conditions as judged by turbidity measurements: the presence of 12 galactose units instead of 24 is still clearly sufficient for effective crosslinking. The effect of halving the number of galactose residues is much more apparent in the disaggregation phase in the presence of excess galactose as a competitor which pushes the equilibrium towards disaggregation.

• Replacing galactose residues by fucose causes a greater reduction in affinity than does replacement of galactose by the same number of glucose residues: hence, the affinity order of individual glycans with Jacalin is galactose > glucose > fucose. This is consistent with the known promiscuity of Jacalin for glycans other than galactose^18^ and is also reflected in the observations with the homo-glycan cages that binding affinities with Jacalin lie in the order Co_8_(L^XX^)_12_ > Co_8_(L^GG^)_12_ > Co_8_(L^FF^)_12_.

We were also interested to evaluate the mixed-glycan type A cages (based on the non-symmetrical bridging ligands) for their crosslinking ability with Jacalin compared to the type B cages. An important consideration here is that the type A cages are considerably more difficult and protracted to synthesise than the type B cages, given the additional synthetic steps required to prepare a non-symmetrically disubstituted bridging ligand (Scheme 2). Fig. 3 and 4b show that the behaviour of Co_8_(L^GX^)_12_ with Jacalin is significantly different from that of Co_8_(L^XX^)_*n*_(L^GG^)_12−*n*_ in the disaggregation phase. Whilst Co_8_(L^GX^)_12_ and Co_8_(L^XX^)_*n*_(L^GG^)_12−*n*_ show comparable aggregation with Jacalin during the first 30 minutes, in the presence of excess galactose as a competitor, Co_8_(L^GX^)_12_ disaggregates significantly more (final *A*_420_ ≈ 0.3) – and hence, has a lower affinity for Jacalin – than does Co_8_(L^XX^)_*n*_(L^GG^)_12−*n*_ (final *A*_420_ ≈ 0.5). The difference is clearly significant with respect to the size of the error bars for the triplicate measurements. The higher affinity of the type B ensemble for Jacalin than the type A ensemble is understandable in terms of its greater compositional heterogeneity: of the 13 different compositions present, it is quite plausible that one is particularly effective at crosslinking Jacalin in a way that does not occur with the type A complex ensemble whose isomers all have the same composition.

#### 2.4.3 Fucose-containing cages: binding to UEA-I

The results of these experiments, collected in Fig. 5, follow the same pattern as those reported above using Jacalin. As before, subsets of this data are extracted for clarity and shown in Fig. 6.

**Fig. 5:**
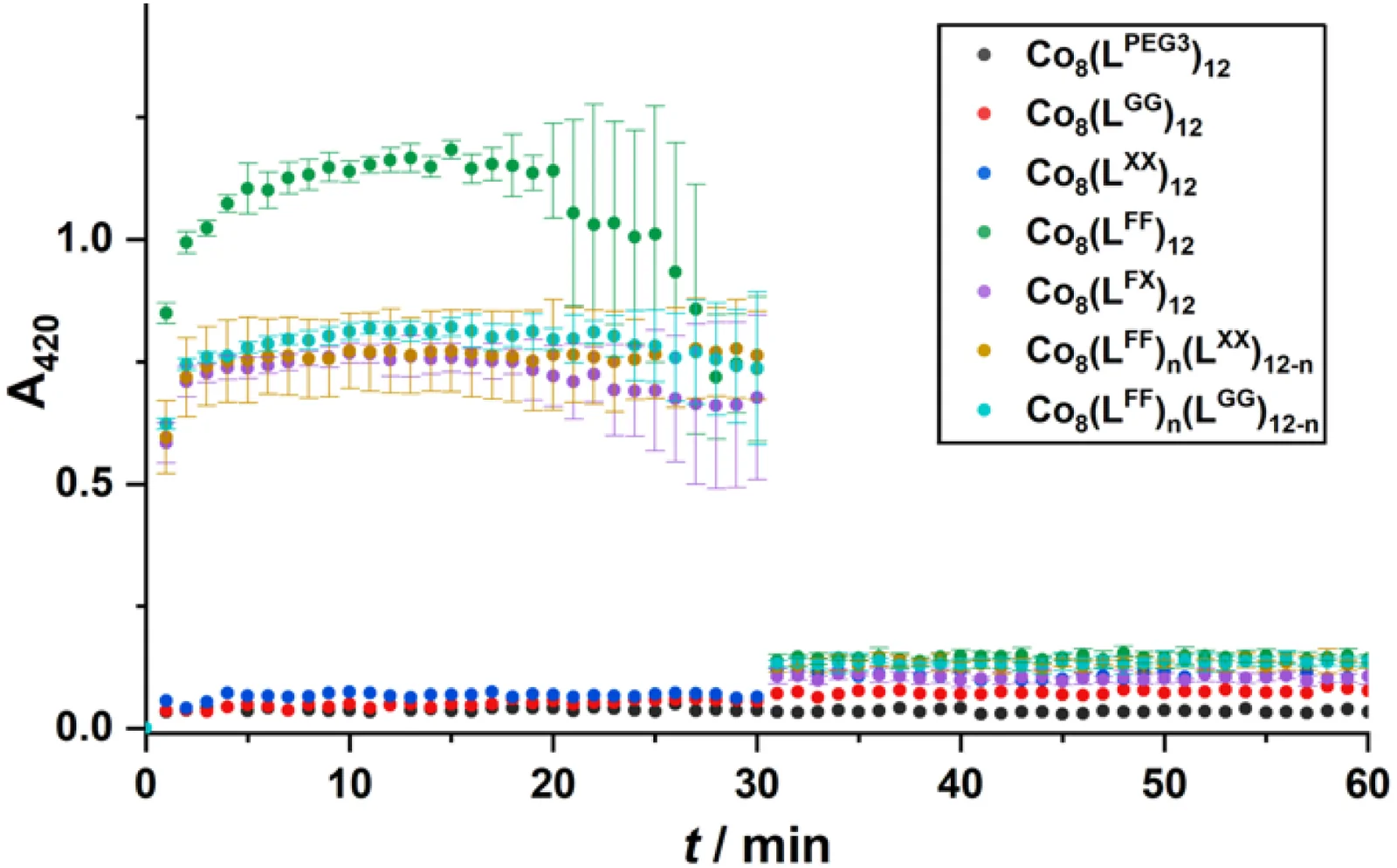
Results of turbidimetry experiments that measure absorbance changes at 420 nm for the binding of UEA-I (10 µM) to the indicated glyco-cages (500 µM). The *x*-axis denotes time after mixing Jacalin and the relevant glyco-cage. Measurements were performed in HEPES buffer (10 mM, plus 0.15 M NaCl, 2 mM CaCl_2_, 0.2 mM MnCl_2_, pH 7.4) at 298 K. In all cases an excess of free l-fucose was added after 30 minutes to disrupt the aggregations.

**Fig. 6:**
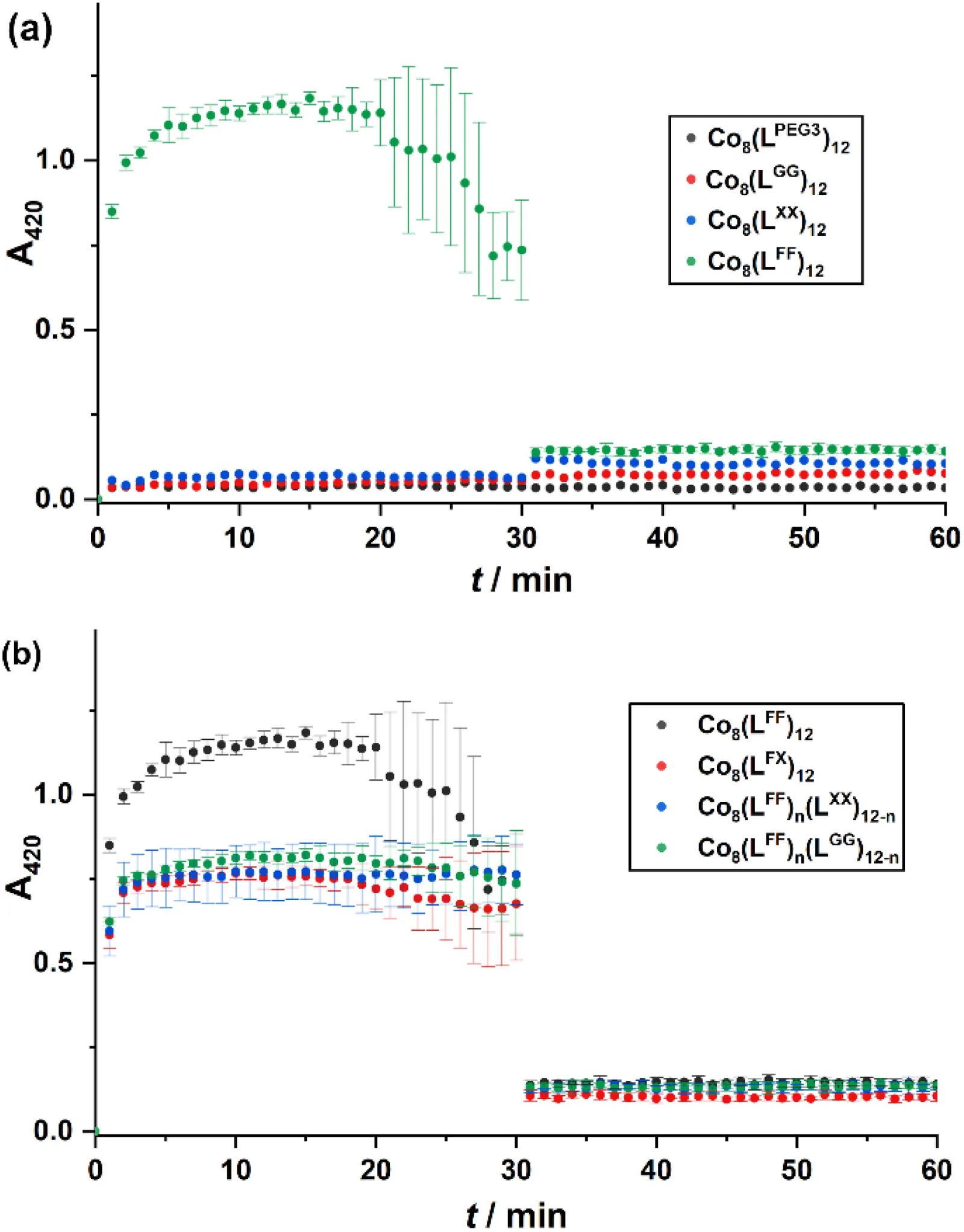
Separation of turbidimetry results from Fig. 5 into two sets for clarity: (a) data for the set of homo-glycan cages with UEA-I; (b) data for the set of mixed-glycan cages compared to the all-fucose homo-glycan cage.

The PEG-ylated cage Co_8_(L^PEG3^)_12_ (no pendant glycans) induces no aggregation with UEA-I, and neither do the homo-glycan cages Co_8_(L^XX^)_12_ and Co_8_(L^GG^)_12_. However the fucose-pendant cage Co_8_(L^FF^)_12_ shows strong aggregation: clearly UEA-I is highly specific for fucose and lacks the promiscuity of Jacalin for other glycans.^18^ Addition of excess fucose disrupts the aggregate essentially completely. We note that the *A*_420_ value following initial aggregation of UEA-I with Co_8_(L^FF^)_12_, which is quickly complete, starts to diminish after about 20 minutes which is associated with the aggregate starting to precipitate and falling out of the optical path in the turbidimetry cell (the effect could be reversed by briefly shaking the cuvette): the measurement error here is commensurately increased. Accordingly we take the *A*_420_ values obtained after 15 minutes (here, limiting *A*_420_ ≈ 1.3) as plateau values when aggregation is complete but precipitation has not started.

From the *A*_420_ values at *t* = 15 minutes we can clearly see that the three mixed-glycan cages Co_8_(L^GG^)_*n*_(L^FF^)_12−*n*_ and Co_8_(L^XX^)_*n*_(L^FF^)_12−*n*_ (type B), and Co_8_(L^FX^)_12_ (type A), all cause significantly less aggregation with UEA-I (limiting *A*_420_ values all similar, in the range 0.7–0.8) than does the homo-glycan cage Co_8_(L^FF^)_12_, which can be ascribed to the reduced number of fucose residues, *i.e.* the cluster glycoside effect. This contrasts with the Jacalin-based aggregates in which a 50% reduction in the fraction of galactose residues in the glycan arrays did not significantly diminish aggregation: this implies stronger binding in the Jacalin/galactose-cage aggregates than the UEA-I/fucose-cage aggregates under the conditions used. We note also that there is no significant difference in aggregation between the type A and type B mixed-glycan cages with UEA-I [*i.e.* Co_8_(L^FX^)_12_ and Co_8_(L^XX^)_*n*_(L^FF^)_12−*n*_ behave similarly], in contrast to what was observed with Jacalin using the galactose-containing cages.

Consistent with binding in the UEA-I/fucose-cage aggregates being weaker than in the Jacalin/galactose-cage aggregates, addition of fucose as a competitor results in essentially complete disaggregation of the UEA-I/fucose-cage aggregates in all cases (residual *A*_420_ values all <0.15, Fig. 4). The weaker binding of the UEA-I/fucose-cage aggregates means that affinity differences are more apparent in the aggregation phase of the turbidimetry experiments than the disaggregation phase in the presence of a competitor, whereas the converse is true for the Jacalin/galactose-cage aggregates.

### 2.5. Homo-glycan cages with extended spacers: effect of spacer length on glycocluster/lectin binding

In this final section we describe how lengthening the flexible spacer between the M_8_ cage core and the pendant glycans, while preserving the composition of the glycan array, has quite different effects in different glyco-cage/lectin combinations. Spacer length in a glycocluster array is an important variable in determining lectin binding affinities, with two competing factors in play. Principally, increased length (and hence conformational flexibility, assuming that a flexible spacer is used) can provide the glycocluster with more scope to adapt to the steric demands of the lectin binding sites and hence give higher binding affinities, and there are many examples of glycomaterials exhibiting this behaviour.^23^ On the other hand greater rigidity (hence, more preorganisation of the glycan array) comes with the benefit of a reduced entropy loss on binding and can benefit glycocluster/lectin binding – but only if the fit is a good one to start with.^23*i*,24^

There is only a single study of this type in the field of coordination-cage based glycoclusters, which demonstrated modest affinity increases of mannose-pendant glycocages to one lectin (concanavalin-A) as the linker length increased.^7*a*^ Accordingly we were interested to exploit the self-assembly multivalency technique used in this work to investigate effects of increased spacer length on binding affinity with members of our cage family and the two different lectins (Jacalin and UEA-I) that have featured in this paper.

Scheme 3 shows the new ligand synthesis, which follows the method used for the ligands shown in Scheme 1 whose syntheses was reported earlier^6^ – *viz.* a Click reaction between an *O*-acetyl protected glycan azide, and two alkyne groups pendant from the bis(pyrazolyl-pyridine) ligand core (L^PEG5-CC^ in Scheme 3) which supports formation of the M_8_L_12_ cage cores. The difference is the introduction of penta(oxo-ethylene) chain, instead of a single CH_2_OCH_2_ unit, between the pyridyl ring and the pendant triazole group at each ligand terminus. Full synthetic details are in the SI. These ligands were assembled around Co(ii) ions in a 2 : 3 metal : ligand ratio, as before, to form the cages which we denote Co_8_(L^PEG5-FF^)_12_, with 24 pendant fucose residues, and Co_8_(L^PEG5-XX^)_12_, with 24 pendant galactose residues: these are analogues of Co_8_(L^FF^)_12_ and Co_8_(L^XX^)_12_ but containing the longer spacers between cage and pendant glycans. Again, details of synthesis and NMR/MS characterisation are in the SI.

**Scheme 3:**
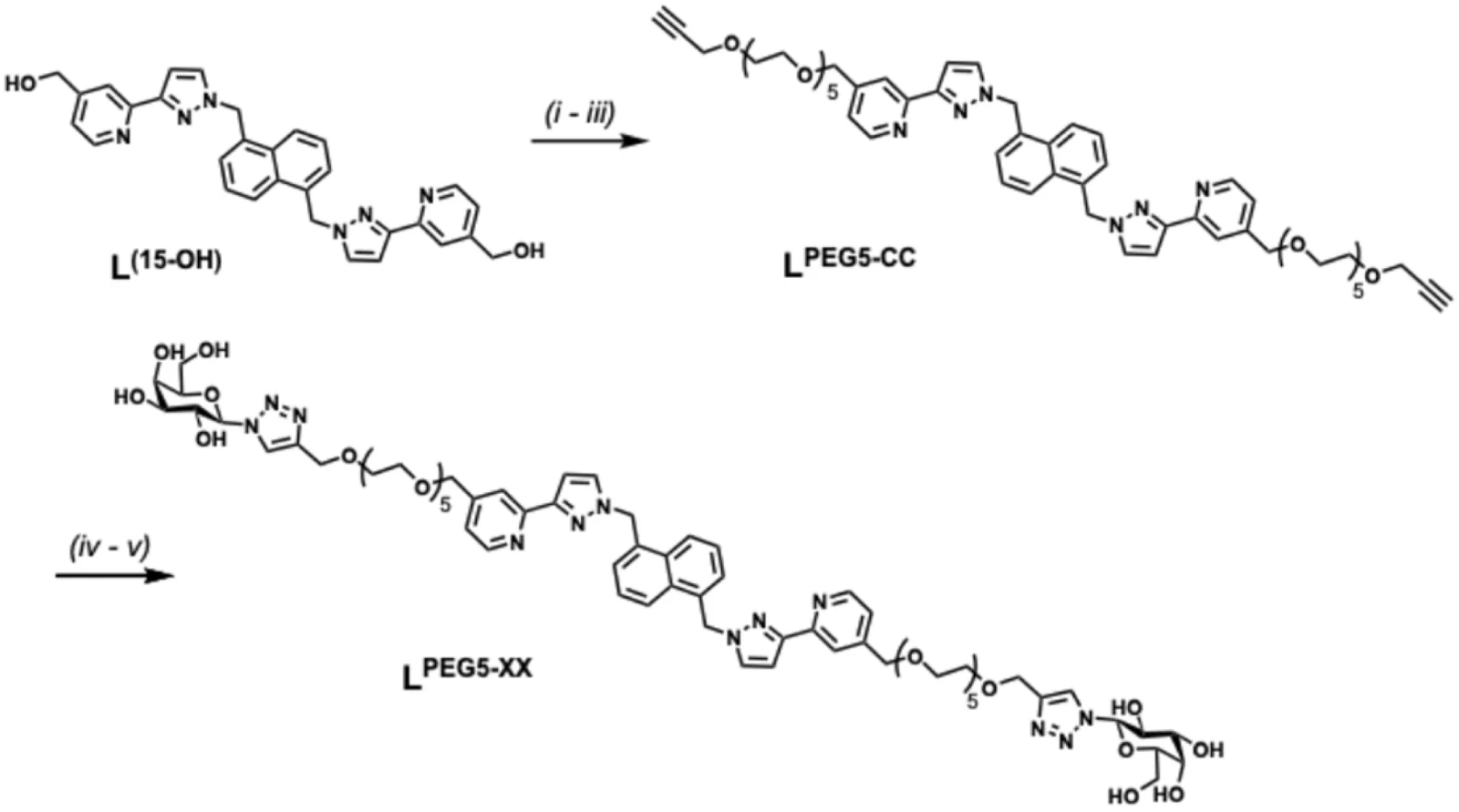
Synthesis of extended spacer ligand L^PEG5-XX^ containing penta-(oxoethylene) chains between ligand core and pendant galactose units (L^PEG5-FF^ was prepared similarly). Reagents: (i) 3,3-diisopropyl-2-methyl-4,7,10,13,16-pentaoxa-3-silaoctadecan-18-yl-4-methylbenzenesulfonate (excess); (ii) TBAF/THF; (iii) propargyl bromide (excess); (iv) 1-azido-1-deoxy-β-d-galactopyranoside tetraacetate; (v) NaOMe/MeOH.

Turbidimetry measurements comparing the ability of Co_8_(L^XX^)_12_ and Co_8_(L^PEG5-XX^)_12_ to induce aggregation with Jacalin are shown in Fig. 7. It is clear that the additional spacers present in Co_8_(L^PEG5-XX^)_12_ result in a greater degree of aggregation with Jacalin compared to Co_8_(L^XX^)_12_: the limiting *A*_420_ value, reached after *ca.* 15 minutes, is approximately doubled for Co_8_(L^PEG5-XX^)_12_ (again the reduction in *A*_420_ between *t* = 15 and 30 minutes can be ascribed to slow precipitation of the aggregate, as it disappears on manual agitation of the cuvette). The *A*_420_ plateau region is slower to be reached with Co_8_(L^PEG5-XX^)_12_ – *ca.* 10 minutes, compared to essentially instant with Co_8_(L^XX^)_12_ – which is likely an effect of the greater conformational flexibility of Co_8_(L^PEG5-XX^)_12_ whose decreased preorganisation slows down the aggregation phase. Addition of excess galactose as a competitor at *t* = 30 minutes results in a comparable decrease in *A*_420_ in both cases, though the rate of decrease is notably slower with Co_8_(L^PEG5-XX^)_12_, with the slower disaggregation phase compared to Co_8_(L^XX^)_12_ consistent with stronger Co_8_(L^PEG5-XX^)_12_/Jacalin binding. In general the clearly increased affinity of Co_8_(L^PEG5-XX^)_12_ for Jacalin arising from the increased flexibility of the linker chains follows established precedents.^7*a*,23^

**Fig. 7:**
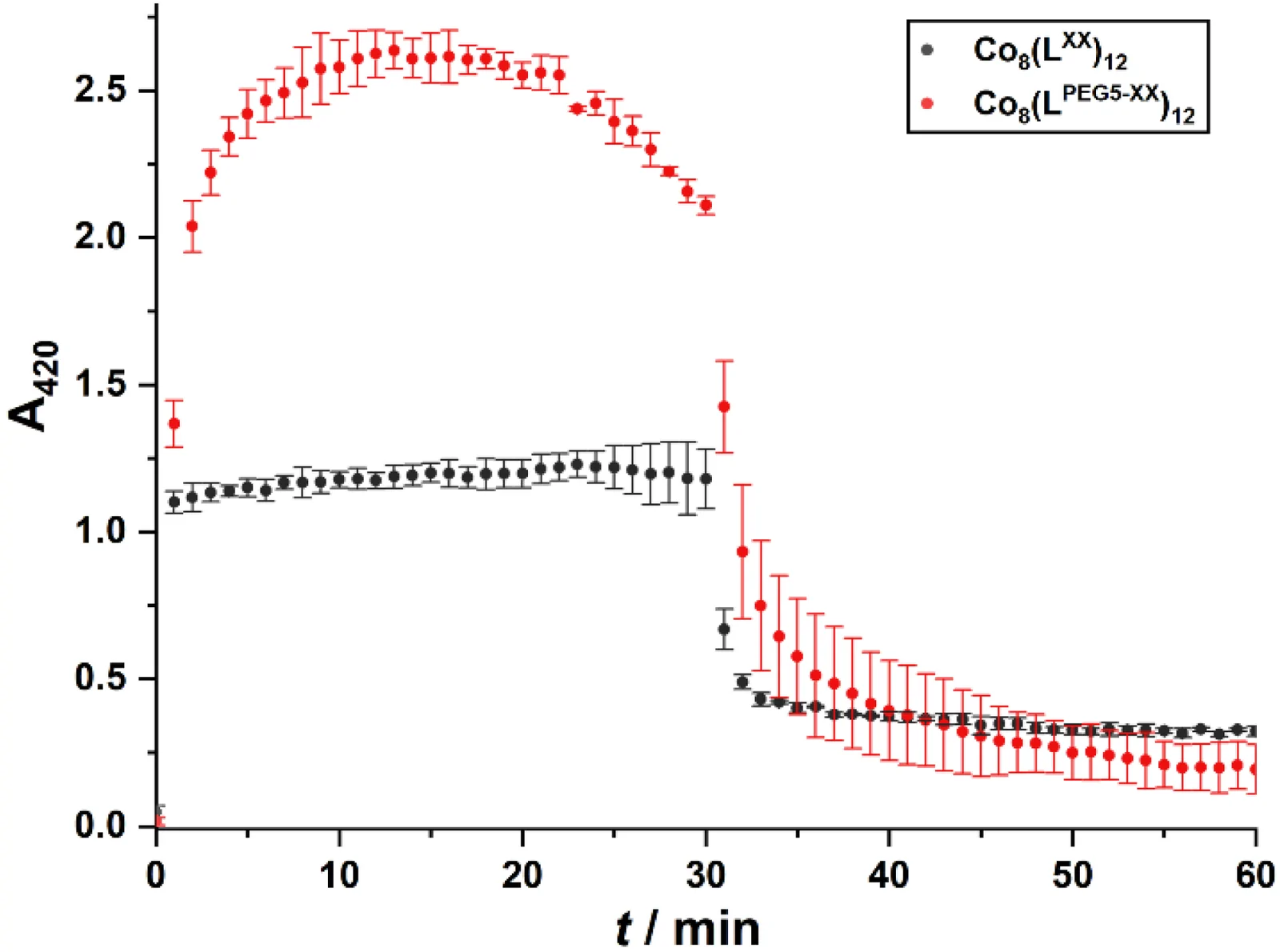
Results of turbidimetry experiments that measure absorbance changes at 420 nm for the binding of Jacalin (10 µM) to the indicated glyco-cages (50 µM). The *x*-axis denotes time after mixing Jacalin and the relevant glyco-cage. Measurements were performed in HEPES buffer (10 mM, plus 0.1 mM CaCl_2_, pH 8.5) at 298 K. A solution of an excess of d-galactose (1 M) was added at *t* = 30 minutes to disrupt the binding.

The behaviour of the equivalent fucose-substituted cage Co_8_(L^PEG5-FF^)_12_ with UEA-I is strikingly different (Fig. 8). In this case the additional flexibility of the longer spacers has reduced the affinity of the cage for UEA-I compared to Co_8_(L^FF^)_12_, to the extent that aggregation is virtually undetectable under these conditions. This suggests a geometric mismatch between the arrangement of the fucose binding sites in UEA-I and the presentation of the fucose array of Co_8_(L^PEG5-FF^)_12_ in a way which clearly does not happen with the Co_8_(L^PEG5-XX^)_12_/Jacalin combination.

**Fig. 8:**
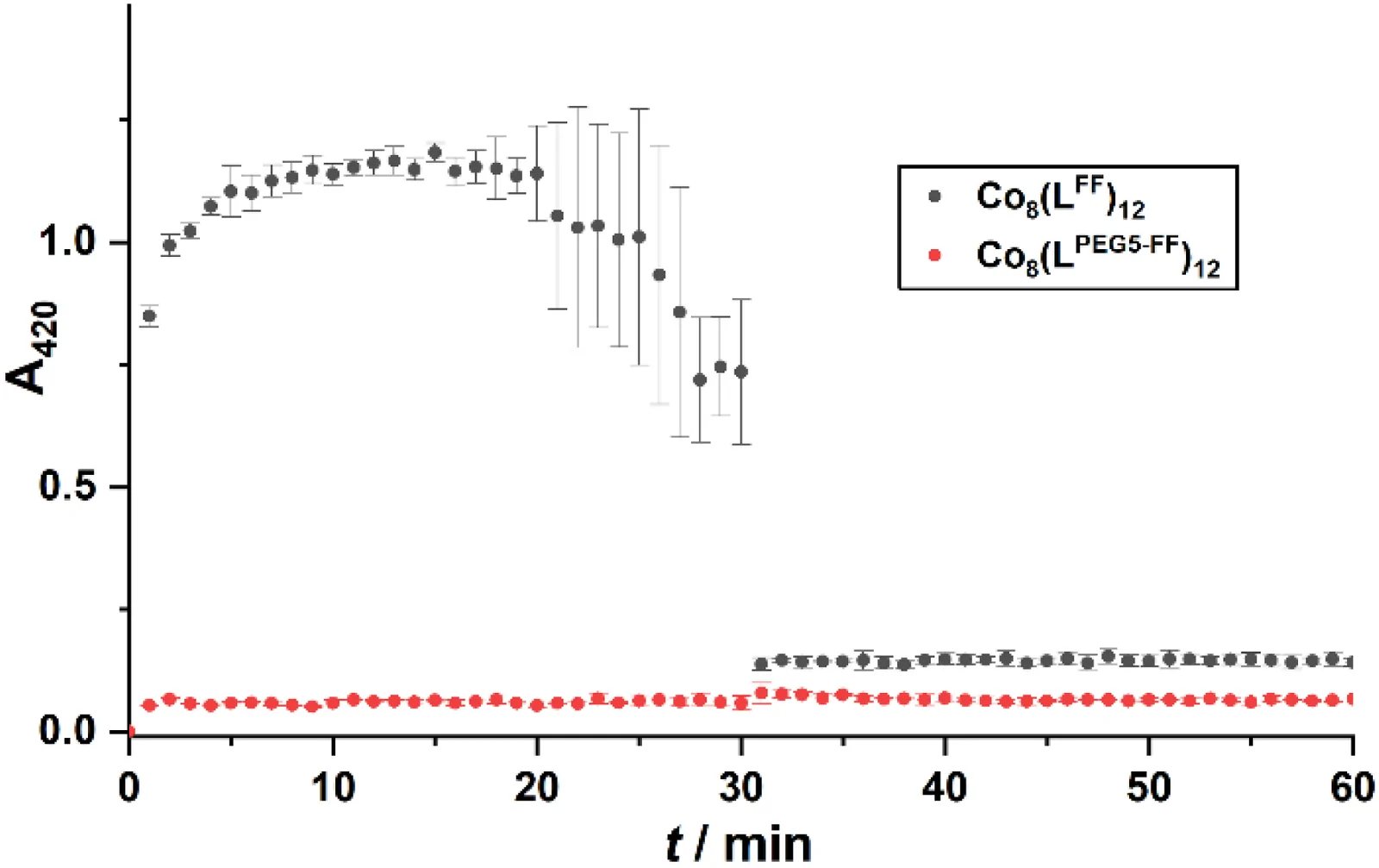
Results of turbidimetry experiments that measure absorbance changes at 420 nm for the binding of UEA-I (10 µM) to the indicated glyco-cages (500 µM). The *x*-axis denotes time after mixing UEA-I and the relevant glyco-cage. Measurements were performed in HEPES buffer (10 mM, plus 2 mM CaCl_2_, 0.2 mM MnCl_2_, 0.15 M NaCl, pH 7.4) at 298 K. A solution of an excess of l-fucose (1 M) was added at *t* = 30 minutes to disrupt the binding.

UEA-I (a dimer) contains two identical fucose binding sites which, depending on the exact conformation, are *ca.* 20 Å apart,^25^ a separation which is comparable to the size of Co_8_(L^FF^)_12_: thus Co_8_(L^FF^)_12_ may be able to span the fucose recognition sites of UEA-I and bind as a chelating ligand. However this also implies that the greater linker length in Co_8_(L^PEG5-FF^)_12_ may be unhelpful for binding to UEA-I, with the chains being sufficiently long that multivalent binding would require a substantial degree of folding and loss of conformational entropy.^23*i*,24^ In contrast tetrameric Jacalin has a much greater separation between binding sites (50–60 Å),^26^ and clearly benefits from the greater length of the spacers in Co_8_(L^PEG5-XX^)_12_ which affords a greater degree of aggregation compared to Co_8_(L^XX^)_12_.

## 3 Conclusions

Following our earlier work on using a family of coordination cages as scaffolds for multi-glycan arrays with a high degree of spatial and geometric control,^6^ we have extended this further in two ways.

Firstly, we have developed two different synthetic routes to preparation of M_8_L_12_ (approximately cubic) cages which bear 24 pendant glycans, as before – but now with the glycan shell consisting of a mixture of two different glycan types (glucose/galactose; glucose/fucose; fucose/galactose). The type A cages are based on non-symmetrical bridging ligands which bear a different glycan at each terminus, such that assembly of 12 of these ligands around a core of 8 M(ii) ions results in a combination 12 units of each of the two glycan types around the cage, with multiple isomers possible according to the orientation of each edge-bridging ligand in the assembly. The type B cages are prepared using a 1 : 1 mixture of two ligand types which each bear two identical glycans, which will result in a statistical mixture of 13 different glycan compositions in the glycan array surrounding the cage core, again with multiple isomers present for most of the compositions.

Turbidimetry measurements to investigate binding of these cages to the plant lectins Jacalin (matched with the galactose-containing cages) and UEA-I (matched with the fucose-containing cages) reveal some interesting patterns which are summarised as follows.

• The mixed-glycan cages show in all cases weaker binding to their respective lectins than the homo-glycan cages, associated with a reduction in the number of primary glycans from 24 to (on average) 12. This reduction in affinity is apparent in some cases in the aggregation phase of the turbidimetry experiments (greater limiting *A*_420_ absorbance value) and in some cases by a reduction in the extent of disaggregation (higher residual *A*_420_ absorbance value after additional of the free glycan as a competitor).

• Jacalin and UEA-I both show the highest affinity with the homo-glycan cages that match the known selectivity of those lectins, *i.e.* M_8_(L^XX^)_12_ and M_8_(L^FF^)_12_ respectively: however Jacalin also shows some affinity for M_8_(L^GG^)_12_, whereas UEA-I is more selective and shows no such secondary preference.

• The type B mixed-glycan cages containing galactose residues show higher affinity for Jacalin (less disaggregation in the presence of free galactose) than the type A cages containing the same overall glycan combination: we ascribe this to the higher compositional heterogeneity of the type B system (13 different compositions) compared to the type A cages which all contain strictly 12 glycans of each type. No such effect was observed with UEA-I, with the fucose-containing A-type and B-type cages showing similar degrees of aggregation with UEA-I.

Secondly, we have prepared new homo-glycan cages Co_8_(L^PEG5-FF^)_12_ and Co_8_(L^PEG5-XX^)_12_ which are analogues of Co_8_(L^FF^)_12_ and Co_8_(L^XX^)_12_ (24 pendant glycans) but with substantially longer linker groups [a CH_2_OCH_2_ group has been replaced by a (CH_2_OCH_2_)_5_ group at all 24 positions attaching the glycans to the cage core], with the aim of extending the very limited set of studies on how linker length affects glycocage/lectin affinity in coordination-cage based glycoclusters.^7*a*^ We find that increasing the spacer length results in a substantial increase in affinity of Co_8_(L^PEG5-XX^)_12_ for Jacalin compared to Co_8_(L^XX^)_12_, consistent with much known data indicating that longer linkers generally promote multivalent glycan binding to lectins by providing flexibility to match the lectin steric requirements. In contrast, increasing the spacer length removes any detectable affinity of Co_8_(L^PEG5-FF^)_12_ for UEA-I compared to Co_8_(L^XX^)_12_, which we suggest is because the greater spacer length between glycans and cage core results in the glycans in Co_8_(L^PEG5-FF^)_12_ being too far apart for multivalent binding to the relatively closely-spaced pair of binding sites UEA-I.

Overall this work has demonstrated the value of the simple self-assembly multivalency approach to glycocluster synthesis,^9^ using a geometrically precise 3-D coordination cage as the underlying scaffold, for (a) the synthesis of new hetero-glycan arrays of interest for analysing glycan/lectin interactions and (b) synthesis of cages with different spacer lengths to the pendant glycans whose effect is clearly highly lectin-dependent.

Finally we note that, although cross-linking (the basis of the turbidimetry experiments) is a crude/qualitative measure of binding affinity, cross-linking potential is a key desirable property of multivalent glycans, bringing receptors together to trigger signal transduction, for example. Our findings are novel, directly relevant to this, and suggest many future roles for structurally well-defined cages in glycobiology. Based on the findings we have reported here a key next step will be method development of assays that allow more quantitative analysis of binding affinities.¶

## Author contributions

CLP: Synthesis and characterisation of most complexes; DLS measurements; most turbidimetry experiments. FK: assistance with synthesis of some complexes. ML: some turbidimetry measurements. MIG and MDW: Project conception and supervision, data analysis, manuscript preparation.

## Conflicts of interest

There are no conflicts to declare.

## Supplementary Material

SC-OLF-D6SC01981A-s001

## Data Availability

The data supporting this article are included in the supplementary information (SI). Supplementary information is available: all experimental information including synthesis and characterisation by NMR spectrocopy and mass spectrometry, and methods used for DLS and turbidimetry measurements. See DOI: https://doi.org/10.1039/d6sc01981a.
