# Supplementary material for: Coordination-cage-templated multivalent glycan arrays: effects of mixed-glycan composition and chain length on lectin binding
Source: Chem Sci. 2026 Sep 28. Online ahead of print. doi: 10.1039/d6sc01981a (PMC13643526; doi:10.1039/d6sc01981a)
Supplement: Supplementary file 1 [file SC-OLF-D6SC01981A-s001.pdf]

Supplementary Information (SI) for Chemical Science.
This journal is © The Royal Society of Chemistry 2026
Supplementary Information (SI) for Chemical Science.
This journal is © The Royal Society of Chemistry

**Coordination-cage-templated multivalent glycan arrays: effects of mixed-glycan composition and chain length on lectin binding**

Callum L. Pritchard, Melissa Ligorio, Filip Kusnir, Matthew I. Gibson and Michael D. Ward*

***Supporting information: full experimental details***


| | |
|---|---|
| **1. Materials and methods** | **2** |
| **2. Selected NMR spectra of ligands** | **23** |
| **3. Selected HRMS spectra of ligands** | **43** |
| **4. Selected HRMS and NMR spectra of cubic cages** | **51** |
| **5. Tabulated $^{1}$H NMR data for $[Co_8(L^{FF})_n(L^{XX})]_{12-n}(BF_4)_{16}$** | **63** |
| **6. Dynamic Light Scattering (DLS) Experiments** | **66** |
| **7. Turbidimetry experiments** | **71** |
| **8. References** | **73** |

# 1. Materials and Methods

## Materials

All reagents and solvents used within the synthesis and purification were purchased from commercial sources (Sigma-Aldrich®, Fischer-Scientific®, Acros-Organics® or Fluorochem Ltd.) and used without prior purification unless otherwise stated. The following material was prepared using literature procedures: 1-azido-1-deoxy-β-D-glucopyranoside tetraacetate and 1-azido-1-deoxy-β-D-galactopyranoside tetraacetate;[1] $L^{(15\text{-}CC)}$;[2] $L^{(15\text{-}OH)}$;[3] 3,3-diisopropyl-2-methyl-4,7,10,13,16-pentaoxa-3-silaoctadecan-18-yl 4-methylbenzenesulfonate.[4,5] Dry MeOH was kept over pre-dried 4Å molecular sieves prior to use. L-fucose was purchased from Biosynth. Jacalin was purchased from Cambio and UEA-I and D-galactose were purchased from Merck®. The symbol nomenclature for glycans is applied to all glycan-based compounds within this ESI (where glucose = blue, galactose = yellow and fucose = red). Ligands functionalised with glycans are given a shorthand name and compound number, to cut down long IUPAC nomenclature.[6]

## Techniques

Air-sensitive reactions were performed under nitrogen or argon atmospheres using typical Schlenk techniques. $^{1}H$ NMR and $^{13}C\{^{1}H\}$ NMR spectra were recorded at 400 or 500 MHz ($^{1}H$) and 100 or 125 MHz ($^{13}C$) respectively on Bruker Avance III HD (400 MHz), Bruker Avance NEO (400 MHz) and Bruker Avance III HD (500 MHz) spectrometers. All NMR spectra were measured at 25°C in the indicated deuterated solvents unless stated otherwise. Proton and carbon chemical shifts (δ) are reported in ppm and coupling constants (*J*) are reported in Hertz (Hz). The resonance multiplicity in the $^{1}H$ NMR spectra are described as "s" (singlet), "d" (doublet), "t" (triplet), "q" (quartet), "dd" (doublet of doublets), "ddd" (doublet of doublet of doublets) and "m" (multiplet) and broad resonances are indicated by "br".

2D homonuclear correlation $^{1}H$-$^{1}H$ COSY and 2D heteronuclear correlation $^{1}H$-$^{13}C$ HETCOR experiments (HMQC, HMBC) were used to confirm NMR peak assignments. Infrared spectra were recorded on a Bruker Alpha IR-PLATINUM-ATR and Agilent Technologies Cary 630 FTIR spectrophotometers using solid samples. Low resolution mass spectrometry was performed using an Agilent 6130B ESI-MS snd accurate mass measurements (ESI-HRMS) were performed on a Bruker Compact ESI-Q-TOF. Either protonated molecular ions $[M + nH]^{n+}$ or sodium adducts $[M + nNa]^{n+}$ were used for empirical formula confirmation.

Purifications by column chromatography were performed using either silica gel (Fluorochem Ltd, 60Å, 40–63μ) or Brockmann III aluminium oxide (Sigma-Aldrich®). Size exclusion chromatography was performed on Sephadex LH-20® or Sephadex G-50®. The purities of the products were established by thin-layer chromatography (TLC) on either silica gel coated aluminium plates (with F254 indicator: layer thickness, 200 μm; particle size, 10-12 μm; pore size 60 Å) or aluminium oxide coated aluminium-backed plates (with F254 indicator: layer thickness, 200 μm; pore size 60 Å).

## Synthesis of cage ligands for use in cubic cages

**(*2S,3S,4R,5R,6S*)-6-methyltetrahydro-2H-pyran-2,3,4,5-tetrayl tetraacetate (1)**[7]

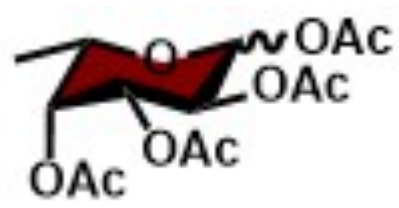


To a solution of L-fucose (1.0 g, 6.0 mmol, 1.0 equiv.) in pyridine (10 mL) was added acetic anhydride (4 mL, 42.0 mmol, 7 equiv.) and DMAP (74 mg, 0.6 mmol, 0.1 equiv.). The solution was stirred overnight under an $N_2$ atmosphere at RT. The reaction was monitored by TLC (hexane:EtOAc 1:1) to ensure the reaction was complete. The solution was then diluted with EtOAc (80 mL) and washed with 0.1M HCl (2 x 25 mL), water (20 mL), saturated $NaHCO_3$ solution (20 mL), and brine (20 mL). The organic phase was dried with $MgSO_4$, and the solvent was removed *in vacuo* to yield a pale-yellow oil. Yield: 1.96 g, 5.9 mmol, 97%.

**$^1$H-NMR** (400 MHz, $CDCl_3$, 298 K): δ 6.31 (1H, d, J = 2.6 Hz, $C^1$-**H**), 5.33-5.29 (3H, m, $C^2$-**H** + $C^3$-**H** + $C^4$-**H**), 4.25 (1H, q, J = 6.6 Hz, $C^5$-**H**), 2.16, 2.12, 1.99. 1.98 (12H, s, COC**H**$_3$), 1.13 (3H, d, J = 6.5 Hz, C**H**$_3$).

**$^{13}$C-NMR** (100 MHz, $CDCl_3$, 298 K): δ 170.5, 170.2, 169.9, 169.1 (**C**OCH$_3$); 89.9 (**C$^1$**); 70.6 (**C$^3$**); 67.8 (**C$^4$**); 67.3 (**C$^5$**); 66.5 (**C$^2$**); 20.9, 20.7, 20.6, 20.6 (CO**C**H$_3$); 15.9 (**C**H$_3$).

**ESI-MS (+)** *m/z* 355.1 $[M + Na]^+$.

**(*2S,3S,4R,5R,6S*)-2-azido-6-methyltetrahydro-2H-pyran-3,4,5-triyl triacetate (2)**

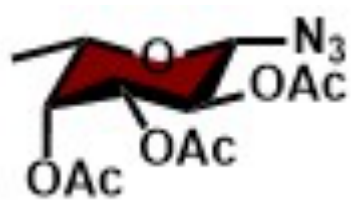


*Compound **2** was made from an adapted literature method.*[8]

To a flame-dried 2-necked round-bottomed flask was added **1** (436 mg, 1.31 mmol, 1.0 equiv.) and dry DCM (3 mL) under an $N_2$ atmosphere. To this solution was added azidotrimethylsilane (0.61 mL, 4.5 mmol, 3.5 equiv.) and $SnCl_4$ (1M in $CH_2Cl_2$, 0.34 mL, 0.34 mmol, 0.25 equiv.) and left to stir at RT. The reaction was monitored by TLC (hexane:toluene:EtOAc 3:3:4) until the disappearance of starting material. Upon completion, $CH_2Cl_2$ (15mL) was added, and the solution was washed with saturated $NaHCO_3$ solution (10 mL), water (10 mL), and brine (10 mL). The organic phase was dried with $Na_2SO_4$, and the solvent was removed *in vacuo.* The crude product was purified via flash chromatography (hexane:EtOAc 3:1) to yield a colourless oil. The product was then recrystallised with hot ethanol yielding colourless crystals. Yield: 275 mg, 0.88 mmol, 67%.

**$^1$H-NMR** (400 MHz, $CDCl_3$, 298 K): δ 5.27 (1H, dd, J = 3.3, 0.9 Hz, $C^4$-**H**); 5.17-5.12 (1H, dd, J = 10.4, 8.8 Hz, $C^2$-**H**); 5.05-5.01 (1H, dd, J = 10.3, 3.4 Hz, $C^3$-**H**); 4.58 (1H, d, J = 8.7 Hz, $C^1$-**H**); 3.90 (1H, q, J = 6.4 Hz, $C^5$-**H**); 2.19, 2.09, 1.99 (9H, s, COC**H**$_3$); 1.26 (3H, d, 6.4 Hz, C**H**$_3$).

**$^{13}$C-NMR** (100 MHz, $CDCl_3$, 298 K): δ 170.5, 170.1, 169.5 (**C**OCH$_3$), 88.2 (**C$^1$**), 71.6 (**C$^4$**), 71.2 (**C$^5$**), 69.9 (**C$^3$**), 68.2 (**C$^2$**), 20.7, 20.6, 20.6 (CO**C**H$_3$), 16.0 (**C**H$_3$).

**ESI-MS (+)** *m/z* 338.1 $[M + Na]^+$.

**General procedure A - Cu-AAC "Click" reaction**

Previously reported **L$^{(15\text{-}CC)}$** (1.0 equiv.) and the relevant acetyl-protected sugar azide derivative (2.5 equiv.) were added to a two-neck RBF under $N_2$, dissolved in $CH_2Cl_2$ (10 mL) and left to stir for 10 mins. A solution of $CuSO_4{\bullet}5H_2O$ (0.72 equiv.) and sodium ascorbate (1.0 equiv.) in $H_2O$ (10 mL) was then added to this mixture, forming a biphasic orange and red coloured system. The mixture was then heated continuously for 48 h at 60 °C.

The reaction was then cooled to RT and quenched with the addition of an $EDTA_{(aq)}$ (5 mL, 1.5 M) solution in water (10 mL) and left to stir overnight with additional $CH_2Cl_2$ (10 mL). The organic phase was separated and the aqueous phase extracted with $CH_2Cl_2$ (3 x 20 mL). Combined organic phases were dried over $Na_2SO_4$ and evaporated to give an orange/brown crude solid. The product was purified on a long Sephadex® LH-20 column using a $CH_2Cl_2$/MeOH (70:30) eluent.

**$L^{FF-Ac}$ (3)**

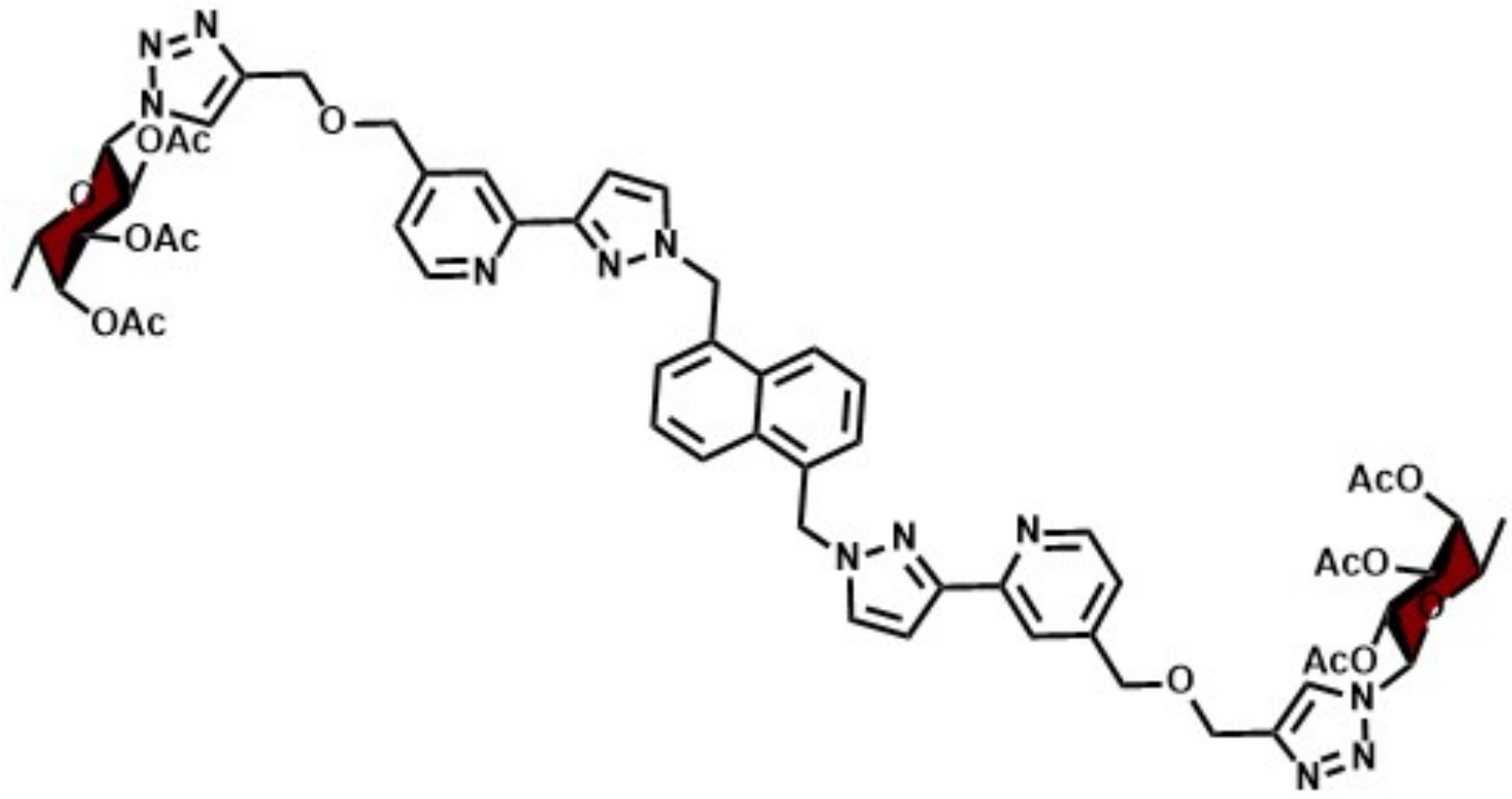

This was prepared using general procedure **A**, starting from **$L^{(15-CC)}$** (294 mg, 0.51 mmol, 1.0 equiv.) and **2** (401 mg, 1.27 mmol, 2.5 equiv.). Yield: 481 mg, 0.40 mmol, 78%.

**$^1$H-NMR** (400 MHz, $CDCl_3$, 298 K): δ 8.58 (2H, d, J = 5.0 Hz, pyridyl-**$H^6$**); 8.01 (2H, d, 8.5 Hz, naphthyl-**$H^{4/8}$**); 7.91 (2H, s, pyridyl-**$H^3$**); 7.88 (2H, s, triazole-**$H^5$**); 7.45 (2H, t, J = 7.9 Hz, naphthyl-**$H^{3/7}$**); 7.29 (2H, d, J = 7.1 Hz, naphthyl-**$H^{2/6}$**); 7.23-7.20 (4H, m, pyrazole-**$H^5$** + pyridyl-**$H^5$**); 6.84 (2H, d, J = 2.3 Hz, pyrazole-**$H^4$**); 5.84 (4H, s, napthyl-C**$H_2$**N); 5.80 (2H, d, J = 9.3 Hz, fucose $C^1$-**H**); 5.49 (2H, t, J = 10.0 Hz, fucose $C^2$-**H**); 5.36 (2H, d, J = 3.4 Hz, fucose $C^4$-**H**); 5.24-5.21 (2H, dd, J = 10.3, 3.4 Hz, fucose $C^3$-**H**); 4.73 (4H, s, $CH_2OC$**$H_2$**-triazole); 4.61 (4H, s, C**$H_2$**$OCH_2$-triazole); 4.09 (2H, q, J = 6.3 Hz, fucose $C^5$-**H**); 2.19, 1.97, 1.85 (18H, s, COC**$H_3$**); 1.22 (6H, d, J = 6.4 Hz, fucose $C^6$-**H**).

**$^{13}$C-NMR** (100 MHz, $CDCl_3$, 298 K): δ 170.3, 169.8, 169.2 (**C**$OCH_3$); 152.3 (pyridyl-**$C^2$**); 151.6 (pyrazole-**$C^3$**); 149.6 (pyridyl-**$C^6$**); 147.7 (pyridyl-**$C^4$**); 145.2 (triazole-**$C^4$**); 132.4 (naphthyl-**$C^{1/5}$** or naphthyl-**$C^{9/10}$**); 131.7 (naphthyl-**$C^{9/10}$** or naphthyl-**$C^{1/5}$**); 130.9 (pyrazole-**$C^5$**), 127.3 (naphthyl-**$C^{2/6}$**); 126.5 (naphthyl-**$C^{3/7}$**); 124.3 (naphthyl-**$C^{4/8}$**); 121.2 (triazole-**$C^5$**); 120.7 (pyridyl-**$C^5$**); 118.3 (pyridyl-**$C^3$**); 105.0 (pyrazole-**$C^4$**); 86.4 (fucose-**$C^1$**); 72.7 (fucose-**$C^5$**); 71.1 (fucose-**$C^3$**); 70.7 (**C**$H_2OCH_2$-triazole); 69.8 (fucose-**$C^4$**); 68.1 (fucose-**$C^2$**); 64.0 ($CH_2O$**C**$H_2$-triazole); 54.7 (napthyl-**C**$H_2$N); 20.7, 20.5, 20.3 (CO**C**$H_3$); 16.1 (fucose-**$C^6$**).

**High-resolution ES-MS**: *m/z* observed, 1209.4615. Calculated for $C_{60}H_{65}N_{12}O_{16}^+$ [$M$ + $H^+$], 1209.4636.

**$\nu_{max}$/cm$^{-1}$**: 2935w, 1748s, 1608m, 1560m, 1366m, 1213s, 1090s, 1043s, 921m, 788m, 772m.

**General procedure B - Zemplén deacetylation**

*O*-acetyl-protected glycan-functionalised ligand (**L$^{FF\text{-}Ac}$** or similar with a different glycan; 1.0 equiv.) was added to a 2-neck RBF under $N_2$, charged with dry MeOH (15 mL). NaOMe (1 M solution in dry MeOH) was then added until pH 9-10 was achieved and a precipitate was observed. The solution was left to stir for 18 h, neutralised with Amberlite® IR-120 (H) resin and then filtered. The solvent was evaporated and the product was used without further purification.

**L$^{FF}$ (4)**

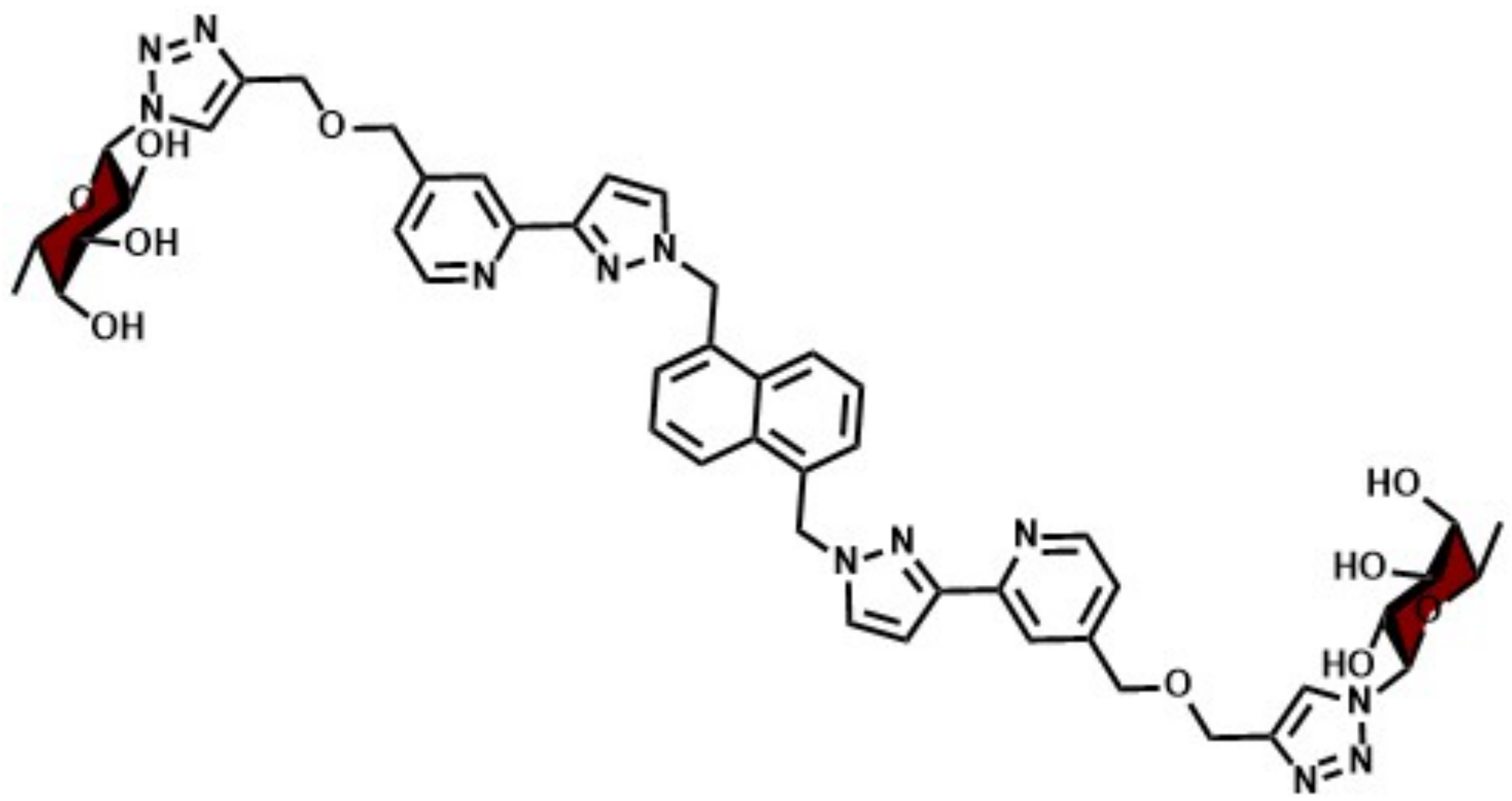


This was prepared following general procedure **B** above starting from acetyl-protected compound **3** (123 mg, 102 μmol, 1.0 equiv.). Yield: 93 mg, 97 μmol, 96%.

**$^1$H-NMR** (400 MHz, DMSO-*d*$_6$, 298 K): δ 8.52 (2H, d, J = 5.1 Hz, pyridyl-**H$^6$**); 8.30 (2H, s, triazole-**H$^5$**); 8.22 (2H, d, J = 8.4 Hz, naphthyl-**H$^{4/8}$**); 7.89 (2H, s, pyridyl-**H$^3$**); 7.86 (2H, d, J = 2.1 Hz, pyrazole-**H$^5$**); 7.57 (2H, t, J = 7.8 Hz, naphthyl-**H$^{3/7}$**); 7.29-7.25 (4H, m, naphthyl-**H$^{2/6}$** + pyridyl-**H$^5$**); 6.86 (2H, d, J = 2.1 Hz, pyrazole-**H$^4$**); 5.95 (4H, s, napthyl-C**H$_2$**N); 5.46 (2H, d, J = 9.2 Hz, fucose C$^1$-**H**); 5.10 (6H, br s, fucose-**OH**); 4.66 (4H, s, CH$_2$OC**H$_2$**-triazole); 4.64 (4H, s, C**H$_2$**OCH$_2$-triazole); 4.02 (2H, t, J = 9.2 Hz, fucose C$^2$-**H**); 3.88 (2H, q, J = 6.5 Hz, fucose C$^5$-**H**); 3.58-3.54 (4H, m, fucose C$^3$-**H** + fucose C$^4$-**H**); 1.13 (6H, d, J = 6.3 Hz, fucose C$^6$-**H**).

**$^{13}$C-NMR** (100 MHz, DMSO-*d*$_6$, 298 K): δ 151.7 (pyridyl-**C$^2$**); 151.0 (pyrazole-**C$^3$**); 149.2 (pyridyl-**C$^6$**); 148.0 (pyridyl-**C$^4$**); 143.5 (triazole-**C$^5$**); 133.7 (naphthyl-**C$^{1/5}$** or naphthyl-**C$^{9/10}$**); 130.9 (naphthyl-**C$^{9/10}$** or naphthyl-**C$^{1/5}$**); 126.3 (naphthyl-**C$^{2/6}$**); 126.2 (naphthyl-**C$^{3/7}$**); 123.8

(naphthyl-**C**$^{4/8}$); 123.0 (triazole-**C**$^{5}$); 120.5 (pyridyl-**C**$^{5}$); 117.1 (pyridyl-**C**$^{3}$); 104.4 (pyrazole-**C**$^{4}$); 88.1 (fucose-**C**$^{1}$); 73.8 (fucose-**C**$^{3}$); 73.3 (fucose-**C**$^{5}$); 71.2 (fucose-**C**$^{4}$); 69.7 (**C**H$_2$OCH$_2$-triazole); 69.0 (fucose-**C**$^{2}$); 63.3 (CH$_2$O**C**H$_2$-triazole); 53.4 (napthyl-**C**H$_2$N); 16.5 (fucose-**C**$^{6}$).

**High-resolution ES-MS**: *m/z* observed, 957.3997. Calculated for $C_{48}H_{53}N_{12}O_{10}^{+}$ [*M* + H$^{+}$], 957.4002.

**ν$_{max}$/cm$^{-1}$**: 3350s (br), 2918m, 2850m, 1596s, 1493m, 1376m, 1089s, 1050s, 770s.

**L$^{(G\text{-}Ac)(15\text{-}CC)}$ (5)**

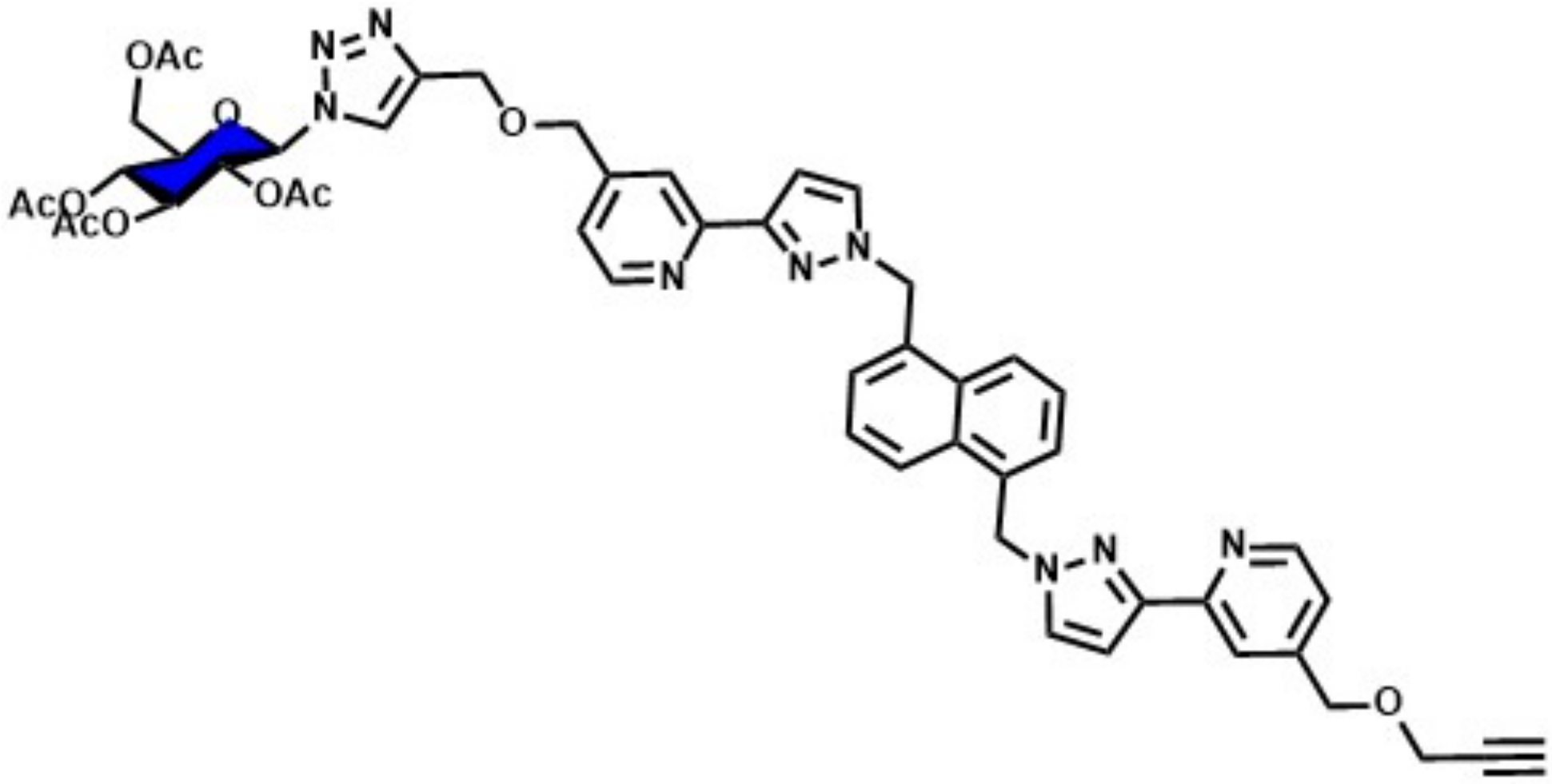


This was prepared using general procedure **A**, starting from **L$^{(15\text{-}CC)}$** (100 mg, 173 µmol, 1.0 equiv.) and 1-azido-1-deoxy-β-D-glucopyranoside tetraacetate (54 mg, 144 µmol, 0.83 equiv.). Yield: 127 mg, 67 µmol, 93%.

**$^{1}$H-NMR** (400 MHz, CDCl$_3$, 298 K): δ 8.59 (2H, d, J = 4.9 Hz, pyridyl-**H**$^{6}$); 8.02 (2H, d, J = 8.5 Hz, naphthyl-**H**$^{4/8}$); 7.93 (2H, s, pyridyl-**H**$^{3}$); 7.83 (1H, s, triazole-**H**$^{5}$); 7.46 (2H, t, J = 7.3 Hz, naphthyl-**H**$^{3/7}$); 7.30 (2H, d, J = 6.7 Hz, naphthyl-**H**$^{2/6}$); 7.24-7.19 (4H, m, pyrazole-**H**$^{5}$ + pyridyl-**H**$^{5}$); 6.85 (2H, d, J = 2.3 Hz, pyrazole-**H**$^{4}$); 5.86 (1H, d, J = 9.1 Hz, glucose C$^{1}$-**H**); 5.85 (4H, s, napthyl-C**H$_2$**N); 5.46-5.38 (2H, m, glucose C$^{2}$-**H** + glucose C$^{3}$-**H**); 5.22 (1H, t, J = 9.7 Hz, glucose C$^{4}$-**H**); 4.73 (2H, s, CH$_2$OC**H**$_2$-triazole); 4.64 (2H, s, C**H**$_2$OCH$_2$-C≡C); 4.62 (2H, s, C**H**$_2$OCH$_2$-triazole); 4.31-4.26 (1H, dd, J = 12.7, 5.1 Hz, glucose-CH$_a$**H$_b$**); 4.24 (2H, d, J = 2.3 Hz, CH$_2$OC**H**$_2$-C≡C); 4.14-4.11 (1H, dd, J = 12.5, 1.7 Hz, glucose-C**H$_a$**H$_b$); 4.00-3.96 (1H, m, glucose C$^{5}$-**H**); 2.49 (1H, t, J = 2.3 Hz, C≡C-**H**); 2.05, 2.04, 2.00, 1.85 (12H, s, COC**H**$_3$).

**$^{13}$C-NMR** (100 MHz, $CDCl_3$, 298 K): δ 170.5, 169.9, 169.4, 168.9 (**C**$OCH_3$); 152.4, 152.4 (pyridyl-**C**$^2$); 151.6 (pyrazole-**C**$^3$); 149.7, 149.6 (pyridyl-**C**$^6$); 147.5, 147.3 (pyridyl-**C**$^4$); 145.5 (triazole-**C**$^4$); 132.4 (naphthyl-**C**$^{1/5}$ or naphthyl-**C**$^{9/10}$); 131.7 (naphthyl-**C**$^{9/10}$ or naphthyl-**C**$^{1/5}$); 130.9, 130.9 (pyrazole-**C**$^5$), 127.4, 127.3 (naphthyl-**C**$^{2/6}$); 126.5 (naphthyl-**C**$^{3/7}$); 124.3 (naphthyl-**C**$^{4/8}$); 121.1 (triazole-**C**$^5$); 120.8, 120.7 (pyridyl-**C**$^5$); 118.4, 118.3 (pyridyl-**C**$^3$); 105.1 (pyrazole-**C**$^4$); 85.8 (glucose-**C**$^1$); 75.2 (C≡**C**-H); 75.2 (glucose-**C**$^5$); 72.6 (glucose-**C**$^3$); 70.7 (**C**$H_2OCH_2$-triazole); 70.4 (glucose-**C**$^2$); 70.0 (**C**$H_2OCH_2$-C≡C); 67.7 (glucose-**C**$^4$); 63.9 ($CH_2O$**C**$H_2$-triazole); 61.5 (glucose-**C**$^6$); 57.9 ($CH_2O$**C**$H_2$-C≡C); 54.7 (napthyl-**C**$H_2$N); 20.7, 20.5, 20.5, 20.2 (CO**C**$H_3$).

**High-resolution ES-MS**: *m/z* observed, 952.3622. Calculated for $C_{50}H_{50}N_9O_{11}^+$ [$M$ + $H^+$], 952.3624.

**$\nu_{max}$/cm$^{-1}$**: 3287w, 2958w, 2854w, 2110w, 1756s, 1610m, 1465m, 1368m, 1226s, 1096s, 1042s, 790m, 773m.

**L$^{(F\text{-}Ac)(15\text{-}CC)}$ (6)**

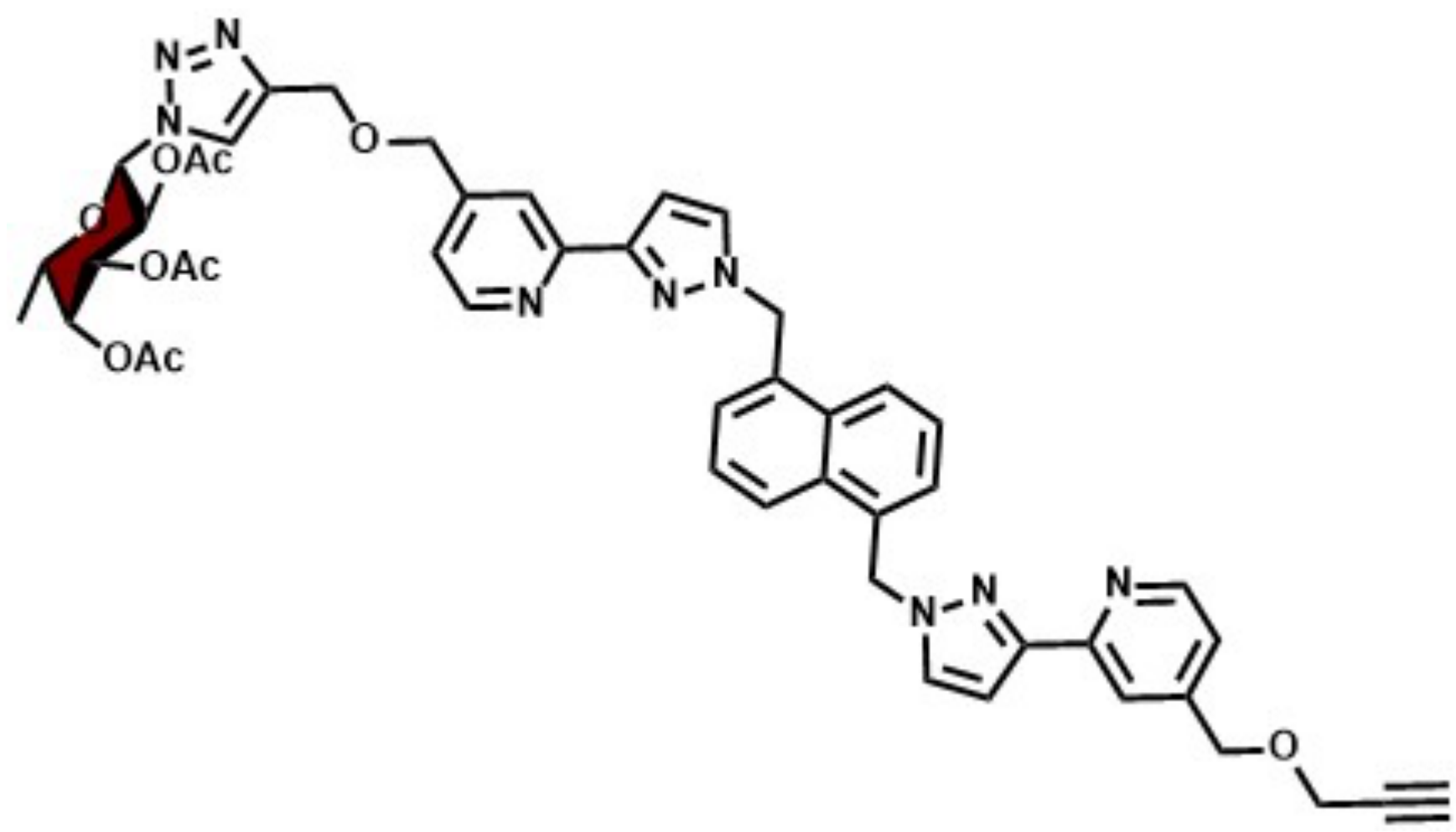


This was prepared from **L$^{(15\text{-}CC)}$** (300 mg, 0.52 mmol, 1.0 equiv.) and **2** (136 mg, 0.43 mmol, 0.83 equiv.) using general procedure **A**. Yield: 137 mg, 0.15 mmol, 36%.

**$^1$H-NMR** (400 MHz, $CDCl_3$, 298 K: δ 8.56 (2H, d, J = 4.7 Hz, pyridyl-**H**$^6$); 7.99 (2H, d, J = 8.4 Hz, naphthyl-**H**$^{4/8}$); 7.91 (2H, s, pyridyl-**H**$^3$); 7.87 (1H, s, triazole-**H**$^5$); 7.43 (2H, t, J = 7.6 Hz, naphthyl-**H**$^{3/7}$); 7.27 (2H, d, J = 6.7 Hz, naphthyl-**H**$^{2/6}$); 7.23-7.17 (4H, m, pyrazole-**H**$^5$ + pyridyl-**H**$^5$); 6.85 (2H, s, pyrazole-**H**$^4$); 5.81 (4H, s, napthyl-C**H**$_2$N); 5.80 (1H, d, J = 9.2 Hz, fucose C$^1$-**H**); 5.48 (1H, t, J = 9.7 Hz, fucose C$^2$-**H**); 5.34 (1H, d, J = 2.9 Hz, fucose C$^4$-**H**); 5.23-5.19 (1H, dd, J = 10.3, 3.1 Hz, fucose C$^3$-**H**); 4.71 (2H, s, $CH_2OC$**H**$_2$-triazole); 4.62 (2H,

s, C**H**$_2$OCH$_2$-C≡C); 4.59 (2H, s, C**H**$_2$OCH$_2$-triazole); 4.21 (2H, d, J = 2.3 Hz, CH$_2$OC**H**$_2$-C≡C); 4.07 (1H, q, J = 6.3 Hz, fucose C$^5$-**H**); 2.47 (1H, t, J = 2.1 Hz, C≡C-**H**); 2.18, 1.97, 1.84 (9H, s, COC**H**$_3$); 1.20 (3H, d, J = 6.2 Hz, fucose C$^6$-**H**).

**$^{13}$C-NMR** (100 MHz, CDCl$_3$, 298 K): δ 170.3, 169.8, 169.2 (**C**OCH$_3$); 152.3, 152.2 (pyridyl-**C**$^2$); 151.5 (pyrazole-**C**$^3$); 149.5 (pyridyl-**C**$^6$); 147.8, 147.4 (pyridyl-**C**$^4$); 145.2 (triazole-**C**$^4$); 132.4, 132.4 (naphthyl-**C**$^{1/5}$ or naphthyl-**C**$^{9/10}$); 131.7 (naphthyl-**C**$^{9/10}$ or naphthyl-**C**$^{1/5}$); 130.9, 130.9 (pyrazole-**C**$^5$), 127.3, 127.3 (naphthyl-**C**$^{2/6}$); 126.5 (naphthyl-**C**$^{3/7}$); 124.3 (naphthyl-**C**$^{4/8}$); 121.2 (triazole-**C**$^5$); 120.8 (pyridyl-**C**$^5$); 118.4, 118.3 (pyridyl-**C**$^3$); 105.1 (pyrazole-**C**$^4$); 86.3 (fucose-**C**$^1$); 75.3 (C≡**C**-H); 72.7 (fucose-**C**$^5$); 71.1 (fucose-**C**$^3$); 70.6 (**C**H$_2$OCH$_2$-triazole); 69.9 (**C**H$_2$OCH$_2$-C≡C); 69.8 (fucose-**C**$^4$); 68.1 (fucose-**C**$^2$); 64.0 (CH$_2$O**C**H$_2$-triazole); 57.9 (CH$_2$O**C**H$_2$-C≡C); 54.7 (napthyl-**C**H$_2$N); 20.7, 20.5, 20.3 (CO**C**H$_3$); 16.1 (fucose-**C**$^6$).

**High-resolution ES-MS**: *m/z* observed, 916.3385. Calculated for $C_{48}H_{47}N_9NaO_9^+$ [*M* + Na$^+$], 916.3389.

**$\nu_{max}$/cm$^{-1}$**: 3288w, 2937w, 2855w, 2109w, 1746s, 1608m, 1560m, 1360m, 1239s, 1213s, 1090s, 1043s, 924m, 788m, 772m.

**L$^{GX-Ac}$ (7)**

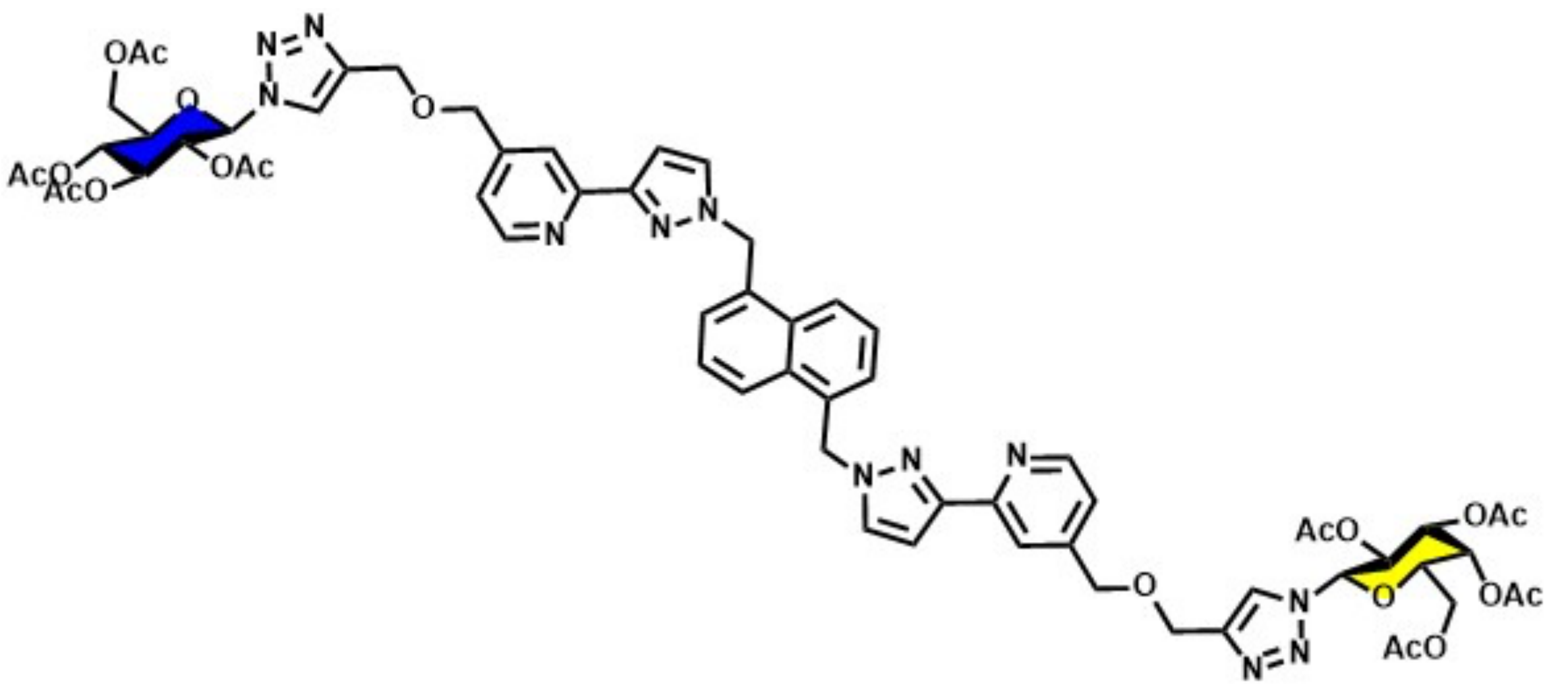


This was prepared from compound **5** (112 mg, 0.12 mmol, 1.0 equiv.) and 1-azido-1-deoxy-β-D-galactopyranoside tetraacetate (88 mg, 0.24 mmol, 2.0 equiv.) using general procedure **A**. Yield: 135 mg, 0.10 mmol, 87%.

**$^1$H-NMR** (400 MHz, $CDCl_3$, 298 K): δ 8.59 (2H, d, J = 4.5 Hz, pyridyl-$\mathbf{H^6}$); 8.02 (2H, d, J = 8.5 Hz, naphthyl-$\mathbf{H^{4/8}}$); 7.92 (2H, s, pyridyl-$\mathbf{H^3}$); 7.88 (1H, s, triazole-$\mathbf{H^5}$); 7.84 (1H, s, triazole-$\mathbf{H^5}$); 7.46 (2H, t, J = 7.3 Hz, naphthyl-$\mathbf{H^{3/7}}$); 7.30 (2H, d, J = 7.3 Hz, naphthyl-$\mathbf{H^{2/6}}$); 7.23-7.20 (4H, m, pyrazole-$\mathbf{H^5}$ + pyridyl-$\mathbf{H^5}$); 6.84 (2H, d, J = 1.6 Hz, pyrazole-$\mathbf{H^4}$); 5.88-5.83 (6H, m, napthyl-C$\mathbf{H_2}$N + galactose $C^1$-**H** + glucose $C^1$-**H**); 5.55-5.50 (2H, m, galactose $C^2$-**H** + galactose $C^4$-**H**); 5.45-5.38 (2H, m, glucose $C^2$-**H** + glucose $C^3$-**H**); 5.25-5.20 (2H, m, galactose $C^3$-**H** + glucose $C^4$-**H**); 4.73, 4.72 (4H, s, $CH_2OC\mathbf{H_2}$-triazole); 4.62 (4H, d, J = 3.1 Hz, $C\mathbf{H_2}OCH_2$-triazole); 4.28 (1H, dd, J = 12.7, 4.9 Hz, glucose-$CH_a\mathbf{H_b}$); 4.23-4.19 (1H, m, galactose $C^5$-**H**); 4.18-4.09 (3H, m, galactose-$C\mathbf{H_a}H_b$ + galactose-$CH_a\mathbf{H_b}$ + glucose-$C\mathbf{H_a}H_b$); 3.99 (1H, m, glucose $C^5$-**H**); 2.19, 2.04, 2.04, 2.00, 2.00, 1.98, 1.86, 1.85 (24H, s, $COC\mathbf{H_3}$).

**$^{13}$C-NMR** (100 MHz, $CDCl_3$, 298 K): δ 170.6, 170.4, 170.1, 170.0, 170.0, 169.5, 169.2, 169.1 (**C**$OCH_3$); 152.5 (pyridyl-$\mathbf{C^2}$); 151.7 (pyrazole-$\mathbf{C^3}$); 149.8 (pyridyl-$\mathbf{C^6}$); 147.7, 147.6 (pyridyl-$\mathbf{C^4}$); 145.6, 145.5 (triazole-$\mathbf{C^4}$); 132.5 (naphthyl-$\mathbf{C^{1/5}}$ or naphthyl-$\mathbf{C^{9/10}}$); 131.8 (naphthyl-$\mathbf{C^{9/10}}$ or naphthyl-$\mathbf{C^{1/5}}$); 131.0 (pyrazole-$\mathbf{C^5}$), 127.5 (naphthyl-$\mathbf{C^{2/6}}$); 126.6 (naphthyl-$\mathbf{C^{3/7}}$); 124.5 (naphthyl-$\mathbf{C^{4/8}}$); 121.4, 121.3 (triazole-$\mathbf{C^5}$); 120.9 (pyridyl-$\mathbf{C^5}$); 118.5 (pyridyl-$\mathbf{C^3}$); 105.2 (pyrazole-$\mathbf{C^4}$); 86.4 (galactose-$\mathbf{C^1}$); 85.9 (glucose-$\mathbf{C^1}$); 75.3 (glucose-$\mathbf{C^5}$); 74.2 (galactose-$\mathbf{C^5}$); 72.7 (glucose-$\mathbf{C^3}$); 70.9 ($\mathbf{C}H_2OCH_2$-triazole + galactose-$\mathbf{C^3}$); 70.5 (glucose-$\mathbf{C^2}$); 68.1 (galactose-$\mathbf{C^2}$); 67.8 (glucose-$\mathbf{C^4}$); 67.0 (galactose-$\mathbf{C^4}$); 64.1 ($CH_2O\mathbf{C}H_2$-triazole); 61.7 (glucose-$\mathbf{C^6}$); 61.3 (galactose-$\mathbf{C^6}$); 54.8 (napthyl-$\mathbf{C}H_2N$); 20.8, 20.7, 20.6, 20.4, 20.3 ($CO\mathbf{C}H_3$).

**High-resolution ES-MS**: *m/z* observed, 1347.4564. Calculated for $C_{64}H_{68}N_{12}O_{20}Na^+$ [$M$ + $Na^+$], 1347.4565.

**$\nu_{max}$/cm$^{-1}$**: 2924w, 2853w, 1750s, 1638m, 1617m, 1370m, 1223s, 1094m, 1046m.

**$L^{FX\text{-}Ac}$ (8)**

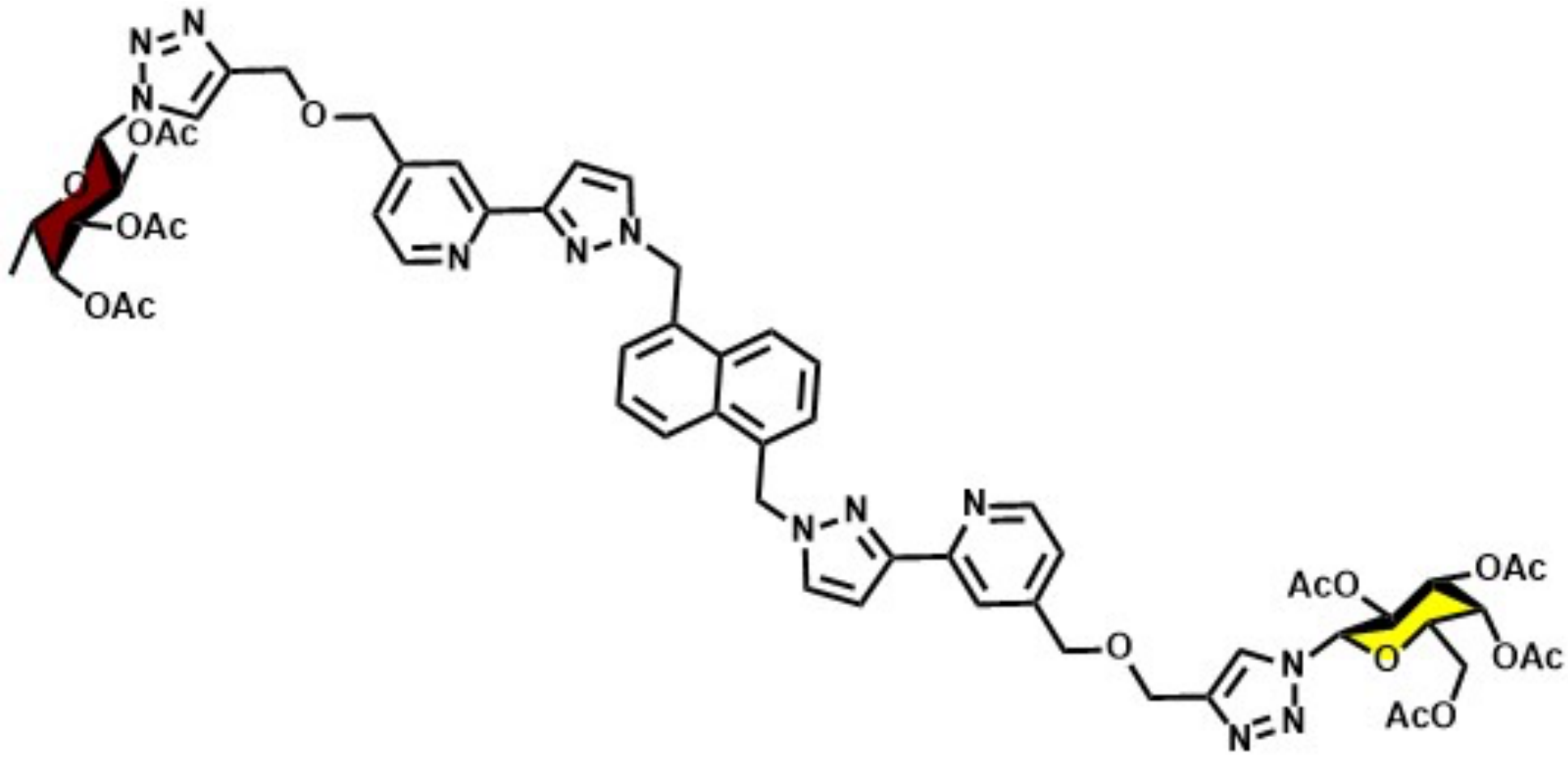


This was prepared from compound **6** (137 mg, 0.15 mmol, 1.0 equiv.) and 1-azido-1-deoxy-β-D-galactopyranoside tetraacetate (115 mg, 0.31 mmol, 2.0 equiv.) using general procedure **A**. Yield: 168 mg, 0.13 mmol, 85%.

**$^1$H-NMR** (400 MHz, $CDCl_3$, 298 K): δ 8.56 (2H, d, J = 4.5 Hz, pyridyl-**H$^6$**); 7.99 (2H, d, J = 8.5 Hz, naphthyl-**H$^{4/8}$**); 7.90 (2H, s, pyridyl-**H$^3$**); 7.87 (2H, s, triazole-**H$^5$**); 7.43 (2H, t, J = 7.3 Hz, naphthyl-**H$^{3/7}$**); 7.27 (2H, d, J = 7.3 Hz, naphthyl-**H$^{2/6}$**); 7.21-7.19 (4H, m, pyrazole-**H$^5$** + pyridyl-**H$^5$**); 6.84 (2H, d, J = 1.6 Hz, pyrazole-**H$^4$**); 5.85-5.78 (6H, m, napthyl-C**$H_2$**N + galactose C$^1$-**H** + fucose C$^1$-**H**); 5.53-5.45 (3H, m, galactose C$^2$-**H** + galactose C$^4$-**H** + fucose C$^2$-**H**); 5.34 (2H, d, J = 3.1 Hz, fucose C$^4$-**H**); 5.25-5.19 (2H, m, galactose C$^3$-**H** + fucose C$^3$-**H**); 4.71 (4H, s, $CH_2OC$**$H_2$**-triazole); 4.59 (4H, d, J = 3.1 Hz, C**$H_2$**$OCH_2$-triazole); 4.23-4.06 (4H, m, fucose C$^5$-**H** + galactose-C**$H_a$**$H_b$ + galactose-$CH_a$**$H_b$** + galactose C$^5$-**H**); 2.17, 2.15, 1.98, 1.96, 1.83 (21H, s, COC**$H_3$**); 1.20 (3H, d, J = 6.3 Hz, fucose C$^6$-**H**).

**$^{13}$C-NMR** (100 MHz, $CDCl_3$, 298 K): δ 170.3, 170.3, 169.9, 169.8, 169.8, 169.2, 169.1 (**C**$OCH_3$); 152.2, 152.2 (pyridyl-**C$^2$**); 151.4 (pyrazole-**C$^3$**); 149.5, 149.4 (pyridyl-**C$^6$**); 147.8, 147.7 (pyridyl-**C$^4$**); 145.3, 145.2 (triazole-**C$^4$**); 132.3 (naphthyl-**C$^{1/5}$** or naphthyl-**C$^{9/10}$**); 131.7 (naphthyl-**C$^{9/10}$** or naphthyl-**C$^{1/5}$**); 130.9 (pyrazole-**C$^5$**), 127.3 (naphthyl-**C$^{2/6}$**); 126.5 (naphthyl-**C$^{3/7}$**); 124.3 (naphthyl-**C$^{4/8}$**); 121.3, 121.2 (triazole-**C$^5$**); 120.7 (pyridyl-**C$^5$**); 118.3 (pyridyl-**C$^3$**); 105.1 (pyrazole-**C$^4$**); 86.3 (fucose-**C$^1$**); 86.2 (galactose-**C$^1$**); 74.0 (galactose-**C$^5$**); 72.7 (fucose-**C$^5$**); 71.1 (fucose-**C$^3$**); 70.7 (**C**$H_2OCH_2$-triazole + galactose-**C$^3$**); 69.8 (fucose-**C$^4$**); 68.1 (fucose-**C$^2$**); 68.0 (galactose-**C$^2$**); 66.9 (galactose-**C$^4$**); 63.9, 63.9 ($CH_2O$**C**$H_2$-triazole); 61.2 (galactose-**C$^6$**); 54.7 (napthyl-**C**$H_2$N); 20.7, 20.6, 20.6, 20.5, 20.5, 20.3, 20.3 (CO**C**$H_3$); 16.0 (fucose-**C$^6$**).

**High-resolution ES-MS**: *m/z* observed, 1267.4690. Calculated for $C_{62}H_{67}N_{12}O_{18}^{+}$ [$M$ + $H^{+}$], 1267.4691.

**$\nu_{max}$/cm$^{-1}$**: 2933w, 2868w, 1744s, 1608m, 1368m, 1211s, 1090s, 1044s, 923m, 790m, 772m.

**$L^{GX}$ (9)**

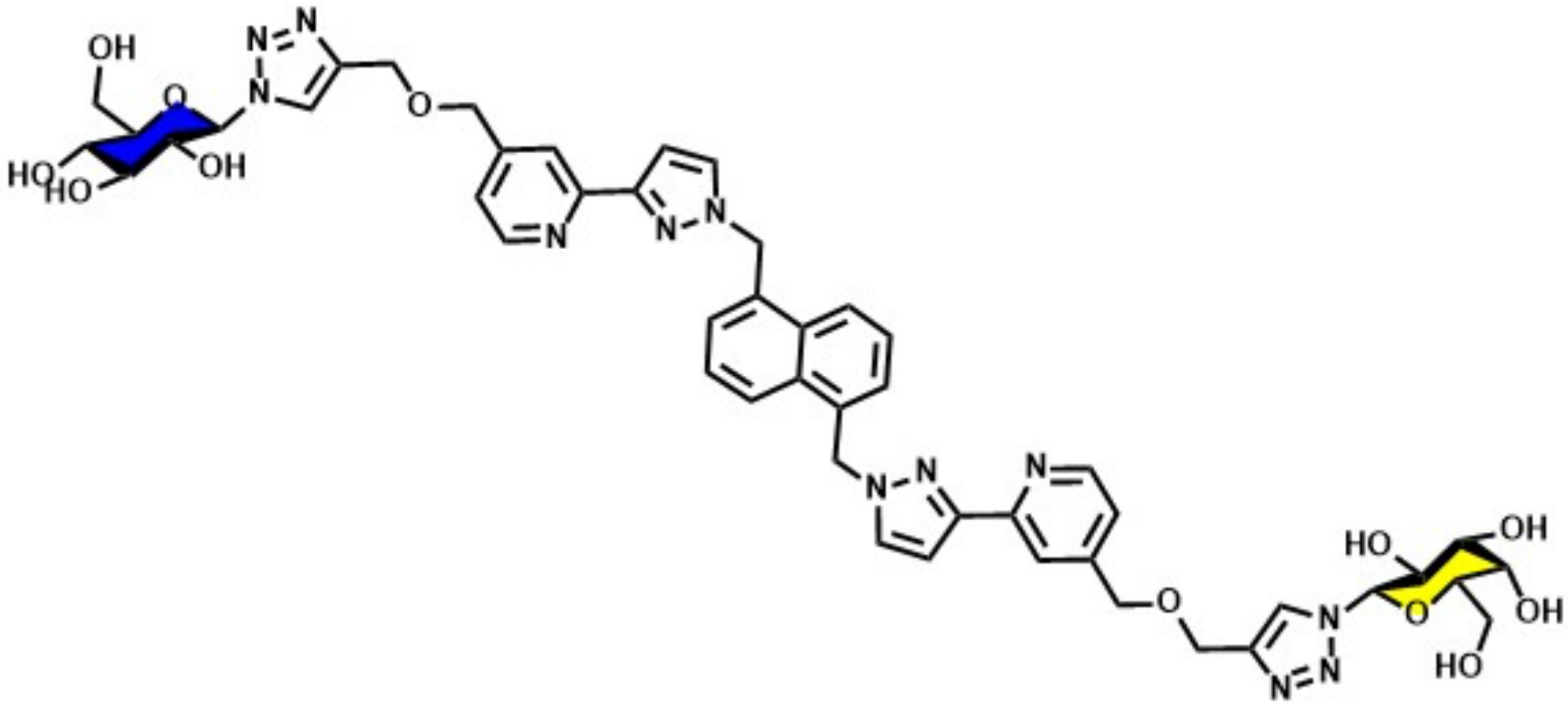

This was prepared from compound **7** (90 mg, 68 µmol, 1.0 equiv.) using general procedure **B**. The product was then washed with EtOAc (3 x 10 mL) under centrifugation and the solvent was removed. Yield: 66 mg, 67 µmol, 98%.

**$^{1}$H-NMR** (400 MHz, DMSO-$d_6$, 298 K): δ 8.52 (2H, d, J = 4.0 Hz, pyridyl-**H$^{6}$**); 8.38 (1H, s, triazole-**H$^{5}$**); 8.31 (1H, s, triazole-**H$^{5}$**); 8.21 (2H, d, J = 8.0 Hz, naphthyl-**H$^{4/8}$**); 7.88 (2H, s, pyridyl-**H$^{3}$**); 7.85 (2H, s, pyrazole-**H$^{5}$**); 7.57 (2H, t, J = 7.2 Hz, naphthyl-**H$^{3/7}$**); 7.29-7.25 (4H, m, naphthyl-**H$^{2/6}$** + pyridyl-**H$^{5}$**); 6.85 (2H, s, pyrazole-**H$^{4}$**); 5.94 (4H, s, napthyl-C**H$_2$**N); 5.53 (1H, d, J = 9.2 Hz, glucose C$^{1}$-**H**); 5.48 (1H, d, J = 9.0 Hz, galactose C$^{1}$-**H**); 4.64 (8H, s, $CH_2OC$**H$_2$**-triazole + C**H$_2$**$OCH_2$-triazole); 4.06 (1H, t, J = 8.7 Hz, galactose C$^{2}$-**H**); 3.80-3.75 (2H, m, glucose C$^{2}$-**H** + galactose C$^{5}$-**H**); 3.73-3.65 (2H, m, glucose C$^{6}$-**H** + galactose C$^{4}$-**H**); 3.58-3.39 (6H, m, galactose C$^{3}$-**H** + galactose-C**H$_a$**H$_b$ + galactose-CH$_a$**H$_b$** + glucose C$^{3}$-**H** + glucose C$^{4}$-**H** + glucose-C**H$_a$**H$_b$ + glucose-CH$_a$**H$_b$**); 3.27-3.22 (1H, m, glucose C$^{5}$-**H**).

**$^{13}$C-NMR** (100 MHz, DMSO-$d_6$, 298 K): δ 151.8 (pyridyl-**C$^{2}$**); 151.0 (pyrazole-**C$^{3}$**); 149.3 (pyridyl-**C$^{6}$**); 148.0 (pyridyl-**C$^{4}$**); 143.5, 143.5 (triazole-**C$^{4}$**); 133.7 (naphthyl-**C$^{1/5}$** or naphthyl-**C$^{9/10}$**); 132.3 (pyrazole-**C$^{5}$**); 130.9 (naphthyl-**C$^{9/10}$** or naphthyl-**C$^{1/5}$**); 126.4 (naphthyl-**C$^{2/6}$**); 126.2 (naphthyl-**C$^{3/7}$**); 123.8 (naphthyl-**C$^{4/8}$**); 123.4, 123.1 (triazole-**C$^{5}$**); 120.6 (pyridyl-**C$^{5}$**); 117.1 (pyridyl-**C$^{3}$**); 104.5 (pyrazole-**C$^{4}$**); 88.2 (galactose-**C$^{1}$**); 87.6 (glucose-**C$^{1}$**); 80.0 (glucose-

**C**$^4$); 78.5 (galactose-**C**$^4$); 76.9 (glucose-**C**$^3$); 73.7 (galactose-**C**$^3$); 72.1 (glucose-**C**$^2$); 69.7 (**C**$H_2OCH_2$-triazole); 69.6 (glucose-**C**$^5$); 69.4 (galactose-**C**$^2$); 68.4 (galactose-**C**$^5$); 63.2 ($CH_2O$**C**$H_2$-triazole); 60.8 (glucose-**C**$^6$); 60.4 (galactose-**C**$^6$); 53.4 (napthyl-**C**$H_2N$).

**High-resolution ES-MS**: *m/z* observed, 1011.3727. Calculated for $C_{48}H_{52}N_{12}O_{12}Na^+$ [$M$ + $Na^+$], 1011.3720.

**$\nu_{max}$/cm$^{-1}$**: 3284s (br), 2922w, 2851w, 1610m, 1562m, 1364m, 1338m, 1243m, 1092s, 1047s, 1016s.

**$L^{FX}$ (10)**

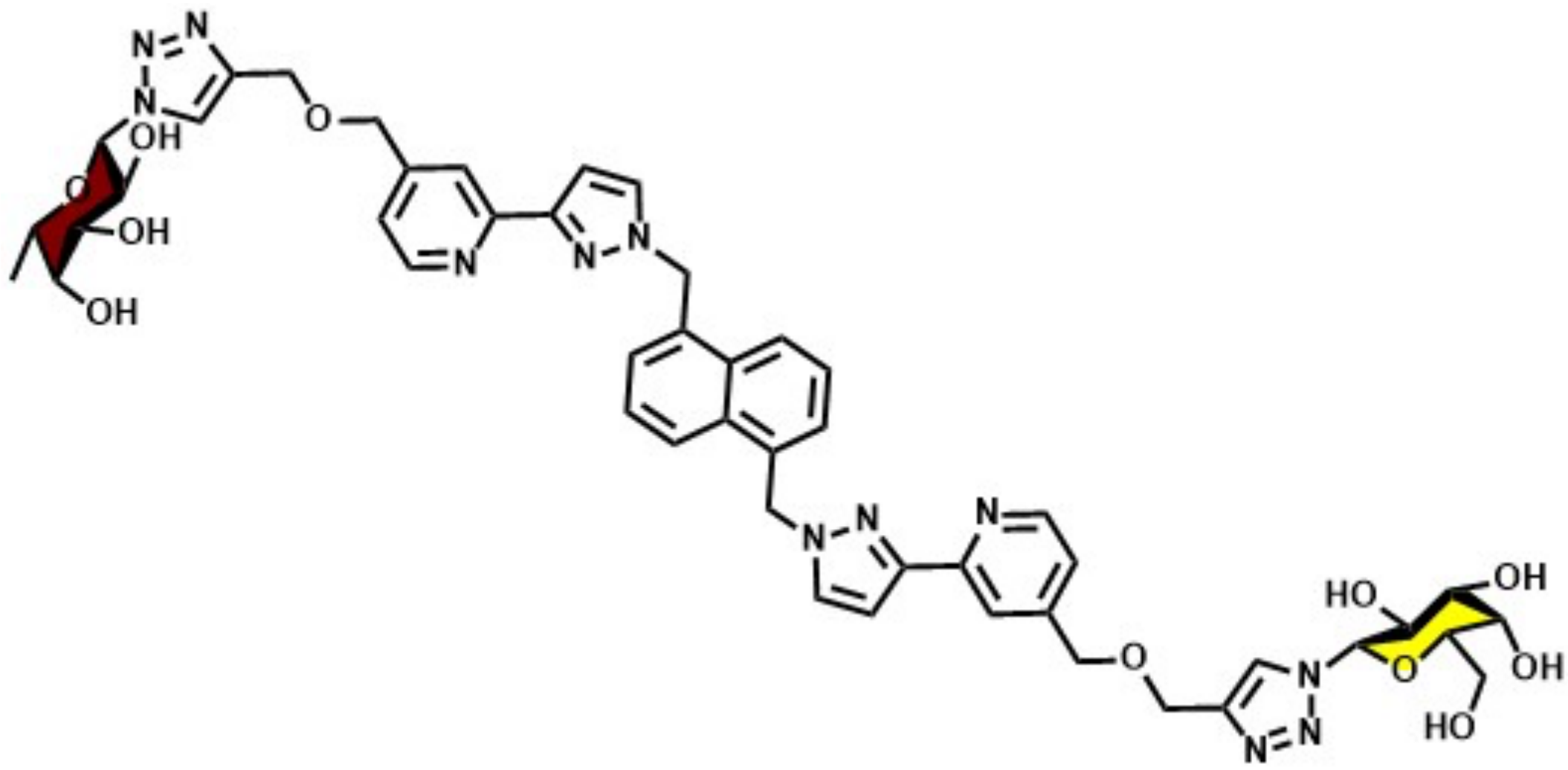


This was prepared from compound **8** (100 mg, 79 µmol, 1.0 equiv.) using general procedure **B**. Yield: 75 mg, 77 µmol, 98%.

**$^1$H-NMR** (400 MHz, DMSO-$d_6$, 298 K): δ 8.52 (2H, d, J = 4.5 Hz, pyridyl-**H**$^6$); 8.33 (1H, s triazole-**H**$^5$); 8.30 (1H, s, triazole-**H**$^5$); 8.22 (2H, d, J = 8.3 Hz, naphthyl-**H**$^{4/8}$); 7.89 (2H, s, pyridyl-**H**$^3$); 7.86 (2H, s, pyrazole-**H**$^5$); 7.57 (2H, t, J = 7.2 Hz, naphthyl-**H**$^{3/7}$); 7.29-7.25 (4H, m, naphthyl-**H**$^{2/6}$ + pyridyl-**H**$^5$); 6.86 (2H, d, J = 2.1 Hz, pyrazole-**H**$^4$); 6.08 (brs, galactose-O**H** + fucose-O**H**); 5.94 (4H, s, napthyl-C**H$_2$**N); 5.48 (2H, d, J = 10.1 Hz, galactose C$^1$-**H** + fucose C$^1$-**H**); 4.66 (4H, s, $CH_2OC$**H**$_2$-triazole); 4.64 (4H, s, C**H**$_2OCH_2$-triazole); 4.09-4.00 (2H, m, galactose C$^2$-**H** + fucose C$^2$-**H**); 3.88 (1H, q, J = 6.4 Hz, fucose C$^5$-**H**); 3.79 (1H, s, galactose C$^5$-**H**); 3.72 (1H, t, J = 5.8 Hz, galactose C$^4$-**H**); 3.58-3.47 (5H, m, galactose C$^3$-**H** + galactose-C**H$_a$**$H_b$ + galactose-$CH_a$**H$_b$** + fucose C$^3$-**H** + fucose C$^4$-**H**); 1.13 (3H, d, J = 6.3 Hz, fucose C$^6$-**H**).

**$^{13}$C-NMR** (100 MHz, DMSO-$d_6$, 298 K): δ 152.3 (pyridyl-**C$^2$**); 151.5 (pyrazole-**C$^3$**); 149.7 (pyridyl-**C$^6$**); 148.5 (pyridyl-**C$^4$**); 144.0, 144.0 (triazole-**C$^4$**); 134.2, 134.2 (naphthyl-**C$^{1/5}$** or naphthyl-**C$^{9/10}$**); 132.8 (pyrazole-**C$^5$**); 131.4 (naphthyl-**C$^{9/10}$** or naphthyl-**C$^{1/5}$**); 126.8 (naphthyl-**C$^{2/6}$**); 126.7 (naphthyl-**C$^{3/7}$**); 124.3 (naphthyl-**C$^{4/8}$**); 123.6, 123.5 (triazole-**C$^5$**); 121.0 (pyridyl-**C$^5$**); 117.6 (pyridyl-**C$^3$**); 105.0 (pyrazole-**C$^4$**); 88.6, 88.6 (galactose-**C$^1$** + fucose-**C$^1$**); 78.9 (galactose-**C$^4$**); 74.3 (fucose-**C$^3$**); 74.1 (galactose-**C$^3$**); 73.7 (fucose-**C$^5$**); 71.6 (fucose-**C$^4$**); 70.2 (**C**$H_2OCH_2$-triazole); 69.8 (galactose-**C$^2$**); 69.5 (fucose-**C$^2$**); 68.9 (galactose-**C$^5$**); 63.7 ($CH_2O$**C**$H_2$-triazole); 60.9 (galactose-**C$^6$**); 53.8 (napthyl-**C**$H_2N$); 16.9 (fucose-**C$^6$**).

**High-resolution ES-MS**: *m/z* observed, 487.2002. Calculated for $C_{48}H_{54}N_{12}O_{11}^{2+}$ [$M$ + 2H$^+$], 487.2012.

**$\nu_{max}$/cm$^{-1}$**: 3299s (br), 3140s (br), 2891w, 1709m, 1610m, 1361m, 1331m, 1226m, 1191m, 1088s, 1051s, 1003s, 880m, 790s, 766s.

**L$^{PEG5\text{-}OTIPS}$ (11)**

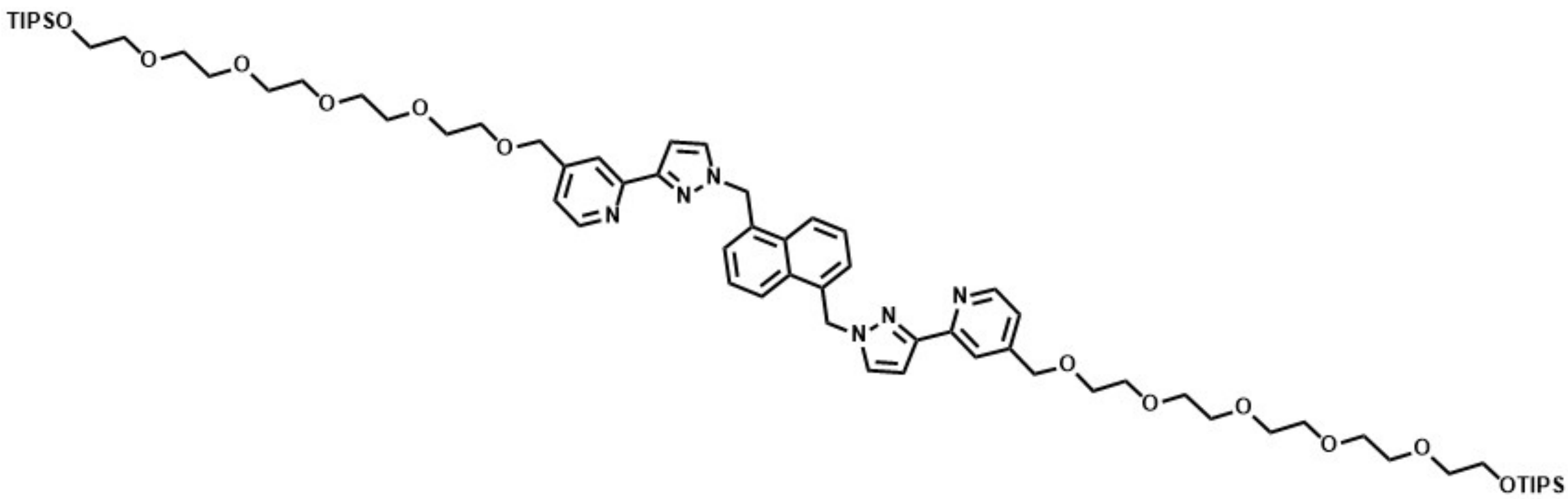


NaH (557 mg, 14.0 mmol, 60 wt. % dispersion in mineral oil, 5.0 equiv.) and 15-crown-5 ether (1.10 mL, 5.6 mmol, 2.0 equiv.) were added to a flame dried two neck RBF charged with dry THF (50 mL): the mixture was stirred for 30 mins under $N_2$. To this suspension, a solution of **L$^{(15\text{-}OH)}$** (1.40 g, 2.8 mmol, 1.0 equiv.) in dry THF (100 mL) was transferred via cannula and the resulting suspension was refluxed for 2 h before the addition of 3,3-diisopropyl-2-methyl-4,7,10,13,16-pentaoxa-3-silaoctadecan-18-yl 4-methylbenzenesulfonate (5.38 g, 9.8 mmol, 3.5 equiv.). The mixture was left to reflux further for 48 h. Once cooled to RT, the reaction was quenched with the addition of MeOH (50 mL) and the solvents evaporated to give a brown crude oil. The mineral oil was removed by filtration through a celite®/$CH_2Cl_2$ plug. The crude product was then purified through an $Al_2O_3$ column (Brockmann activity III) using $CH_2Cl_2$/MeOH (99:1) as eluent to remove unreacted starting material and a silica column using

$CH_2Cl_2$/MeOH (96:4) as eluent to remove crown ether impurities. Yield: 2.40 g, 1.93 mmol, 69% (yellow oil).

**$^1$H-NMR** (400 MHz, $CDCl_3$, 298 K) $\delta$ppm: 8.58 (2H, d, J = 5.1 Hz, pyridyl-**$H^6$**); 8.03 (2H, d, J = 8.4 Hz, naphthyl-**$H^{4/8}$**); 7.90 (2H, s, pyridyl-**$H^3$**); 7.47 (2H, t, J = 7.6 Hz, naphthyl-**$H^{3/7}$**); 7.31 (2H, d, J = 6.9 Hz, naphthyl-**$H^{2/6}$**); 7.24-7.21 (4H, m, pyrazole-**$H^5$** + pyridyl-**$H^5$**); 6.84 (2H, d, J = 2.1 Hz, pyrazole-**$H^4$**); 5.85 (4H, s, napthyl-C**$H_2$**N); 4.62 (4H, s, C**$H_2$**$OCH_2$-PEG); 3.81 (4H, t, J = 5.6 Hz, C**$H_2$**-OTIPS); 3.71-3.59 (32H, m, **H**$_{aliphaticPEG}$); 3.57-3.54 (4H, t, J = 5.4 Hz, TIPSO-$CH_2$-C**$H_2$**); 1.12-0.99 (42H, m, O**TIPS**).

**$^{13}$C-NMR** (100 MHz, $CDCl_3$, 298 K) $\delta$ppm: 152.3 (pyridyl-**$C^2$**); 151.8 (pyrazole-**$C^3$**); 149.7 (pyridyl-**$C^6$**); 148.3 (pyridyl-**$C^4$**); 132.4 (naphthyl-**$C^{1/5}$** or naphthyl-**$C^{9/10}$**); 131.8 (naphthyl-**$C^{9/10}$** or naphthyl-**$C^{1/5}$**); 130.9 (pyrazole-**$C^5$**), 127.4 (naphthyl-**$C^{2/6}$**); 126.6 (naphthyl-**$C^{3/7}$**); 124.4 (naphthyl-**$C^{4/8}$**); 120.7 (pyridyl-**$C^5$**); 118.3 (pyridyl-**$C^3$**); 105.1 (pyrazole-**$C^4$**); 72.8 (TIPSO-$CH_2$-**C**$H_2$); 71.8 (**C**$H_2OCH_2$-PEG); 70.9, 70.8, 70.7, 70.7, 70.7, 70.2 (**C**$_{aliphaticPEG}$); 63.0 (**C**$H_2$-OTIPS); 54.8 (napthyl-**C**$H_2$N); 18.0, 12.0 (O**TIPS**).

**High-resolution ES-MS**: *m/z* observed, 1255.7489. Calculated for $C_{68}H_{107}N_6O_{12}Si_2^+$ [$M + H^+$], 1255.7480.

**$\nu_{max}$/cm$^{-1}$**: 2941m, 2922m, 2865s, 1728w, 1607w, 1560w, 1459m, 1351m, 1326m, 1284m, 1247m, 1098s, 1046m, 882m, 788m, 770m, 680m.

**L$^{PEG5\text{-}OH}$ (12)**

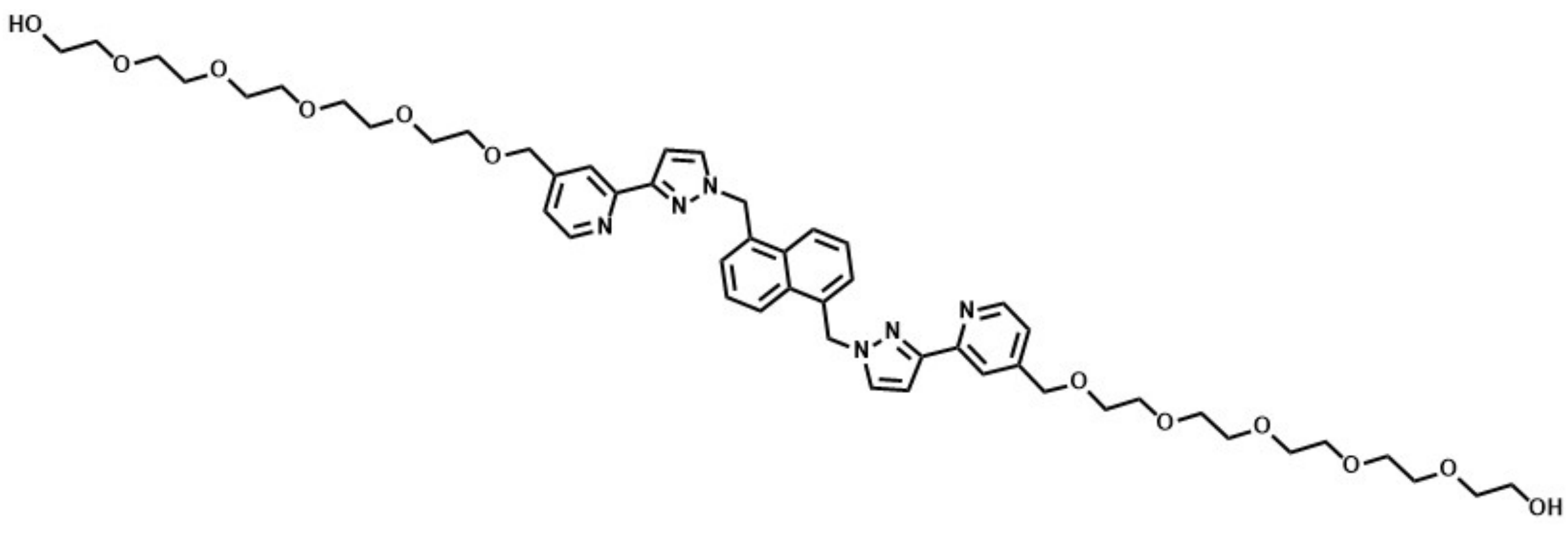


A solution of **11** (2.40 g, 1.93 mmol, 1.0 equiv.) and tetrabutylammonium fluoride (1 M in dry THF, 1.66 mL, 5.78 mmol, 3.0 equiv.) in THF (100 mL) was stirred at RT overnight. After 16

h, THF was removed. The crude oil was purified through a silica column using $CH_2Cl_2$/MeOH (96:4) as the eluent. Yield: 970 mg, 1.03 mmol, 54% (yellow oil).

**$^1$H-NMR** (400 MHz, $CDCl_3$, 298 K) *δ*ppm: 8.58 (2H, d, J = 4.5 Hz, pyridyl-**$H^6$**); 8.02 (2H, d, J = 8.4 Hz, naphthyl-**$H^{4/8}$**); 7.90 (2H, s, pyridyl-**$H^3$**); 7.46 (2H, t, J = 7.6 Hz, naphthyl-**$H^{3/7}$**); 7.30 (2H, d, J = 7.1 Hz, naphthyl-**$H^{2/6}$**); 7.25-7.21 (4H, m, pyrazole-**$H^5$** + pyridyl-**$H^5$**); 6.85 (2H, d, J = 1.8 Hz, pyrazole-**$H^4$**); 5.84 (4H, s, napthyl-C**$H_2$**N); 4.61 (4H, s, C**$H_2$**$OCH_2$-PEG); 3.71-3.58 (36H, m, **H**$_{aliphaticPEG}$); 3.56-3.54 (4H, m, HO-$CH_2$-C**$H_2$**); 2.57 (2H, brs, $CH_2$-$CH_2$-O**H**).

**$^{13}$C-NMR** (100 MHz, $CDCl_3$, 298 K) *δ*ppm: 152.3 (pyridyl-**$C^2$**); 151.7 (pyrazole-**$C^3$**); 149.7 (pyridyl-**$C^6$**); 148.3 (pyridyl-**$C^4$**); 132.4 (naphthyl-**$C^{1/5}$** or naphthyl-**$C^{9/10}$**); 131.8 (naphthyl-**$C^{9/10}$** or naphthyl-**$C^{1/}$**); 131.0 (pyrazole-**$C^5$**), 127.4 (naphthyl-**$C^{2/6}$**); 126.5 (naphthyl-**$C^{3/7}$**); 124.4 (naphthyl-**$C^{4/8}$**); 120.8 (pyridyl-**$C^5$**); 118.3 (pyridyl-**$C^3$**); 105.1 (pyrazole-**$C^4$**); 72.8 (HO-$CH_2$-**C**$H_2$); 71.8 (**C**$H_2OCH_2$-PEG); 70.7, 70.6, 70.6, 70.6, 70.3, 70.2 (**C**$_{aliphaticPEG}$); 61.7 (**C**$H_2$-OH); 54.7 (napthyl-**C**$H_2$N).

**High-resolution ES-MS**: *m/z* observed, 965.4626. Calculated for $C_{50}H_{66}N_6O_{12}Na^+$ [$M$ + $Na^+$], 965.4631.

**$\nu_{max}$/cm$^{-1}$**: 3412m (br), 2872m (br), 1720m, 1607m, 1560m, 1459m, 1350m, 1284m, 1247m, 1094s, 943m, 837m, 790m, 767m.

**L$^{PEG5\text{-}CC}$ (13)**

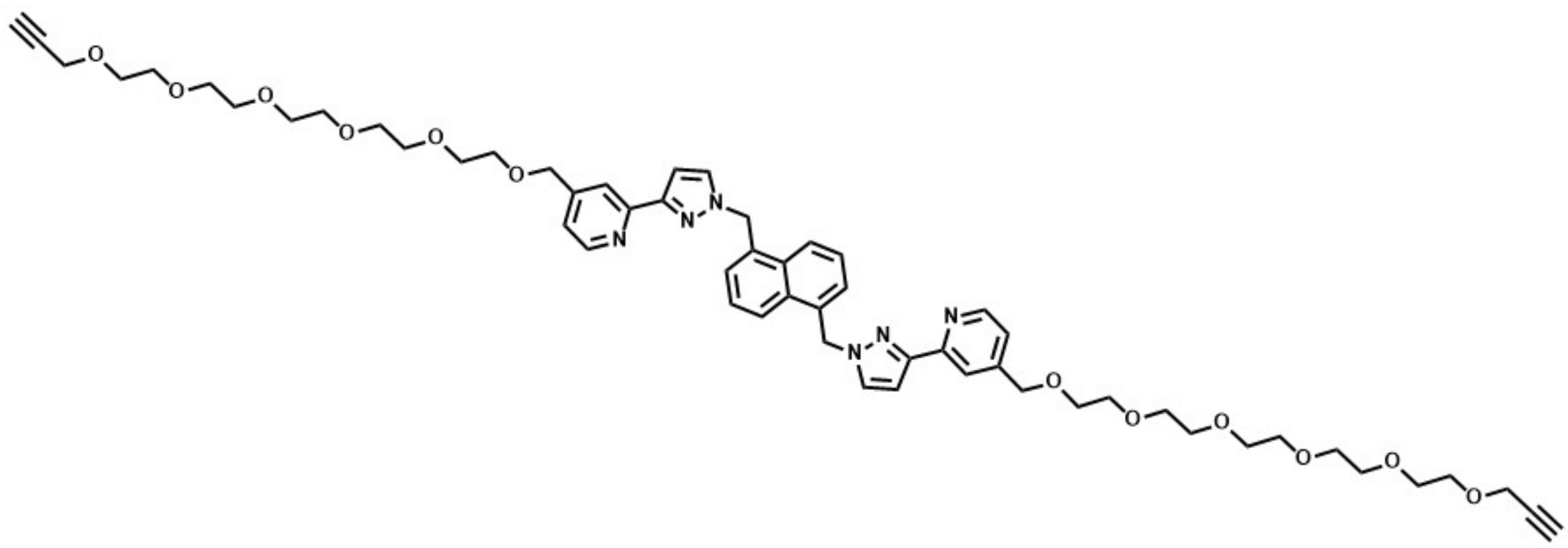

NaH (64 mg, 1.6 mmol, 60 wt. % dispersion in mineral oil, 5.0 equiv.) and 15-crown-5 ether (0.13 mL, 0.64 mmol, 2.0 equiv.) were added to a flame dried two neck RBF charged with dry THF (50 mL): the mixture was stirred for 30 mins under $N_2$. To this suspension, a solution of

**12** (300 mg, 0.32 mmol, 1.0 equiv.) in dry THF (100 mL) was transferred via cannula and the resulting suspension was refluxed for 2 h before the addition of propargyl bromide (106 μL, 0.95 mmol, 80 wt. % in toluene, 3.0 equiv.) with an instant colour change to brown observed. The mixture was refluxed for 72 h, with a darker brown colour developing over time. Once cooled to RT, the reaction was quenched with the addition of MeOH (50 mL) and the solvents evaporated to give a brown oil. The mineral oil was removed by filtration through a celite®/$CH_2Cl_2$ plug. The crude product was then purified through a silica column using $CH_2Cl_2$/MeOH (95:5) as eluent. Yield: 254 mg, 0.25 mmol, 78% (brown oil).

**$^1$H-NMR** (400 MHz, $CDCl_3$, 298 K) *δ*ppm: 8.55 (2H, d, J = 5.0 Hz, pyridyl-**H$^6$**); 8.00 (2H, d, J = 8.5 Hz, naphthyl-**H$^{4/8}$**); 7.88 (2H, s, pyridyl-**H$^3$**); 7.44 (2H, t, J = 7.8 Hz, naphthyl-**H$^{3/7}$**); 7.28 (2H, d, J = 7.1 Hz, naphthyl-**H$^{2/6}$**); 7.22 (2H, d, J = 2.3 Hz, pyrazole-**H$^5$**); 7.21 (2H, d, J = 5.1 Hz, pyridyl-**H$^5$**); 6.85 (2H, d, J = 2.2 Hz, pyrazole-**H$^4$**); 5.82 (4H, s, napthyl-C**H$_2$**N); 4.59 (4H, s, C**H$_2$**OCH$_2$-PEG); 4.16-4.13 (4H, d, J = 2.3 Hz, O-C**H$_2$**-C≡C); 3.69-3.56 (40H, m, **H**$_{aliphaticPEG}$); 2.40 (2H, t, J = 2.4 Hz, CH$_2$-C≡C-**H**).

**$^{13}$C-NMR** (100 MHz, $CDCl_3$, 298 K) *δ*ppm: 152.0 (pyridyl-**C$^2$**); 151.4 (pyrazole-**C$^3$**); 149.3 (pyridyl-**C$^6$**); 148.5 (pyridyl-**C$^4$**); 132.4 (naphthyl-**C$^{1/5}$** or naphthyl-**C$^{9/10}$**); 131.7 (naphthyl-**C$^{9/10}$** or naphthyl-**C$^{1/5}$**); 130.9 (pyrazole-**C$^5$**); 127.3 (naphthyl-**C$^{2/6}$**); 126.5 (naphthyl-**C$^{3/7}$**); 124.3 (naphthyl-**C$^{4/8}$**); 120.7 (pyridyl-**C$^5$**); 118.3 (pyridyl-**C$^3$**); 105.1 (pyrazole-**C$^4$**); 79.7 (**C**≡C-H); 74.6 (C≡**C**-H); 71.7 (**C**H$_2$OCH$_2$-PEG); 70.7, 70.6, 70.4, 70.2 (**C**$_{aliphaticPEG}$); 69.1 (**C**H$_2$-O-CH$_2$-C≡C); 58.4 (**C**H$_2$-C≡C); 54.7 (napthyl-**C**H$_2$N).

**High-resolution ES-MS**: *m/z* observed, 1019.5126. Calculated for $C_{56}H_{71}N_6O_{12}^+$ [*M* + H$^+$], 1019.5124.

**$\nu_{max}$/cm$^{-1}$**: 3439w (br), 3252w, 2868m (br), 2113w, 1720m, 1607m, 1560m, 1459m, 1351m, 1284m, 1247m, 1087s, 943m, 837m, 790m, 760m.

**L$^{PEG5-XX-Ac}$ (14)**

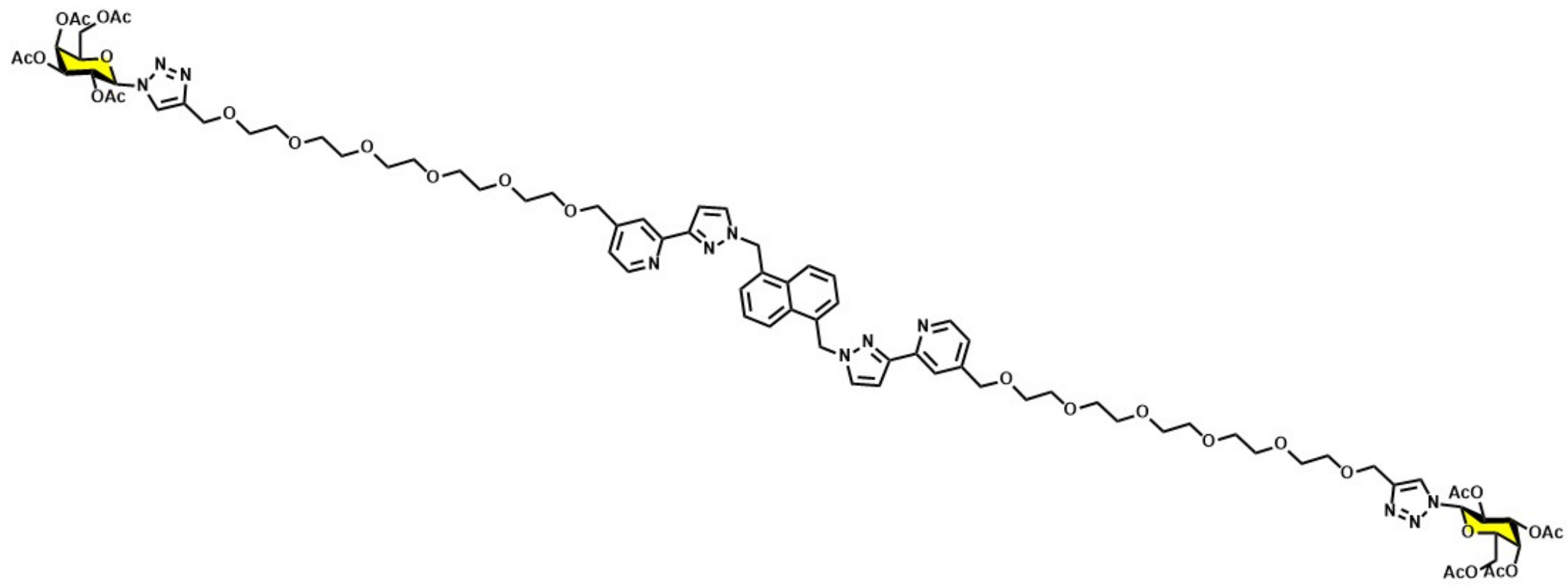

This was prepared using general procedure **A**, starting from **L$^{PEG5-CC}$** (125 mg, 0.12 mmol, 1.0 equiv.) and 1-azido-1-deoxy-β-D-galactopyranoside tetraacetate (114 mg, 0.31 mmol, 2.5 equiv.). Yield: 143 mg, 80 μmol, 66%.

**$^{1}$H-NMR** (400 MHz, $CDCl_3$, 298 K) *δ*ppm: 8.56 (2H, d, J = 5.0 Hz, pyridyl-**H$^{6}$**); 8.01 (2H, d, J = 8.4 Hz, naphthyl-**H$^{4/8}$**); 7.88 (2H, s, pyridyl-**H$^{3}$**); 7.82 (2H, s, triazole-**H$^{5}$**); 7.45 (2H, t, J = 7.7 Hz, naphthyl-**H$^{3/7}$**); 7.29 (2H, d, J = 6.9 Hz, naphthyl-**H$^{2/6}$**); 7.22 (2H, d, J = 2.2 Hz, pyrazole-**H$^{5}$**); 7.20 (2H, d, J = 5.0 Hz, pyridyl-**H$^{5}$**); 6.82 (2H, d, J = 2.3 Hz, pyrazole-**H$^{4}$**); 5.86-5.79 (6H, m, napthyl-C**H$_2$**N + galactose C$^{1}$-**H**); 5.54-5.50 (4H, m, galactose C$^{2}$-**H** + galactose C$^{4}$-**H**); 5.23-5.19 (2H, dd, J = 10.3, 3.4 Hz, galactose C$^{3}$-**H**); 4.65 (4H, s, $CH_2OC$**H$_2$**-triazole); 4.56 (4H, s, C**H$_2$**$OCH_2$-PEG); 4.22-4.07 (6H, m, galactose-C**H$_a$**H$_b$ + galactose-CH$_a$**H$_b$** + galactose C$^{5}$-**H** ); 3.67-3.57 (40H, m, **H**$_{aliphaticPEG}$); 2.18, 2.00, 1.97, 1.85 (24H, s, COC**H$_3$**).

**$^{13}$C-NMR** (100 MHz, $CDCl_3$, 298 K) *δ*ppm: 170.3, 170.0, 169.8, 169.0 (**C**$OCH_3$); 152.3 (pyridyl-**C$^{2}$**); 151.7 (pyrazole-**C$^{3}$**); 149.6 (pyridyl-**C$^{6}$**); 148.2 (pyridyl-**C$^{4}$**); 145.9 (triazole-**C$^{4}$**); 132.4 (naphthyl-**C$^{1/5}$** or naphthyl-**C$^{9/10}$**); 131.7 (naphthyl-**C$^{9/10}$** or naphthyl-**C$^{1/}$**); 130.9 (pyrazole-**C$^{5}$**), 127.4 (naphthyl-**C$^{2/6}$**); 126.5 (naphthyl-**C$^{3/7}$**); 124.4 (naphthyl-**C$^{4/8}$**); 121.2 (triazole-**C$^{5}$**); 120.7 (pyridyl-**C$^{5}$**); 118.3 (pyridyl-**C$^{3}$**); 105.0 (pyrazole-**C$^{4}$**); 86.2 (galactose-**C$^{1}$**); 74.0 (galactose-**C$^{5}$**); 71.7 (**C**H$_2$OCH$_2$-PEG); 70.8, 70.7, 70.6, 70.5, 70.2, 69.8 (**C**$_{aliphaticPEG}$ + galactose-**C$^{3}$**); 67.9 (galactose-**C$^{2}$**); 66.9 (galactose-**C$^{4}$**); 64.5 (CH$_2$O**C**H$_2$-triazole); 61.2 (galactose-**C$^{6}$**); 54.7 (napthyl-**C**H$_2$N); 20.7, 20.5, 20.3 (CO**C**H$_3$).

**High-resolution ES-MS**: *m/z* observed, 1787.7360. Calculated for $C_{84}H_{108}N_{12}NaO_{30}^{+}$ [*M* + Na$^{+}$], 1787.7187. Observed, 905.3489. Calculated for $C_{84}H_{108}N_{12}Na_2O_{30}^{2+}$ [*M* + 2Na$^{+}$], 905.3539.

**ν$_{max}$/cm$^{-1}$**: 2919m, 2871m, 1735s, 1608m, 1459m, 1351m, 1260m, 1094s, 1021m, 798m.

**L$^{PEG5\text{-}XX}$ (15)**

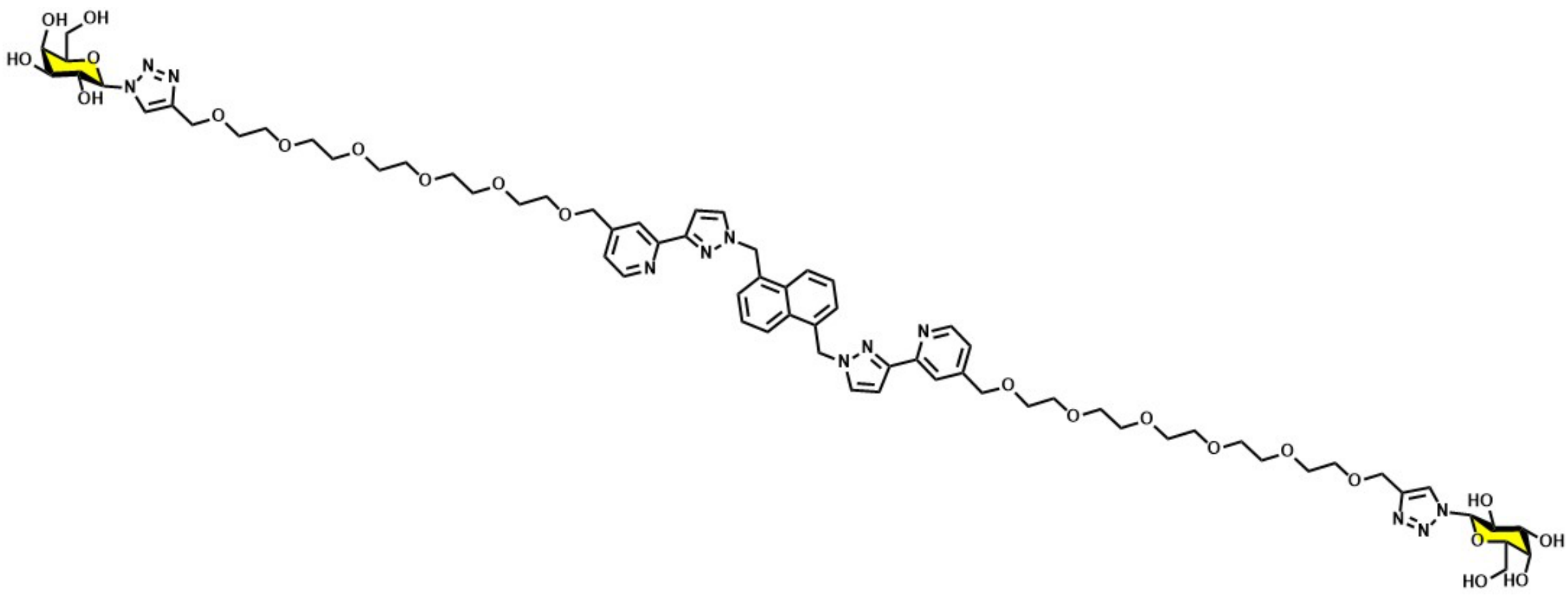

This was prepared following general procedure **B** above starting from acetyl-protected compound **14** (135 mg, 81 µmol, 1.0 equiv.). The crude product was then immediately purified by size-exclusion chromatography on G-50 Sephadex®, using $H_2O$ as the eluent, and lyophilised. Yield: 114 mg, 80 µmol, 98%.

**$^{1}$H-NMR** (500 MHz, DMSO-$d_6$, 298 K) *δ*ppm: 8.51 (2H, d, J = 5.0 Hz, pyridyl-**H$^{6}$**); 8.22 (2H, d, J = 8.8 Hz, naphthyl-**H$^{4/8}$**); 8.20 (2H, s, triazole-**H$^{5}$**); 7.87-7.86 (4H, m, pyrazole-**H$^{5}$** + pyridyl-**H$^{3}$**); 7.57 (2H, t, J = 7.7 Hz, naphthyl-**H$^{3/7}$**); 7.27 (2H, d, J = 7.1 Hz, naphthyl-**H$^{2/6}$**); 7.22 (2H, d, J = 4.8 Hz, pyridyl-**H$^{5}$**); 6.85 (2H, d, J = 2.2 Hz, pyrazole-**H$^{4}$**); 5.93 (4H, s, napthyl-C**H$_2$**N); 5.46 (2H, d, J = 9.2 Hz, galactose C$^{1}$-**H**); 4.58 (4H, s, C**H$_2$**OCH$_2$-PEG); 4.52 (4H, s, CH$_2$OC**H$_2$**-triazole); 4.04 (2H, t, J = 9.3 Hz, galactose C$^{2}$-**H**); 3.78 (2H, d, J = 2.6 Hz, galactose C$^{5}$-**H**); 3.70 (2H, t, J = 6.0 Hz, galactose C$^{4}$-**H**); 3.62-3.46 (46H, m, **H**$_{aliphaticPEG}$ + galactose C$^{3}$-**H** + galactose-C**H$_a$**H$_b$ + galactose-CH$_a$**H$_b$**).

**$^{13}$C-NMR** (125 MHz, DMSO-$d_6$, 298 K): δ 151.8 (pyridyl-**C$^{2}$**); 151.0 (pyrazole-**C$^{3}$**); 149.2 (pyridyl-**C$^{6}$**); 148.4 (pyridyl-**C$^{4}$**); 143.9 (triazole-**C$^{4}$**); 133.8 (naphthyl-**C$^{1/5}$** or naphthyl-**C$^{9/10}$**); 132.4 (pyrazole-**C$^{5}$**); 130.9 (naphthyl-**C$^{9/10}$** or naphthyl-**C$^{1/5}$**); 126.3 (naphthyl-**C$^{2/6}$**); 126.2 (naphthyl-**C$^{3/7}$**); 123.8 (naphthyl-**C$^{4/8}$**); 122.8 (triazole-**C$^{5}$**); 120.5 (pyridyl-**C$^{5}$**); 117.1 (pyridyl-**C$^{3}$**); 104.5 (pyrazole-**C$^{4}$**); 88.1 (galactose-**C$^{1}$**); 78.4 (galactose-**C$^{4}$**); 73.8 (galactose-**C$^{3}$**); 70.5 (**C**H$_2$OCH$_2$-triazole); 69.8 (galactose-**C$^{2}$**); 69.8, 69.7, 69.7, 69.6, 69.4, 69.1, 69.0 (**C**$_{aliphaticPEG}$); 68.4 (galactose-**C$^{5}$**); 63.4 (CH$_2$O**C**H$_2$-triazole); 60.4 (galactose-**C$^{6}$**); 53.3 (napthyl-**C**H$_2$N).

**High-resolution ES-MS**: *m/z* observed, 1451.7275. Calculated for $C_{68}H_{92}N_{12}NaO_{22}^{+}$ [*M* + $Na^{+}$], 1451.6341. Observed, 737.3571. Calculated for $C_{68}H_{92}N_{12}Na_{2}O_{22}^{2+}$ [*M* + $2Na^{+}$], 737.3117.

**$\nu_{max}$/cm$^{-1}$**: 3349w (br), 2870m, 1595m, 1560m, 1458w, 1349m, 1299w, 1246w, 1087s, 946m, 883m, 841m, 789m, 773m, 755m, 700m.

**$L^{PEG5\text{-}FF\text{-}Ac}$ (16)**

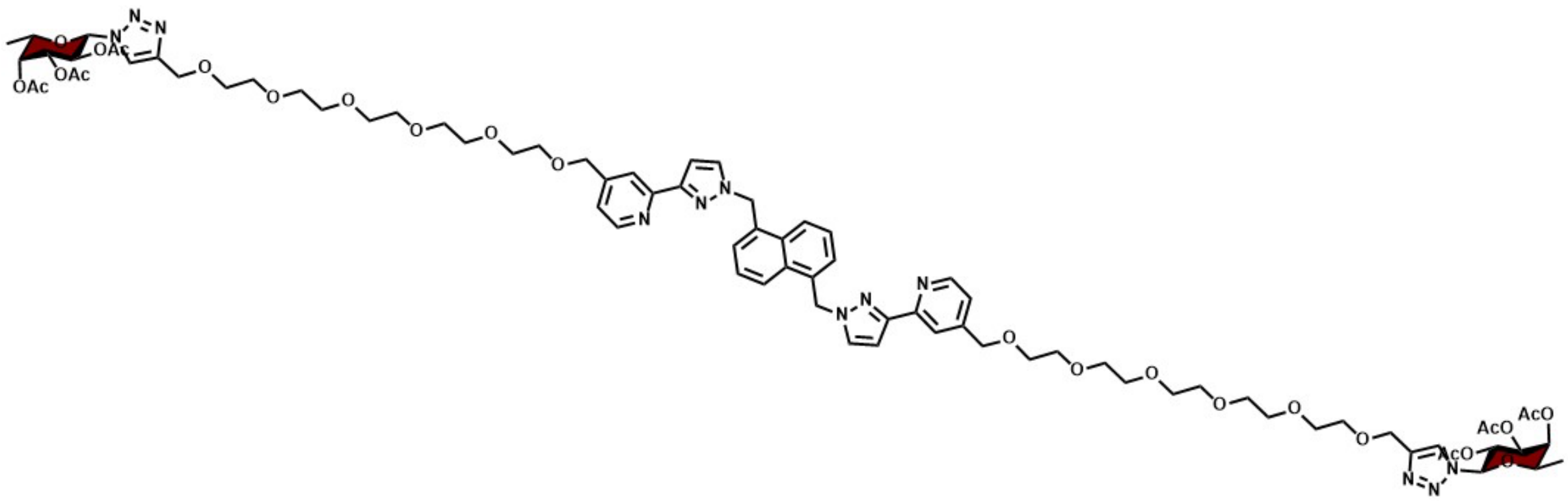

This was prepared using general procedure **A**, starting from **$L^{PEG5\text{-}CC}$** (144 mg, 0.14 mmol, 1.0 equiv.) and **2** (111 mg, 0.35 mmol, 2.5 equiv.). Yield: 153 mg, 90 μmol, 66%.

**$^{1}$H-NMR** (400 MHz, $CDCl_3$, 298 K) *δ*ppm: 8.54 (2H, d, J = 5.0 Hz, pyridyl-**H$^{6}$**); 7.99 (2H, d, J = 8.5 Hz, naphthyl-**H$^{4/8}$**); 7.87 (2H, s, pyridyl-**H$^{3}$**); 7.81 (2H, s, triazole-**H$^{5}$**); 7.43 (2H, t, J = 7.5 Hz, naphthyl-**H$^{3/7}$**); 7.27 (2H, d, J = 7.1 Hz, naphthyl-**H$^{2/6}$**); 7.22 (2H, d, J = 1.9 Hz, pyrazole-**H$^{5}$**); 7.19 (2H, d, J = 4.8 Hz, pyridyl-**H$^{5}$**); 6.83 (2H, d, J = 1.6 Hz, pyrazole-**H$^{4}$**); 5.82 (4H, s, napthyl-C**H$_2$**N); 5.75 (2H, d, J = 9.1 Hz, fucose C$^{1}$-**H**); 5.46 (2H, t, J = 9.9 Hz, fucose C$^{2}$-**H**); 5.33 (2H, d, J = 3.0 Hz, fucose C$^{4}$-**H**); 5.20-5.17 (2H, dd, J = 10.3, 3.4 Hz, fucose C$^{3}$-**H**); 4.63 (4H, s, $CH_2OC$**H$_2$**-triazole); 4.58 (4H, s, C**H$_2$**$OCH_2$-PEG); 4.05 (2H, q, J = 6.3 Hz, fucose C$^{5}$-**H**); 3.65-3.58 (40H, m, **H**$_{aliphaticPEG}$); 2.18, 1.95, 1.82 (18H, s, methyl C**H$_3$**); 1.19 (6H, d, J = 6.3 Hz, fucose C$^{6}$-**H**).

**$^{13}$C-NMR** (100 MHz, $CDCl_3$, 298 K) *δ*ppm: 170.3, 169.8, 169.1 (**C**$OCH_3$); 152.1 (pyridyl-**C$^{2}$**); 151.4 (pyrazole-**C$^{3}$**); 149.4 (pyridyl-**C$^{6}$**); 148.4 (pyridyl-**C$^{4}$**); 145.7 (triazole-**C$^{4}$**); 132.4 (naphthyl-**C$^{1/5}$** or naphthyl-**C$^{9/10}$**); 131.7 (naphthyl-**C$^{9/10}$** or naphthyl-**C$^{1/5}$**); 130.9 (pyrazole-**C$^{5}$**), 127.3 (naphthyl-**C$^{2/6}$**); 126.5 (naphthyl-**C$^{3/7}$**); 124.3 (naphthyl-**C$^{4/8}$**); 121.0 (triazole-**C$^{5}$**); 120.7 (pyridyl-**C$^{5}$**); 118.2 (pyridyl-**C$^{3}$**); 105.1 (pyrazole-**C$^{4}$**); 86.2 (fucose-**C$^{1}$**); 72.6 (fucose-**C$^{5}$**); 71.7 (**C**$H_2OCH_2$-PEG); 71.2 (fucose-**C$^{3}$**); 70.7, 70.5, 70.5, 70.4, 70.1 (**C**$_{aliphaticPEG}$); 69.8 (fucose-

**C**[4]); 69.6 (**C**$_{aliphaticPEG}$); 68.0 (fucose-**C**[2]); 64.5 ($CH_2OCH_2$-triazole); 54.6 (napthyl-**C**$H_2$N); 20.6, 20.5, 20.3 (CO**C**$H_3$); 16.0 (fucose-**C**[6]).

**High-resolution ES-MS**: *m/z* observed, 1671.7151. Calculated for $C_{80}H_{104}N_{12}NaO_{26}^{+}$ [*M* + Na$^+$], 1671.7077. Observed, 847.3481. Calculated for $C_{80}H_{104}N_{12}Na_2O_{26}^{2+}$ [*M* + 2Na$^+$], 847.3485.

**$\nu_{max}$/cm$^{-1}$**: 2924m, 2868m, 1750s, 1608w, 1459m, 1368m, 1238s, 1217s, 1092s, 1044s, 926m, 794m.

**L$^{PEG5-FF}$ (17)**

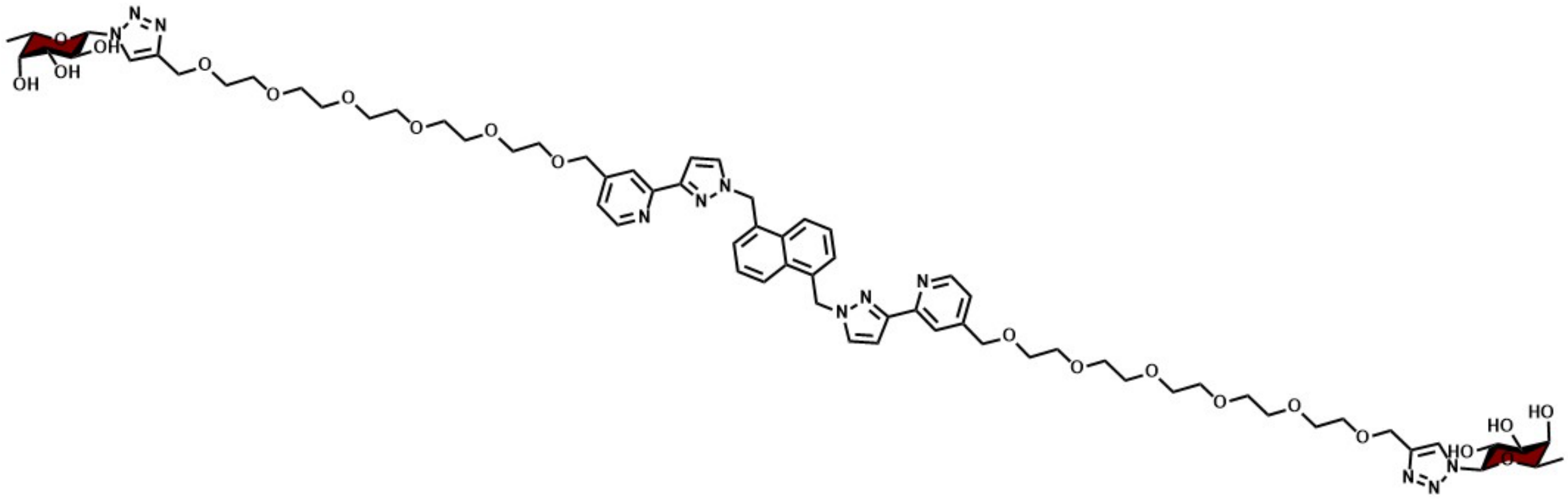

This was prepared following general procedure **B** above starting from acetyl-protected compound **16** (153 mg, 93 μmol, 1.0 equiv.). The crude product was then immediately purified by size-exclusion chromatography on G-50 Sephadex®, using $H_2O$ as the eluent and lyophilised. Yield: 128 mg, 92 μmol, 98%.

**$^1$H-NMR** (400 MHz, DMSO-$d_6$, 298 K) *δ*ppm: 8.52 (2H, d, J = 5.2 Hz, pyridyl-**H**[6]); 8.23 (2H, d, J = 8.5 Hz, naphthyl-**H**[4/8]); 8.18 (2H, s, triazole-**H**[5]); 7.93-7.89 (4H, m, pyrazole-**H**[5] + pyridyl-**H**[6]); 7.57 (2H, t, J = 8.0 Hz, naphthyl-**H**[3/7]); 7.31-7.25 (4H, m, naphthyl-**H**[2/6] + pyridyl-**H**[5]); 6.90 (2H, d, J = 1.6 Hz, pyrazole-**H**[4]); 5.95 (4H, s, napthyl-C**H**$_2$N); 5.44 (2H, d, J = 9.2 Hz, fucose C[1]-**H**); 4.60 (4H, s, C**H**$_2$OCH$_2$-PEG); 4.52 (4H, s, C**H**$_2$OCH$_2$-triazole); 3.99 (2H, t, J = 9.5 Hz, fucose C[2]-**H**); 3.86 (2H, q, J = 6.4 Hz, fucose C[5]-**H**); 3.64-3.43 (44H, m, **H**$_{aliphaticPEG}$ + fucose C[3]-**H** + fucose C[4]-**H**); 1.13 (6H, d, J = 6.3 Hz, fucose C[6]-**H**).

**$^{13}$C-NMR** (125 MHz, DMSO-$d_6$, 298 K) *δ*ppm: 151.7 (pyridyl-**C**[2]); 151.1 (pyrazole-**C**[3]); 149.2 (pyridyl-**C**[6]); 148.4 (pyridyl-**C**[4]); 143.9 (triazole-**C**[5]); 133.8 (naphthyl-**C**[1/5] or naphthyl-**C**[9/10]); 132.3 (naphthyl-**C**[1/5] or naphthyl-**C**[9/10]); 130.9 (pyrazole-**C**[5]); 126.2 (naphthyl-**C**[2/6]); 126.2 (naphthyl-**C**[3/7]); 123.8 (naphthyl-**C**[4/8]); 122.7 (triazole-**C**[5]); 120.5 (pyridyl-**C**[5]); 117.1 (pyridyl-

**C**$^3$); 104.4 (pyrazole-**C**$^4$); 88.1 (fucose-**C**$^1$); 73.9 (fucose-**C**$^3$); 73.2 (fucose-**C**$^5$); 71.1 (fucose-**C**$^4$); 70.5 (**C**$H_2OCH_2$-PEG); 69.8, 69.8, 69.7, 69.7, 69.6, 69.0, 69.0 (**C**$_{aliphaticPEG}$); 69.0 (fucose-**C**$^2$); 63.4 ($CH_2O$**C**$H_2$-triazole); 53.4 (napthyl-**C**$H_2N$); 16.5 (fucose-**C**$^6$).

**High-resolution ES-MS**: *m/z* observed, 721.3317. Calculated for $C_{68}H_{92}N_{12}Na_2O_{20}{}^{2+}$ [*M* + 2Na$^+$], 721.3168.

**$\nu_{max}$/cm$^{-1}$**: 3377m (br), 3140w, 2907m, 2872m, 1718m, 1610m, 1459m, 1349m, 1232m, 1087s, 999s, 880s, 792s, 770s, 732s.

## 2. Selected NMR spectra of ligands

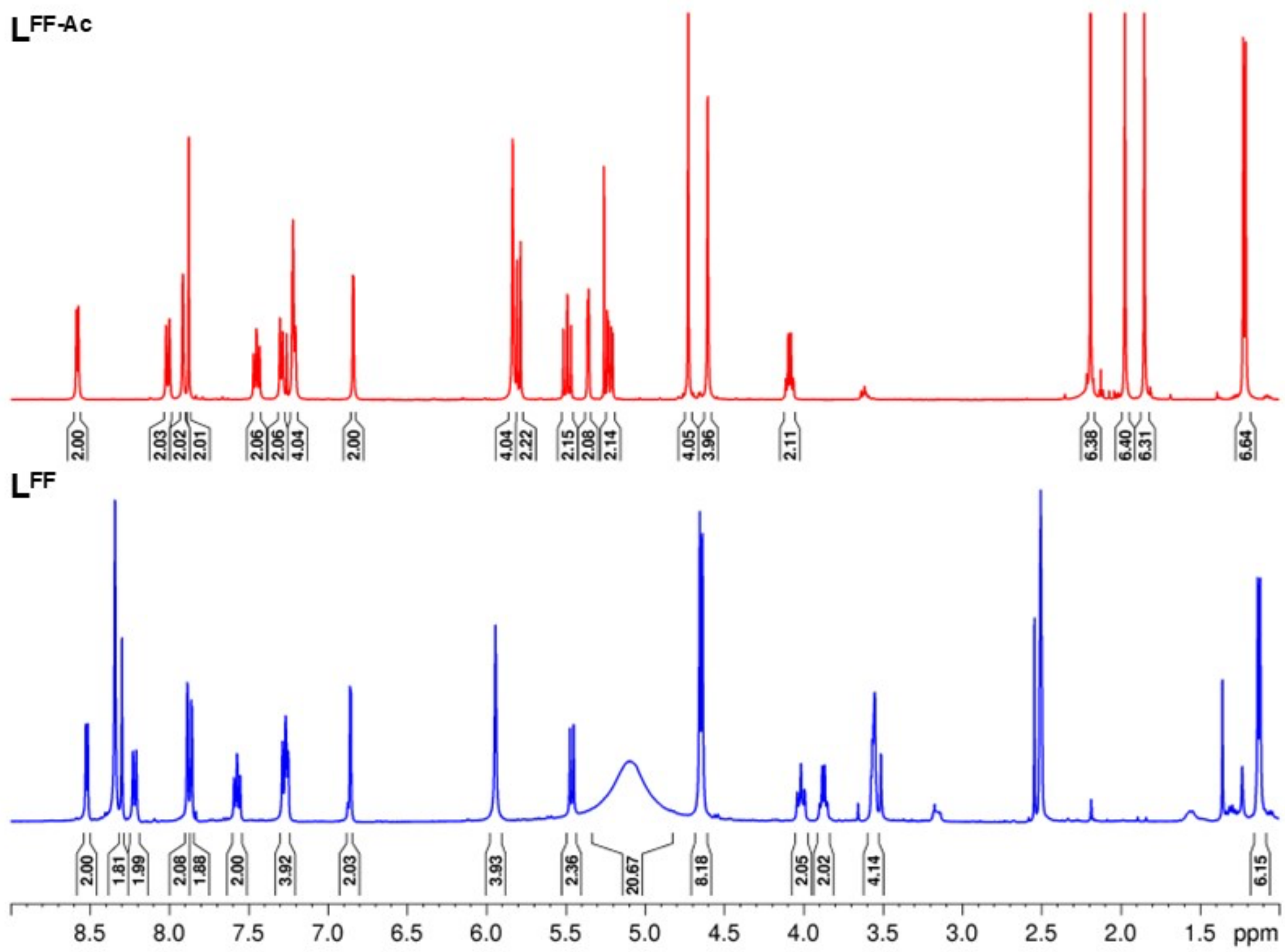


**Figure S1.** $^{1}$H NMR spectrum (400 MHz, $CDCl_3$, 298K) of $\mathbf{L^{FF\text{-}Ac}}$ and $^{1}$H NMR spectrum (400 MHz, DMSO-$d_6$, 298K) of $\mathbf{L^{FF}}$.

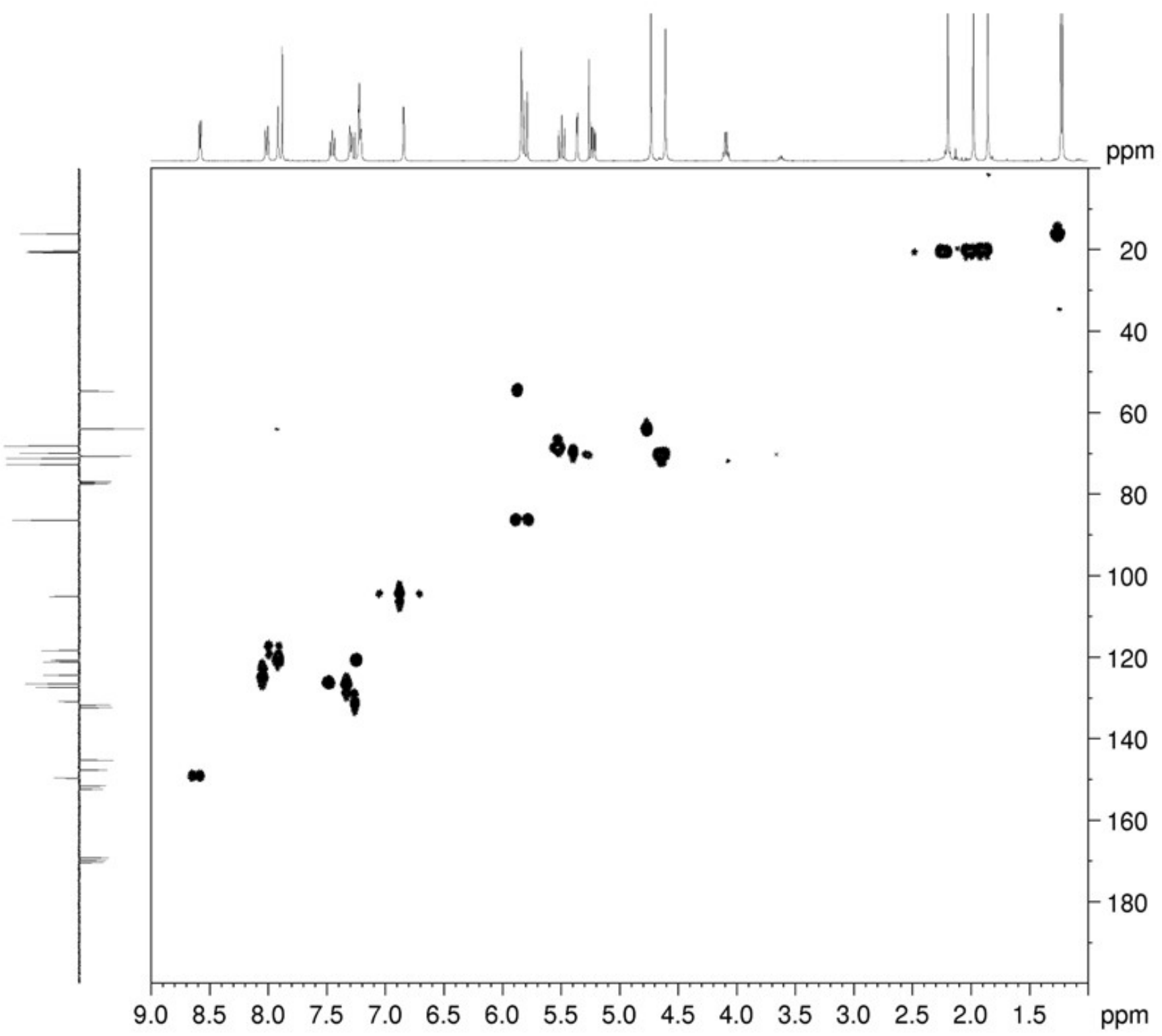


**Figure S2.** 2D $^{1}H$-$^{13}C$ HSQC NMR spectrum ($CDCl_3$, 298 K) of $\mathbf{L^{FF\text{-}Ac}}$.

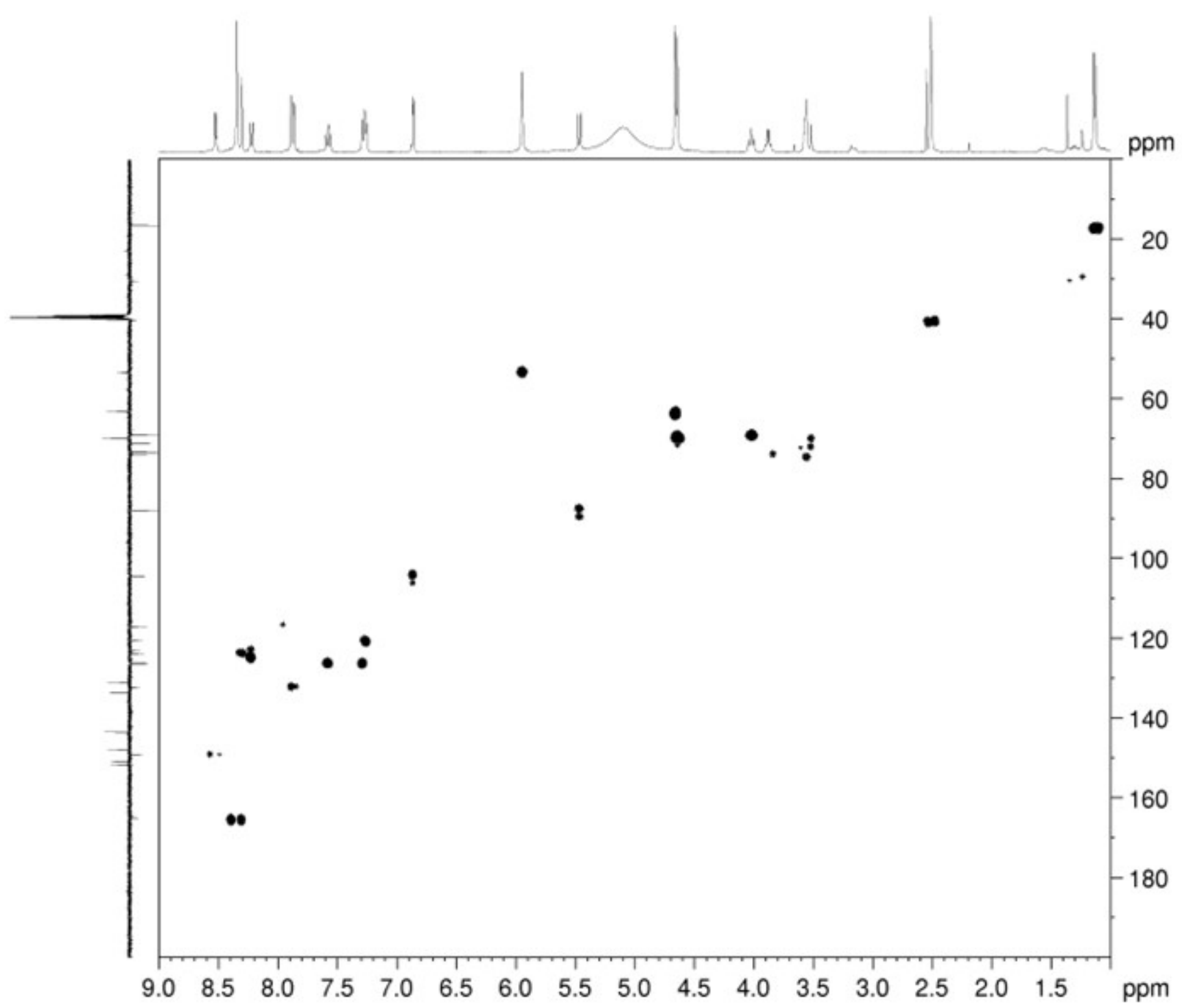


**Figure S3.** 2D $^{1}H$-$^{13}C$ HSQC NMR spectrum (DMSO-$d_6$, 298 K) of $\mathbf{L^{FF}}$.

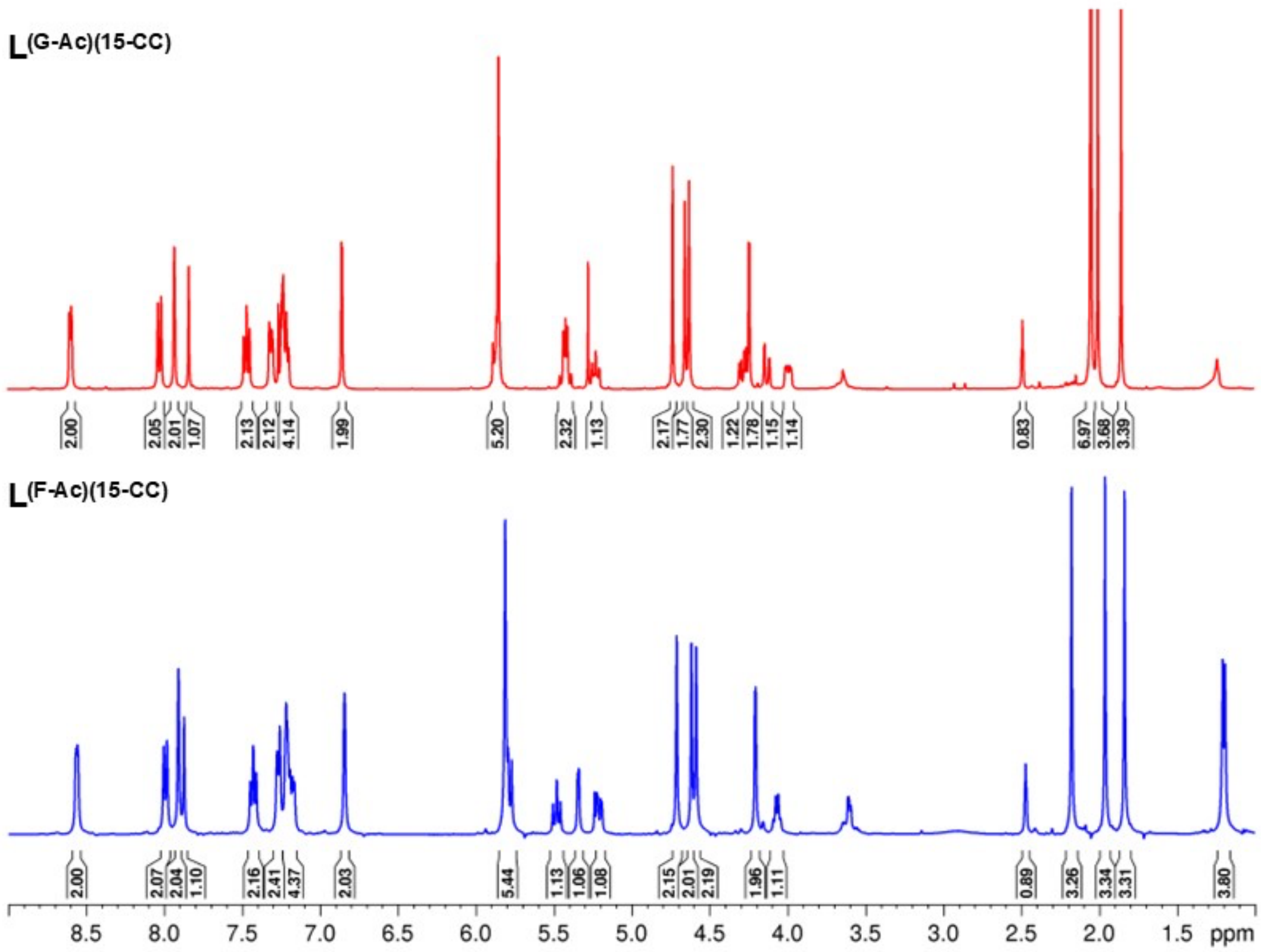


**Figure S4.** $^{1}H$ NMR spectra (400 MHz, $CDCl_3$, 298K) of **L^(G-Ac)(15-CC)** and **L^(F-Ac)(15-CC)**.

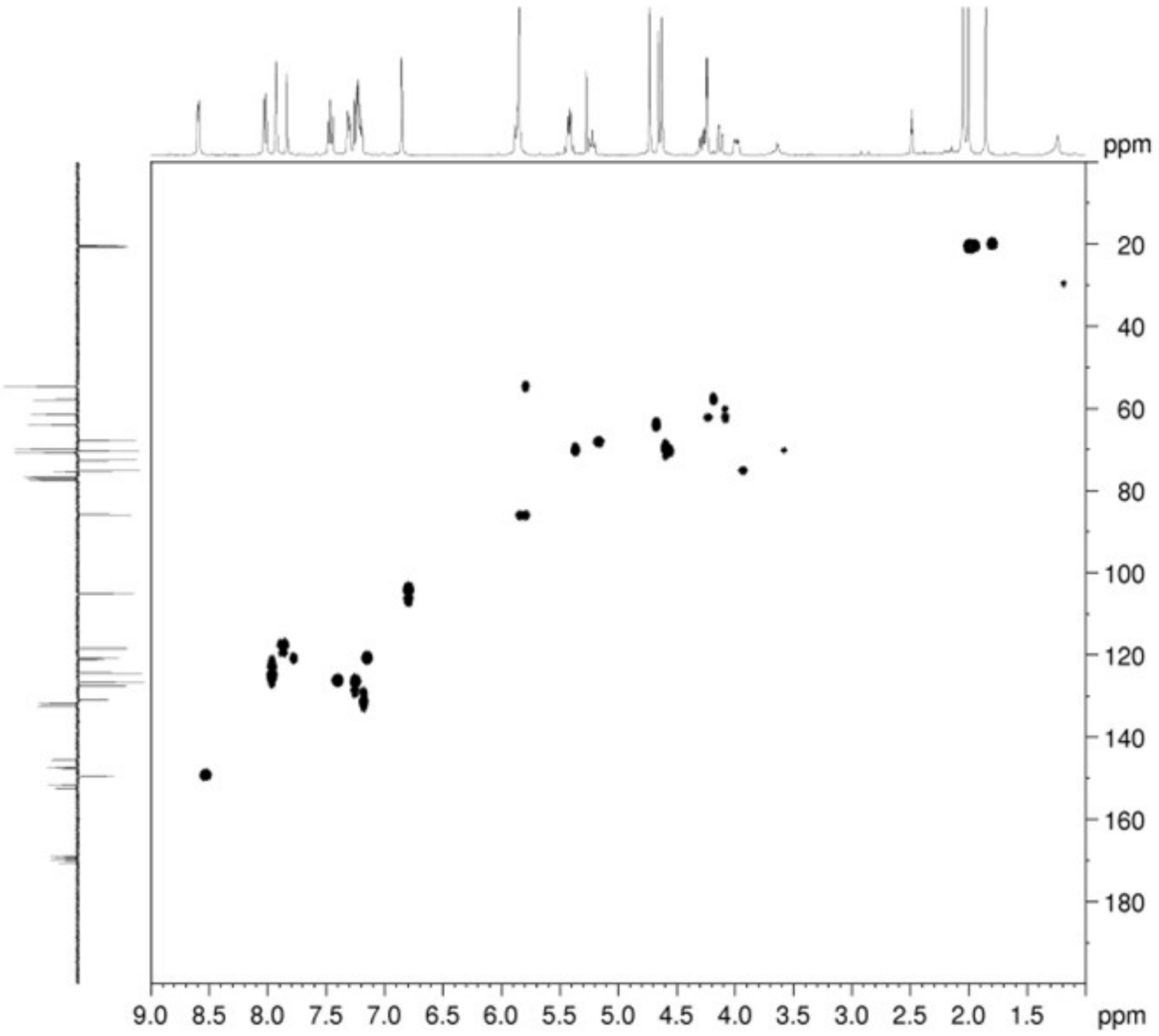


**Figure S5.** 2D $^{1}H$-$^{13}C$ HSQC NMR spectrum ($CDCl_3$, 298 K) of $\mathbf{L}^{\mathbf{(G\text{-}Ac)(15\text{-}CC)}}$.

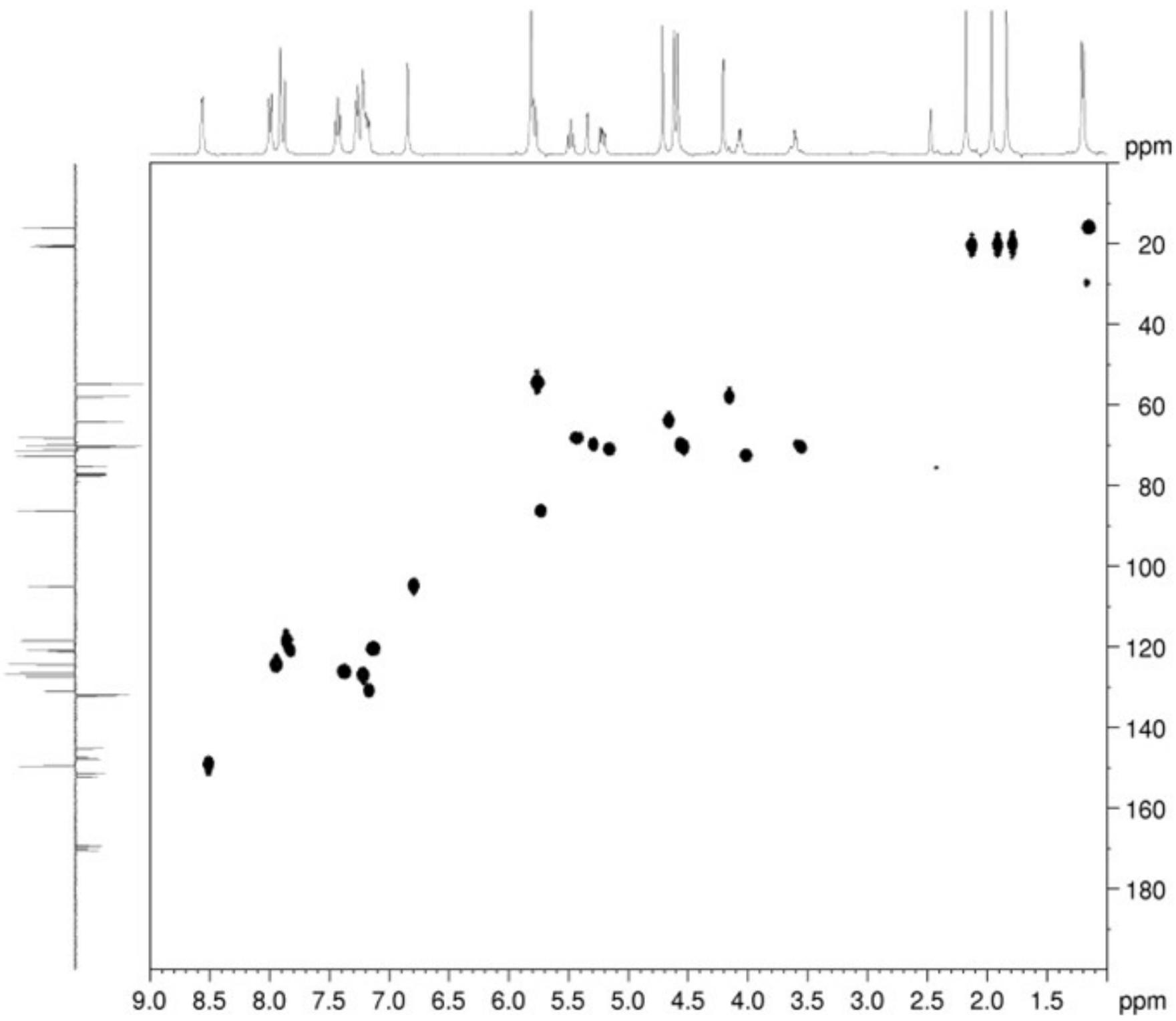


**Figure S6.** 2D $^{1}H$-$^{13}C$ HSQC NMR spectrum ($CDCl_3$, 298 K) of $\mathbf{L}^{\mathbf{(F\text{-}Ac)(15\text{-}CC)}}$.

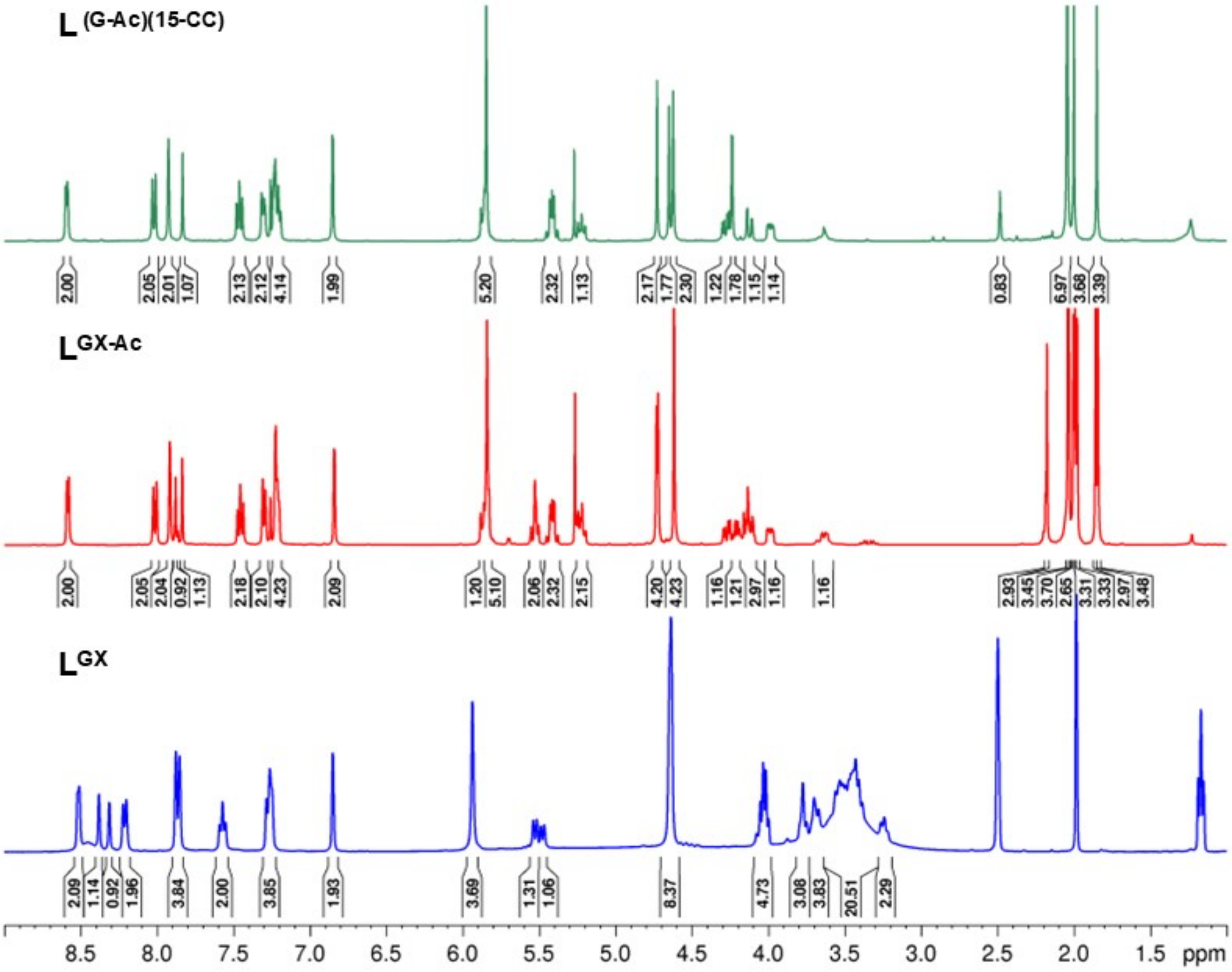


**Figure S7.** $^{1}$H NMR spectra (400 MHz, $CDCl_3$, 298K) of **L$^{(G-Ac)(15-CC)}$** and **L$^{GX-Ac}$**, and $^{1}$H NMR spectrum (400 MHz, DMSO-$d_6$, 298K) of **L$^{GX}$**. For ligand **L$^{GX}$**, residual amounts of EtOAc are present in the spectrum after washing.

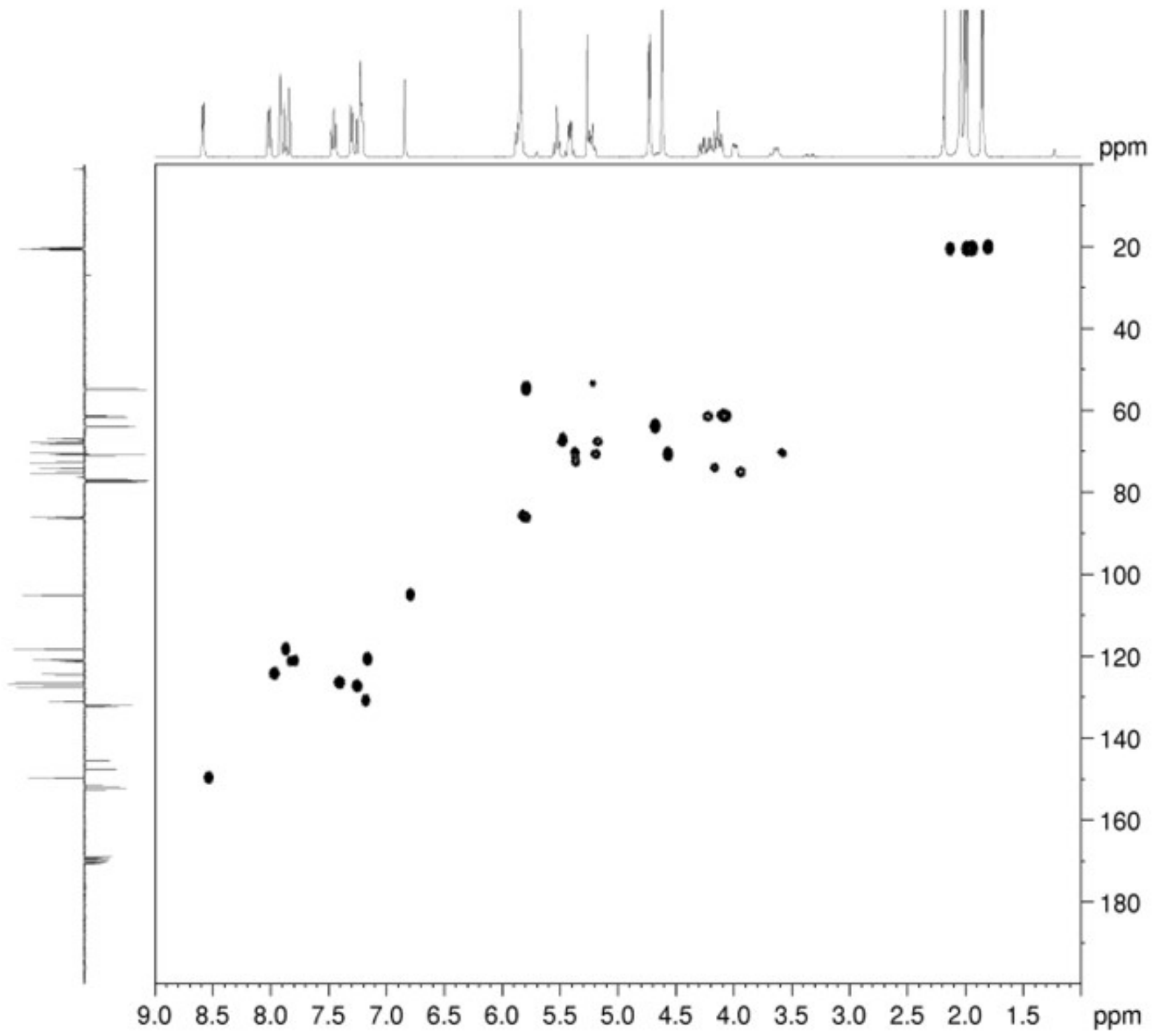


**Figure S8.** 2D $^{1}H$-$^{13}C$ HSQC NMR spectrum ($CDCl_3$, 298 K) of $\mathbf{L}^{\mathbf{GX\text{-}Ac}}$.

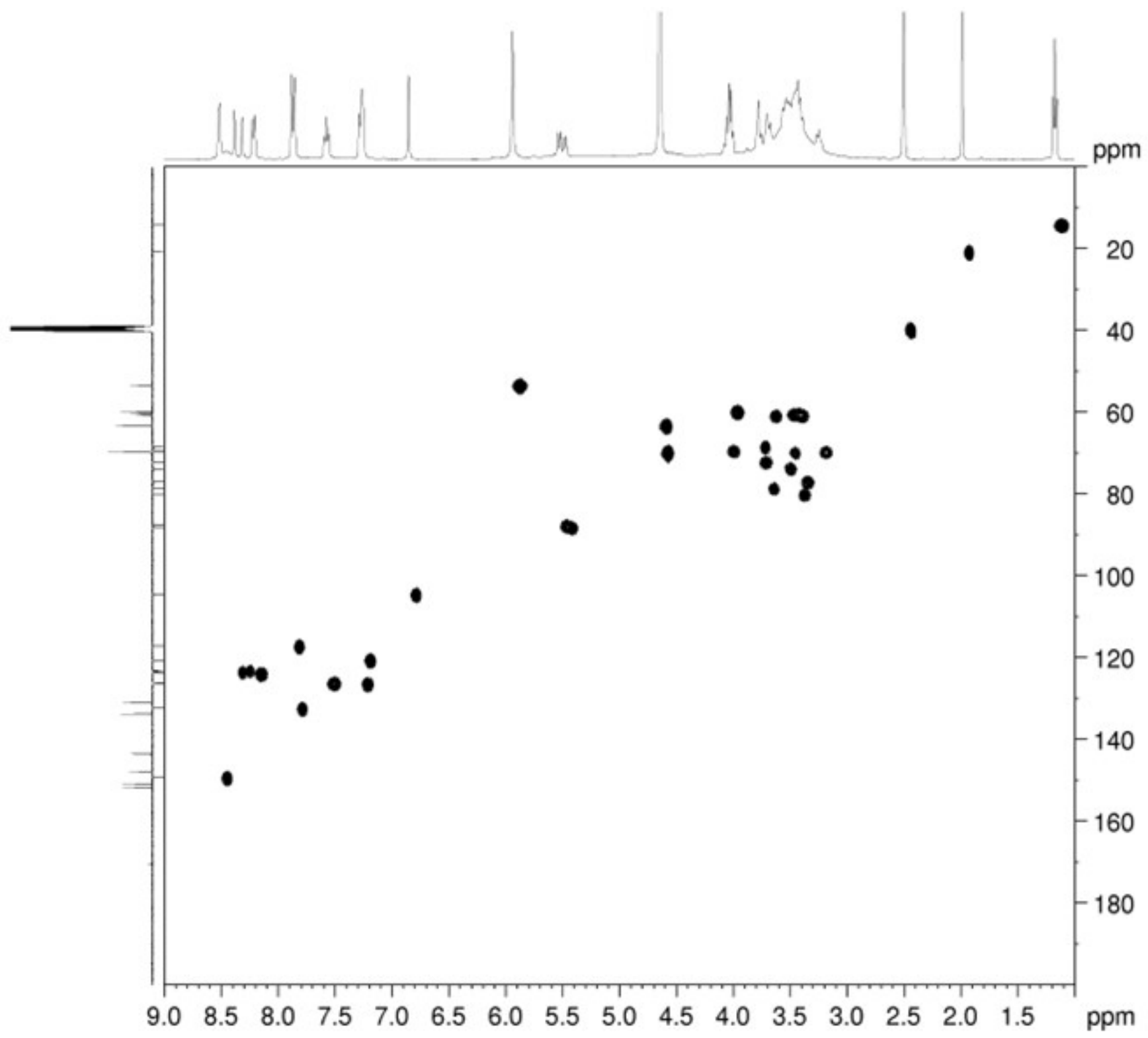


**Figure S9.** 2D $^{1}H$-$^{13}C$ HSQC NMR spectrum (DMSO-$d_6$, 298 K) of $\mathbf{L^{GX}}$. Residual amounts of EtOAc are present in the spectrum after washing.

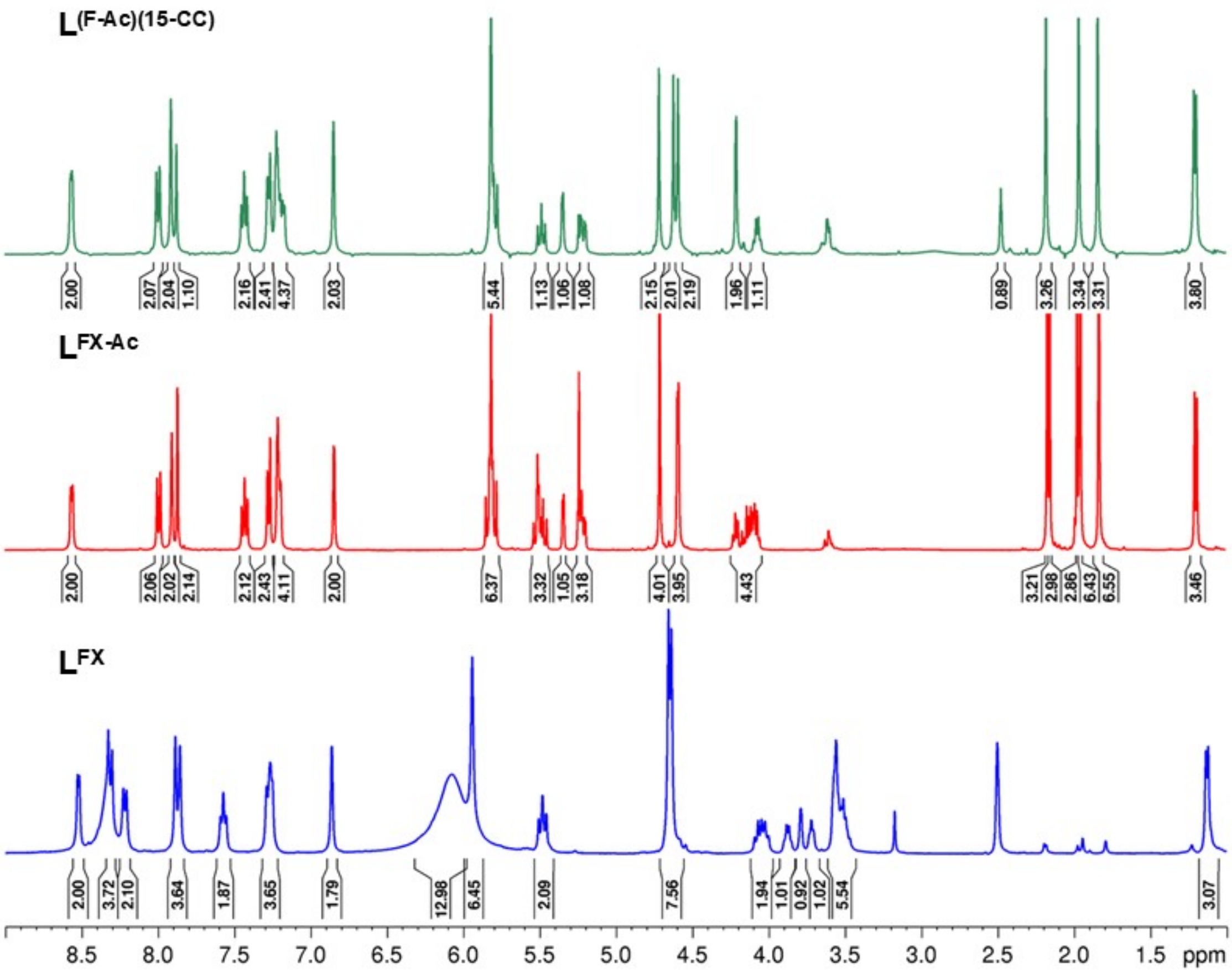


**Figure S10.** $^{1}$H NMR spectra (400 MHz, $CDCl_3$, 298K) of **L$^{(F-Ac)(15-CC)}$** and **L$^{FX-Ac}$**, and $^{1}$HNMR spectrum (400 MHz, DMSO-$d_6$, 298K) of **L$^{FX}$**.

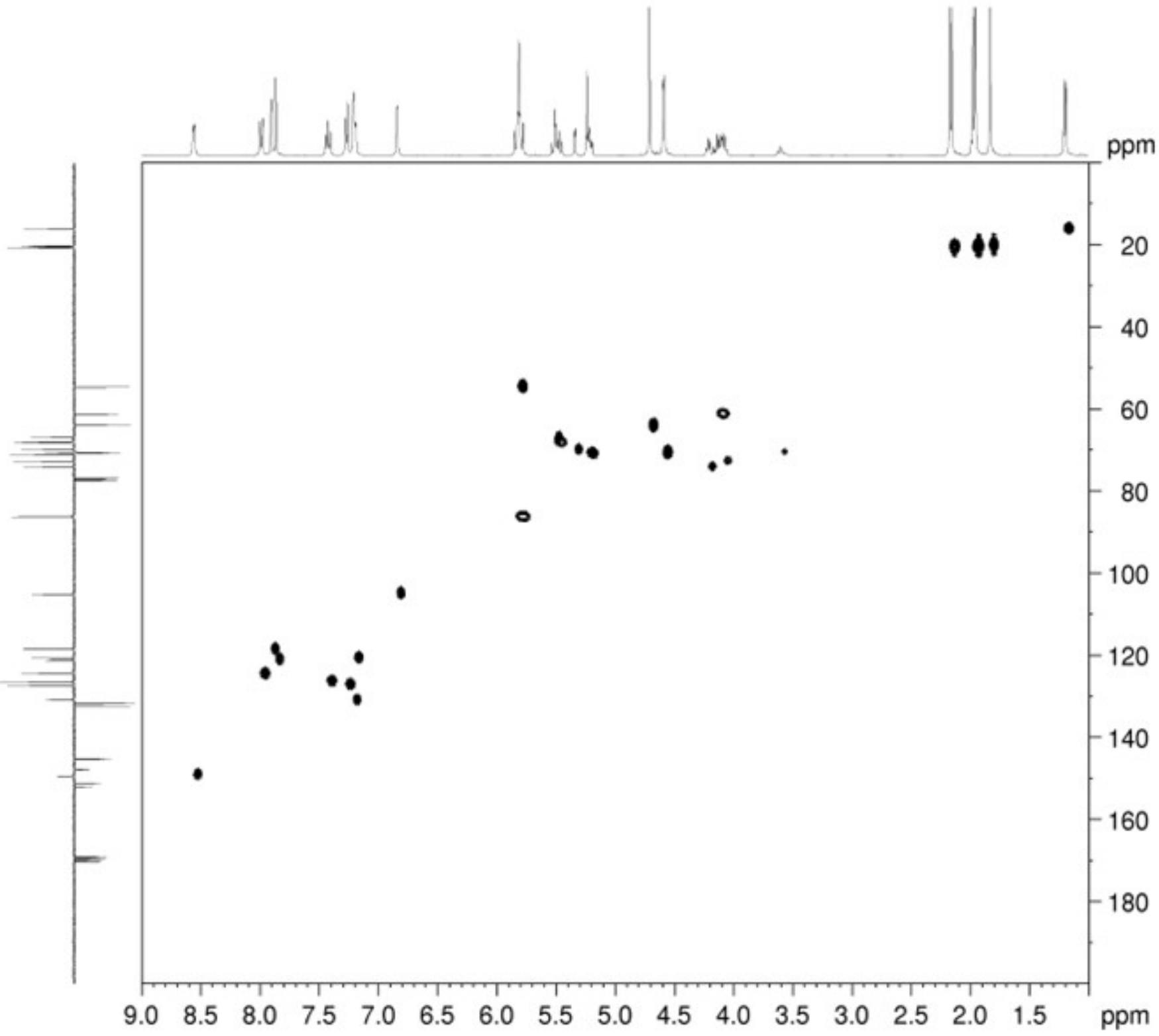


**Figure S11.** 2D $^{1}H$-$^{13}C$ HSQC NMR spectrum ($CDCl_3$, 298 K) of $\mathbf{L^{FX\text{-}Ac}}$.

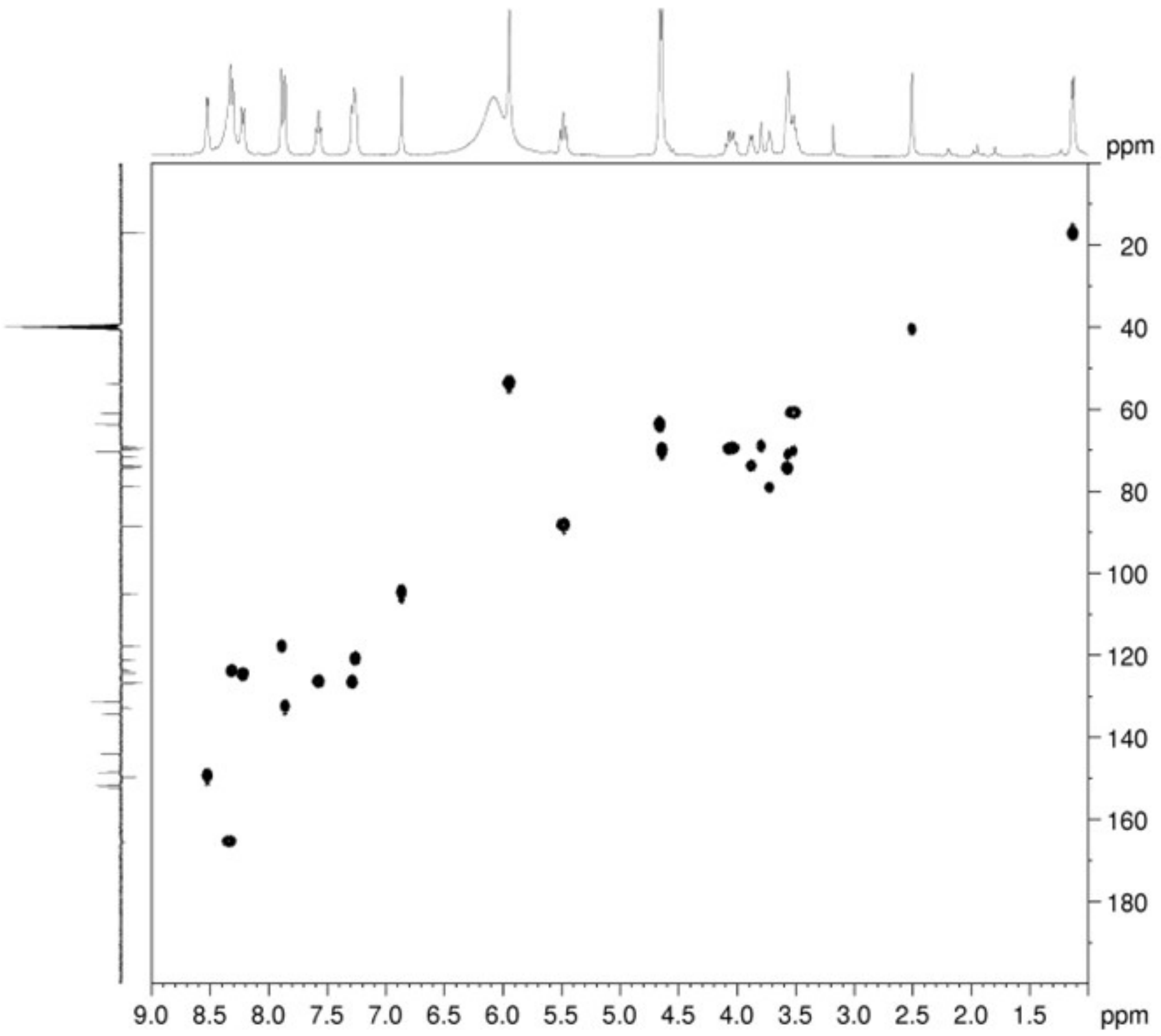


**Figure S12.** 2D $^{1}H$-$^{13}C$ HSQC NMR spectrum (DMSO-$d_6$, 298 K) of $\mathbf{L^{FX}}$.

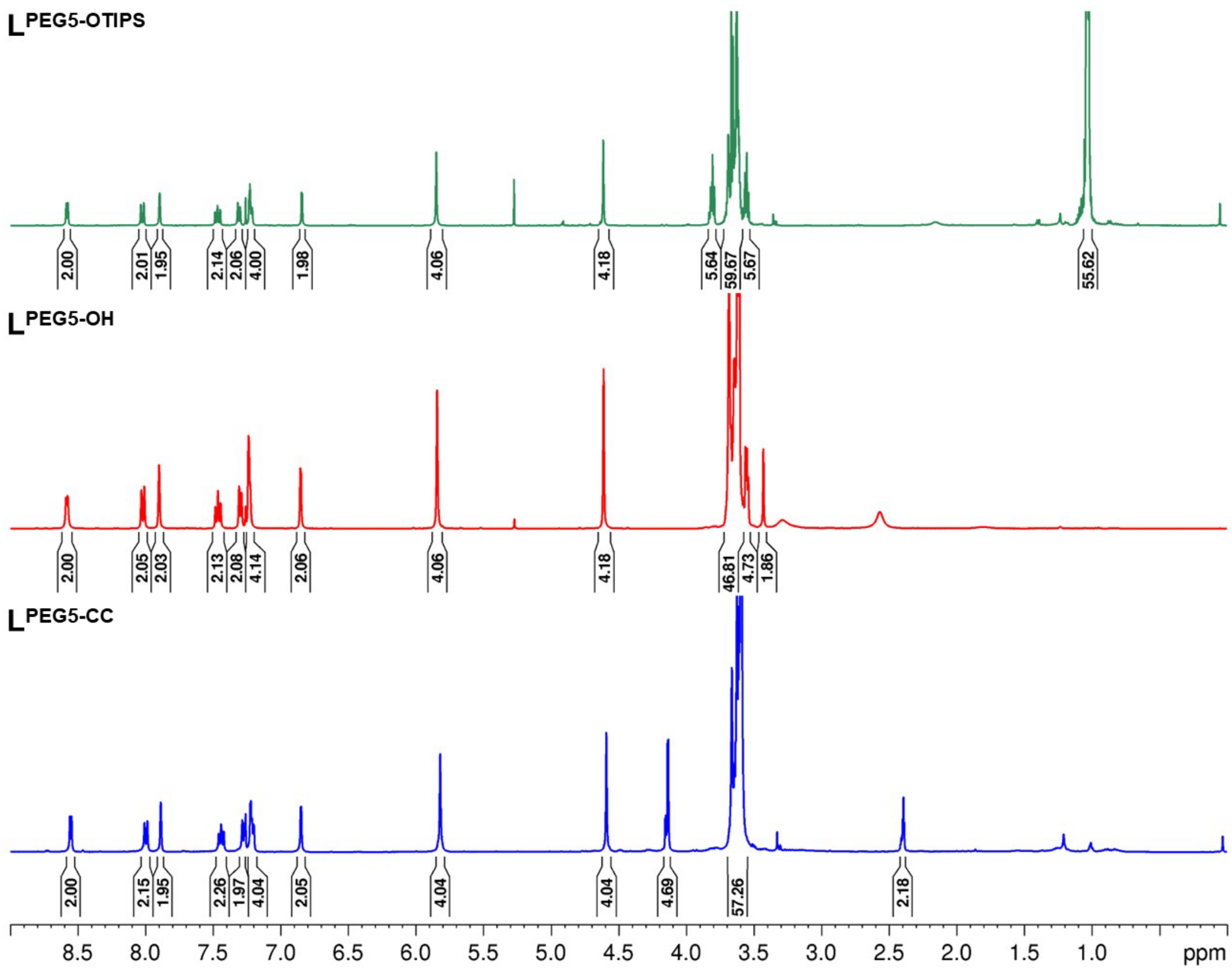


**Figure S13.** $^{1}H$ NMR spectrum (400 MHz, $CDCl_3$, 298K) of $\mathbf{L^{PEG5\text{-}OTIPS}}$, $\mathbf{L^{PEG5\text{-}OH}}$ and $\mathbf{L^{PEG5\text{-}CC}}$.

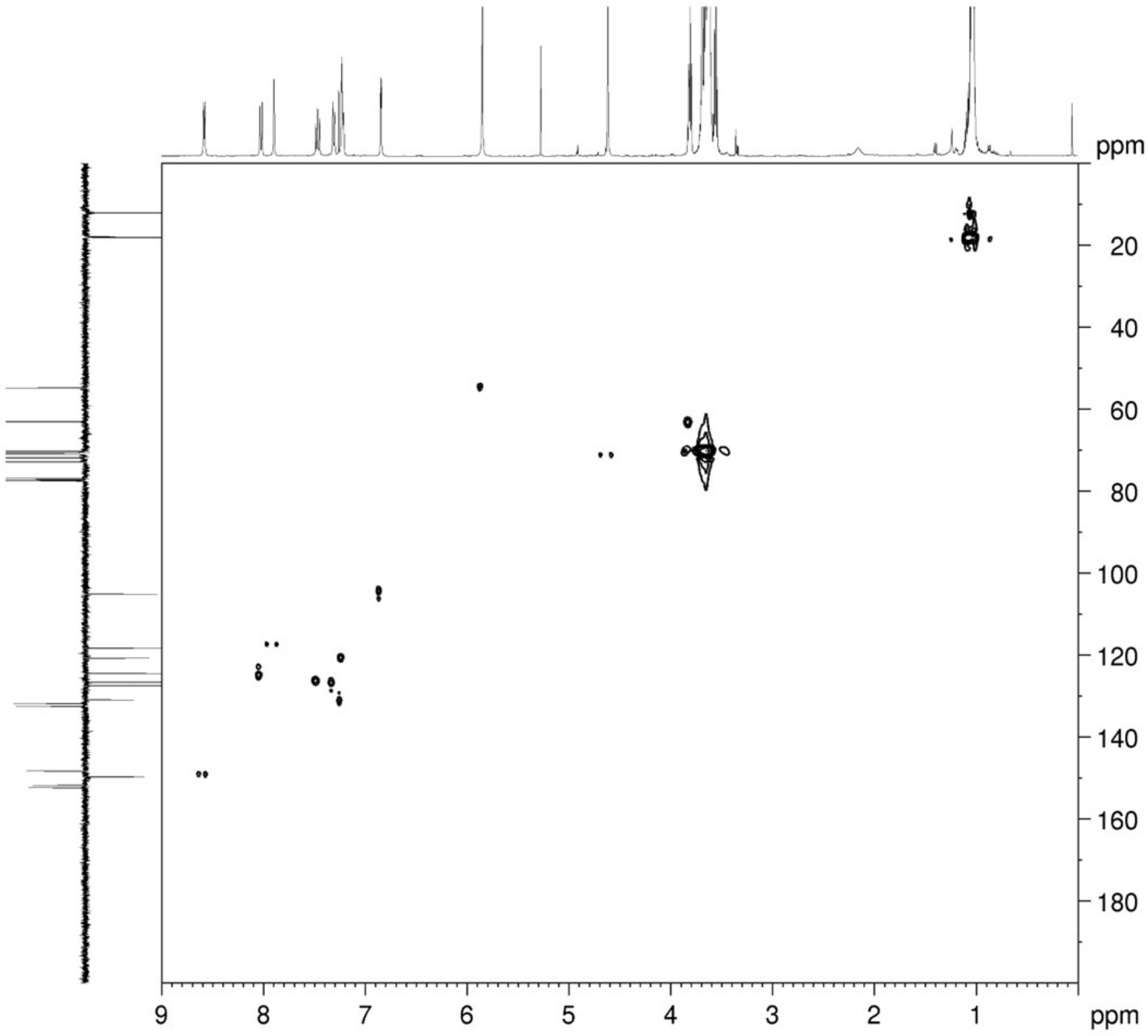


**Figure S14.** 2D $^{1}H$-$^{13}C$ HSQC NMR spectrum ($CDCl_3$, 298 K) of **L$^{PEG5\text{-}OTIPS}$**.

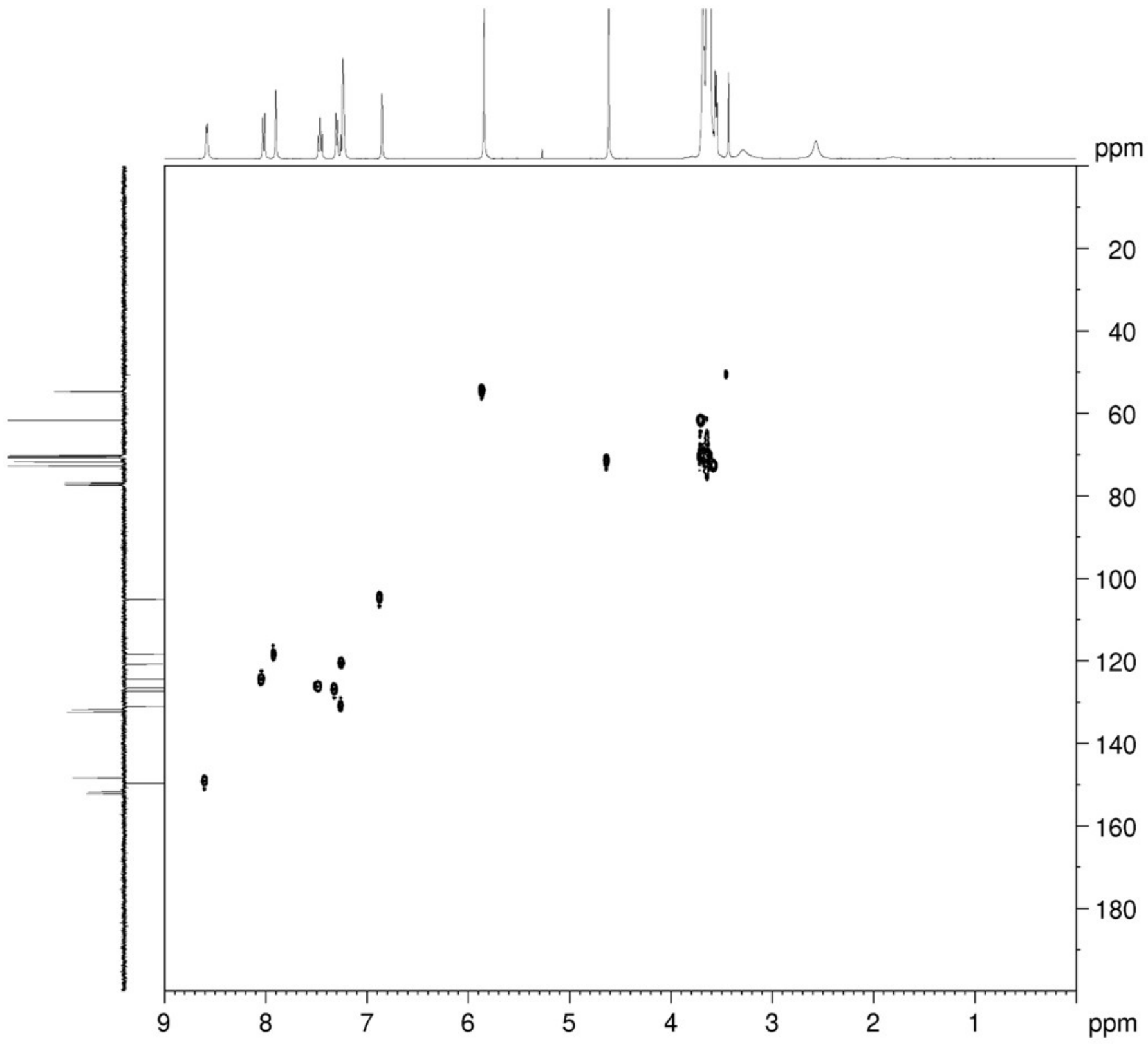


**Figure S15.** 2D $^{1}H$-$^{13}C$ HSQC NMR spectrum ($CDCl_3$, 298 K) of $\mathbf{L}^{\mathbf{PEG5\text{-}OH}}$.

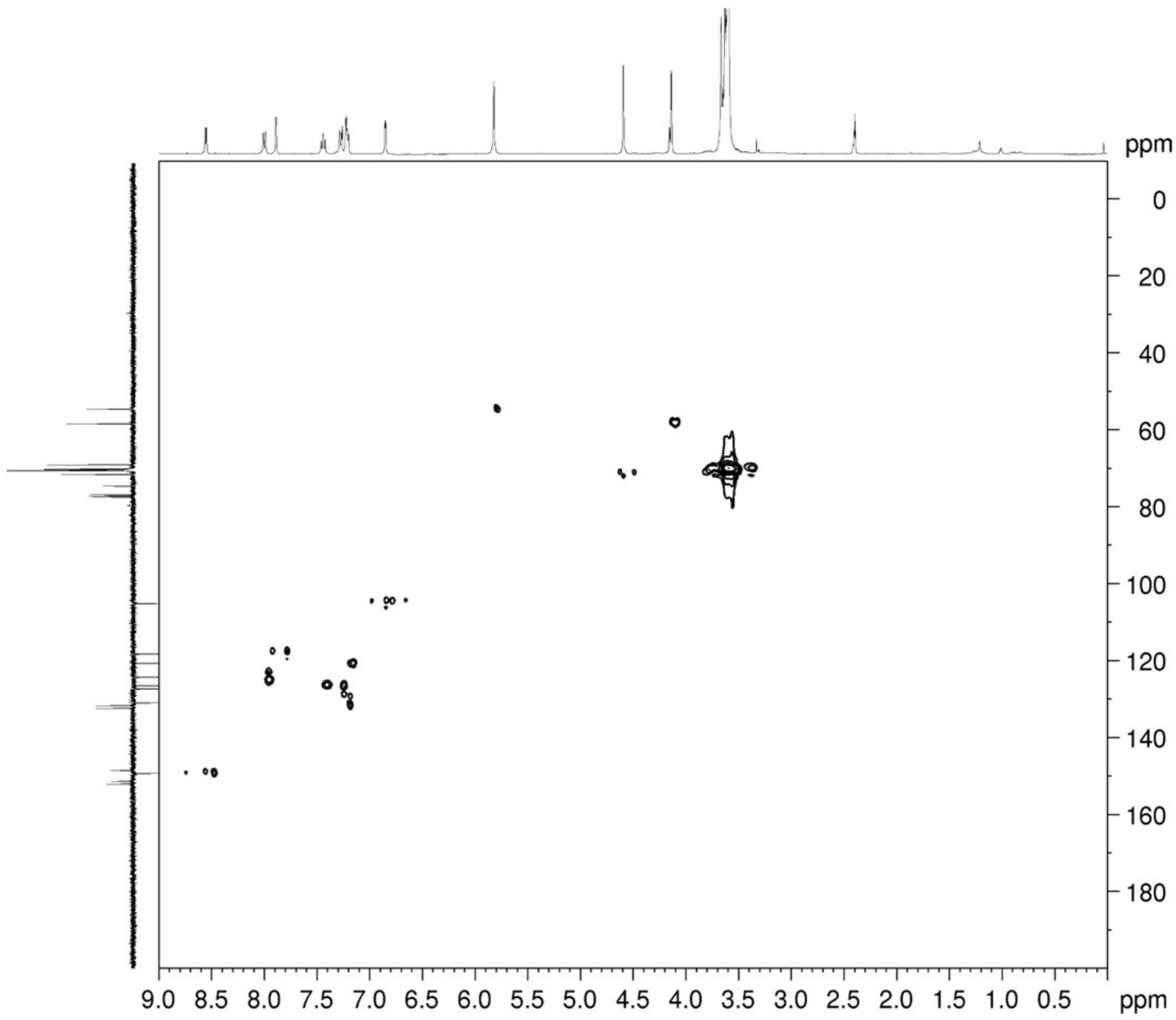


**Figure S16.** 2D $^{1}H$-$^{13}C$ HSQC NMR spectrum ($CDCl_3$, 298 K) of $\mathbf{L^{PEG5\text{-}CC}}$.

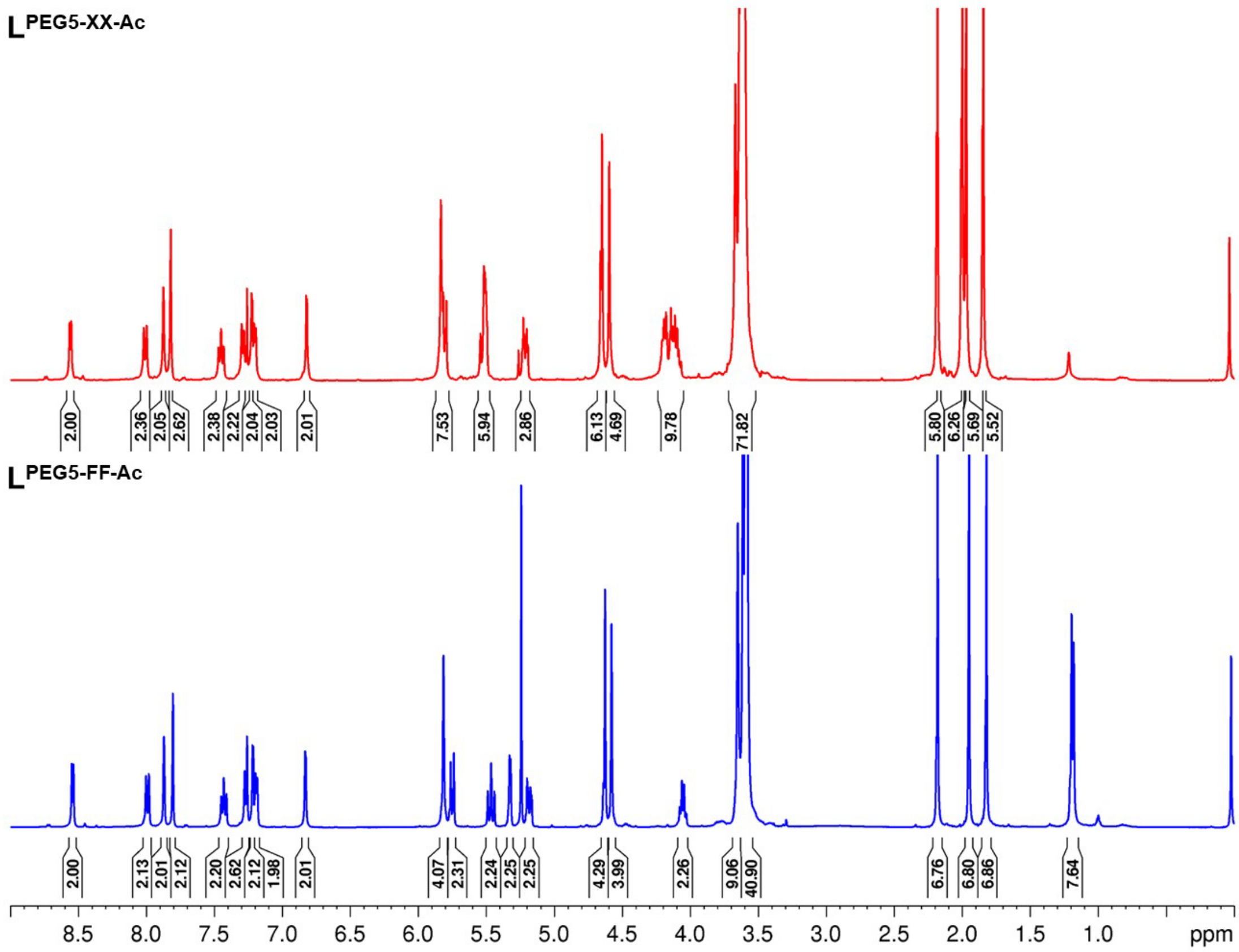


**Figure S17.** $^{1}$H NMR spectra (400 MHz, $CDCl_3$, 298K) of $\mathbf{L^{PEG5\text{-}XX\text{-}Ac}}$ and $\mathbf{L^{PEG5\text{-}FF\text{-}Ac}}$.

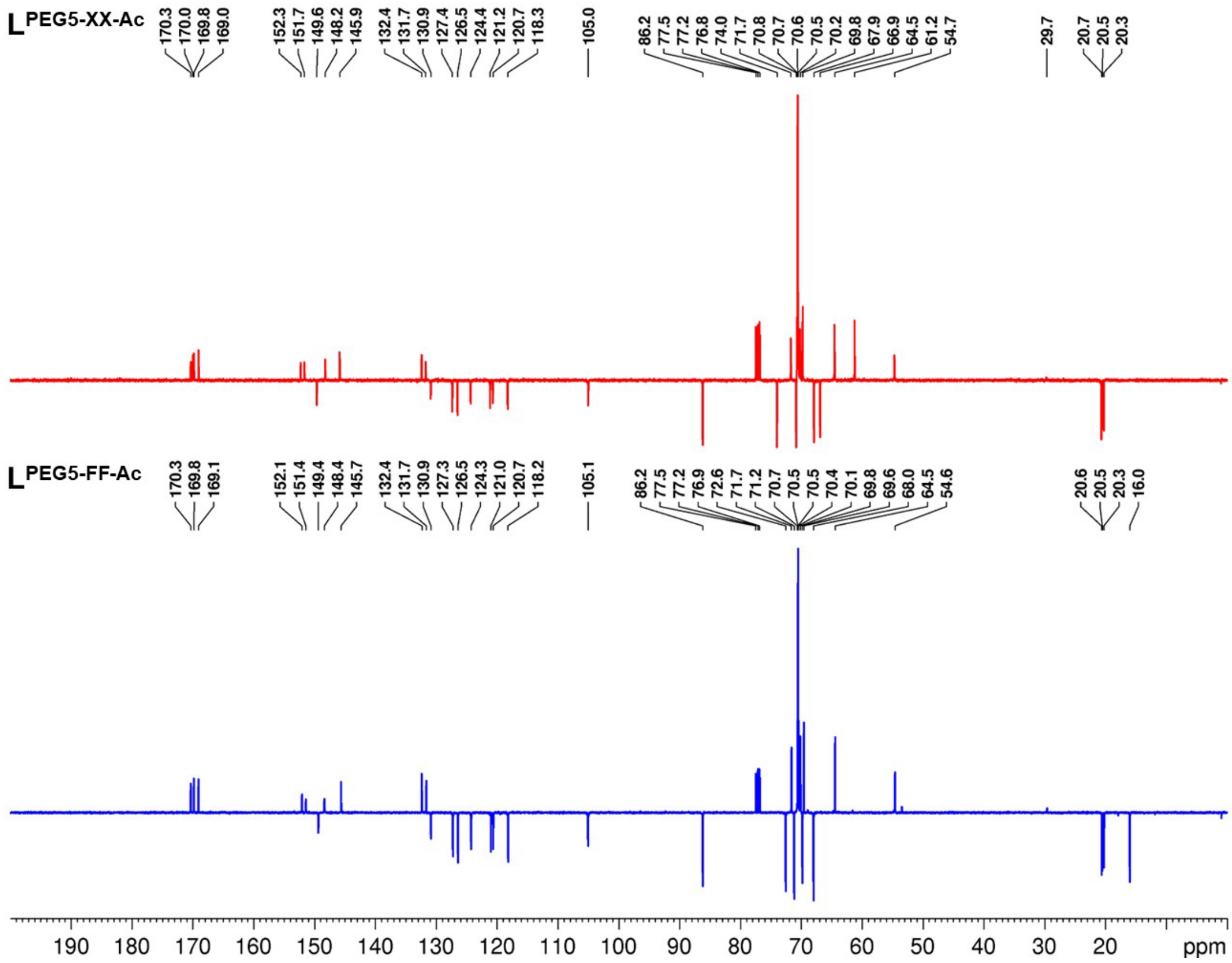


**Figure S18.** $^{13}$C NMR spectra (100 MHz, $CDCl_3$, 298K) of **L$^{PEG5-XX-Ac}$** and **L$^{PEG5-FF-Ac}$**.

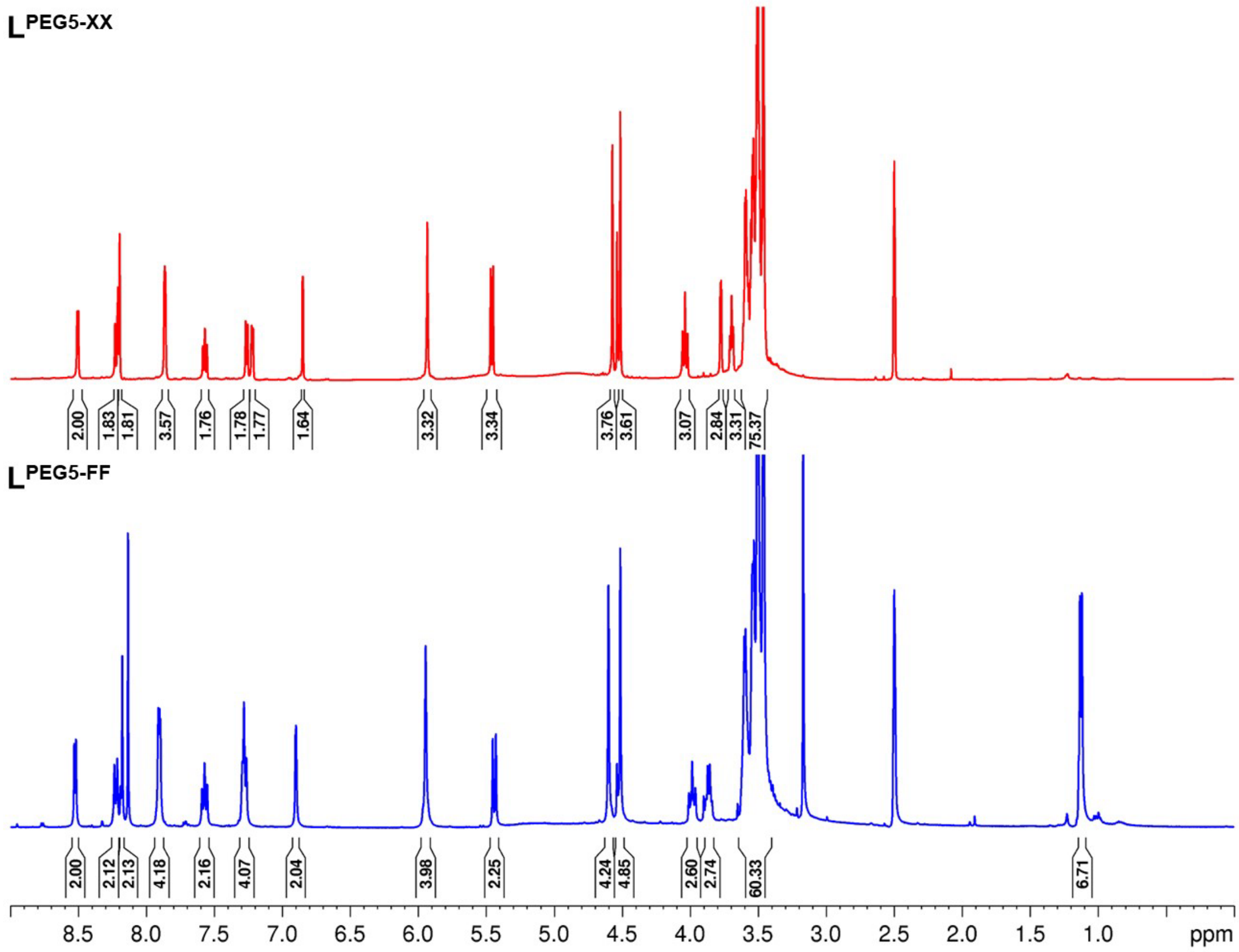


**Figure S19.** $^{1}$H NMR spectrum (500 MHz, DMSO-$d_6$, 298K) of $\mathbf{L^{PEG5\text{-}XX}}$ and $^{1}$H NMR spectrum (400 MHz, DMSO-$d_6$, 298K) of $\mathbf{L^{PEG5\text{-}FF}}$.

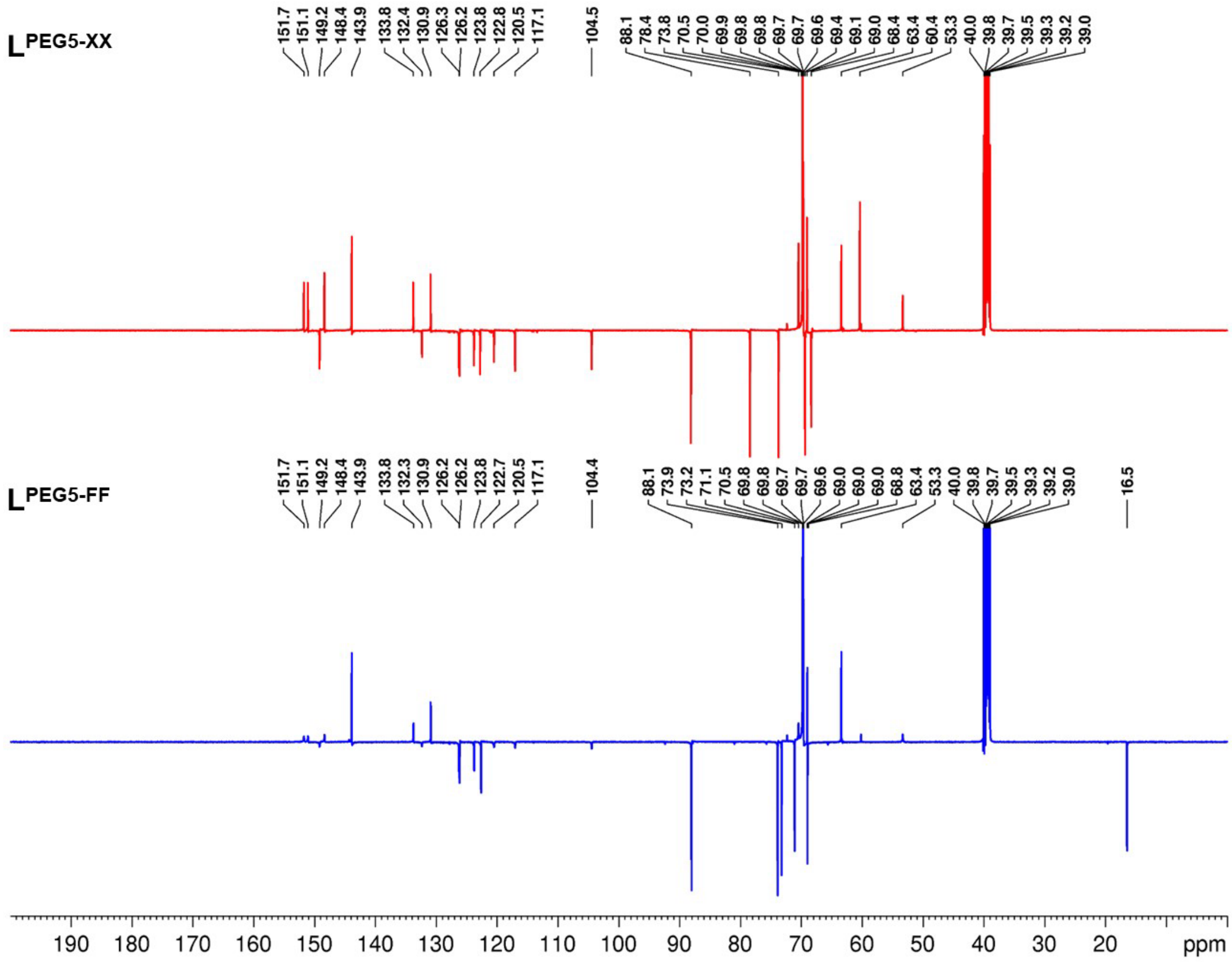


**Figure S20.** $^{13}$C NMR spectra (125 MHz, DMSO-$d_6$, 298K) of $\mathbf{L^{PEG5\text{-}XX}}$ and $\mathbf{L^{PEG5\text{-}FF}}$.

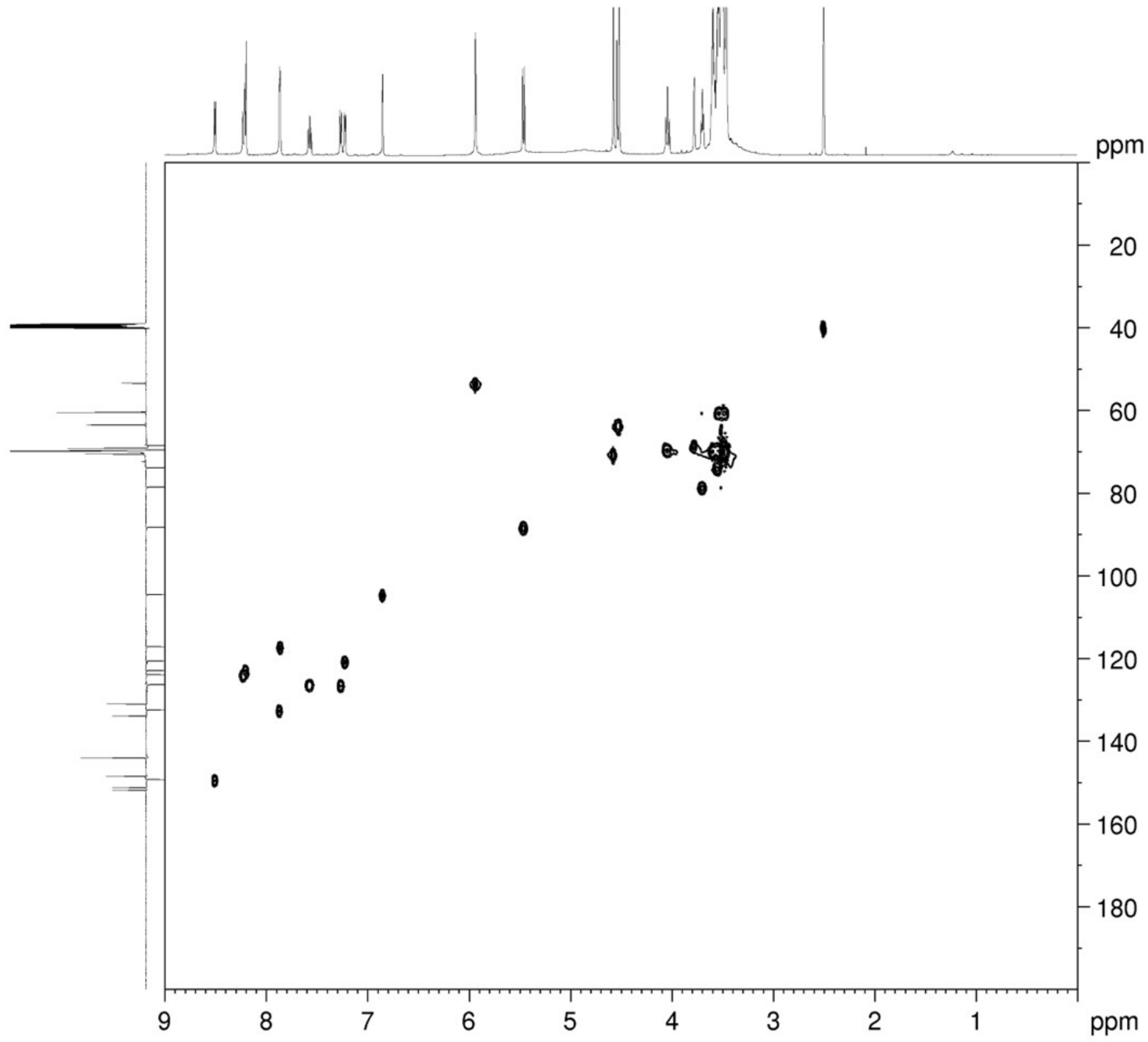


**Figure S21.** 2D $^{1}H$-$^{13}C$ HSQC NMR spectrum (DMSO-$d_6$, 298 K) of $\mathbf{L^{PEG5\text{-}XX}}$.

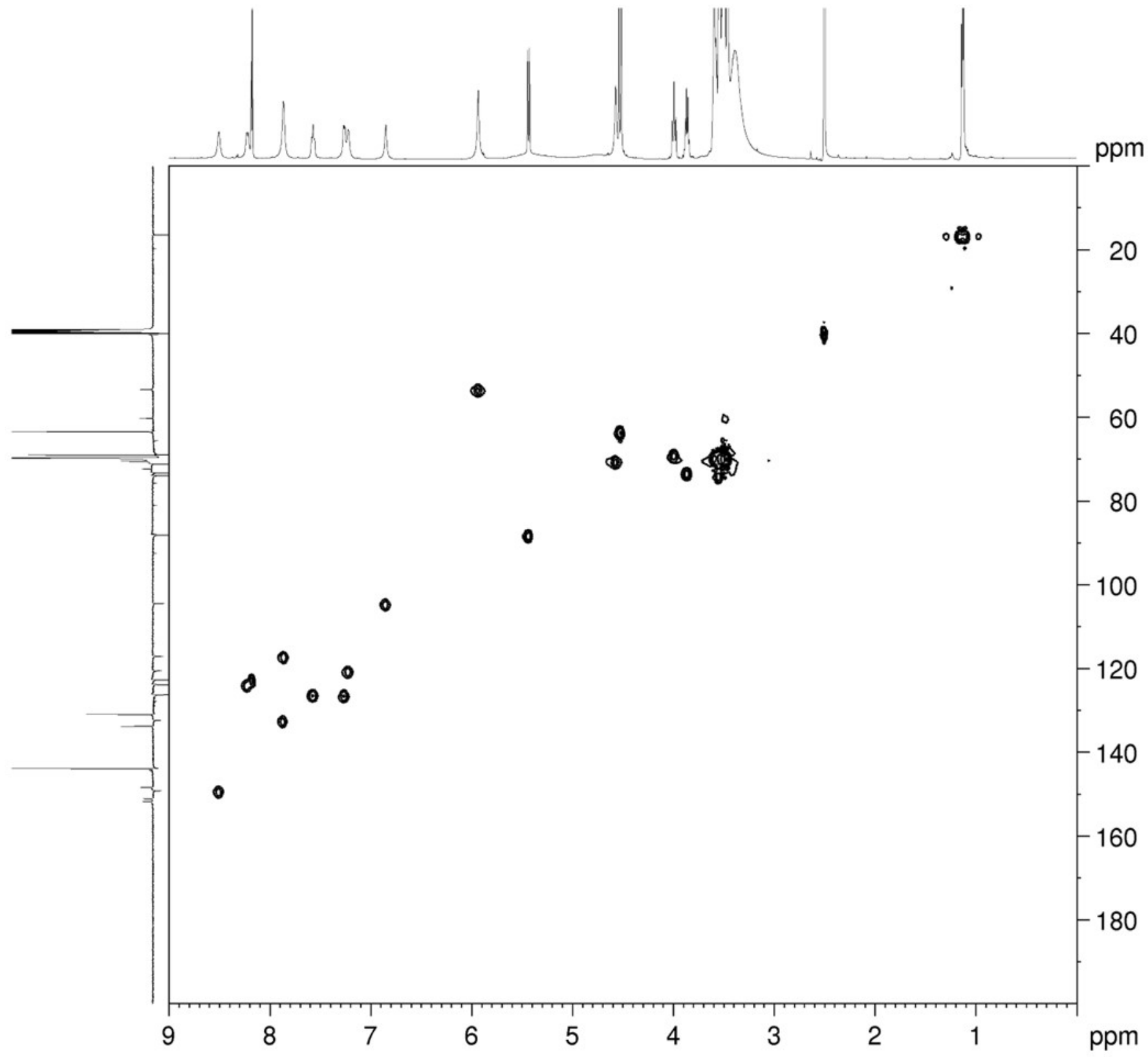


**Figure S22.** 2D $^{1}H$-$^{13}C$ HSQC NMR spectrum (DMSO-$d_6$, 298 K) of $\mathbf{L^{PEG5\text{-}FF}}$.

## 3. Selected HRMS spectra of ligands

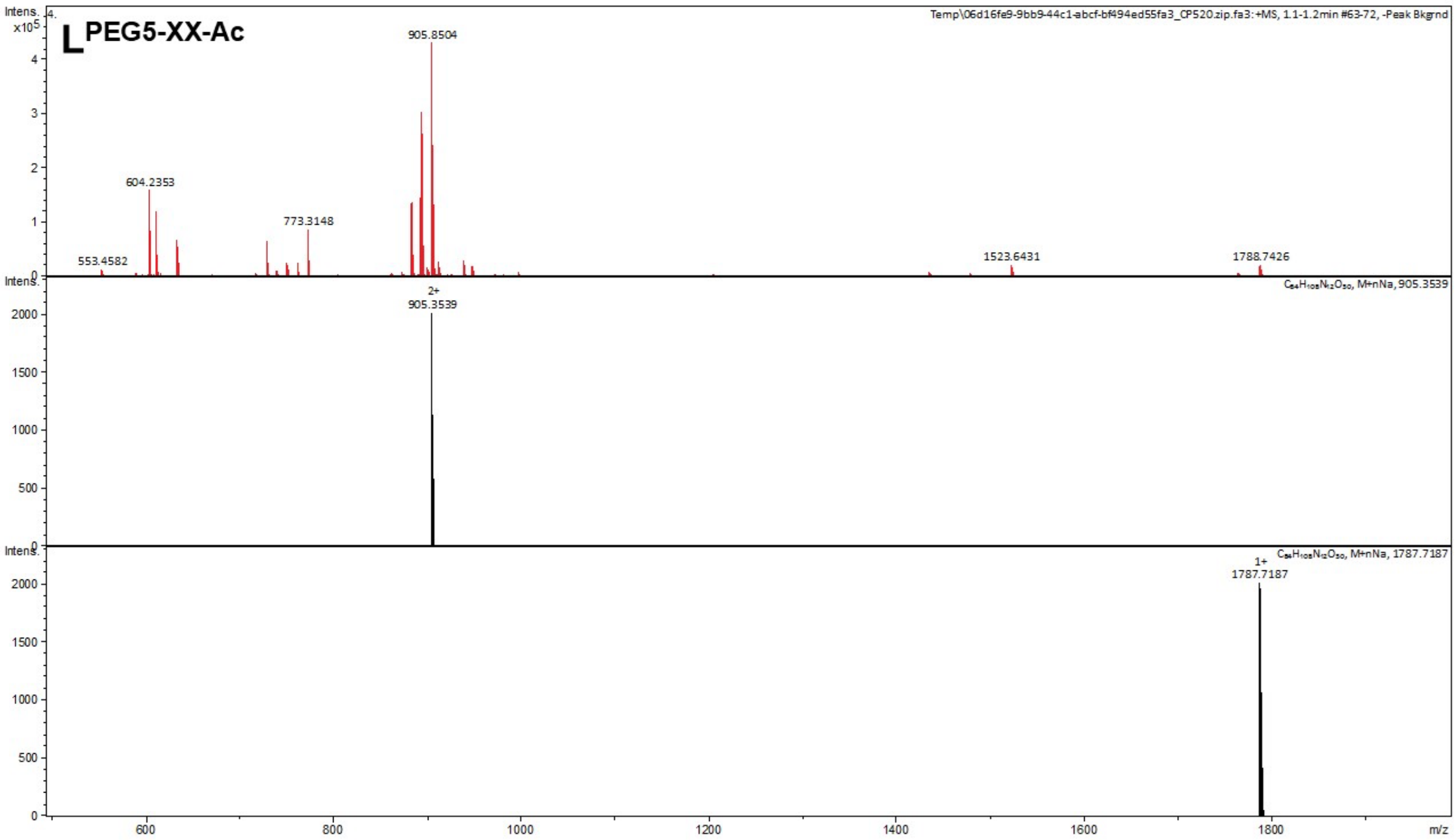


**Figure S23.** HR-ESI-MS of $\mathbf{L}^{\mathbf{PEG5\text{-}XX\text{-}Ac}}$.

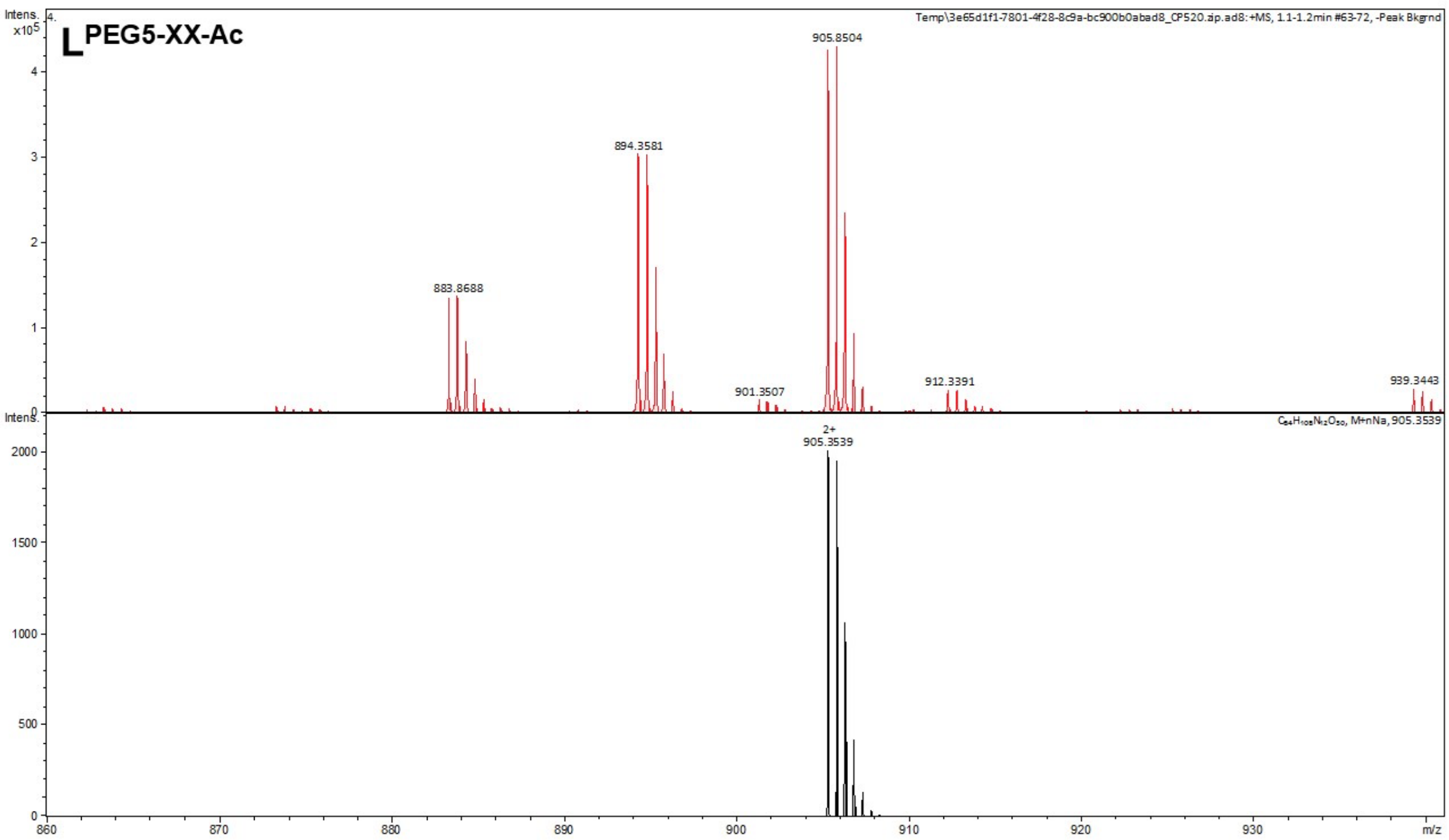


**Figure S24**: see below for caption / details

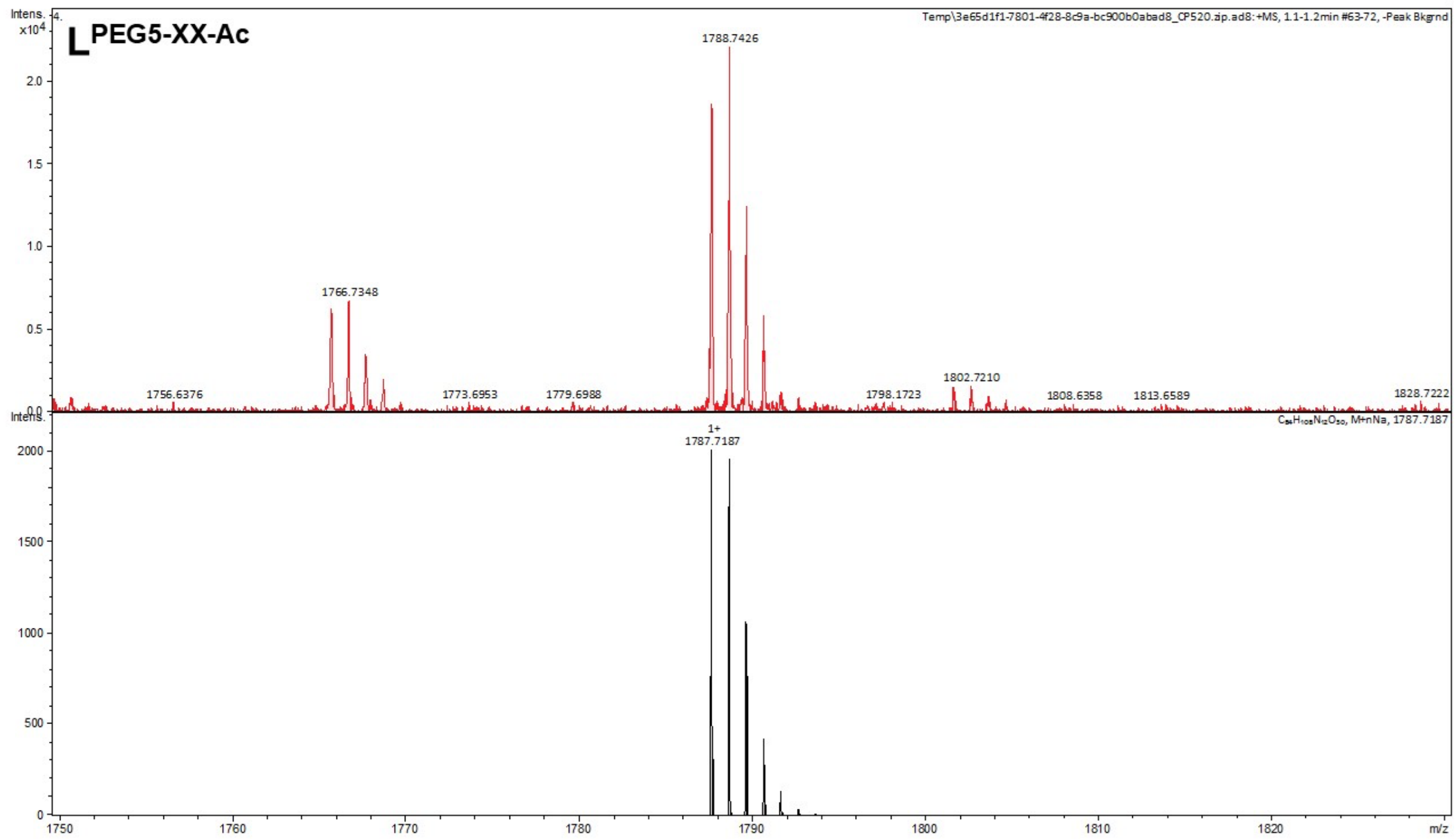


**Figure S25**: see below for caption / details

**Figures S24-25.** Selected expansions of the HR-ESI-MS spectrum for **L$^{\mathbf{PEG5\text{-}XX\text{-}Ac}}$** (main spectrum in Fig. S23) showing expansions of the signals for [$M$ + 2Na$^+$] and [$M$ + Na$^+$].

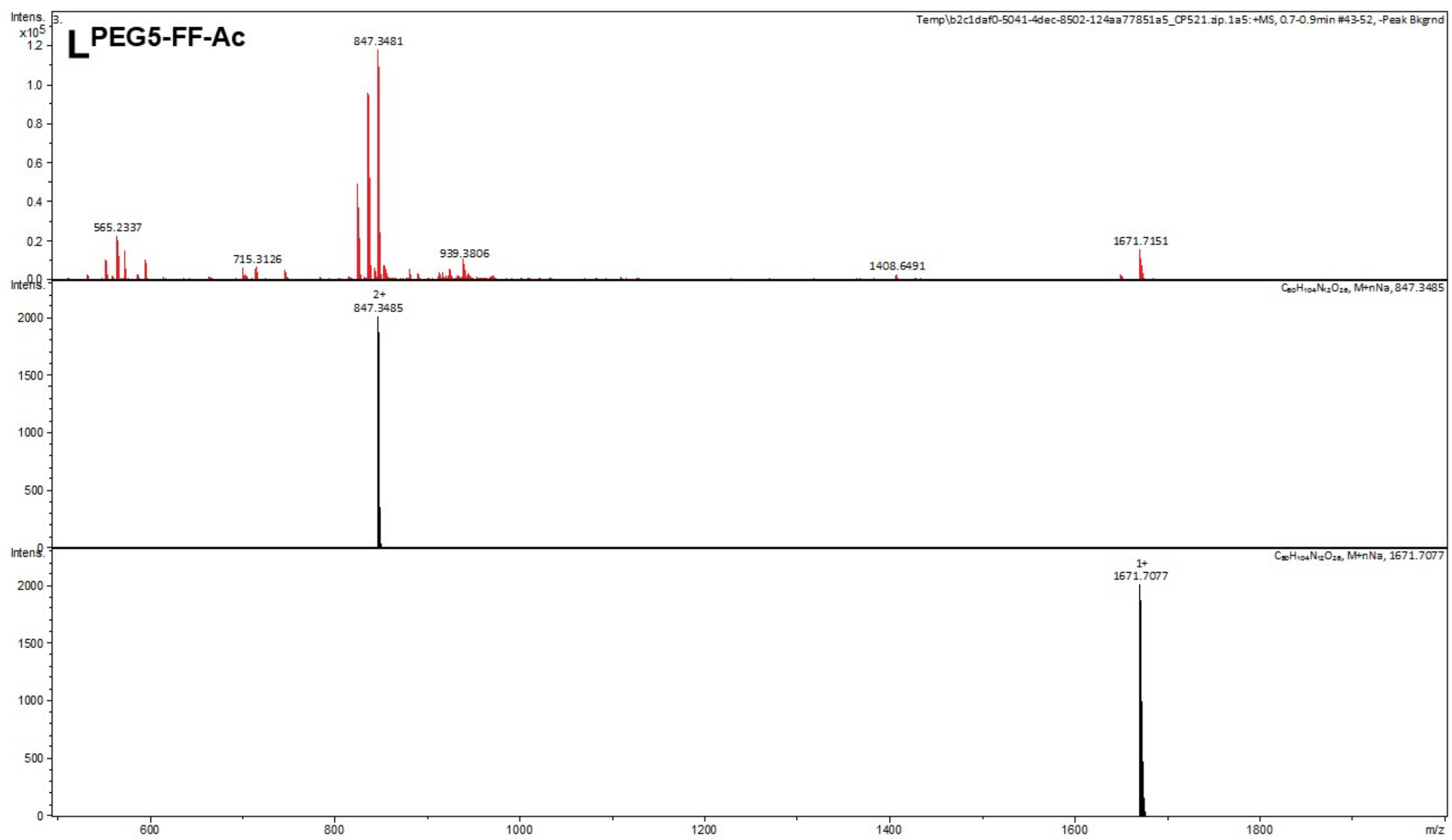


**Figure S26.** HR-ESI-MS of **L$^{\mathbf{PEG5\text{-}FF\text{-}Ac}}$**.

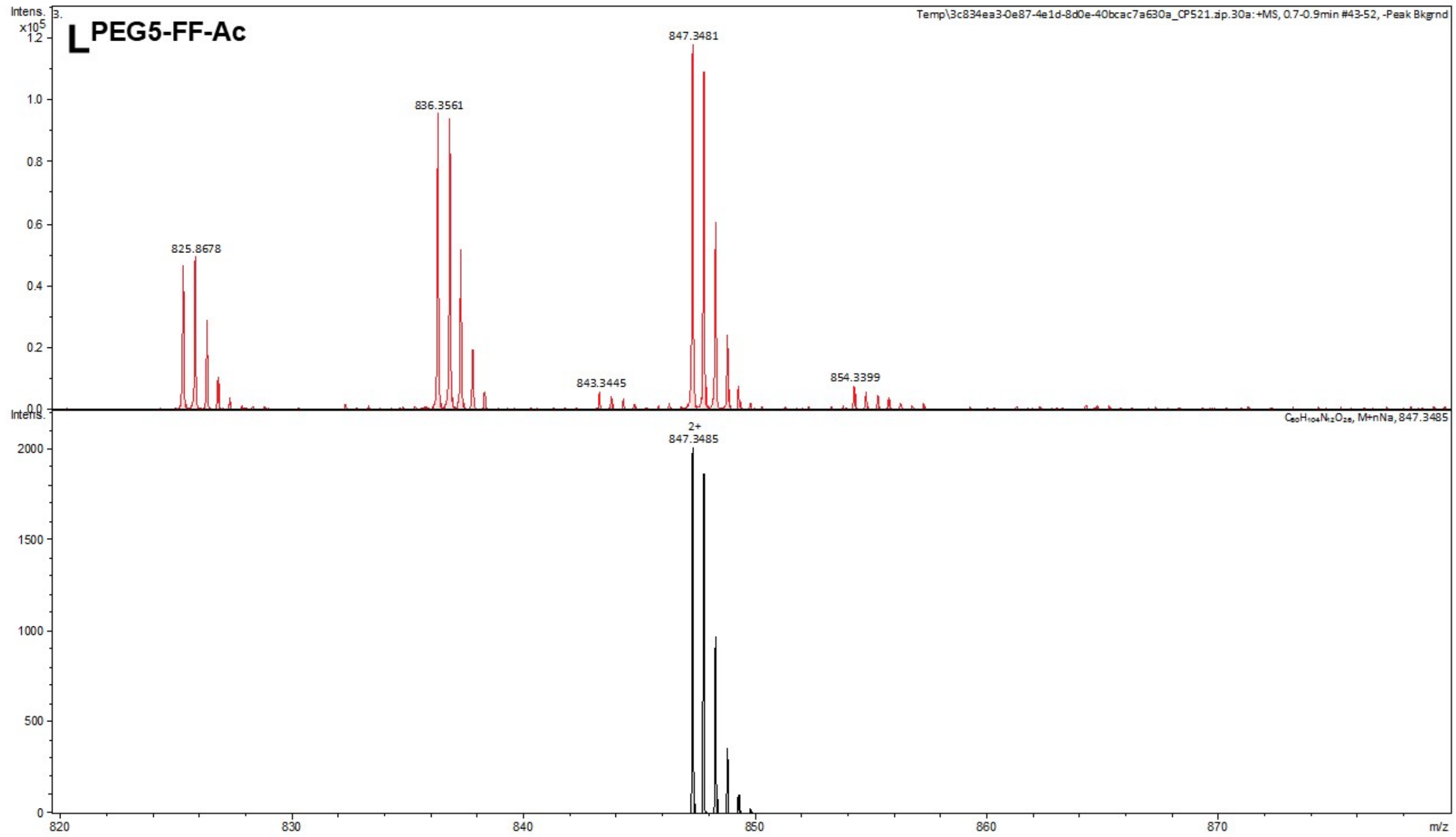


**Figure S27**: see below for caption / details

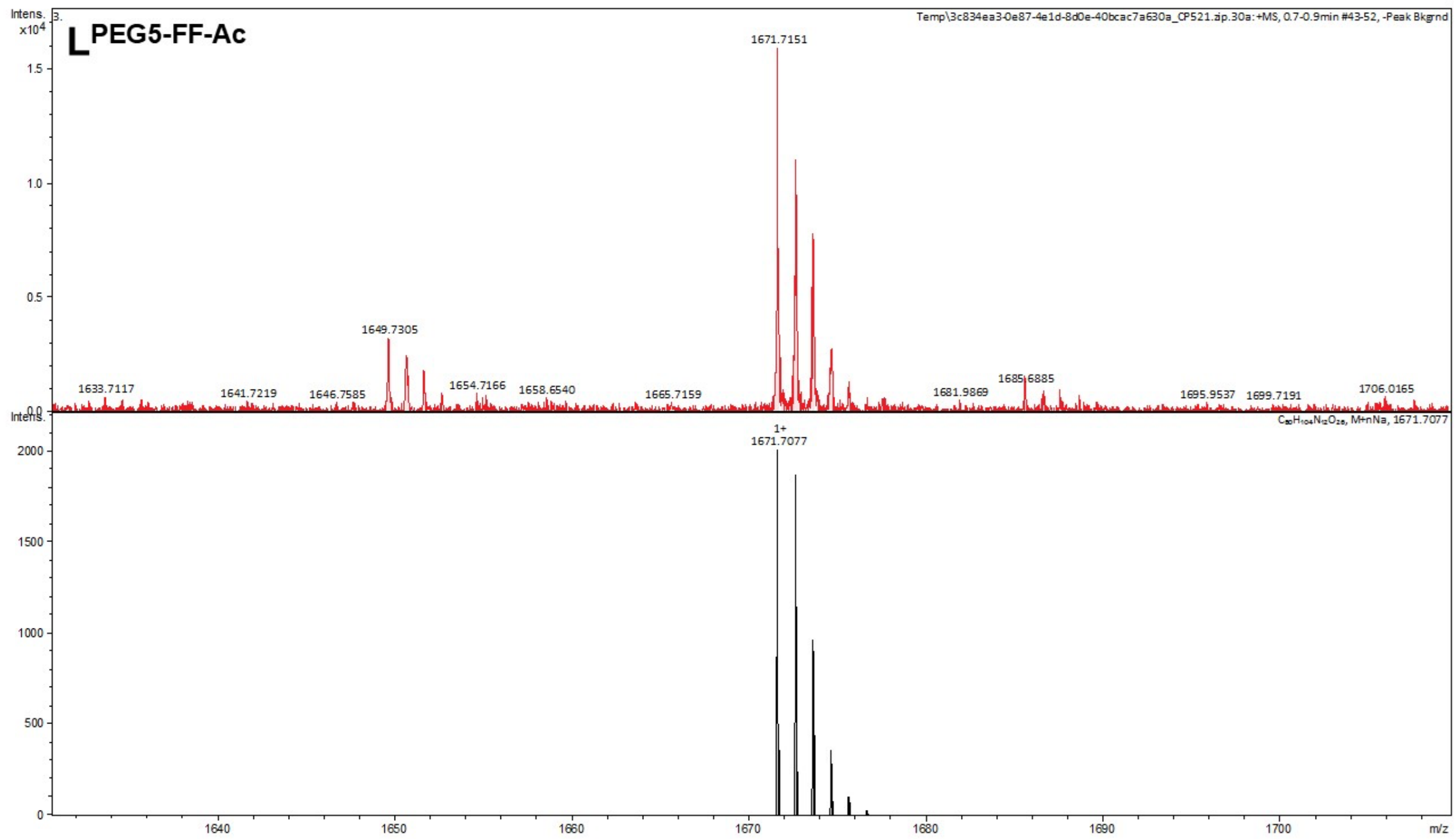


**Figure S28**: see below for caption / details

**Figures S27-28.** Selected expansions of the HR-ESI-MS spectrum for $\mathbf{L}^{\mathbf{PEG5\text{-}FF\text{-}Ac}}$ (main spectrum in Fig. S26) showing expansions of the signals for [$M$ + 2$Na^+$] and [$M$ + $Na^+$].

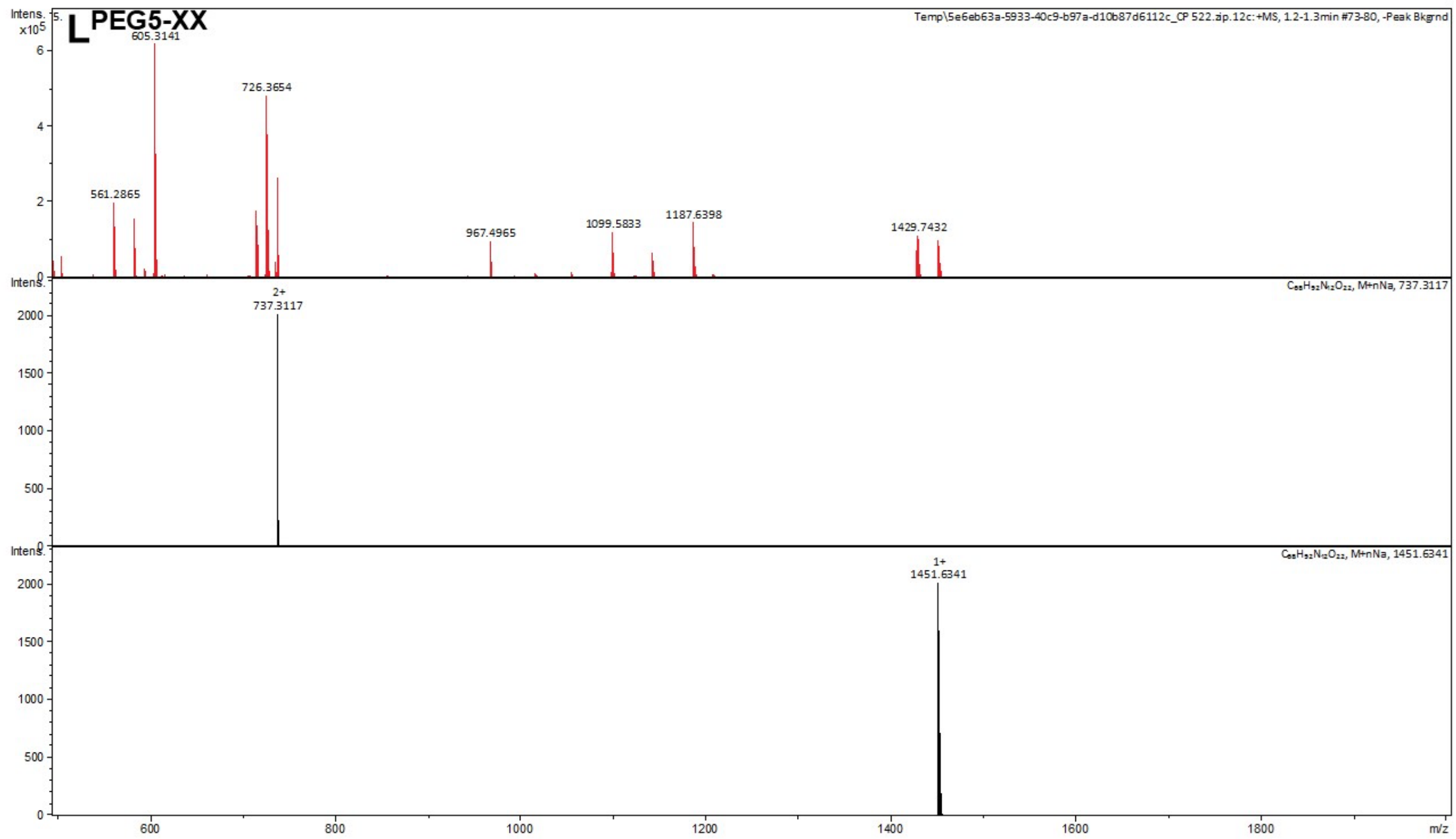


**Figure S29.** HR-ESI-MS of **L$^{\mathbf{PEG5\text{-}XX}}$**.

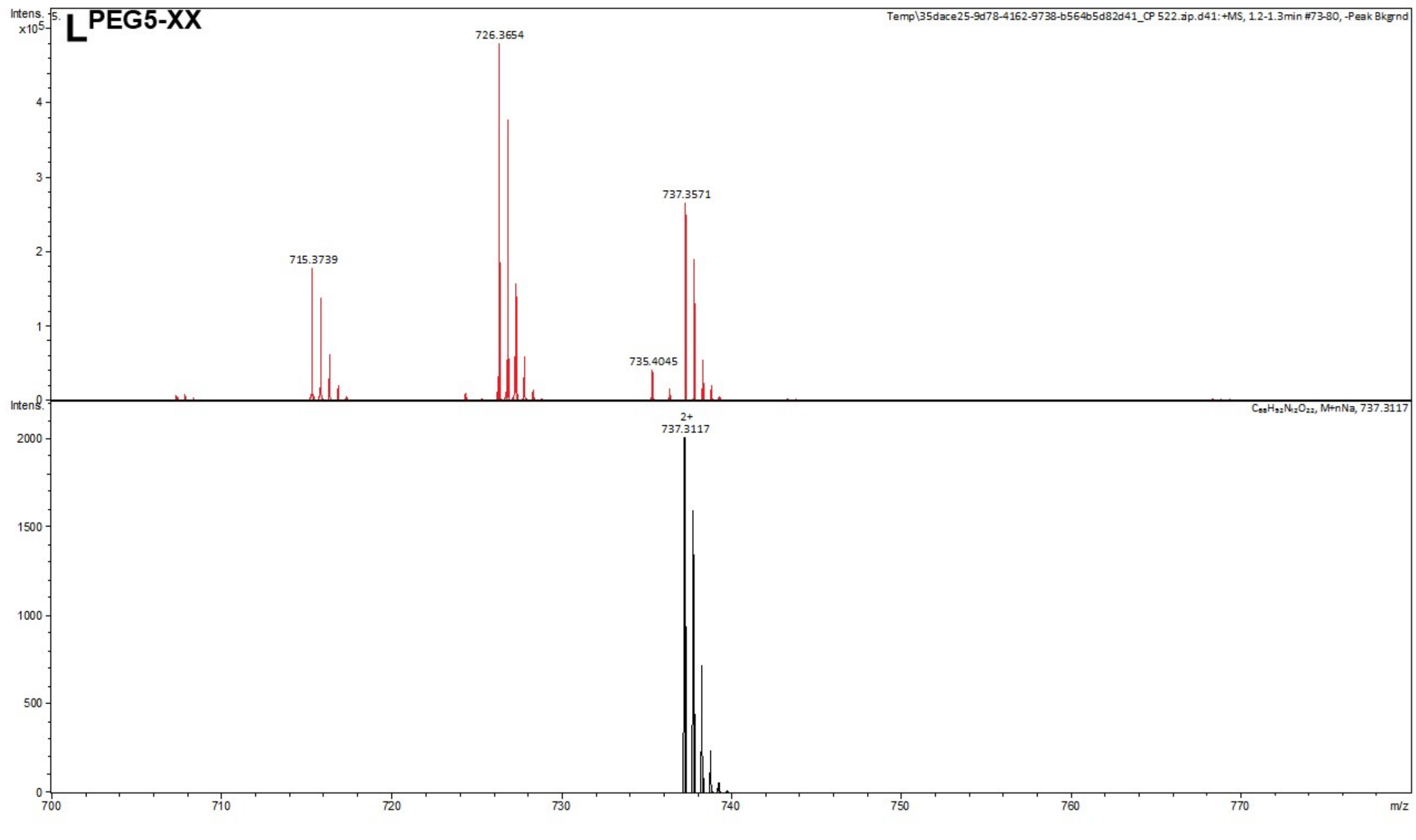


**Figure S30**: see below for caption / details

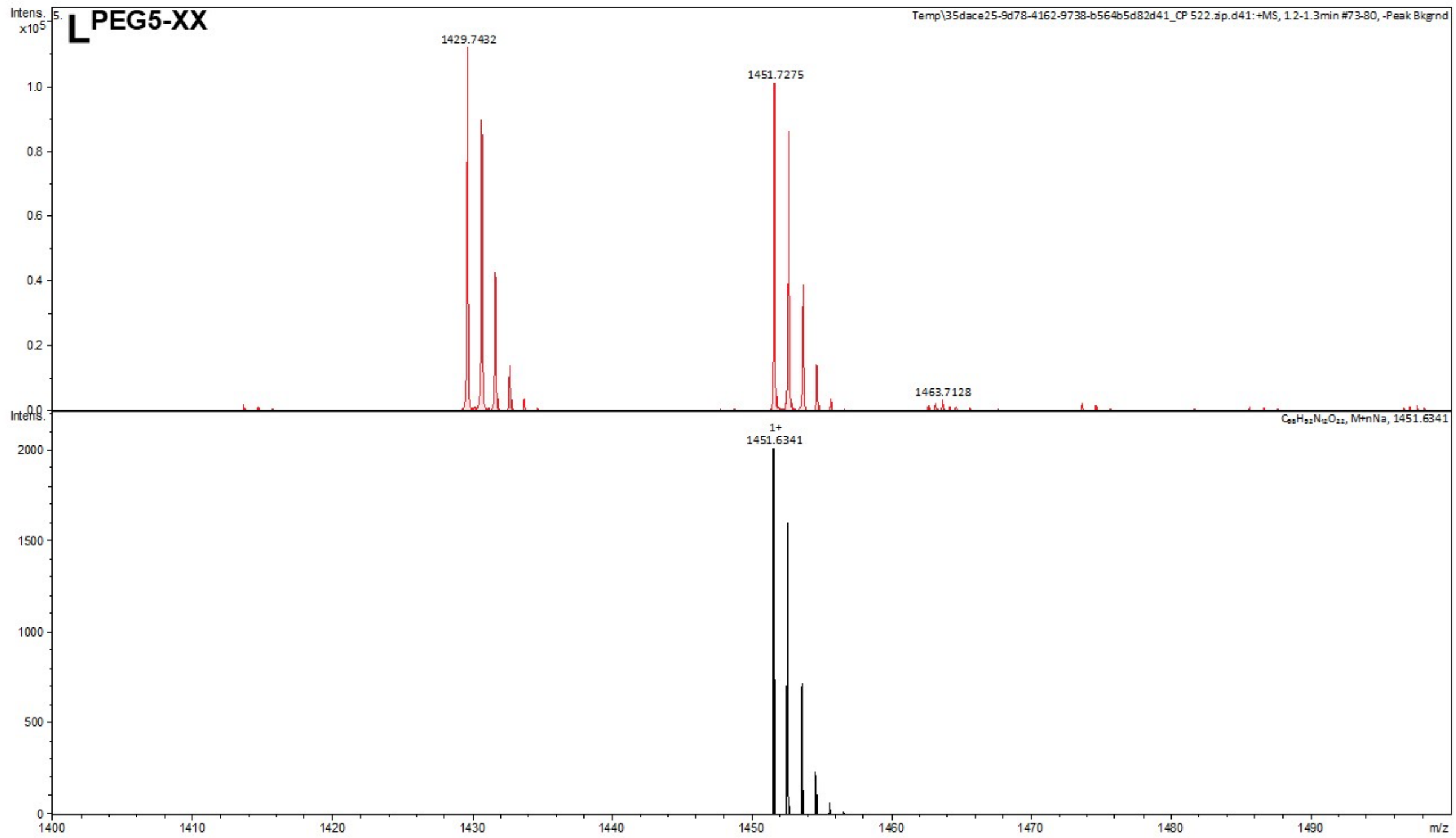


**Figure S31**: see below for caption / details

**Figures S30-31.** Selected expansions of the HR-ESI-MS spectrum for **L**$^{\mathbf{PEG5\text{-}XX}}$ (main spectrum in Fig. S29) showing expansions of the signals for [$M$ + 2Na$^+$] and [$M$ + Na$^+$].

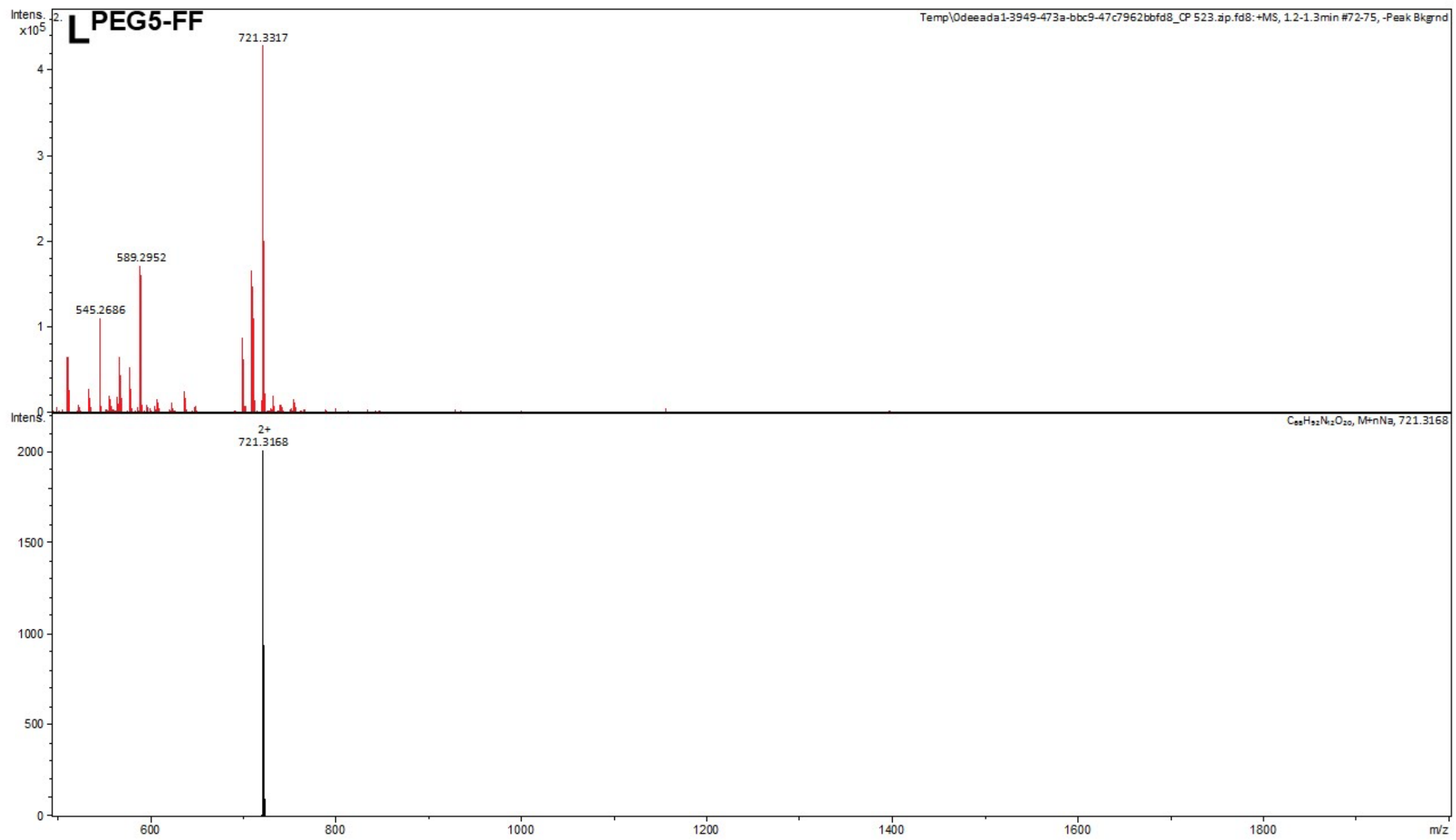


**Figure S32.** HR-ESI-MS of **L**$^{\mathbf{PEG5\text{-}FF}}$.

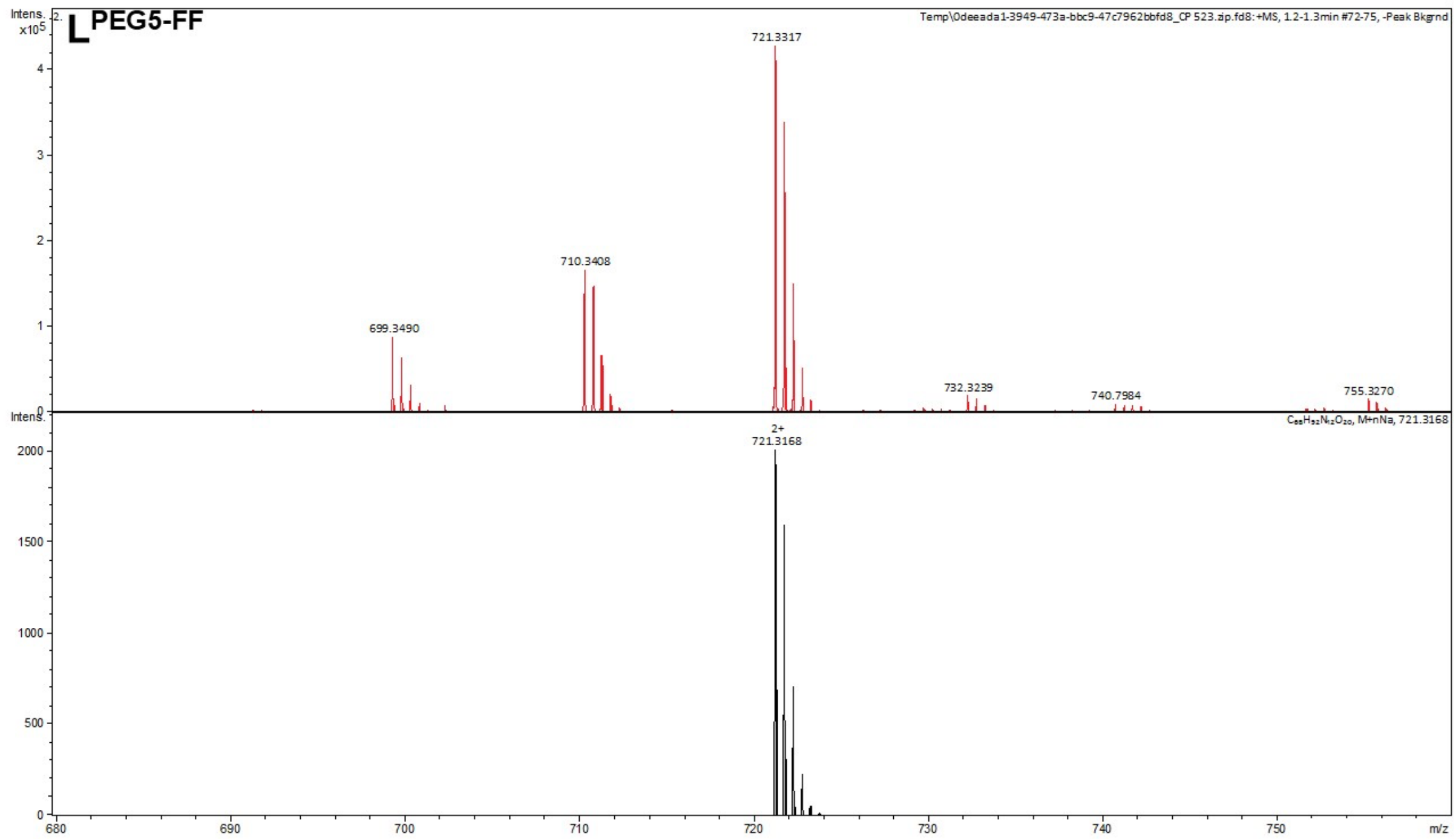


**Figures S33.** Selected expansions of the HR-ESI-MS spectrum for **L$^{PEG5-FF}$** (main spectrum in Fig. S32) showing expansion of the signal for [$M$ + $2Na^+$].

## Synthesis of $M_8L_{12}$ cubic cages

### General procedure C: Cage synthesis

To a stirred solution of the relevant glycan-functionalised ligand (1.5 equiv.) in MeOH (10 mL) was added a solution of $Co(BF_4)_2$•$xH_2O$ (1.0 equiv.) in MeOH (5 mL); a precipitate immediately formed. Water (5 mL) was then added to the mixture which was heated to 50 °C with stirring for 24 h. Once cooled to RT, the solvent was evaporated to leave a light pink solid, which was then re-dissolved in $D_2O$ (~1 mL) to confirm its identify by NMR analysis. The crude product was then immediately purified by size-exclusion chromatography on G-50 Sephadex®, using $H_2O$ as the eluent.

### [$Co_8(L^{FF})_{12}$]($BF_4$)$_{16}$

This was prepared from compound **4** (116 mg, 0.121 mmol) and $Co(BF_4)_2$•$6H_2O$ (28 mg, 81 μmol) using general procedure C. Yield: 80 mg, 59%, salmon pink solid.

**$^1$H-NMR** spectrum: see **Figure S47**

**High-resolution ES-MS:** *m/z* 1819.4131 $[\{Co_8(L^{FF})_{12}\}(BF_4)_9]^{7+}$, 1581.1243 $[\{Co_8(L^{FF})_{12}\}(BF_4)_8]^{8+}$, 1395.7844 $[\{Co_8(L^{FF})_{12}\}(BF_4)_7]^{9+}$, 1247.6064 $[\{Co_8(L^{FF})_{12}\}(BF_4)_6]^{10+}$, 1126.2839 $[\{Co_8(L^{FF})_{12}\}(BF_4)_5]^{11+}$.

**$[Co_8(L^{GX})_{12}](BF_4)_{16}$**

This was prepared from compound **9** (20 mg, 20 μmol) and $Co(BF_4)_2{\bullet}6H_2O$ (4.6 mg, 13 μmol) using general procedure C. Yield: 16 mg, 69%, green solid.
**$^1$H-NMR** spectrum: see **Figure S49**

**High-resolution ES-MS:** *m/z* 1878.7006 $[\{Co_8(L^{GX})_{12}\}(BF_4)_9]^{7+}$, 1623.1150 $[\{Co_8(L^{GX})_{12}\}(BF_4)_8]^{8+}$, 1438.3342 $[\{Co_8(L^{GX})_{12}\}(BF_4)_7]^{9+}$

**$[Co_8(L^{FX})_{12}](BF_4)_{16}$**

This was prepared from compound **10** (77 mg, 79 μmol) and $Co(BF_4)_2{\bullet}6H_2O$ (18 mg, 53 μmol) using general procedure C. Yield: 71 mg, 80%, salmon pink solid.
**$^1$H-NMR** spectrum: see **Figure S49**

**High-resolution ES-MS:** *m/z* 1846.8982 $[\{Co_8(L^{FX})_{12}\}(BF_4)_9]^{7+}$, 1605.2833 $[\{Co_8(L^{FX})_{12}\}(BF_4)_8]^{8+}$, 1417.2548 $[\{Co_8(L^{FX})_{12}\}(BF_4)_7]^{9+}$, 1266.8265 $[\{Co_8(L^{FX})_{12}\}(BF_4)_6]^{10+}$.

**$[Co_8(L^{GG})_n(L^{XX})_{12-n}](BF_4)_{16}$**

This was prepared from a mixture of **$L^{GG}$** (110 mg, 0.11 mmol) and **$L^{XX}$** (110 mg, 0.11 mmol), and $Co(BF_4)_2{\bullet}6H_2O$ (51 mg, 0.15 mmol), using general procedure C. Yield: 214 mg, 84%, salmon pink solid.
**$^1$H-NMR** spectrum: see **Figure S47**

**High-resolution ES-MS:** *m/z* 1874.1514 $[\{Co_8(L^{GG})_n(L^{XX})_{12-n}\}(BF_4)_9]^{7+}$, 1629.1333 $[\{Co_8(L^{GG})_n(L^{XX})_{12-n}\}(BF_4)_8]^{8+}$, 1438.4545 $[\{Co_8(L^{GG})_n(L^{XX})_{12-n}\}(BF_4)_7]^{9+}$, 1286.0097 $[\{Co_8(L^{GG})_n(L^{XX})_{12-n}\}(BF_4)_6]^{10+}$.

**$[Co_8(L^{FF})_n(L^{GG})_{12-n}](BF_4)_{16}$**

This was prepared from a mixture of compound **4** (53 mg, 55 μmol) and **$L^{GG}$** (54 mg, 55 μmol), and $Co(BF_4)_2{\bullet}6H_2O$ (25 mg, 74 μmol), using general procedure C. Yield: 100 mg, 80%, salmon pink solid.
**$^1$H-NMR** spectrum: see **Figure S47**

**High-resolution ES-MS:** *m/z* 1842.1834 $[\{Co_8(L^{FF})_n(L^{GG})_{12-n}\}(BF_4)_9]^{7+}$, 1601.1623 $[\{Co_8(L^{FF})_n(L^{GG})_{12-n}\}(BF_4)_8]^{8+}$, 1413.5916 $[\{Co_8(L^{FF})_n(L^{GG})_{12-n}\}(BF_4)_7]^{9+}$.

**$[Co_8(L^{FF})_n(L^{XX})_{12-n}](BF_4)_{16}$**

This was prepared from a mixture of compound **4** (53 mg, 55 μmol) and **$L^{XX}$** (54 mg, 55 μmol), and $Co(BF_4)_2•6H_2O$ (25 mg, 74 μmol), using general procedure C. Yield: 88 mg, 70%, salmon pink solid.
**$^1$H-NMR** spectrum: see **Figure S47**

**High-resolution ES-MS:** *m/z* 1821.4022 $[\{Co_8(L^{FF})_n(L^{XX})_{12-n}\}(BF_4)_9]^{7+}$, 1579.7408 $[\{Co_8(L^{FF})_n(L^{XX})_{12-n}\}(BF_4)_8]^{8+}$, 1399.3329 $[\{Co_8(L^{FF})_n(L^{XX})_{12-n}\}(BF_4)_7]^{9+}$, 1260.4022 $[\{Co_8(L^{FF})_n(L^{GG})_{12-n}\}(BF_4)_6]^{10+}$.

**$[Co_8(L^{PEG5\text{-}XX})_{12}](BF_4)_{16}$**

This was prepared from compound **15** (81 mg, 57 μmol) and $Co(BF_4)_2•6H_2O$ (13 mg, 38 μmol), using general procedure C. Yield: 62 mg, 68%, salmon pink solid.
**$^1$H-NMR** spectrum: see **Figure S51**

**High-resolution ES-MS:** Not observed.

**$[Co_8(L^{PEG5\text{-}FF})_{12}](BF_4)_{16}$**

This was prepared from compound **17** (85 mg, 61 μmol) and $Co(BF_4)_2•6H_2O$ (14 mg, 41 μmol), using general procedure C. Yield: 68 mg, 72%, salmon pink solid.
**$^1$H-NMR** spectrum: see **Figure S51**

**High-resolution ES-MS:** Not observed.

## 4. Selected HRMS and NMR spectra of cubic cages

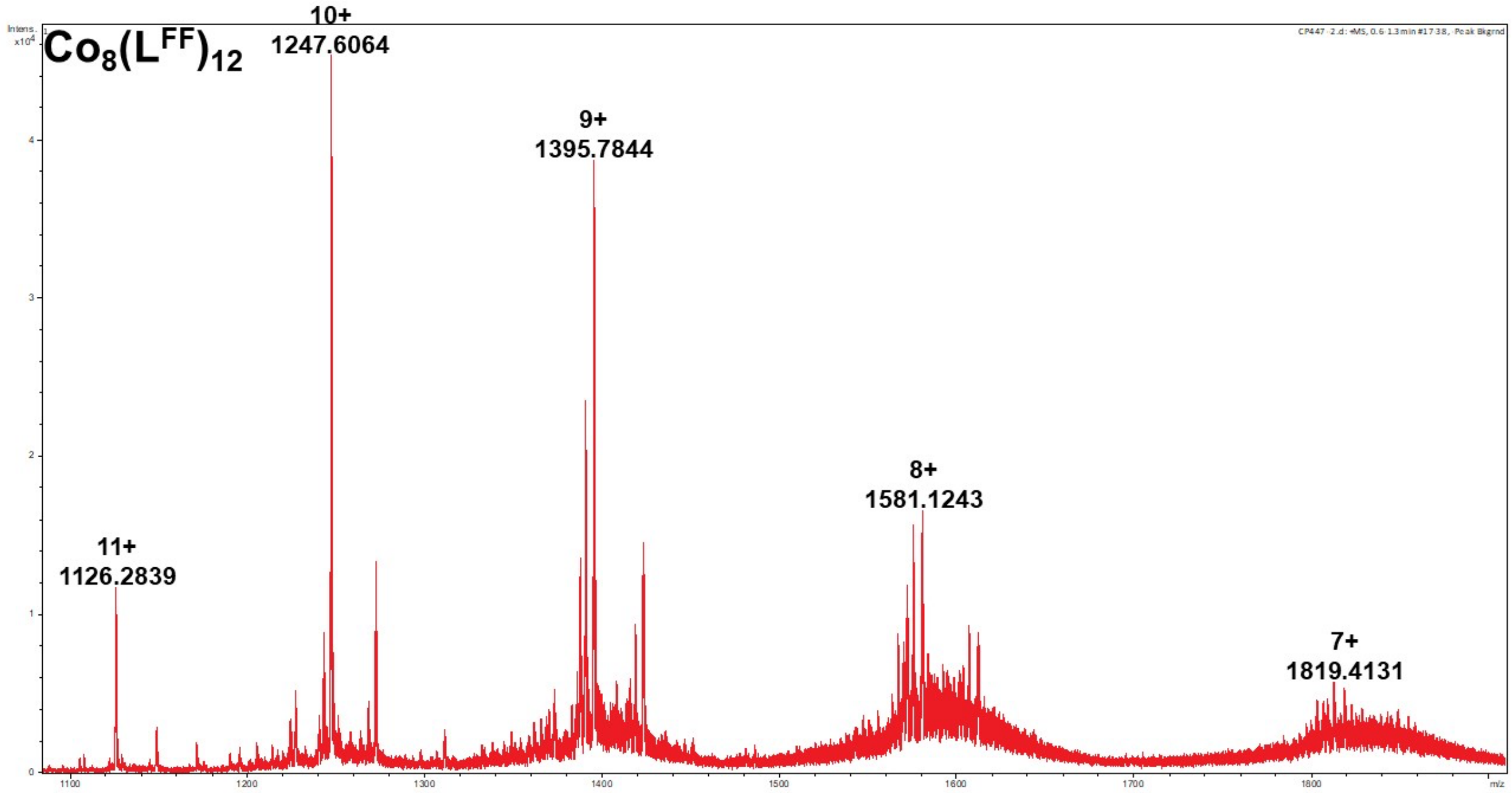


**Figure S34.** HR-ESI-MS of $[Co_8(L^{FF})_{12}](BF_4)_{16}$ in $H_2O$ (250 μM) where n = number of $BF_4$ anions lost and hence the charge in the sequence $[\{Co_8(L^{FF})_{12}\}(BF_4)_{16-n}]^{n+}$.

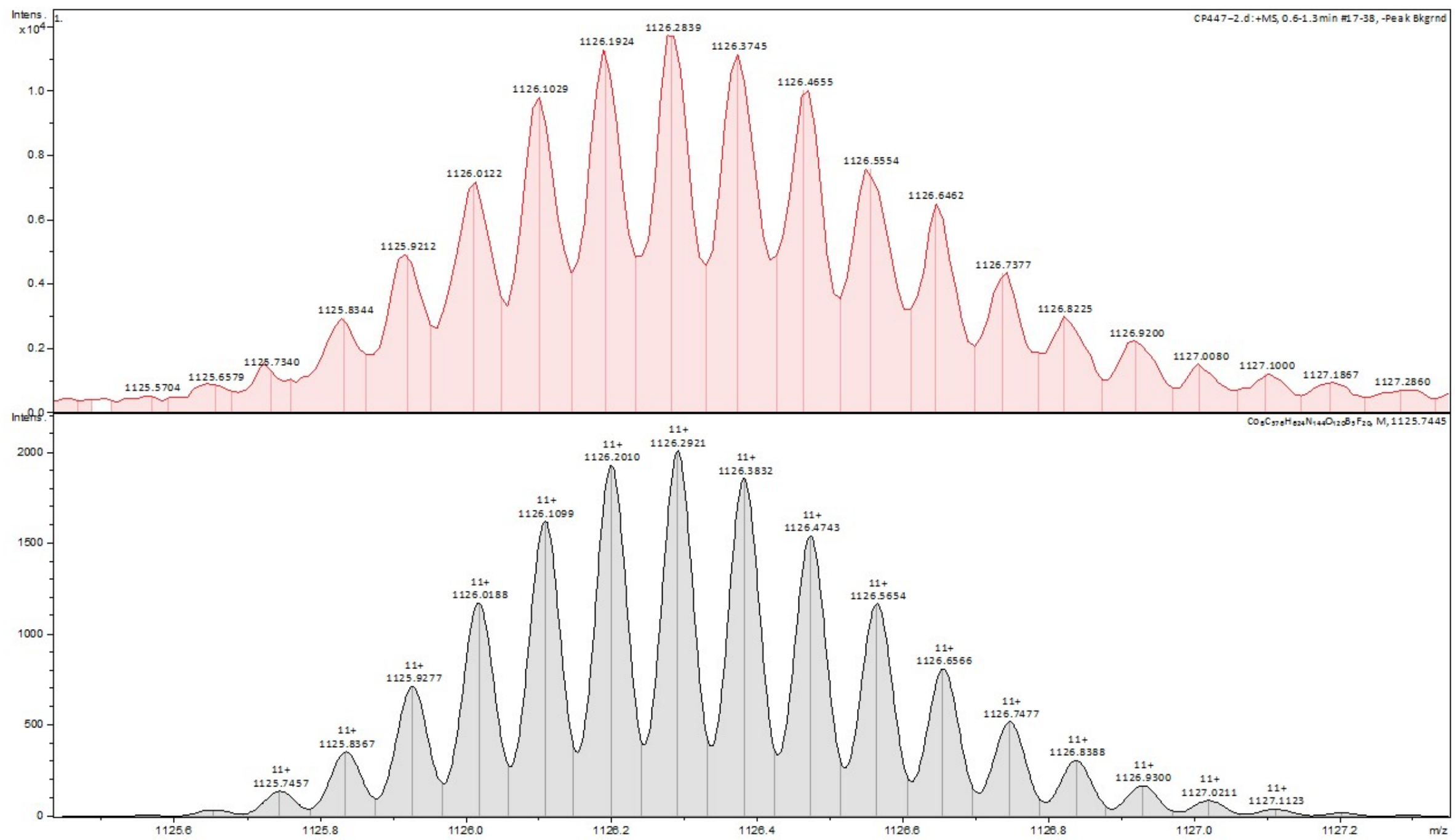


**Figure S35**: see below for caption / details

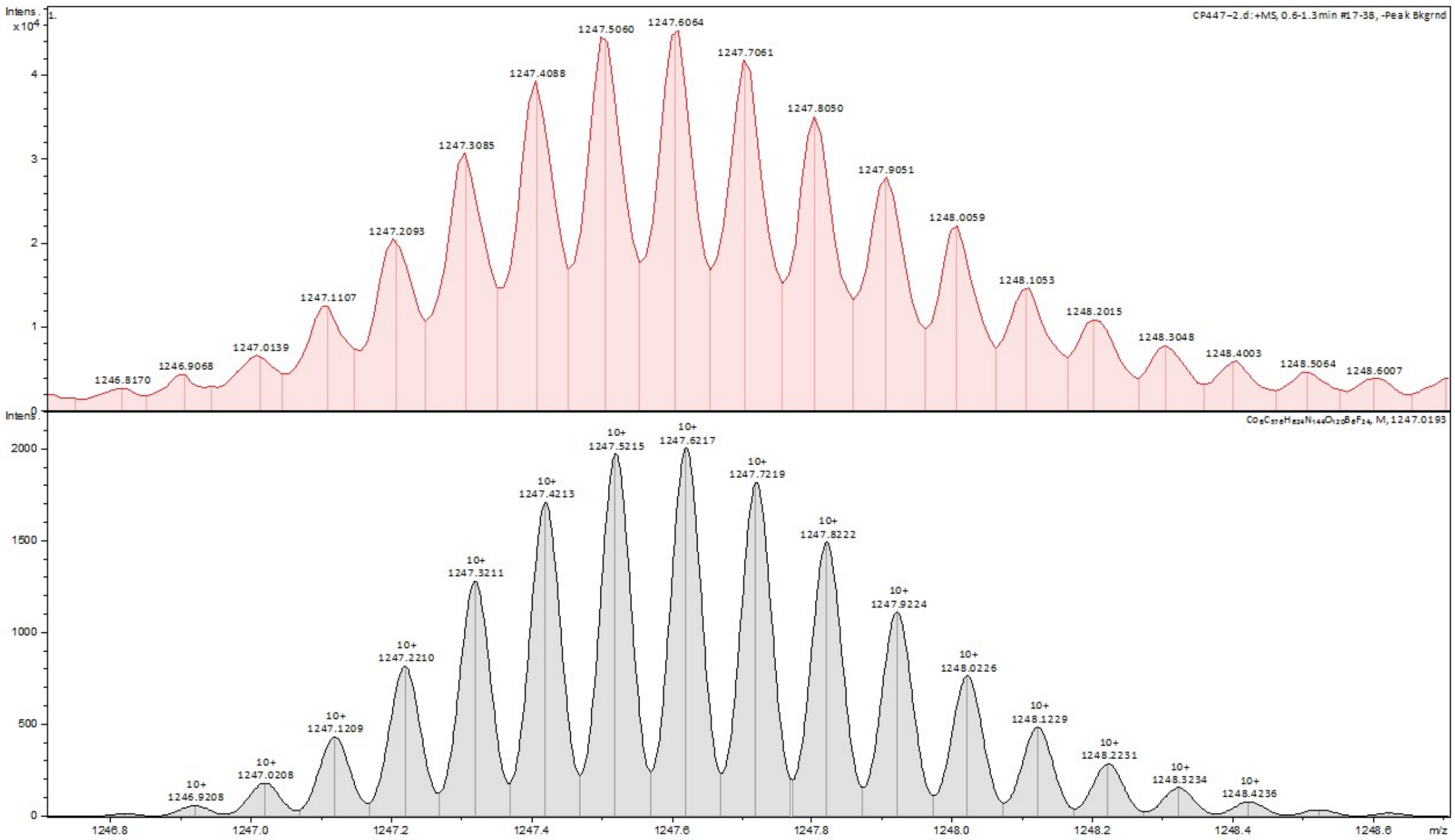

**Figure S36:** see below for caption / details

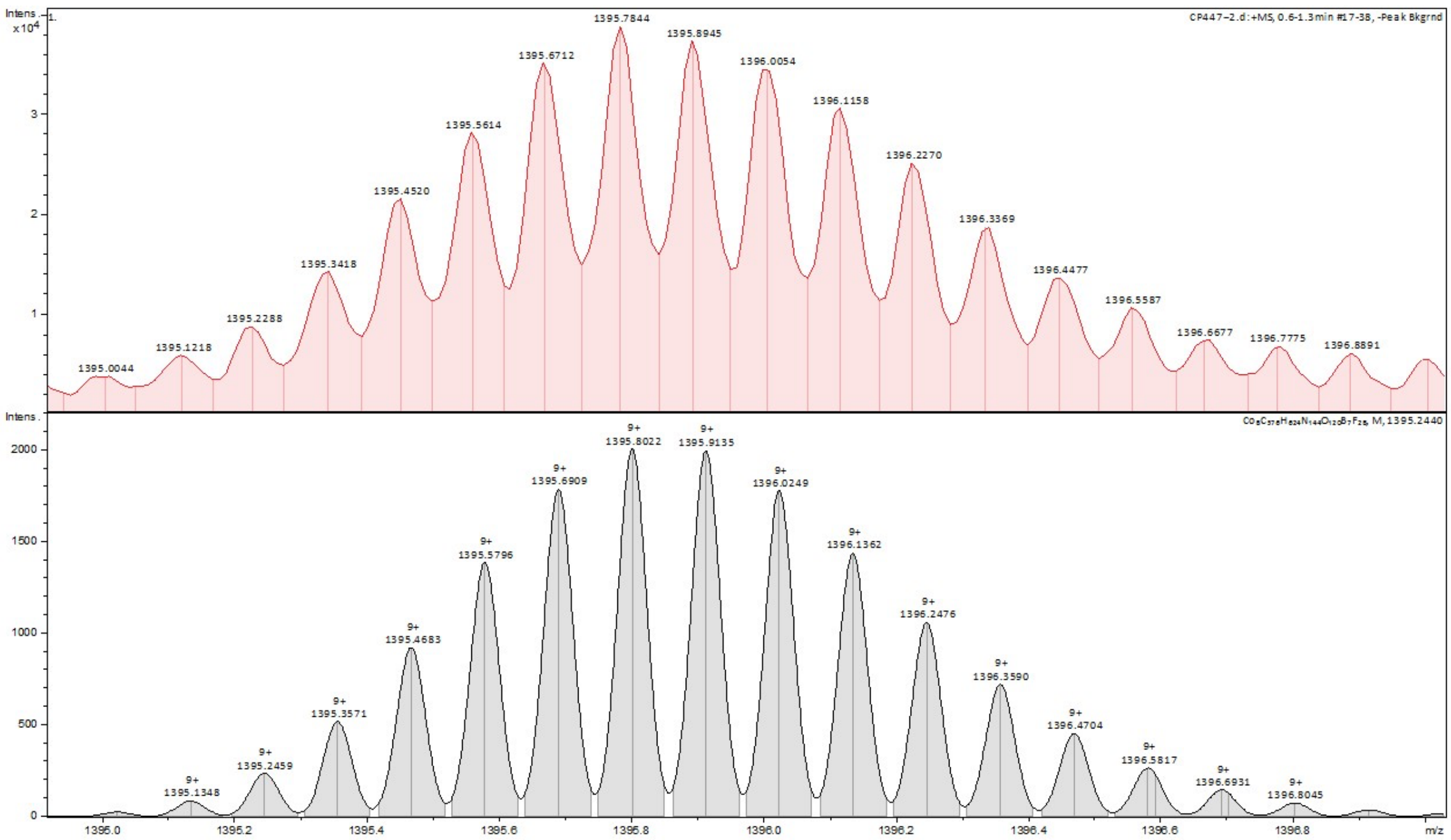

**Figure S37:** see below for caption / details

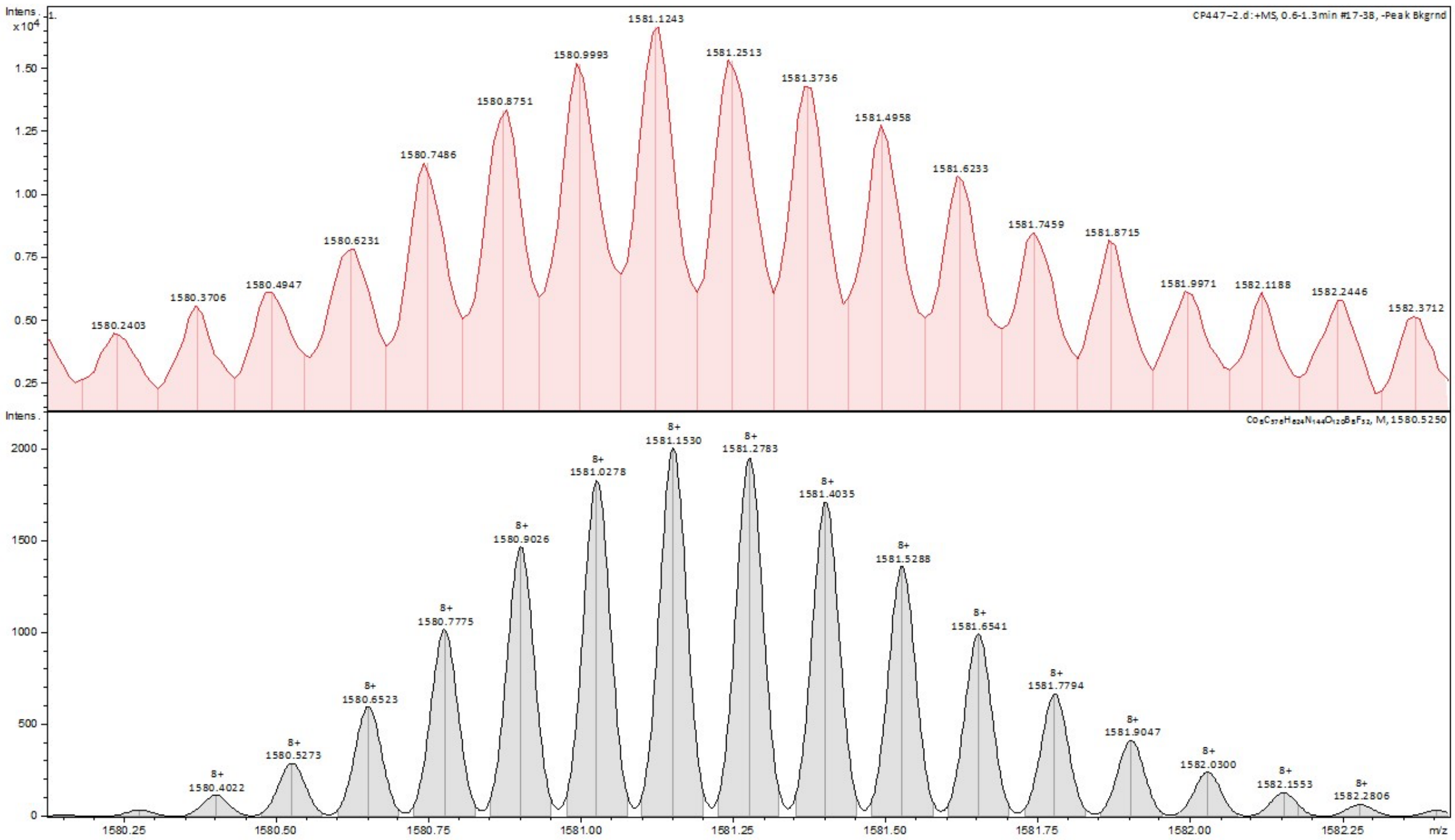


**Figure S38:** see below for caption / details

**Figures S35-38.** Selected expansions of the HR-ESI-MS spectrum for $[Co_8(L^{FF})_{12}](BF_4)_{16}$ (main spectrum in Fig. S34) in $H_2O$ (250 μM) showing expansions of the signals for the species $[\{Co_8(L^{FF})_{12}\}(BF_4)_5]^{11+}$, $[\{Co_8(L^{FF})_{12}\}(BF_4)_6]^{10+}$, $[\{Co_8(L^{FF})_{12}\}(BF_4)_7]^{9+}$ and $[\{Co_8(L^{FF})_{12}\}(BF_4)_8]^{8+}$. The calculated isotopic patterns are shown below each observed signal.

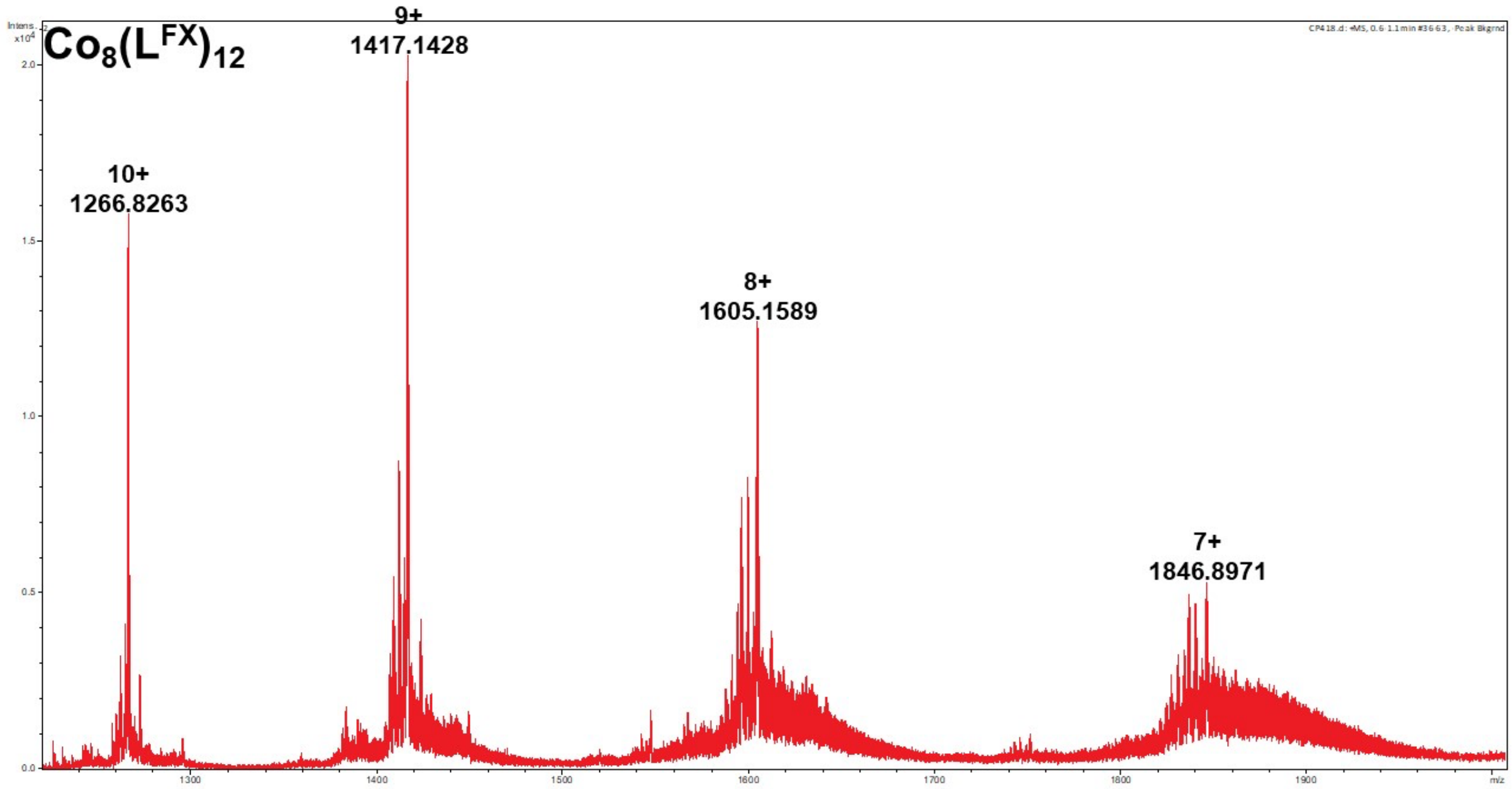


**Figure S39.** HR-ESI-MS of $[Co_8(L^{FX})_{12}(BF_4)_{16}]$ in $H_2O$ (250 μM) where n = number of $BF_4$ anions lost and hence the charge in the sequence $[\{Co_8(L^{FX})_{12}\}(BF_4)_{16-n}]^{n+}$.

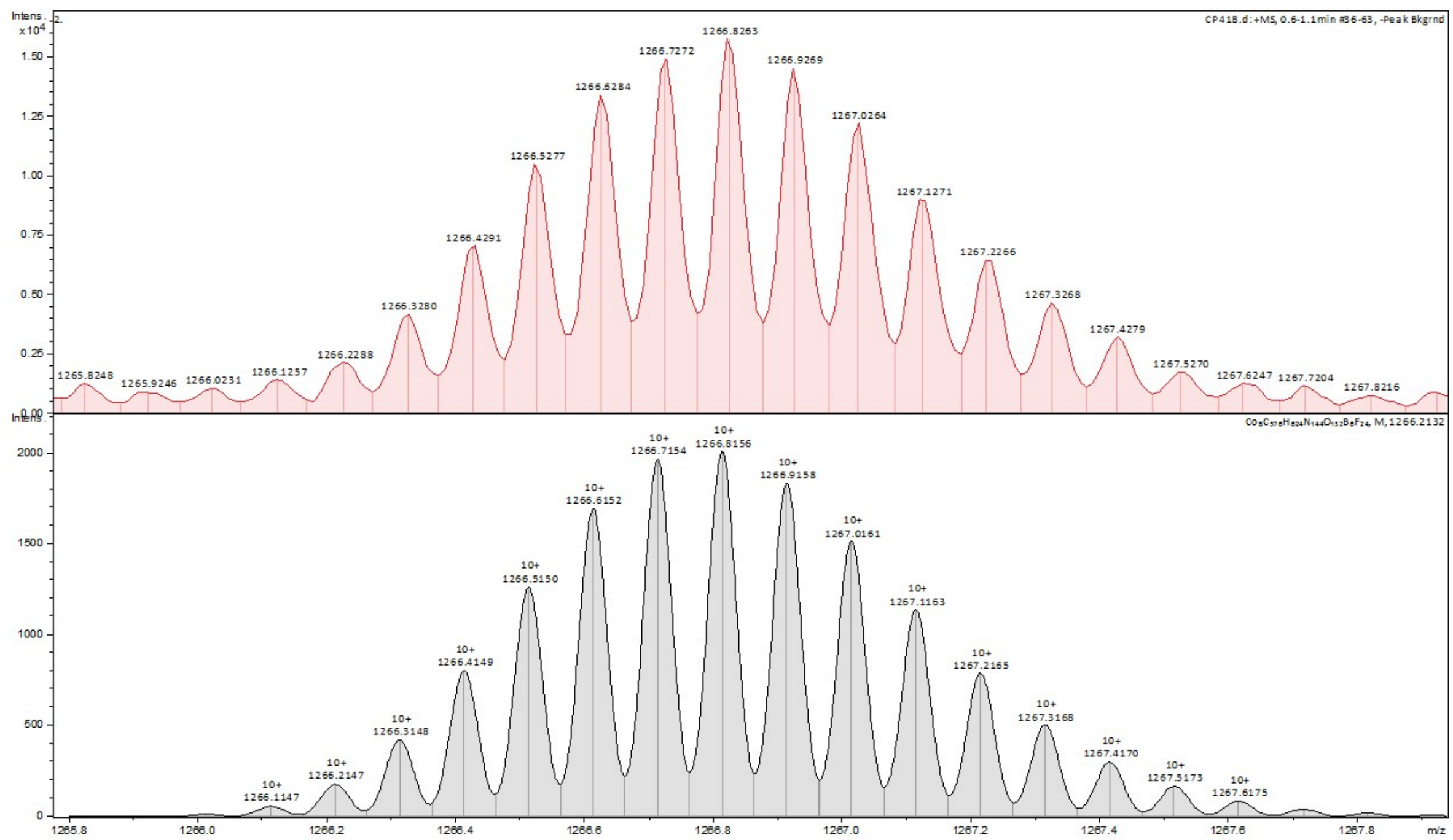

**Figure S40**: see below for caption / details.

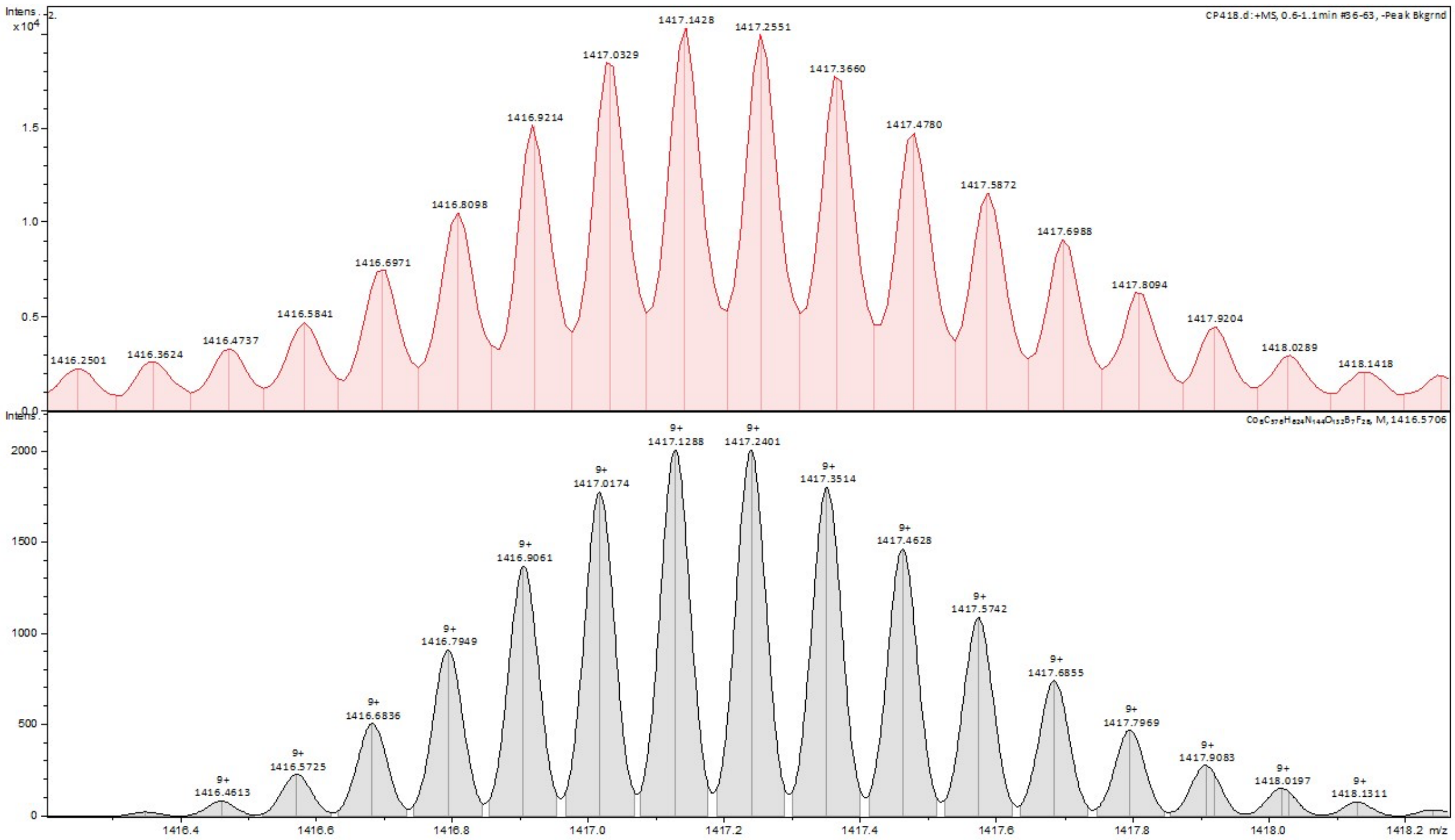


**Figure S41**: see below for caption / details.

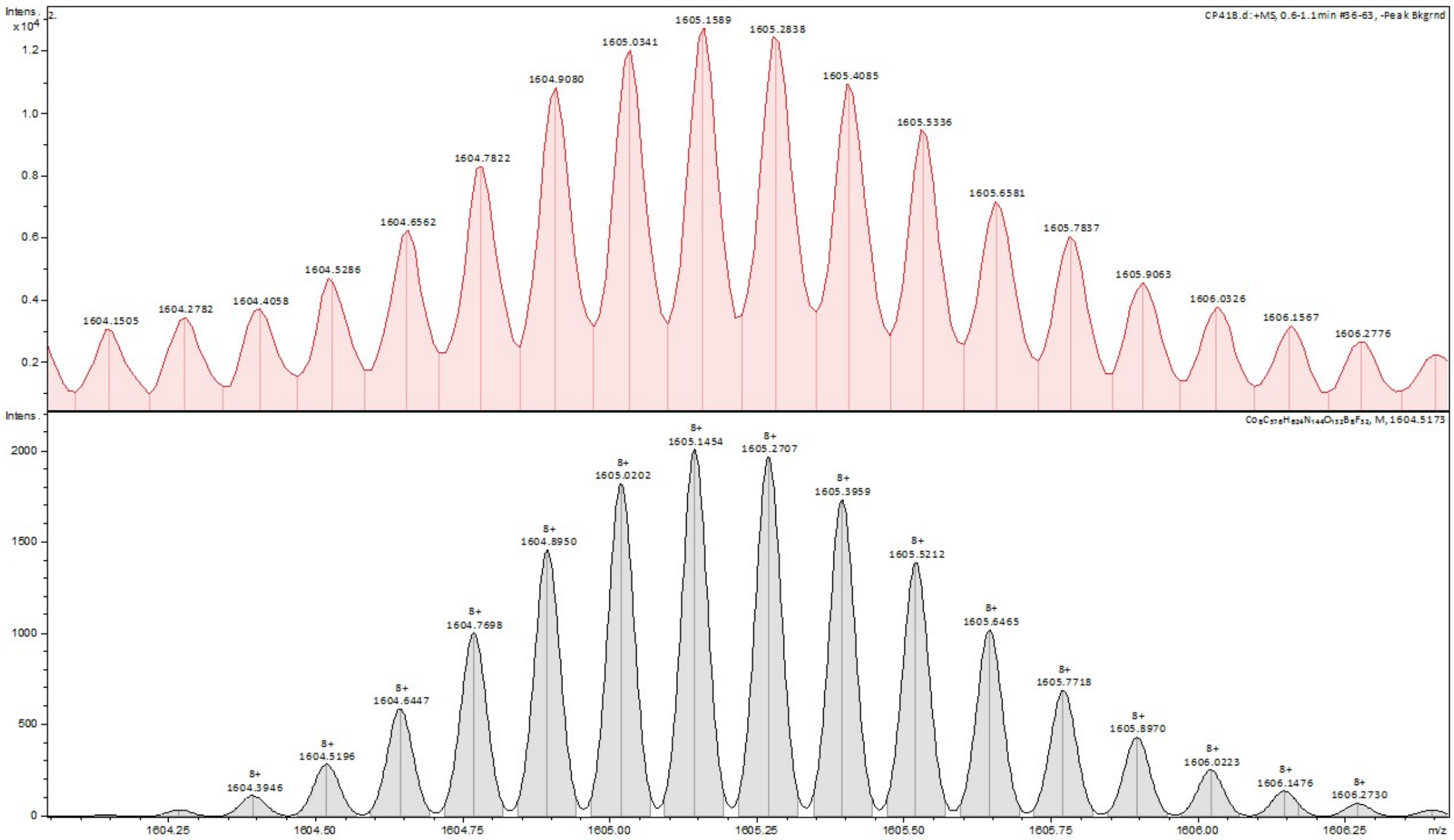


**Figure S42**: see below for caption / details.

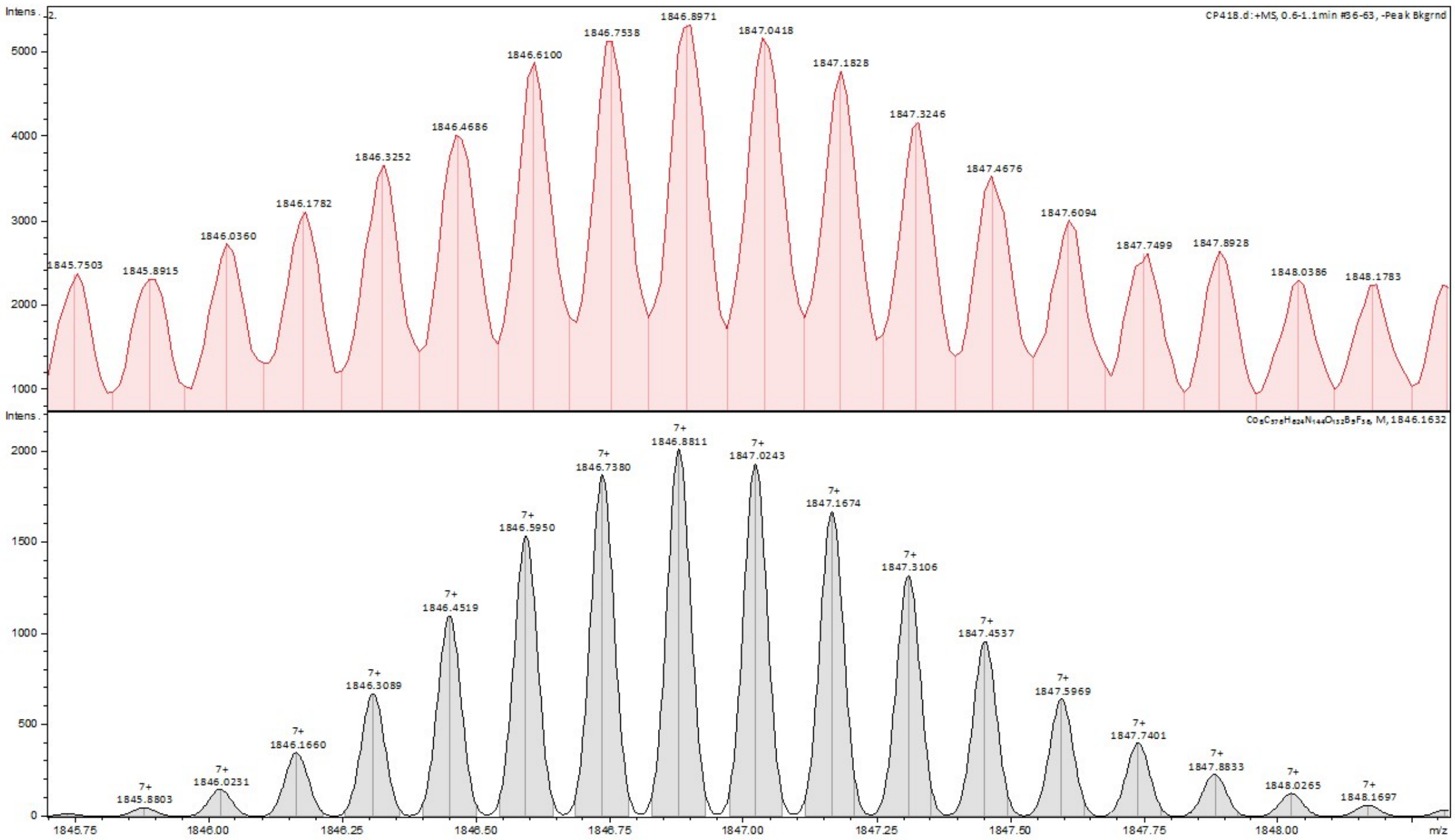


**Figure S43**: see below for caption / details.

**Figures S40-43.** Selected expansions of the HR-ESI-MS spectrum of $[Co_8(L^{FX})_{12}](BF_4)_{16}$ (main spectrum in Fig. S39) in $H_2O$ (250 µM) showing expansions of the signals for the species $[\{Co_8(L^{FX})_{12}\}(BF_4)_6]^{10+}$, $[\{Co_8(L^{FX})_{12}\}(BF_4)_7]^{9+}$, $[\{Co_8(L^{FX})_{12}\}(BF_4)_8]^{8+}$ and $[\{Co_8(L^{FX})_{12}\}(BF_4)_9]^{7+}$. The calculated isotopic patterns are shown below each observed signal.

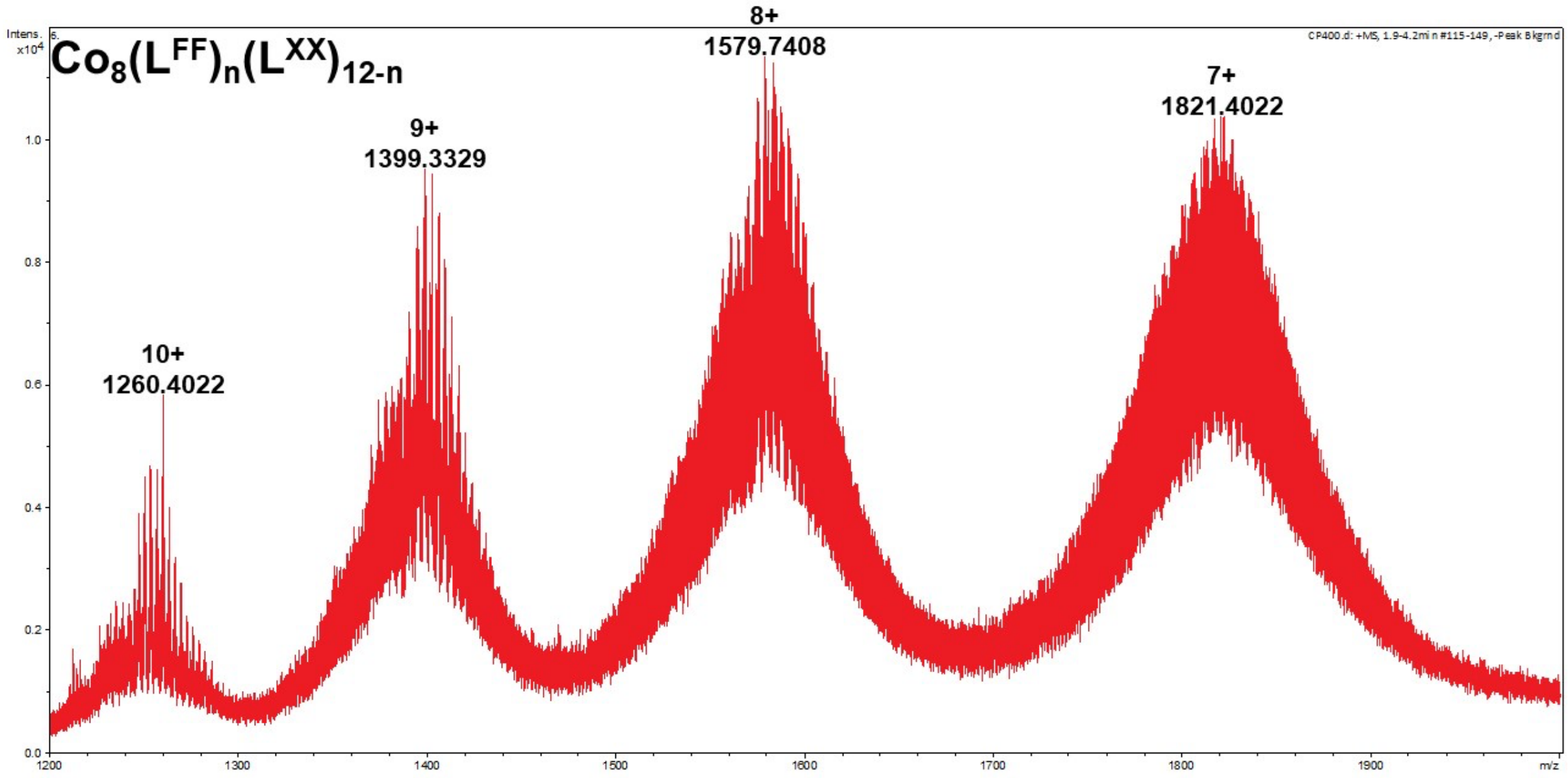


**Figure S44.** HR-ESI-MS of $[Co_8(L^{FF})_n(L^{XX})_{12-n}(BF_4)_{16}]$ in $H_2O$ (250 μM) where n = number of $BF_4$ anions lost and hence the charge in the sequence $[\{Co_8(L^{FF})_n(L^{XX})_{12-n}\}(BF_4)_{16-n}]^{n+}$.

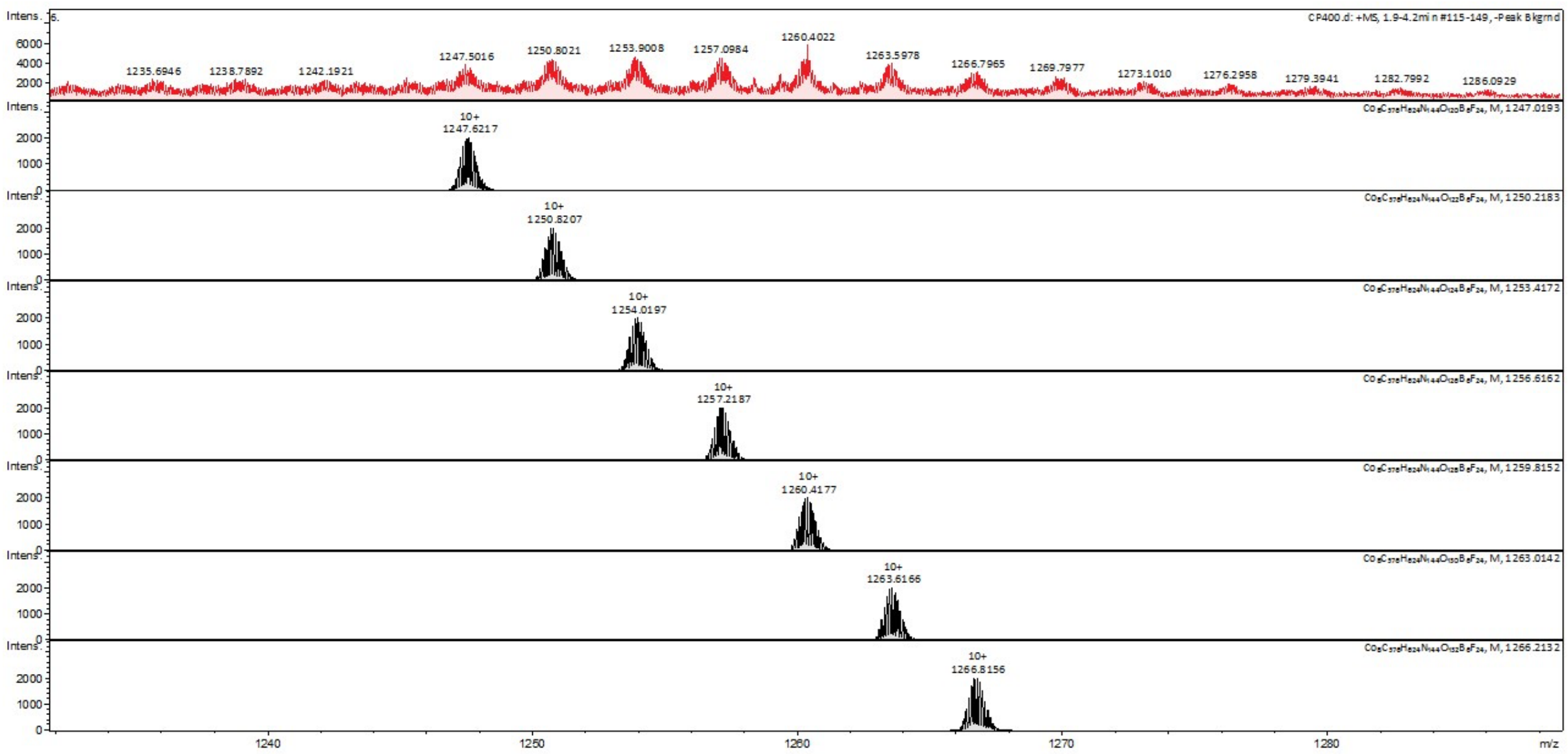


**Figure S45**: see below for caption / details.

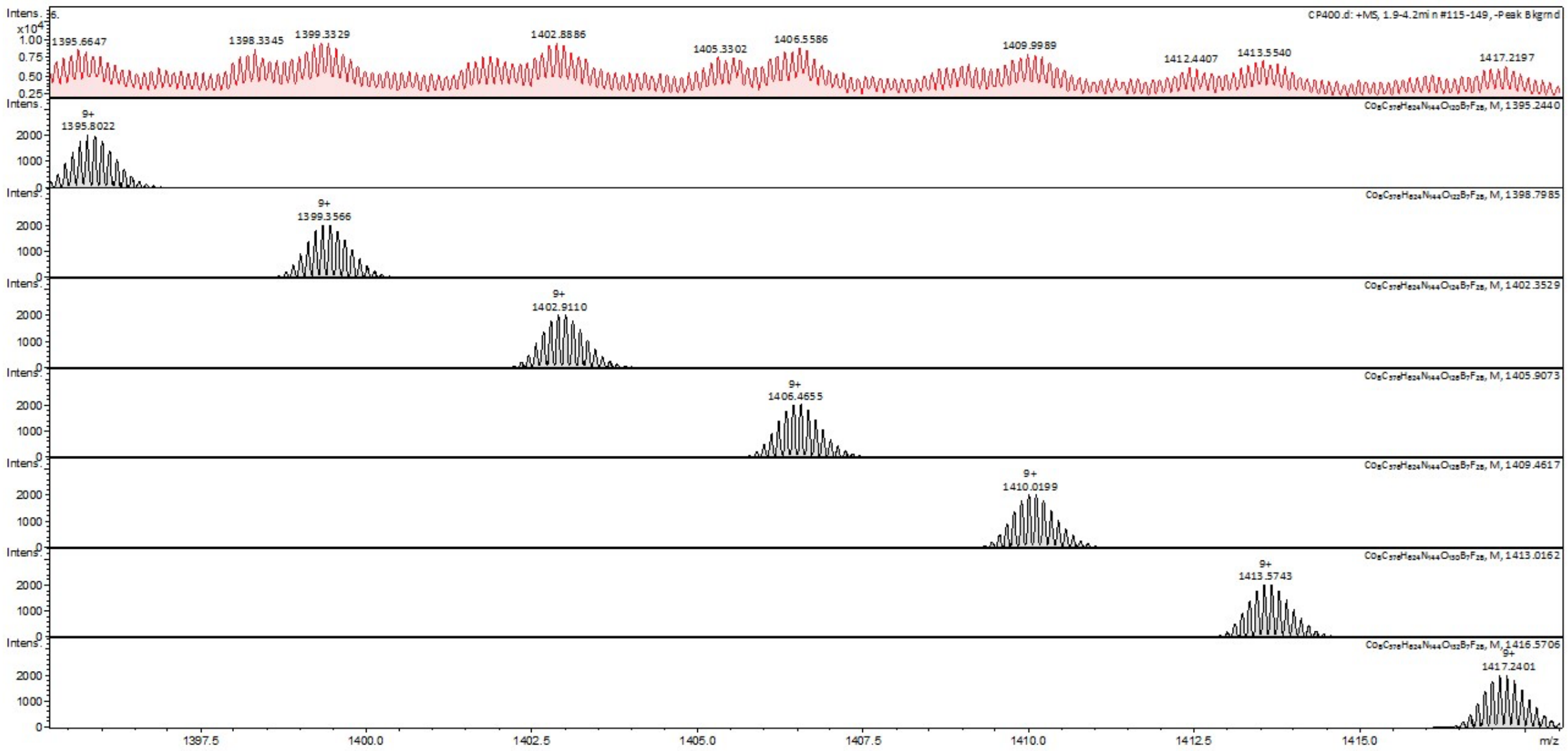

**Figure S46**: see below for caption / details.

**Figures S45-46.** Selected expansions of the HR-ESI-MS spectrum of $[Co_8(L^{FF})_n(L^{XX})]_{12-n}(BF_4)_{16}$ (main spectrum in Fig. S44) in $H_2O$ (250 μM) showing the 10+ species $[\{Co_8(L^{FF})_n(L^{XX})_{12-n}\}(BF_4)_6]^{10+}$ at m/z 1247.0516 to 1266.7965 and the 9+ species $[\{Co_8(L^{FF})_n(L^{XX})_{12-n}\}(BF_4)_7]$ at m/z 1395.6647 to 1417.2197. The theoretical isotopic patterns are shown below each observed isotopic pattern.

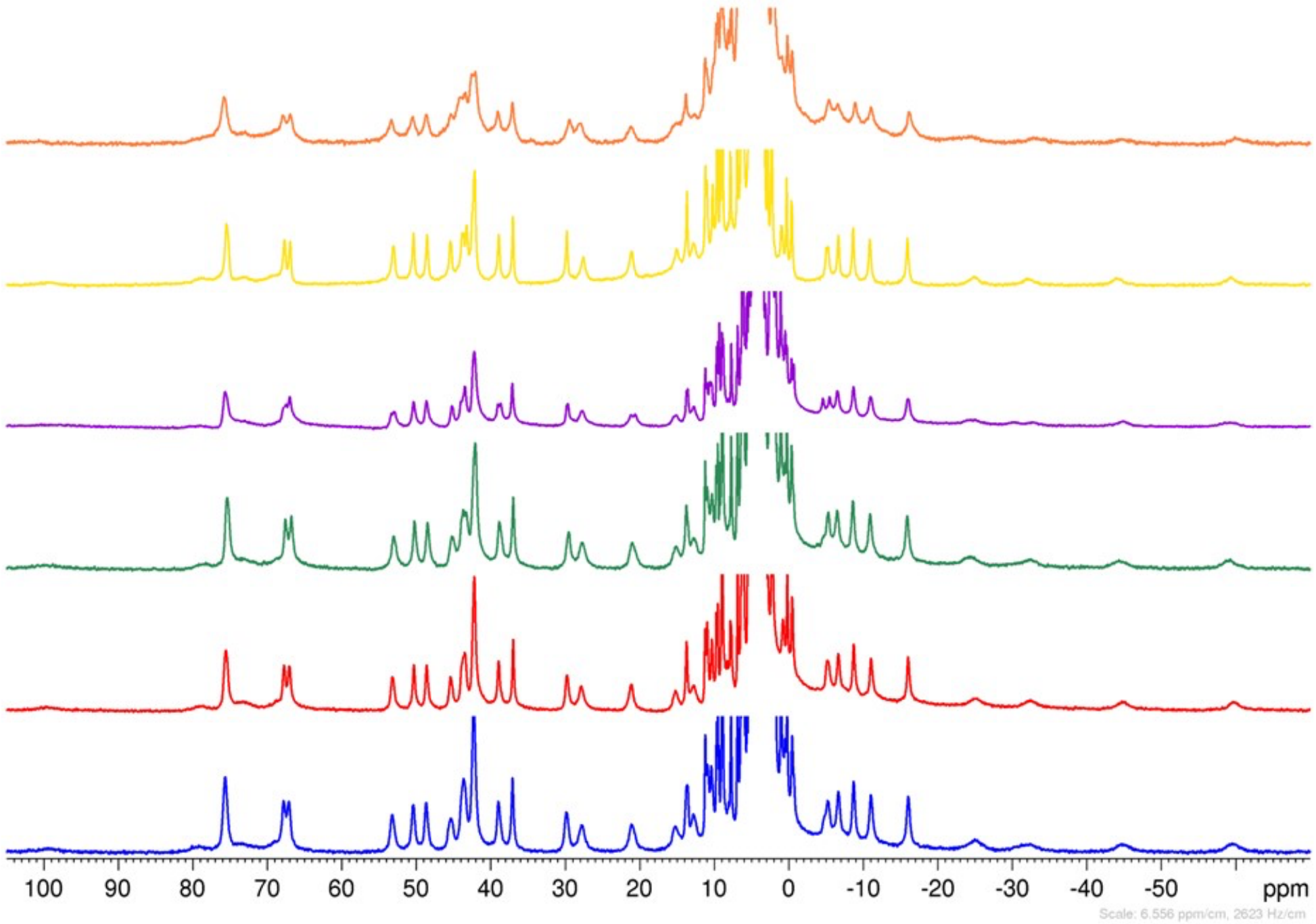


**Figure S47.** $^{1}$H NMR spectra (400 MHz, $D_2O$, 363 K, 200 μM) of **$[Co_8(L^{FF})_n(L^{GG})_{12-n}](BF_4)_{16}$** (blue), **$[Co_8(L^{GG})_n(L^{XX})_{12-n}](BF_4)_{16}$** (red), **$[Co_8(L^{FF})_n(L^{XX})_{12-n}](BF_4)_{16}$** (green), **$[Co_8(L^{FF})_{12}](BF_4)_{16}$** (purple), **$[Co_8(L^{GG})_{12}](BF_4)_{16}$** (yellow) and **$[Co_8(L^{XX})_{12}](BF_4)_{16}$** (orange).

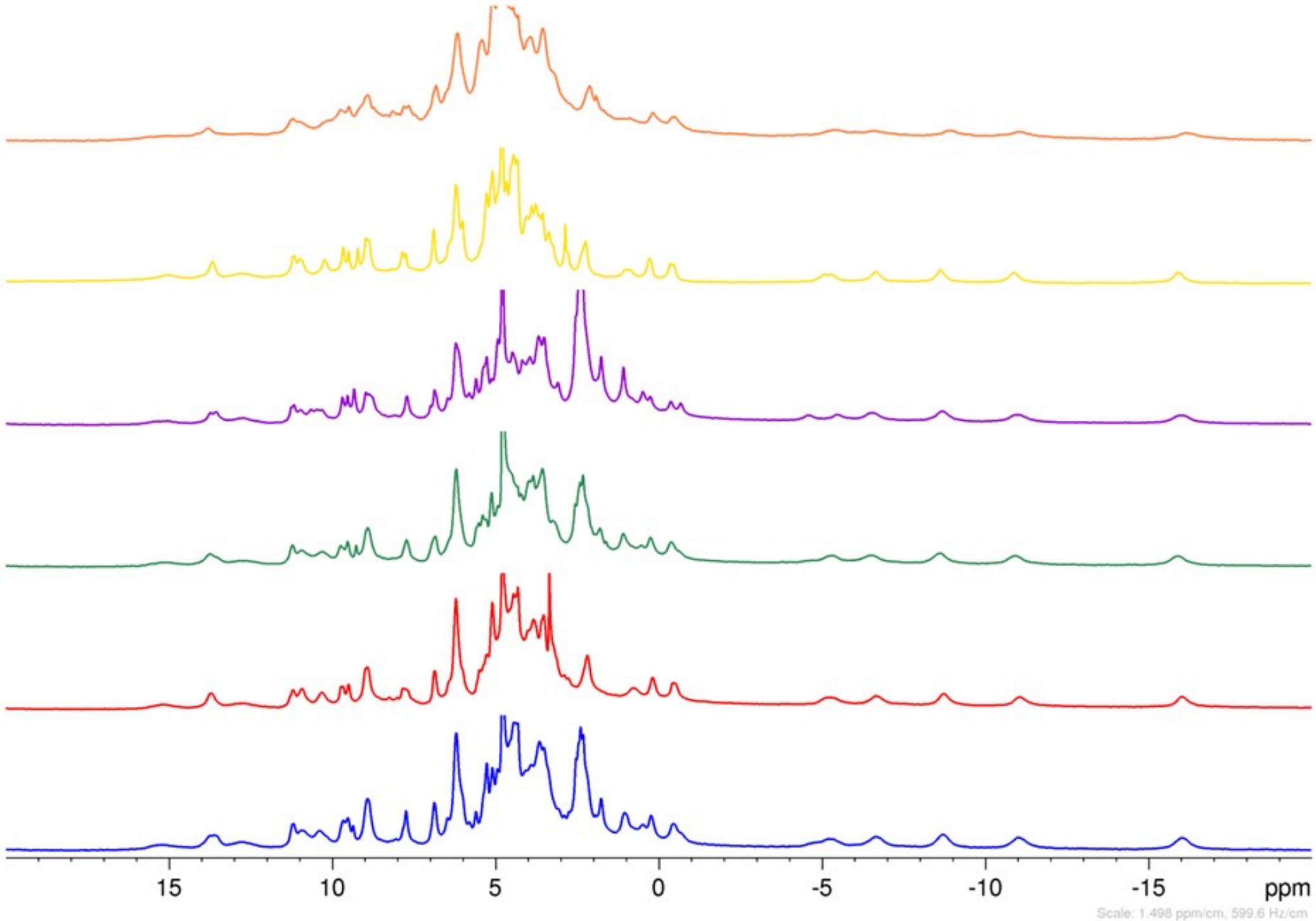


**Figure S48.** Expansion of Fig. S47 between 20 and -20 ppm.

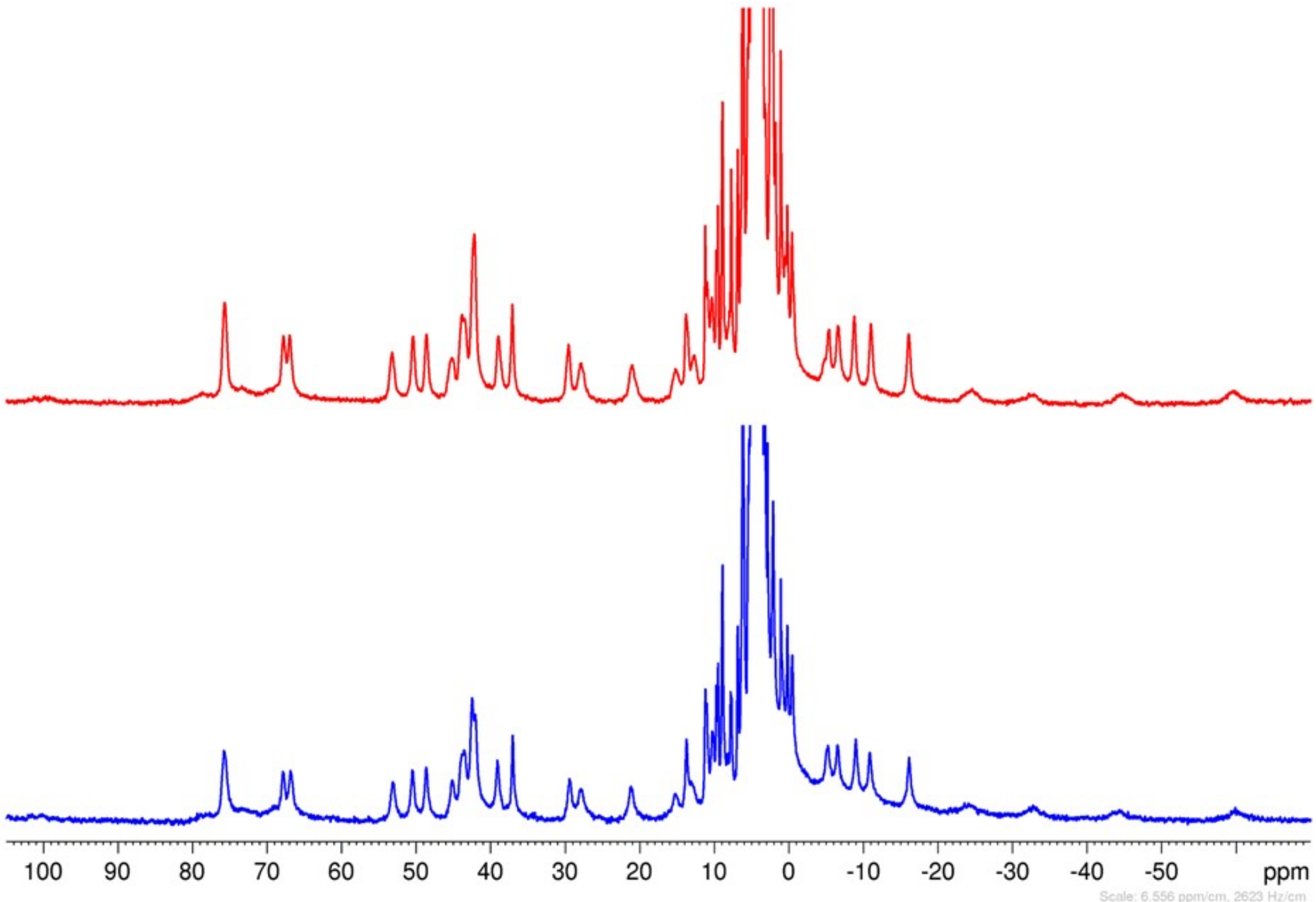


**Figure S49.** $^{1}$H NMR spectra (400 MHz, $D_2O$, 363 K, 200 μM) of **$[Co_8(L^{GX})_{12}](BF_4)_{16}$** (blue) and **$[Co_8(L^{FX})_{12}](BF_4)_{16}$** (red). Note the presence of the four broad signals between –20 and –60 ppm which is highly characteristic of the $S_6$-symmetric octanuclear cage core (ref. 13b, main text).

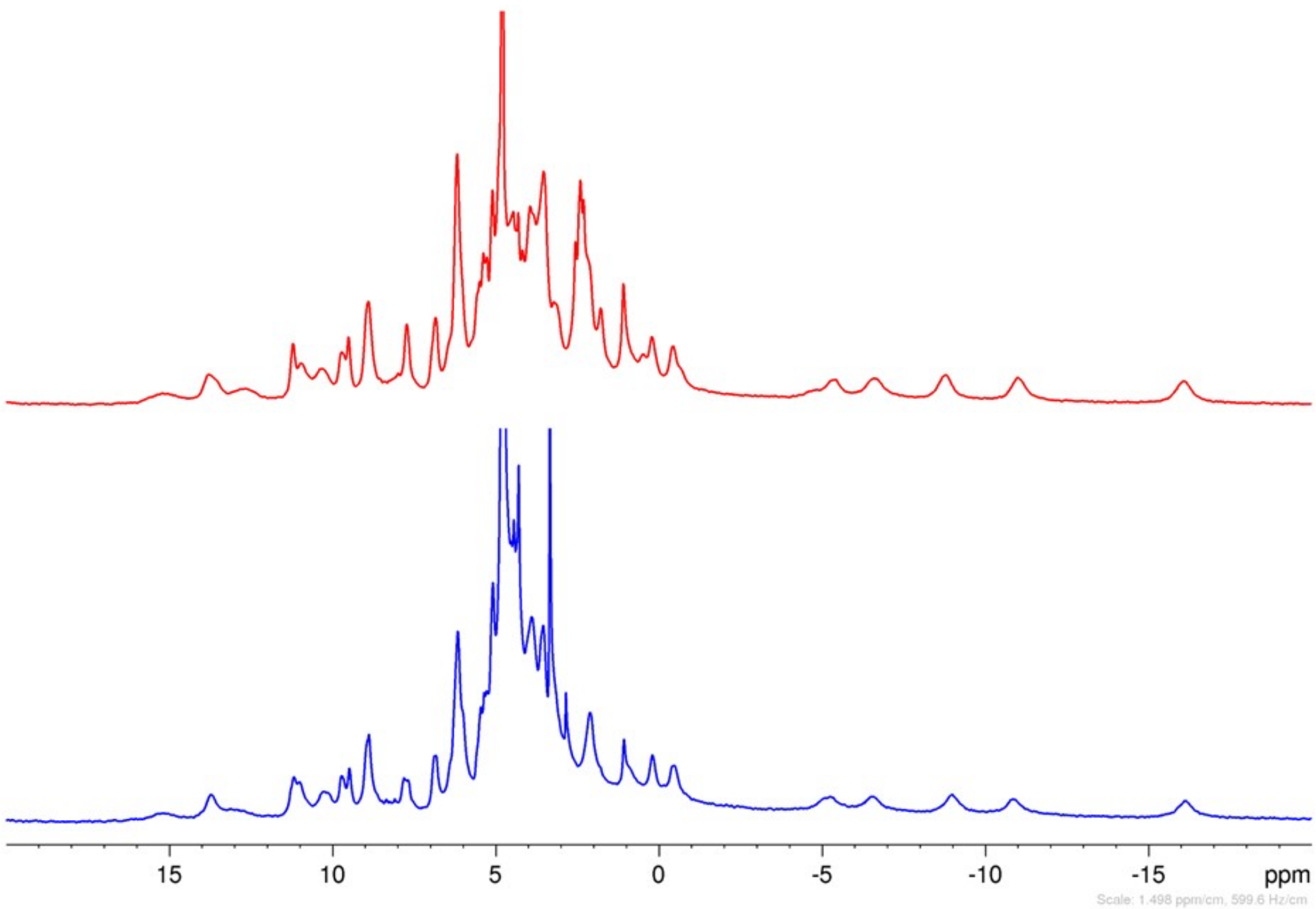


**Figure S50.** Expansion of Fig. S49 between 20 and -20 ppm.

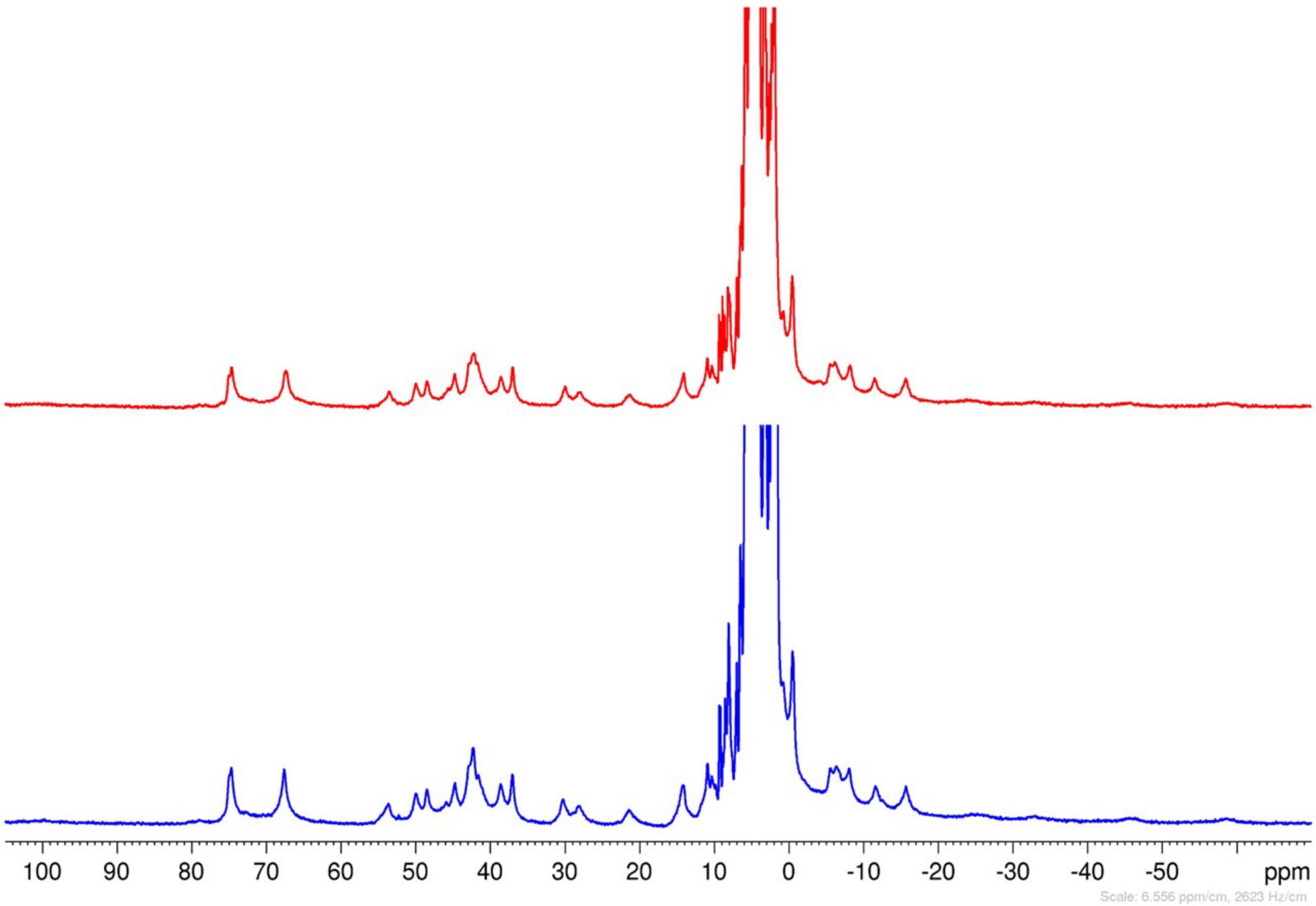


**Figure S51.** $^{1}H$ NMR spectra (400 MHz, $D_2O$, 363 K, 200 μM) of **$[Co_8(L^{PEG5\text{-}FF})_{12}](BF_4)_{16}$** (blue) and **$[Co_8(L^{PEG5\text{-}XX})_{12}](BF_4)_{16}$** (red).

## 5. Tabulated $^1$H NMR data for $[Co_8(L^{FF})_n(L^{XX})]_{12-n}(BF_4)_{16}$

**Table S1.** Tentative $^1$H NMR assignments for $[Co_8(L^{FF})_n(L^{XX})]_{12-n}(BF_4)_{16}$ in $D_2O$ at 363 K consistent with previously reported NMR spectra of cages of this type.[3]

| δ/ppm | Proton Assignment |
|---|---|
| 75.4 | Naphthyl + Pyrazolyl |
| 67.6 | Pyrazolyl |
| 66.8 | Pyrazolyl |
| 53.0 | Pyridyl |
| 50.2 | Pyrazolyl |
| 48.5 | Pyridyl |
| 45.2 | Pyridyl |
| 43.7 | Pyrazolyl |
| 43.3 | Pyrazolyl |
| 42.1 | Naphthyl + Pyrazolyl |
| 38.9 | Pyridyl |
| 37.0 | Naphthyl |
| 29.5 | Pyridyl |
| 27.7 | Naphthyl |
| 21.0 | Naphthyl |
| 15.1 | Naphthyl |
| 13.8 | Pyridyl |
| 12.8 | Naphthyl |
| -0.4 | Pyridyl |
| -5.3 | $CH_2$ |
| -6.5 | $CH_2$ |
| -8.6 | Pyridyl |
| -10.9 | $CH_2$ |
| -15.9 | $CH_2$ |
| -24.3 | $CH_2$ |
| -32.5 | $CH_2$ |
| -44.3 | $CH_2$ |
| -59.3 | $CH_2$ |

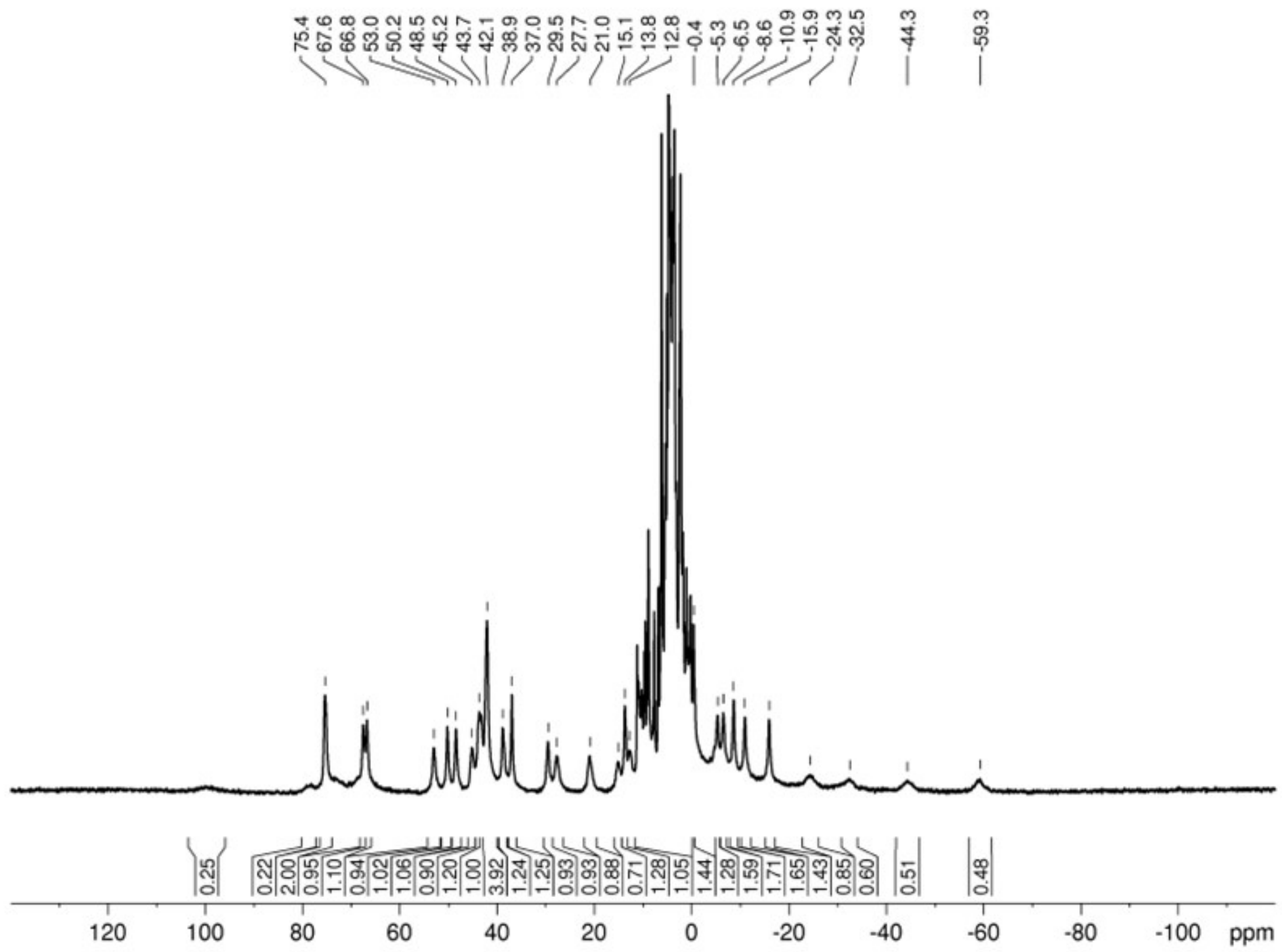

**Figure S52.** Partially assigned $^{1}H$ NMR spectrum (400 MHz, $D_2O$, 363 K, 200 μM) of $\mathbf{[Co_8(L^{FF})_n(L^{XX})]_{12-n}(BF_4)_{16}}$.

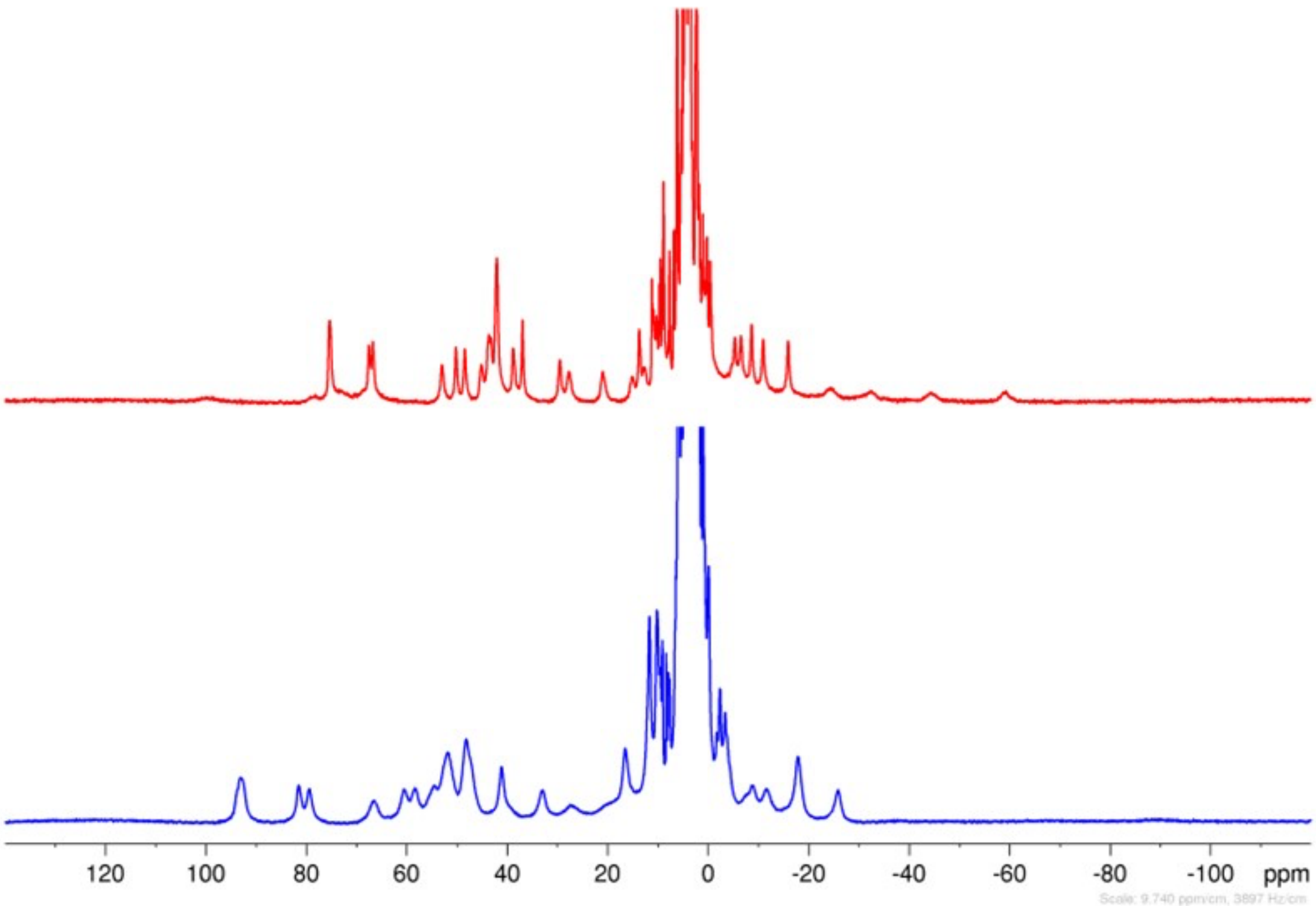


**Figure S53.** $^{1}$H NMR spectra (400 MHz, $D_2O$, 200 μM) of **$[Co_8(L^{FF})_n(L^{XX})_{12-n}](BF_4)_{16}$** at 298 K (blue) and 363 K (red).

# 6. Dynamic Light Scattering (DLS) Experiments

DLS experiments were performed at 25 °C using an Anton Paar Litesizer™ 500 with a 658 nm laser. The measurement angle was varied between 15° and 175° for Particle size series measurements. Quartz cuvettes (Hellma Analytics) and disposable cuvettes (Fisher Scientific) were used with sample volumes of ~1 mL and sample concentrations of between 2 and 10 mg/mL in HEPES buffer (10 mM, 0.1 mM $CaCl_2$, pH 8.5) or water. All sample solutions were filtered through a Whatman® Anotop® 10 0.02 µm syringe filter or a Fisherbrand™ 0.2 µm syringe filter prior to measurements. The hydrodynamic diameter ($D_h$, nm) values are the average of three (10 sec) scans ± standard deviation (σ).

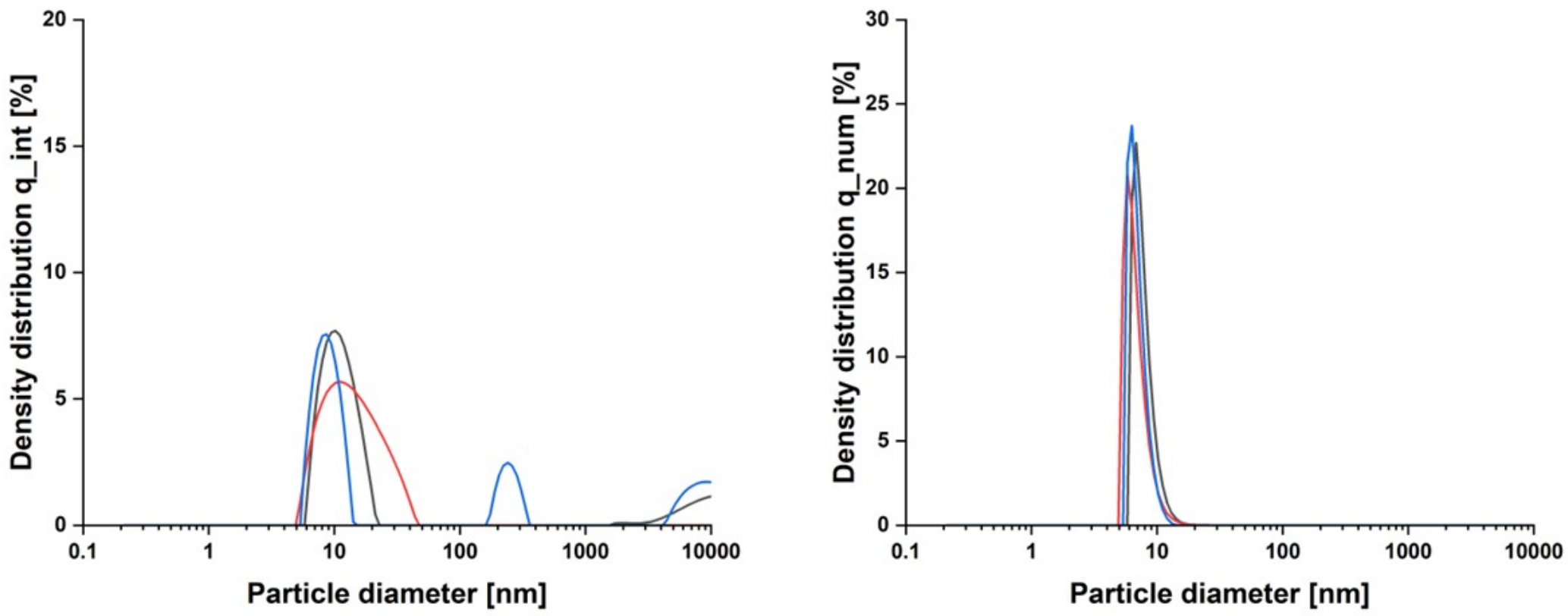


**Figure S54.** DLS data of a 10 µM solution of Jacalin.

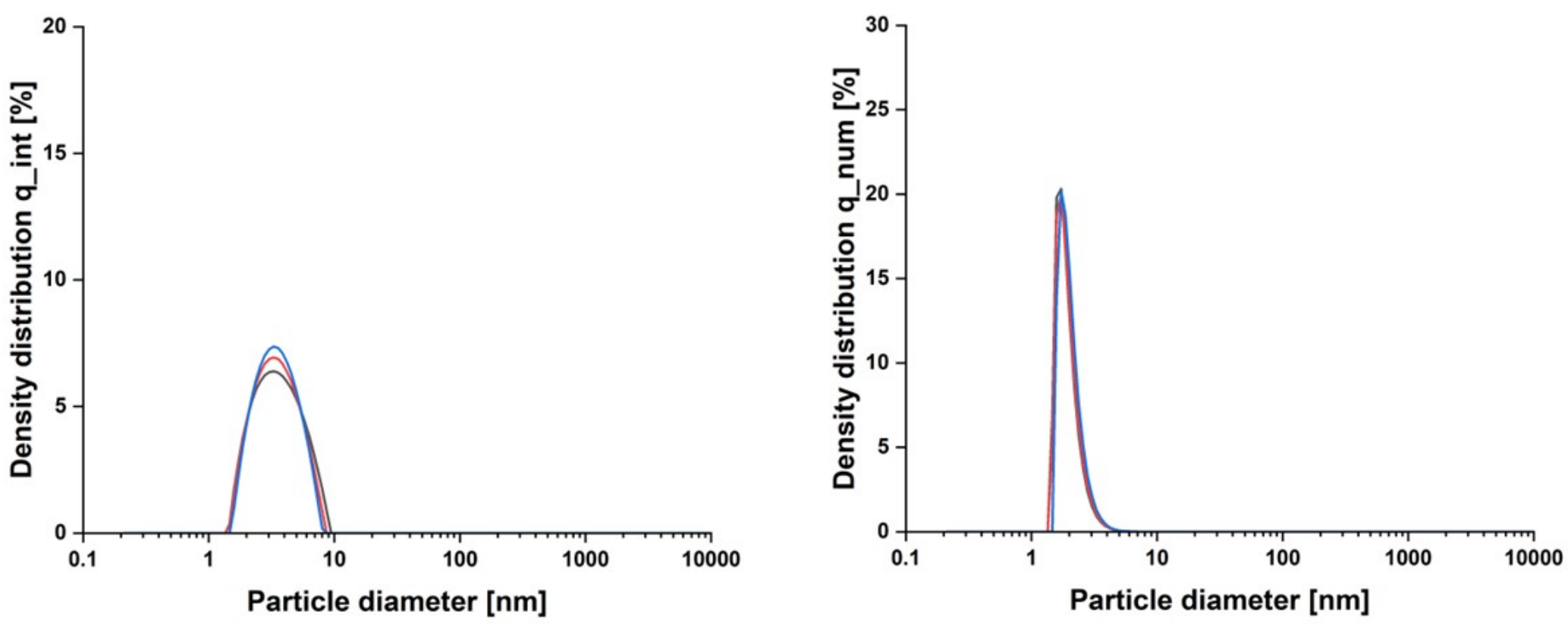


**Figure S55.** DLS data of a 10 mg/mL solution of **$[Co_8(L^{PEG3})_{12}](BF_4)_{16}$**.

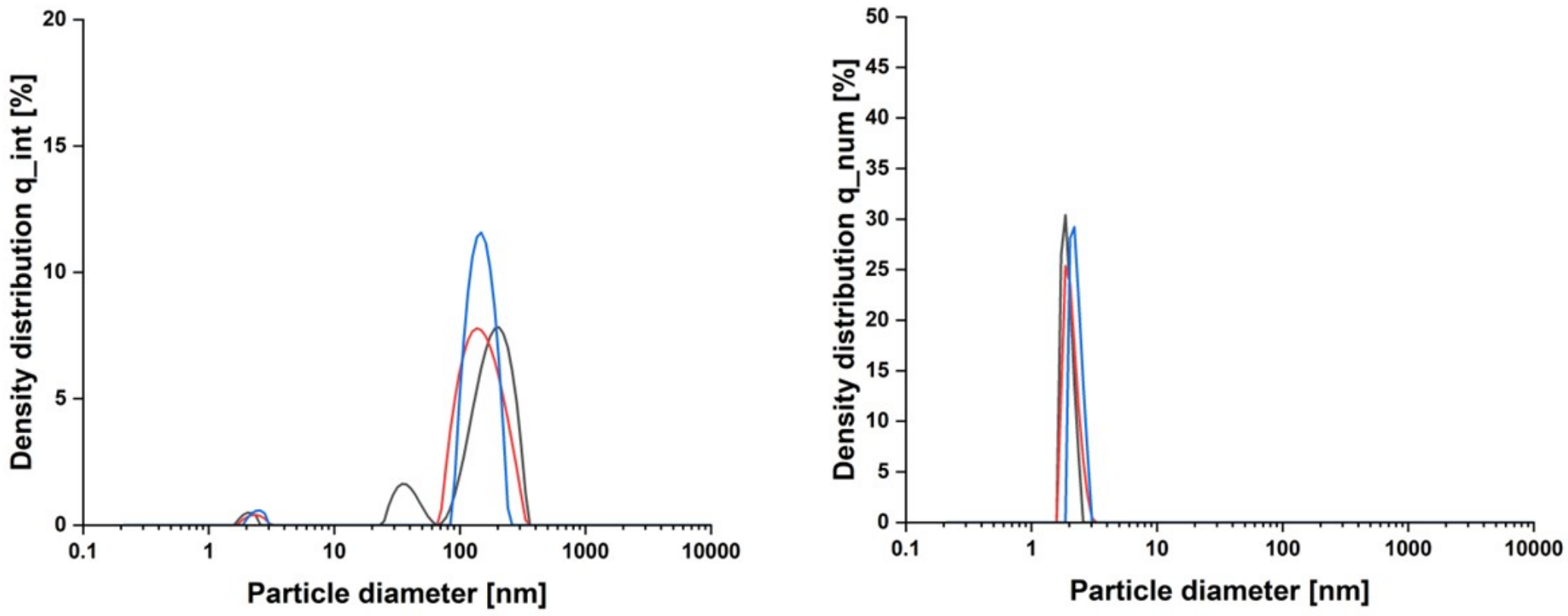


**Figure S56.** DLS data of a 20 mg/mL solution of $\mathbf{[Co_8(L^{XX})_{12}](BF_4)_{16}}$.

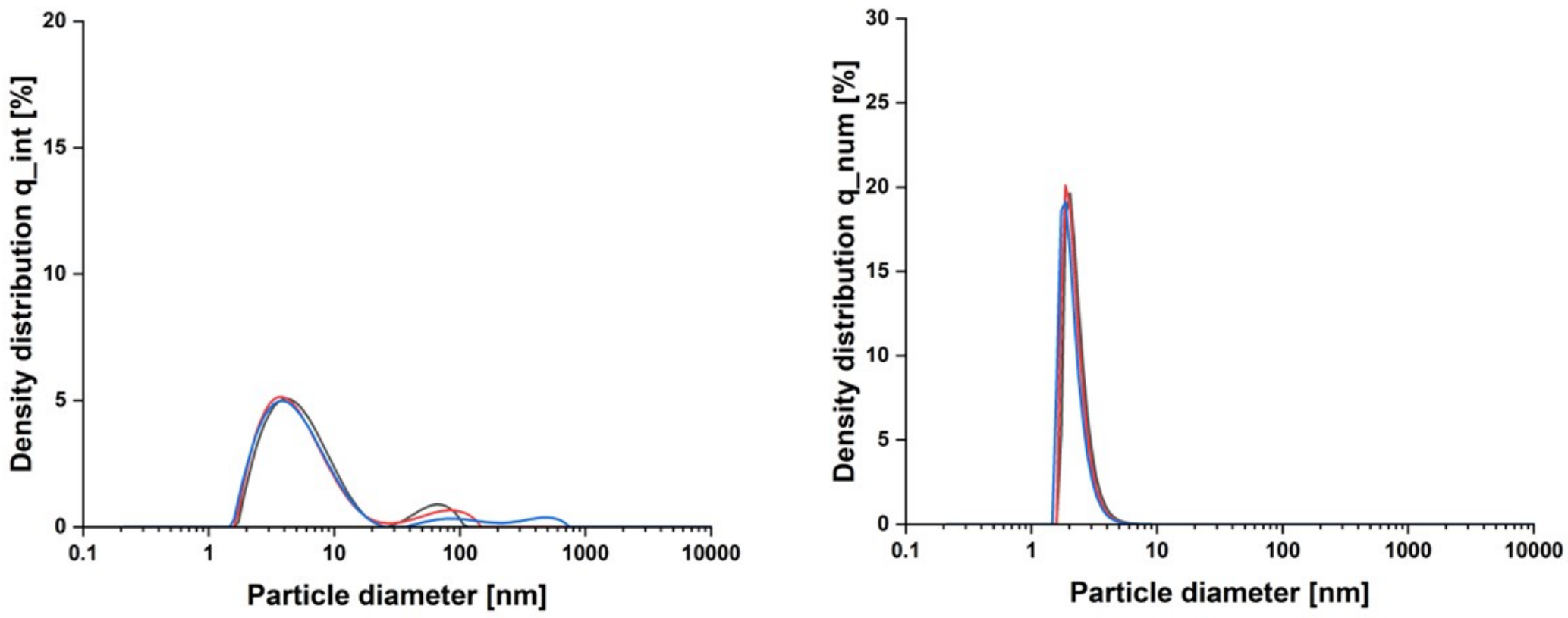


**Figure S57.** DLS data of a 10 mg/mL solution of $\mathbf{[Co_8(L^{FF})_n(L^{XX})_{12\text{-}n}](BF_4)_{16}}$.

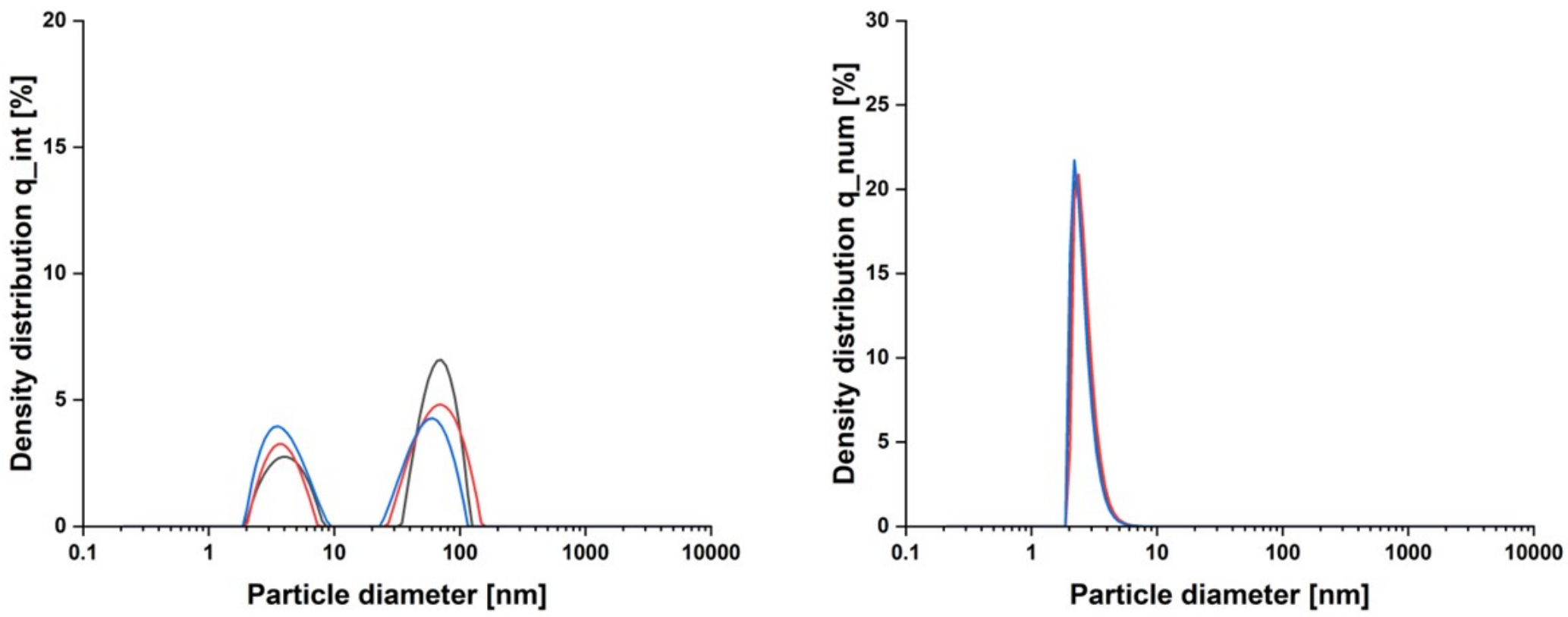


**Figure S58.** DLS data of a 10 mg/mL solution of $\mathbf{[Co_8(L^{PEG5\text{-}XX})_{12}](BF_4)_{16}}$.

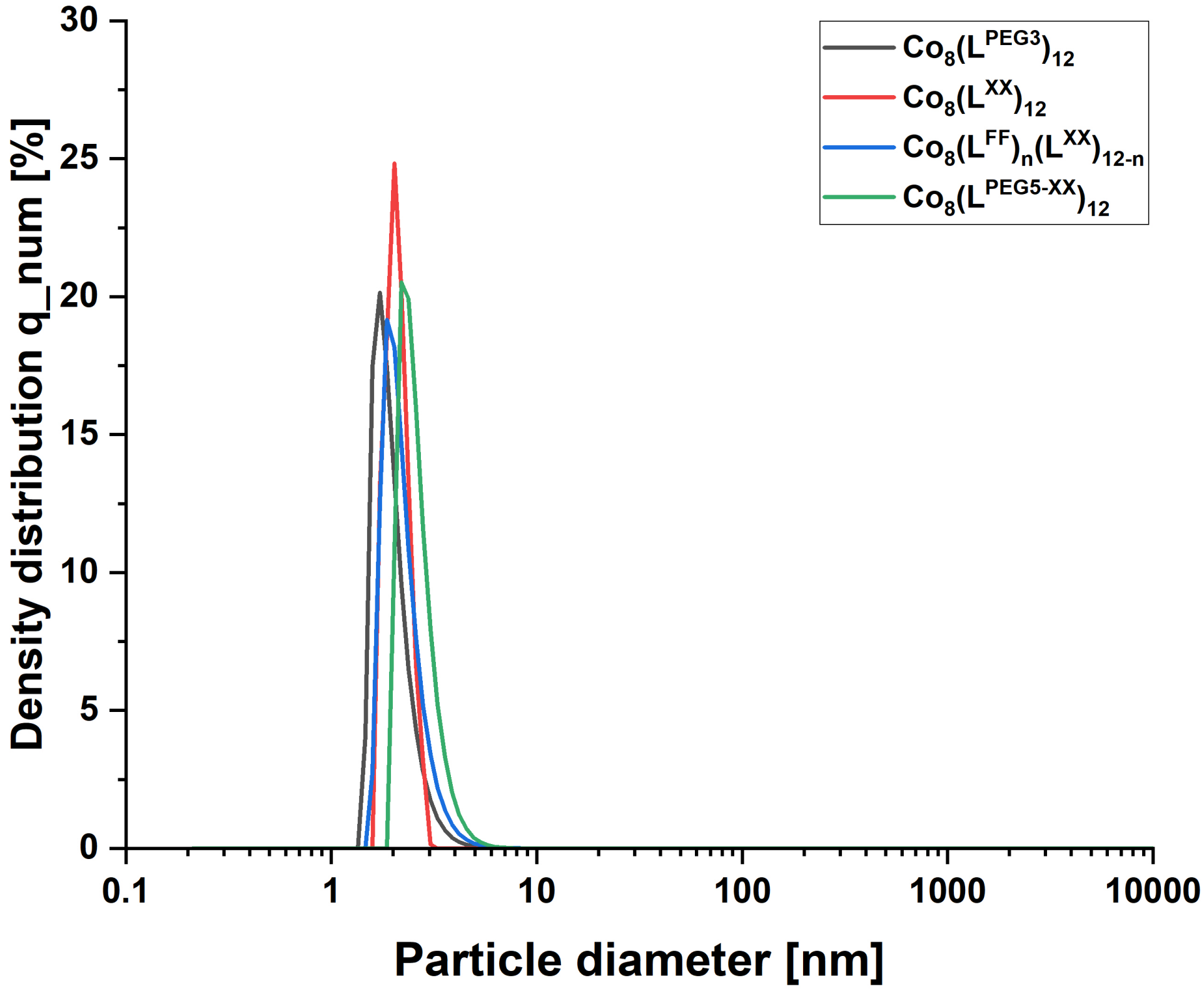


**Figure S59.** Combined DLS data of Figures S55-58.

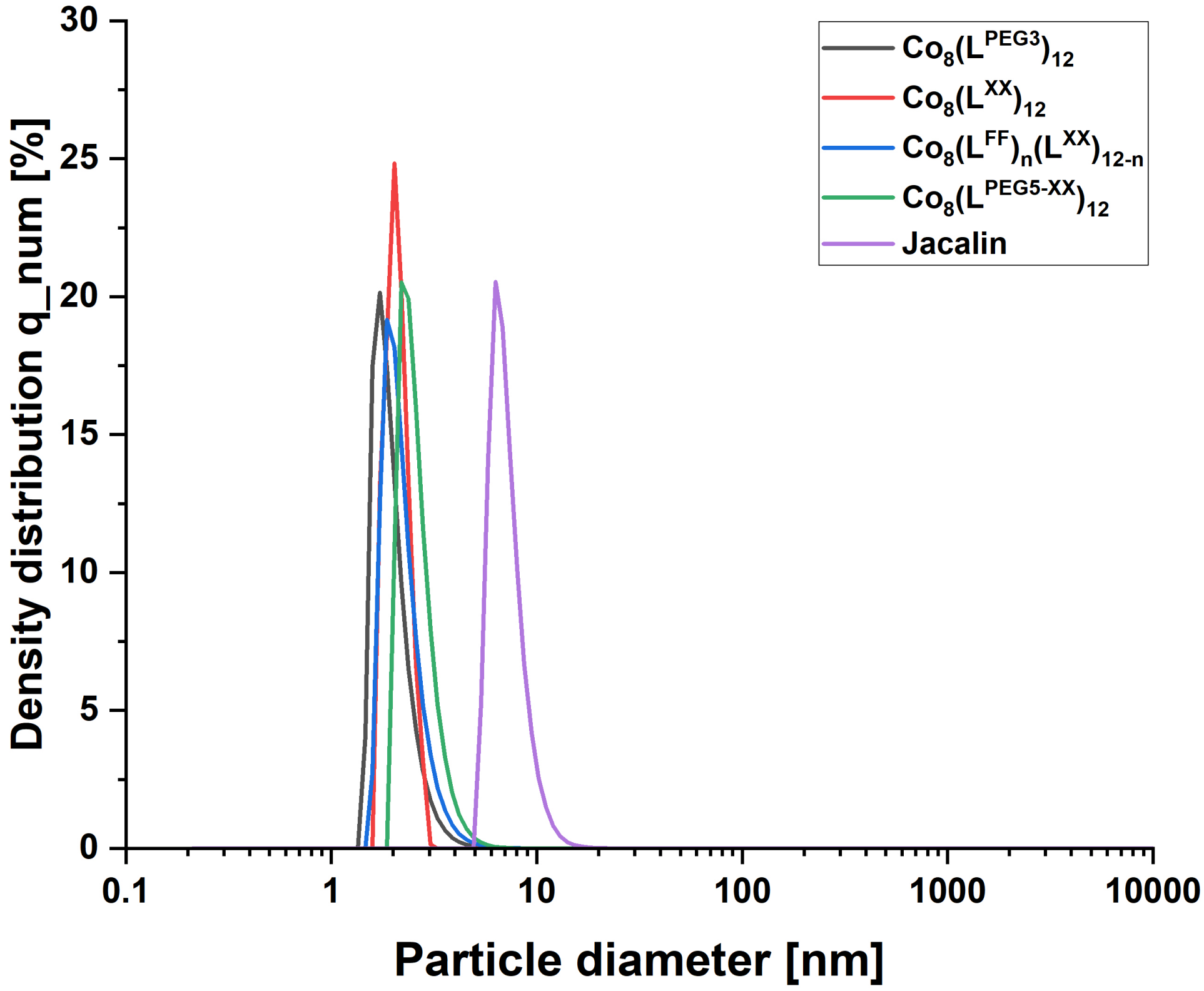


**Figure S60.** Combined DLS data of Figures S54-58.

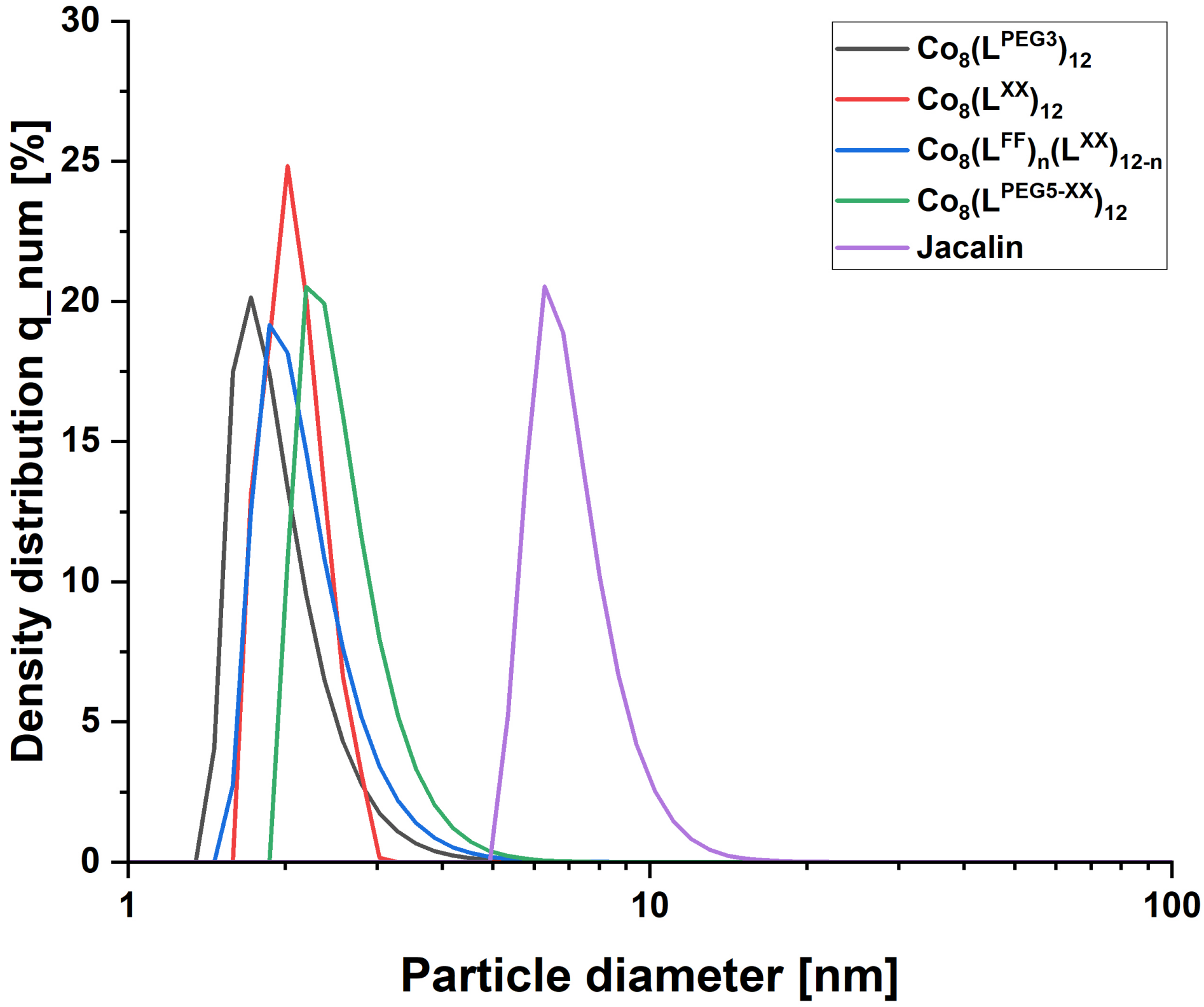


**Figure S61.** Expansion of Figure S60 between 1 and 100 nm.

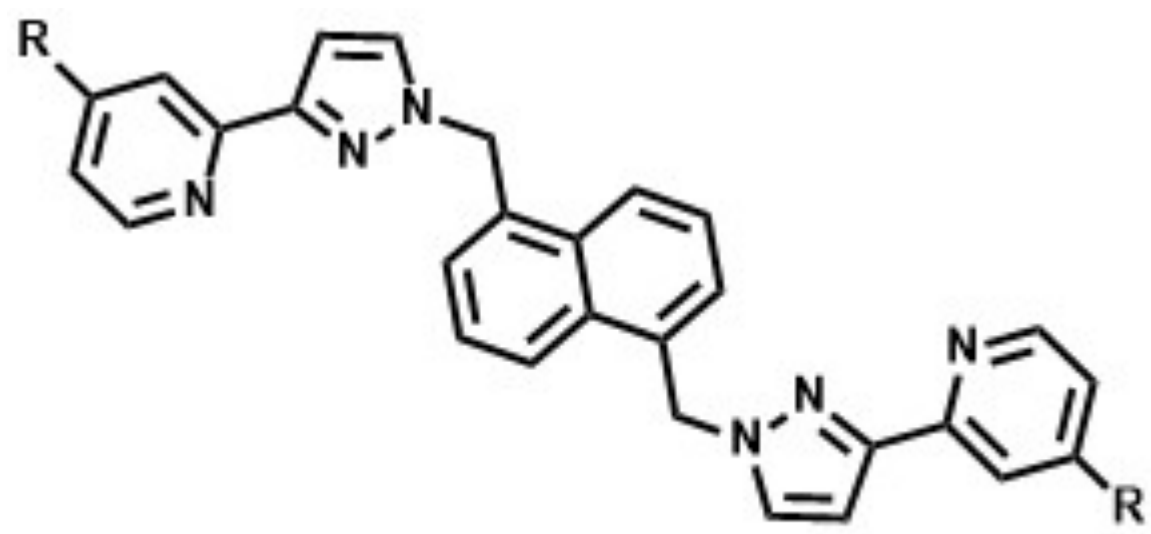


$L^{PEG3}$: R = $(CH_2OCH_2)_3CH_2OMe$

**Figure S62.** Sketch of the ligand $\mathbf{L^{PEG3}}$.

## 7. Turbidimetry Experiments

Turbidimetry experiments were performed on an Implen C40 Nanophotometer® using the preprogrammed Kinetics application and all spectra were collected using Eppendorf UVette® cuvettes with a 2 mm path length. Jacalin was dissolved in HEPES buffer (10 mM, 0.1 mM $CaCl_2$, pH 8.5) and *Ulex europaeus* agglutinin (UEA-I) was dissolved in HEPES buffer (10 mM, 0.15 M NaCl, 2 mM $CaCl_2$, 0.2 mM $MnCl_2$, pH 7.4). Glycan cages were dissolved in either HEPES buffer (10 mM, 0.1 mM $CaCl_2$, pH 8.5) or HEPES buffer (10 mM, 0.15 M NaCl, 2 mM $CaCl_2$, 0.2 mM $MnCl_2$, pH 7.4) depending on the experiment. All lectin and glycan cage solutions were filtered through a Fisherbrand™ 0.2 µm filter and centrifuged for 20 mins at 12,500 rpm. Lectin and glycan cage concentrations were measured using the preprogrammed Protein UV or Wavescan applications on an Implen C40 Nanophotometer®. The exact concentration of Jacalin was determined by UV/vis spectroscopy by measuring the absorbance at 280 nm ($\varepsilon$ = 109,560 $M^{-1}$ $cm^{-1}$ with respect to the tetramer, $M_w$ 66.18 KDa).[9,10] The exact concentration of UEA-I was determined by UV/vis spectroscopy by measuring the absorbance at 280 nm ($\varepsilon$ = 79,090 $M^{-1}$ $cm^{-1}$ with respect to the dimer, $M_w$ 53.21 KDa).[10,11] Experiments were conducted with a solution of tetrameric Jacalin (10 µM with respect to the tetramer), dimeric UEA-I (10 µM with respect to the dimer) and solutions of glycan cages (50-500 µM). For each experiment, an initial UV/vis spectrum of the solution of Jacalin or UEA-I (100 µL) was collected and a solution of glycan cage was added (10 µL). The resulting mixture was quickly mixed and the absorbance of the mixture was then recorded at 420 nm every 60 seconds for a period of 30 minutes. A solution of an excess of D-galactose or L-fucose (4 µL, 1M) was then added. The resulting mixture was quickly mixed and the absorbance recorded every 60 seconds for a further 30 minutes.

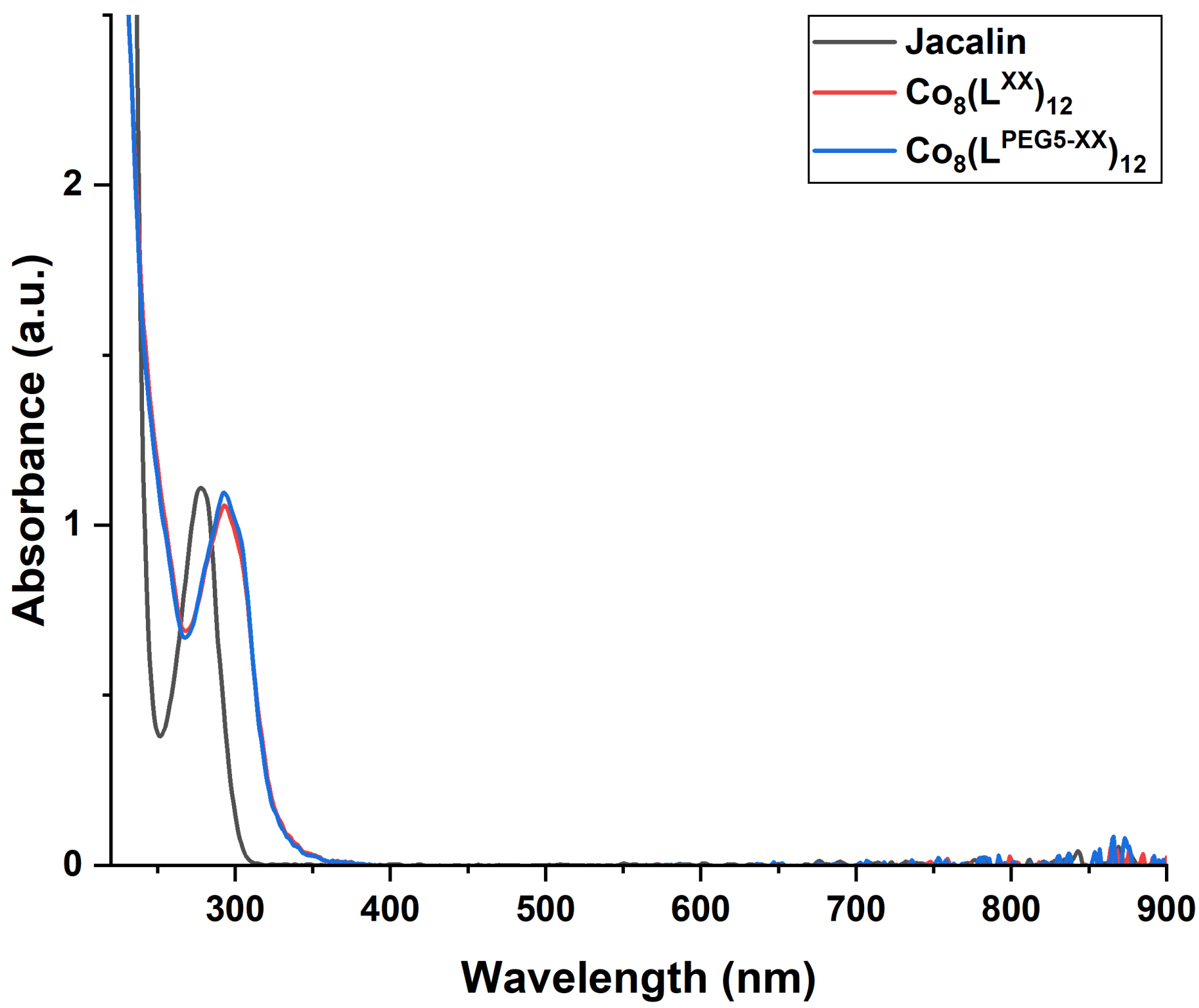


**Figure S63.** Absorbance spectra of $\mathbf{Co_8(L^{XX})_{12}}$ (4 µM, $\lambda_{max}$ 293), $\mathbf{Co_8(L^{PEG5\text{-}XX})_{12}}$ (4 µM, $\lambda_{max}$ 293) and Jacalin (10 µM, $\lambda_{max}$ 280) in HEPES buffer (10 mM, 0.1 mM $CaCl_2$, pH 8.5).

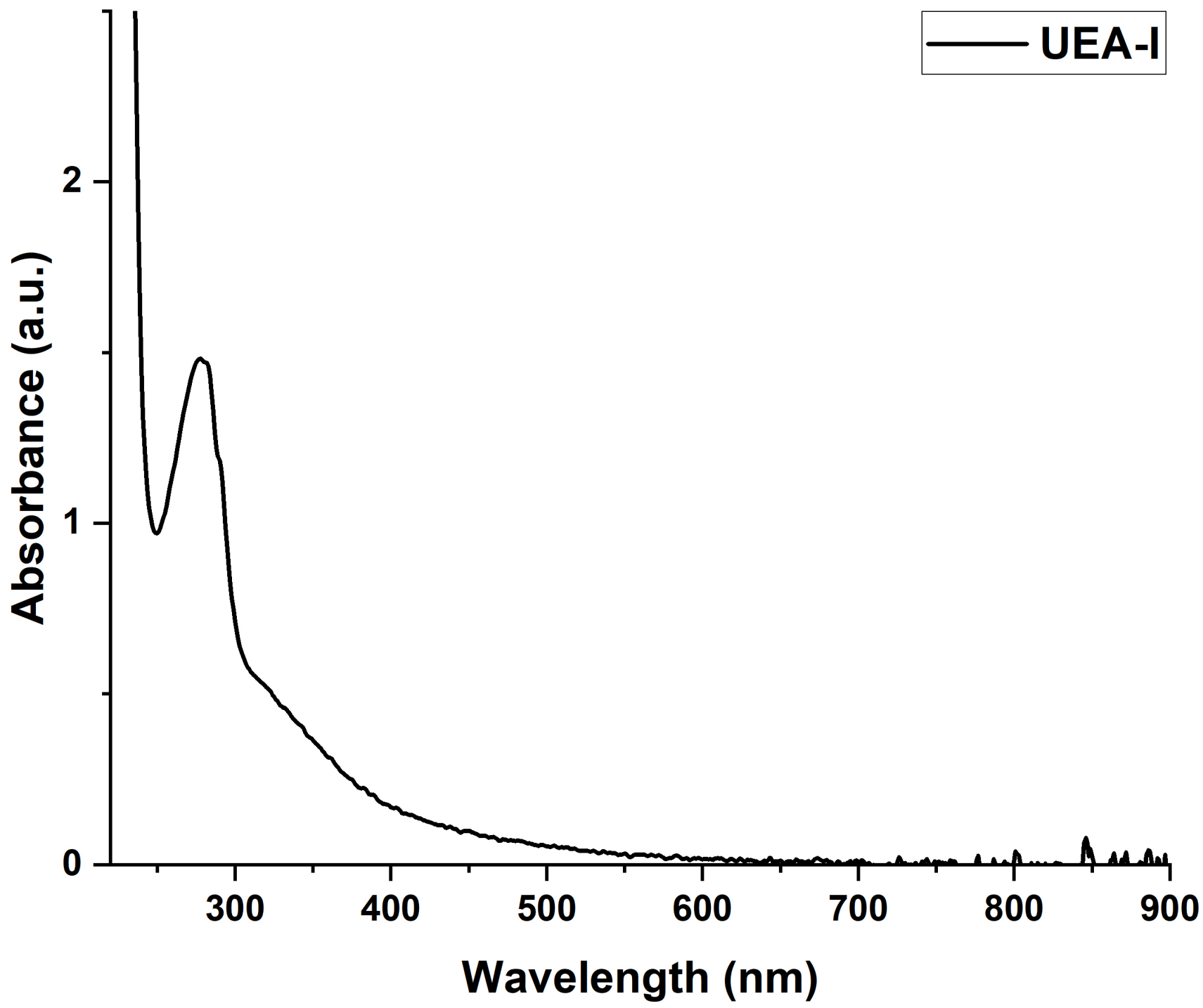


**Figure S64.** Absorbance spectra of UEA-I (10 µM, $\lambda_{max}$ 280) in HEPES buffer (10 mM, 0.15 M NaCl, 2 mM $CaCl_2$, 0.2 mM $MnCl_2$, pH 7.4).

## 8. References


1. C. Pritchard, M. Ligorio, G. D. Jackson, et al., *ACS Appl. Mater. Interfaces.*, 2023, **15**, 36052-36060.
2. G. D. Jackson, M. B. Tipping, C. G. P. Taylor, et al., *Chemistry*, 2021, **3**, 1203-1214.
3. S. Turega, M. Whitehead, B. R. Hall, et al., *Inorg. Chem.* 2013, **52**, 2, 1122-1132.
4. M. Yu, W. Zhang, Z. Guo, et al., *Angew. Chem. Int. Ed.*, 2021, **60**, 15590-15597.
5. L. Ma, C. J. E. Haynes, A. B. Grommet, et al., *Nat. Chem.*, 2020, **12**, 270-27.
6. A. Varki, R. D. Cummings, M. Aebi, et al., *Glycobiology*, 2015, **25**, 1323-1324.
7. K. Adelhorst and G. M. Whitesides, *Carbohydr. Res.*, 1993, **242**, 69-76.
8. V. Percec, P. Leowanawat, H.-J. Sun, et al., *J. Am. Chem. Soc*, 2013, **135**, 9055-9077.
9. R. Sankaranarayanan, K. Sekar, R. Banerjee, et al., *Nat. Struct. Mol. Biol.*, 1996, **3**, 596-603.
10. D. Batabyal, H. Lord, B. Ahlstrom, et al., *Biologicals*, 2021, **71**, 42-47.
11. G. F. Audette, M. Vandonselaar and L. T. J. Delbaere, *J. Mol. Biol.*, 2000, **304**, 423-433.
